# Supplementary material for: Deep learning finds convergent melanocytic morphology despite noisy archival slides
Source: Cell Rep Methods. 2025 Oct 20;5(10):101201. doi: 10.1016/j.crmeth.2025.101201 (PMC12570353; doi:10.1016/j.crmeth.2025.101201)
Supplement: Document S1. Figures S1–S6 and Tables S1–S4 [file mmc1.pdf]

**Cell Reports Methods, Volume *5***

## Supplemental information

# Deep learning finds convergent melanocytic morphology despite noisy archival slides

**Mikio Tada, Garrett Gaskins, Sina Ghandian, Nicholas Mew, Michael James Keiser, and Elizabeth Sarah Keiser**

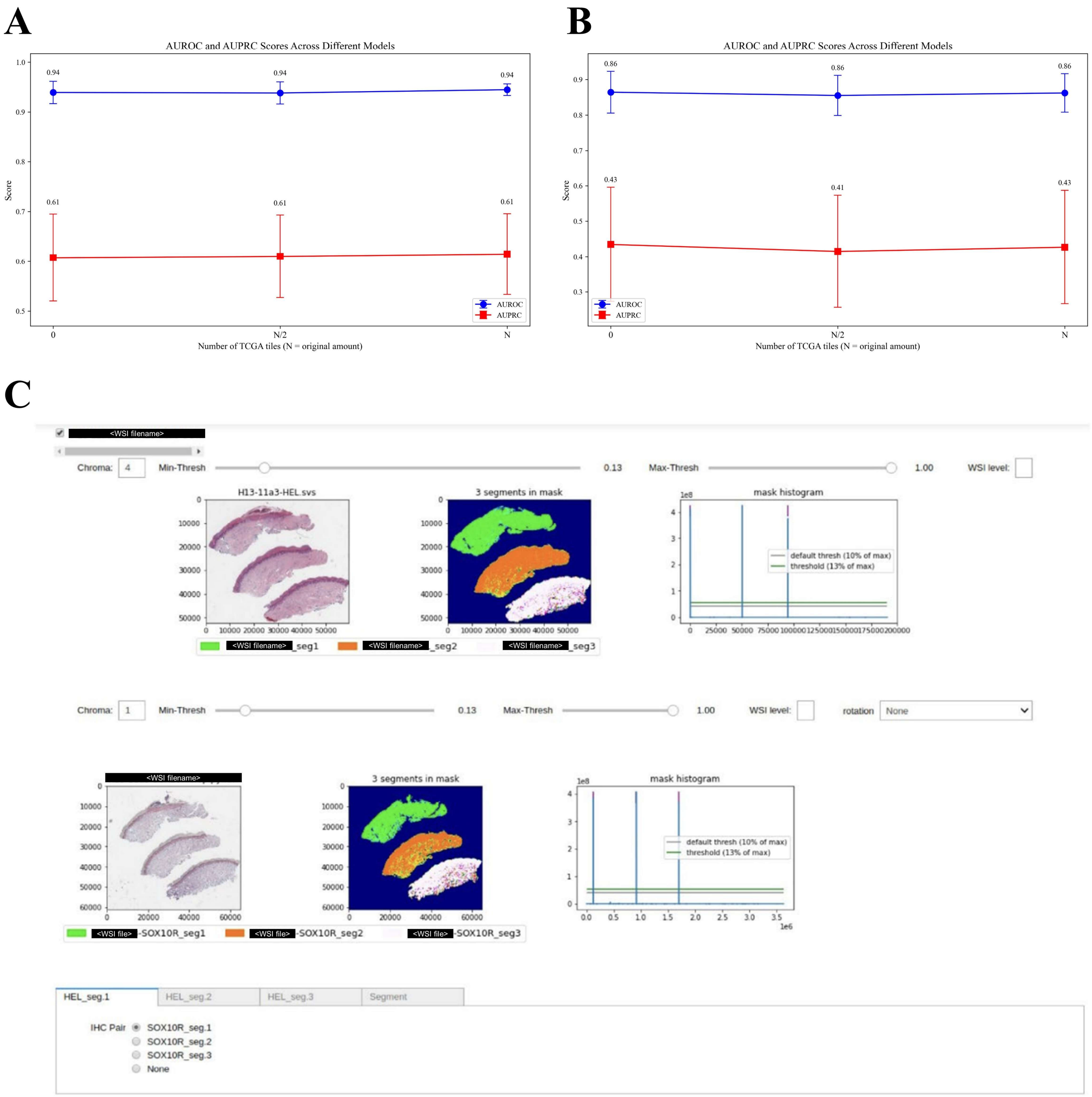


**Figure S1.** Impact of non-cutaneous TCGA tiles on model performance and automated tissue extraction toolkit, related to Figure 1 and STAR Methods. A) AUROC and AUPRC scores for MelanA models trained on varying amounts of TCGA tiles (0, N/2, N). In all cases, $n$ = 5 folds. B) AUROC and AUPRC scores for SOX10 models trained on varying amounts of TCGA tiles (0, N/2, N). $n$ = 5 folds. C) An example of a sample pair extracted using the automated toolkit. The toolkit visualizes both the H&E (top, left) and IHC (middle, left) whole slide images (WSIs) and extracts tissue regions according to threshold values set for each of the ipywidgets provided. Matching occurs automatically based on tissue locations in the WSI; the user can manually adjust it if necessary (bottom).

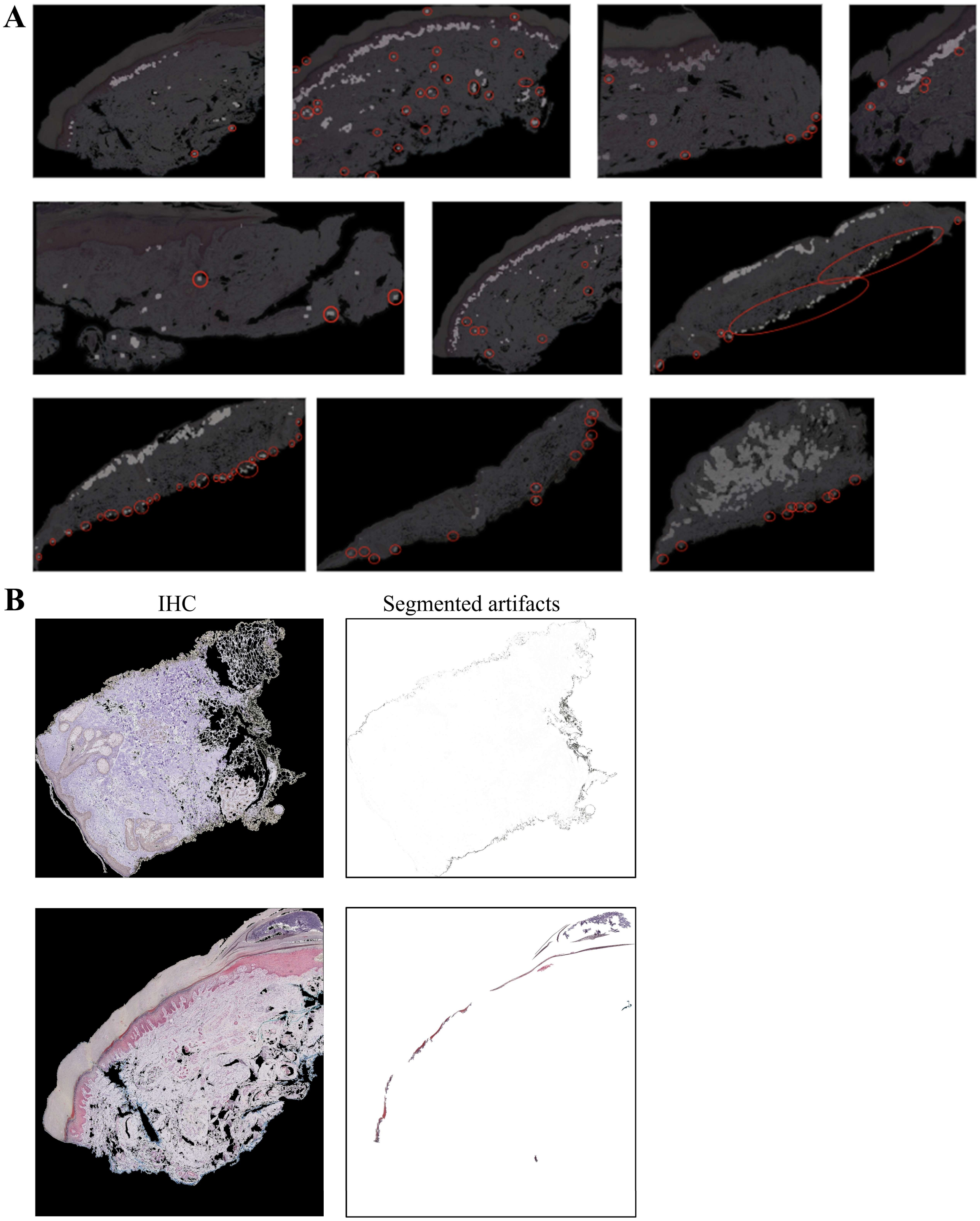


**Figure S2.** Examples of tissue artifacts computationally and manually removed from IHC WSIs, related to STAR Methods. A) Tissue artifacts that were computationally removed. The top row shows ink, and the bottom shows blood. B) Manual label correction by removal of false-positive areas. The labeling pipeline sometimes misidentified non-melanocyte regions as melanocytes due to tissue artifacts. To ensure label accuracy, we manually corrected these errors (red circles).

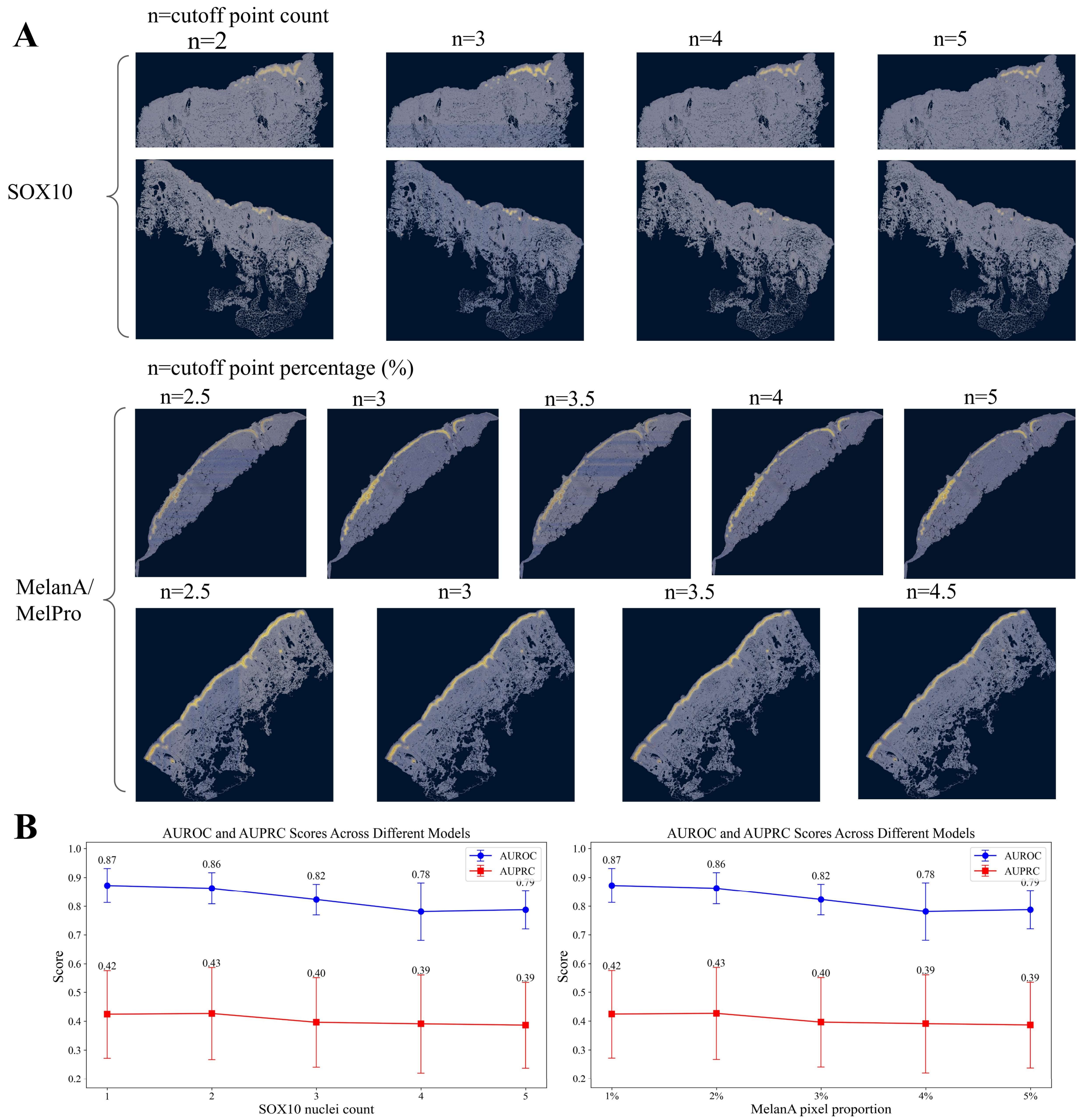


**Figure S3.** The effect of varying minimum signal thresholds on IHC "label maps" and model performance, related to Figures 2 and 3 and STAR Methods. A) "Label maps" using progressively increasing (more stringent) minimum-signal thresholds, *n*, for two SOX10 (top row) and two MelanA/MelPro (bottom row) WSIs. Label maps derive directly from positive IHC stain but are coarser-grained than the stain itself due to the 256x256 pixel tiles. B) Model performance evaluation across different training labels. AUROC and AUPRC scores for models trained with different SOX10 nuclei count thresholds (1-5) evaluated on the standard Test dataset (threshold=2 nuclei). *n*= 5 folds (left). AUROC and AUPRC scores for models trained on varying MelanA pixel proportion thresholds (1-5%) evaluated in all cases on the standard Test dataset (threshold=3%). *n* = 5 folds (right).

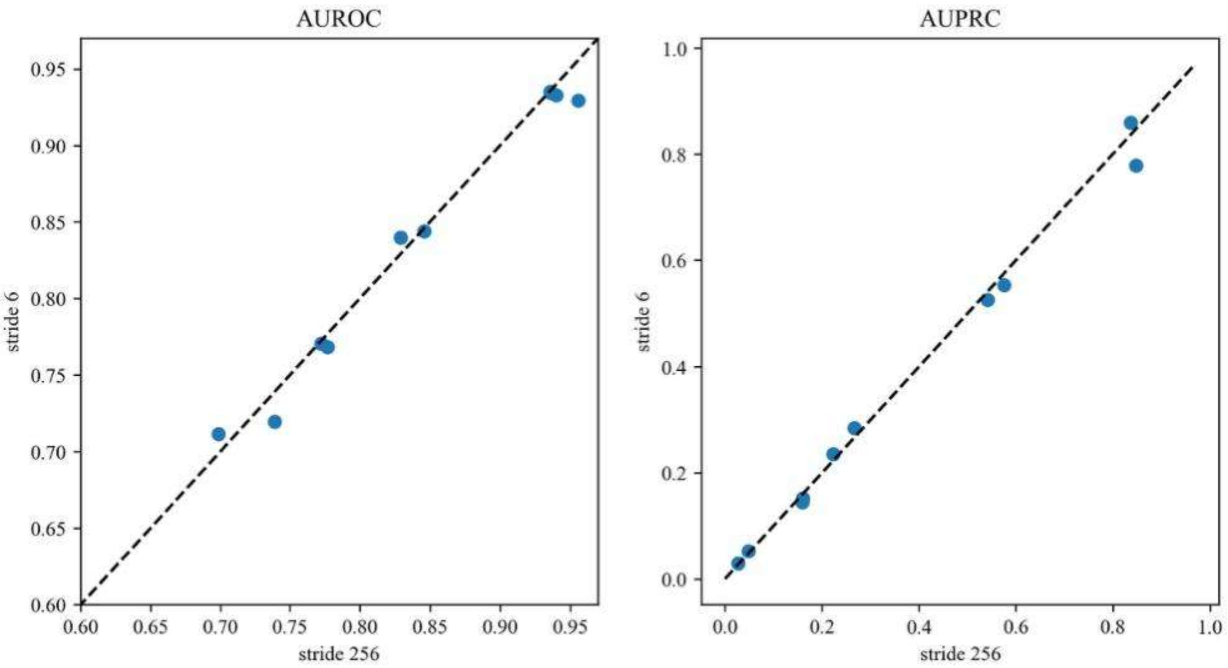


**Figure S4.** Comparison of performance calculated by AUROC score using stride 6-pixel (y-axis) versus 256-pixel (x-axis) strides on two test sets, related to STAR Methods. Each blue dot represents a tissue section. Stride 256-pixel calculations are much faster to compute due to the $n^2$ scaling of the heatmap calculation (where $n$ is proportional to 1/stride).

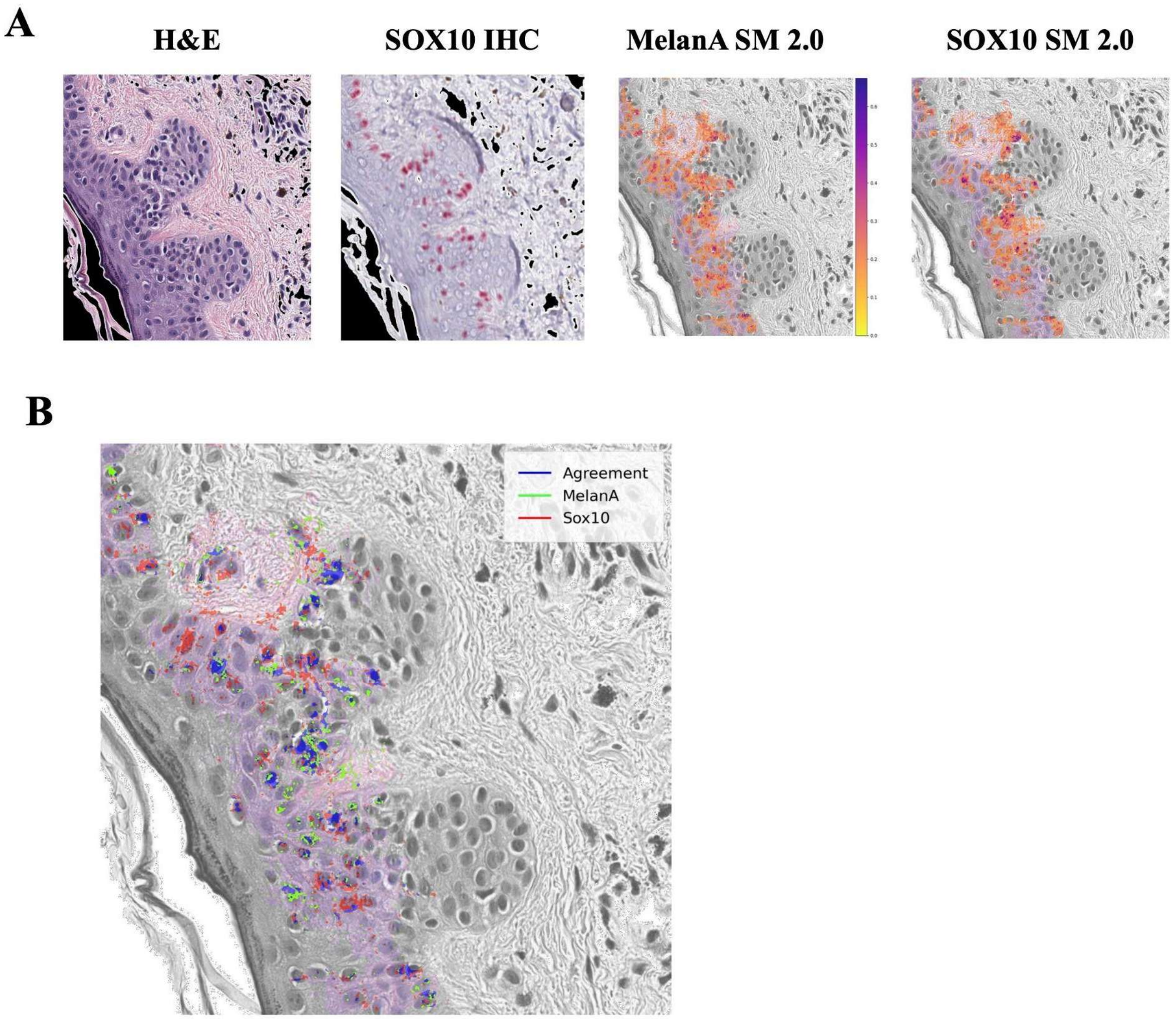


**Figure S5.** Recalculation of saliency maps for Figure 6b-c using a different attribution method, Integrated Gradients (IG), related to Figure 6 and STAR Methods. A) Saliency map comparison of MelanA and SOX10 models using IG. The IG method uses a different reasoning than Guided Grad-CAM to attribute salience to individual pixels, although it can be less intuitive when applied to images. Interestingly, the regions where independent IG calculations on each model converge on the same pixels (blue) qualitatively appear more coherent than those specific to either model alone (red, green). B) Agreement map comparing MelanA and SOX10 attributions on the same tissue region. Grayscale tissue denotes regions where the MelanA model confidence threshold is < 0.9. We only calculate attributions within the colored (high-confidence) region.

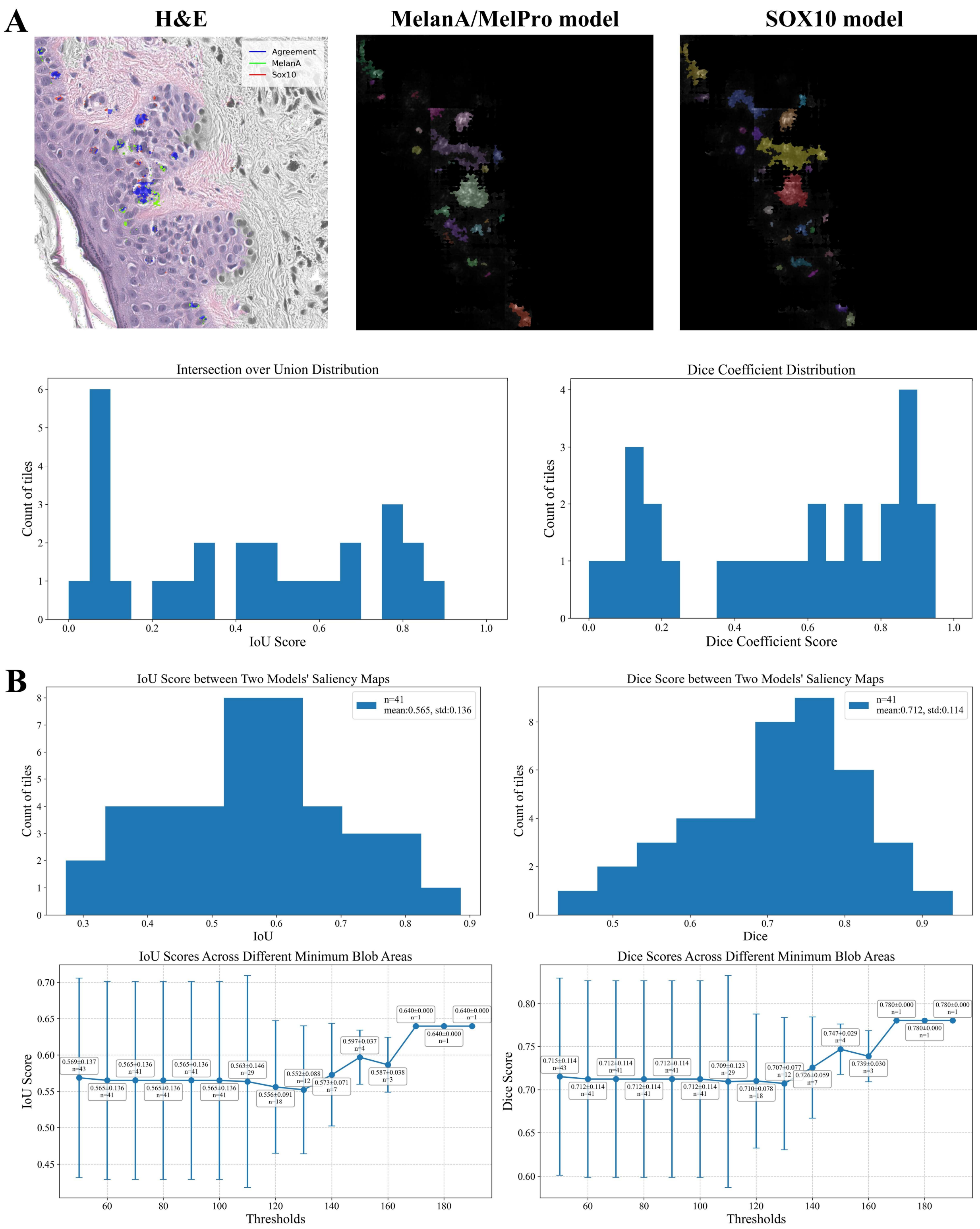

A
H&E
Agreement
MelanA
Sox10
MelanA/MelPro model
SOX10 model
Intersection over Union Distribution
Count of tiles
IoU Score
Dice Coefficient Distribution
Count of tiles
Dice Coefficient Score
B
IoU Score between Two Models' Saliency Maps
n=41
mean:0.565, std:0.136
Count of tiles
IoU
Dice Score between Two Models' Saliency Maps
n=41
mean:0.712, std:0.114
Count of tiles
Dice
IoU Scores Across Different Minimum Blob Areas
IoU Score
Thresholds
0.569±0.137 n=43
0.565±0.136 n=41
0.563±0.146 n=29
0.556±0.091 n=18
0.552±0.088 n=12
0.573±0.071 n=7
0.597±0.037 n=4
0.587±0.038 n=3
0.640±0.000 n=1
Dice Scores Across Different Minimum Blob Areas
Dice Score
Thresholds
0.715±0.114 n=43
0.712±0.114 n=41
0.709±0.123 n=29
0.710±0.078 n=18
0.707±0.077 n=12
0.726±0.059 n=7
0.747±0.029 n=4
0.739±0.030 n=3
0.780±0.000 n=1

**Figure S6.** Quantification of saliency maps similarity between MelanA/MelPro and SOX10 models at the region-of-interest, with an evaluation of different blob-size thresholds at the tile levels, related to Figure 6 and STAR Methods. A) Saliency map similarity at the region-of-interest. The region of H&E image with overlaid saliency maps (top left) from Figure 6c and visualization of individual saliency regions from MelanA/MelPro model (top middle) and SOX10 model (top right), with distinct colors representing distinct detected salient blobs. Histogram quantifying the similarity metrics between the two models' saliency maps. The bottom left panel shows the IoU distribution (0.428±0.286); the bottom right panel shows the Dice coefficient distribution (0.540±0.301). $n$ = 27 blobs identified at ≥100 pixel area each. B) Histogram of IoU scores (top left, 0.565±0.136) and Dice scores (top right, 0.712±0.114) at tile-level between two models' saliency maps. IoU (bottom left) and Dice (bottom right) scores across different choices of minimum pixel-area threshold (50-190px) used to determine candidate cross-stain blobs.

**SUPPLEMENTAL TABLES**

| WSI ID | Stain Type | Institution | # of Sections | Stain Color | Color Extracted |
|---|---|---|---|---|---|
| WSI-42 | SOX10 | UCSF | 2 | Red | Red |
| WSI-44 | SOX10 | UCSF | 2 | Red | Red |
| WSI-47 | SOX10 | UCSF | 3 | Red | Red |
| WSI-52 | SOX10 | Stanford | 6 | Red | Red |
| WSI-55 | SOX10 | Stanford | 2 | Red | Red |
| WSI-57 | SOX10 | Stanford | 1 | Brown | Brown |
| WSI-58 | SOX10 | Stanford | 1 | Brown | Brown |
| WSI-59 | SOX10 | Stanford | 4 | Brown | Brown |
| WSI-60 | SOX10 | Stanford | 2 | Red | Red |
| WSI-61 | SOX10 | Stanford | 3 | Red | Red |
| WSI-02 | MELA | UCSF | 4 | Red | Red |
| WSI-03 | MELA | UCSF | 2 | Red | Red |
| WSI-06 | MELA | UCSF | 7 | Red | Red |
| WSI-07 | MELA | UCSF | 4 | Red | Red |
| WSI-09 | MELA | UCSF | 1 | Red | Red |
| WSI-12 | MELA | UCSF | 2 | Red | Red |
| WSI-13 | MELA | UCSF | 3 | Red | Red |
| WSI-14 | MELA | UCSF | 5 | Red | Red |
| WSI-15 | MELA | UCSF | 1 | Red | Red |
| WSI-16 | MELA | UCSF | 3 | Red | Red |
| WSI-28 | MelPro | UCSF | 2 | Red (cytoplasmic) + Brown (nuclear) | Red |
| WSI-30 | MelPro | Stanford | 1 | Red (cytoplasmic) + Brown (nuclear) | Red |
| WSI-32 | MelPro | Stanford | 2 | Red (cytoplasmic) + Brown (nuclear) | Red |

**Table S1.** Overview of whole slide images used to train and evaluate the models, related to STAR Methods.

| WSI ID | Stain Type | Institution | Sections (HE) | Sections (IHC) | Stain Color | Preview Notes | Action | Alignment Status |
|---|---|---|---|---|---|---|---|---|
| WSI-01 | MELA | UCSF | 6 | 6 | Red | Tissue itself is strange. Previously removed | Not Used | Not Aligned |
| WSI-02 | MELA | UCSF | 8 | 8 | Red | 3rd level image damaged -- damage may impact alignment preview | Used | Aligned |
| WSI-03 | MELA | UCSF | 6 | 2 | Red | 6 slices compared to 2 rotated slices -- 3rd slice on top removed bc of bad alignment | Used | Aligned |
| WSI-04 | MELA | UCSF | 2 | 1 | Red | Good -- can't preview due to huge size disparity -- going to assume at highest res it will work out -- **Have to manually add params | Attempted | Aligned Poorly |
| WSI-05 | MELA | UCSF | 3 | 3 | Red | Iffy -- 3rd pairing is mismatch at equal sizing so removed -- other two may align, but not likely to very well | Not Used | Not Aligned |
| WSI-06 | MELA | UCSF | 8 | 8 | Red | Good | Used | Aligned |
| WSI-07 | MELA | UCSF | 5 | 5 | Red | Good -- keeping segment 1 | Used | Aligned |
| WSI-08 | MELA | UCSF | 8 | 4 | Red | IHC images on wildly larger scale than HE -- can't preview -- can't ECC align | Not Used | Not Aligned |
| WSI-09 | MELA | UCSF | 8 | 8 | Red | Good -- skip first 2 smallest segments as they are difficult to align | Used | Aligned |
| WSI-10 | MELA | UCSF | 8 | 8 | Red | Good -- remove bottom 2 segments as cannot ECC align between HE and IHC | Attempted | Aligned Poorly |
| WSI-11 | MELA | UCSF | 4 | 4 | Red | Good -- likely too big for opencv -- skip for now and use previous alignments | Attempted | Aligned Poorly |
| WSI-12 | MELA | UCSF | 4 | 2 | Red | Good | Used | Aligned |
| WSI-13 | MELA | UCSF | 12 | 12 | Red | Good -- exclude bottom 2 segments by size -- removed section 10-9 HE-IHC for bad alignment | Used | Aligned |
| WSI-14 | MELA | UCSF | 8 | 8 | Red | Good | Used | Aligned |
| WSI-15 | MELA | UCSF | 18 | 6 | Red | Good -- removed bottom slice from each trio (total of 6 slices) due to bad segmentation | Used | Aligned |
| WSI-16 | MELA | UCSF | 6 | 6 | Red | May not be able to align due to size disparity | Used | Aligned |
| WSI-17 | MELA | UCSF | 10 | 10 | Red | Good -- ignore middle 2 slices for all pairings | Attempted | Aligned Poorly |
| WSI-18 | MELA | UCSF | 6 | 2 | Red | Poor pairing -- can't preview -- likely can't align | Not Used | Not Aligned |
| WSI-19 | MELA | UCSF | 5 | 5 | Red | Good -- remove 5<->5 | Attempted | Aligned Poorly |
| WSI-20 | MELA | Stanford | 3 | 3 | Brown | Good | Not Used | Aligned |
| WSI-21 | MELA | Stanford | 3 | 3 | Brown | IHC sections are missing bottom half + angle disparity | Not Used | Not Aligned |
| WSI-22 | MELA | Stanford | 4 | 4 | Brown | IHC sections are missing bottom half + angle disparity | Not Used | Not Aligned |
| WSI-23 | MELA | Stanford | 2 | 2 | Brown | HE seg1 contains schlieren lines on right side -- Seg2 shapes different | Attempted | Aligned Poorly |
| WSI-24 | MELA | Stanford | 4 | 2 | Brown | IHC seg2 needs to be resampled | Not Used | Not Aligned |

| | | | | | | | | |
|---|---|---|---|---|---|---|---|---|
| WSI-25 | MELA | Stanford | 1 | 1 | Brown | Bad IHC stain | Not Used | Not Aligned |
| WSI-26 | MELA | Stanford | 1 | 1 | Brown | Good | Attem pted | Not Aligned |
| WSI-27 | MelPro | UCSF | 12 | 4 | Red | Good | Not Used | Not Aligned |
| WSI-28 | MelPro | UCSF | 18 | 6 | Red | Good -- bottom 3 IHCs are not attached to slide correctly -- applying only to top 3 | Used | Aligned |
| WSI-29 | MelPro | Stanford | 6 | 2 | Red | Good --alignments for pairs 1-3 may be difficult -- Copy in p16 | Attem pted | Not Aligned |
| WSI-30 | MelPro | Stanford | 3 | 3 | Red | Good -- Slight amount of IHC bottom missing in comparison to H&E | Used | Aligned |
| WSI-31 | MelPro | Stanford | 3 | 3 | Red | Good -- IHC 2 may be treated as 2 segments instead of 1 (examine whether chroma 0. vs 1. is necessary) | Not Used | Aligned |
| WSI-32 | MelPro | Stanford | 3 | 3 | Red | Good -- NA | Used | Aligned |
| WSI-33 | SOX10 | UCSF | 3 | 2 | Black | 3 small H&E slices provided. 2 slightly larger IHC slices -- can't perform alignment preview | Not Used | Not Aligned |
| WSI-34 | SOX10 | UCSF | 2 | 1 | Black | 2 small slices compared to 1 large rotated slice -- can't perform alignment preview | Not Used | Not Aligned |
| WSI-35 | SOX10 | UCSF | 2 | 1 | Black | 2 small slices compared to 1 large rotated slice -- can't perform alignment preview | Not Used | Not Aligned |
| WSI-36 | SOX10 | UCSF | 4 | 2 | Black | 4 slices compared to 2 larger rotated slices only -- can't perform alignment preview | Not Used | Not Aligned |
| WSI-37 | SOX10 | UCSF | 2 | 2 | Black | 2 small slices compared to 1 large *broken* rotated slice -- can't perform alignment preview | Not Used | Not Aligned |
| WSI-38 | SOX10 | UCSF | 2 | 1 | Black | 2 small slices compared to 1 large *broken* slice -- can't perform alignment preview | Not Used | Not Aligned |
| WSI-39 | SOX10 | UCSF | 2 | 1 | Black | 2 small slices compared to 1 large rotated slice -- can't perform alignment preview | Not Used | Not Aligned |
| WSI-40 | SOX10 | UCSF | 3 | 2 | Black | Multiple slices compared to 2 separated portion of the same section --can't perform alignment preview | Not Used | Not Aligned |
| WSI-41 | SOX10 | UCSF | 8 | 4 | Black | Tissue is poor/choppy | Not Used | Not Aligned |
| WSI-42 | SOX10 | UCSF | 3 | 3 | Red | Good | Used | Aligned |
| WSI-43 | SOX10 | UCSF | 8 | 4 | Red | Good -- remove bottom slice tissue broken from IHC -- **WSI throws an error for align upon chroma change. Blurry IHC. | Not Used | Aligned |
| WSI-44 | SOX10 | UCSF | 6 | 2 | Red | Good -- All alignments are likely as good as they will get, but not optimal due to fundamental differences in shape between HE and IHC excisions | Used | Aligned |
| WSI-45 | SOX10 | UCSF | 3 | 3 | Red | Iffy -- most segments are too differently shaped / sized to incorporate -- May need to use original images rather than ECC | Not Used | Not Aligned |
| WSI-46 | SOX10 | UCSF | 12 | 12 | Brown | Good -- STAIN (GREY/BROWN) IS | Not | Aligned |

| | | | | | | | | |
|---|---|---|---|---|---|---|---|---|
| | | | | | | DIFFERENT THAN ORIGINAL LABEL. Negative control. | Used | |
| WSI-47 | SOX10 | UCSF | 6 | 6 | Red | Good -- does not perform well on ECC alignment | Used | Aligned |
| WSI-48 | SOX10 | UCSF | 12 | 4 | Red | Poor pairing and can't tell if slice is worthwhile | Not Used | Not Aligned |
| WSI-49 | SOX10 | UCSF | 8 | 8 | Red | Good | Attem pted | Aligned Poorly |
| WSI-50 | SOX10 | UCSF | 6 | 6 | Red | Good -- stain somewhat light | Attem pted | Aligned Poorly |
| WSI-51 | SOX10 | Stanford | 3 | 3 | Brown | Spurious -- Slices are huge and IHC stain is hard to separate from BG -- make sure masks are correctly matched | Attem pted | Aligned Poorly |
| WSI-52 | SOX10 | Stanford | 3 | 3 | Red | COPY of slices in MELA -- HE images seem like bigger area | Used | Aligned |
| WSI-53 | SOX10 | Stanford | 4 | 3 | Red | COPY of slices in MELA -- Could be hard to align if we don't fix angle disparity | Attem pted | Aligned Poorly |
| WSI-54 | SOX10 | Stanford | 4 | 4 | Brown | Good | Attem pted | Aligned Poorly |
| WSI-55 | SOX10 | Stanford | 4 | 4 | Red | Good | Used | Aligned |
| WSI-56 | SOX10 | Stanford | 9 | 3 | Red | Good | Attem pted | Aligned Poorly |
| WSI-57 | SOX10 | Stanford | 1 | 1 | Brown | Good -- See if difference in angle causes alignment to fail | Used | Aligned |
| WSI-58 | SOX10 | Stanford | 1 | 1 | Brown | Good | Used | Aligned |
| WSI-59 | SOX10 | Stanford | 4 | 4 | Brown | Good | Used | Aligned |
| WSI-60 | SOX10 | Stanford | 3 | 3 | Red | Good | Used | Aligned |
| WSI-61 | SOX10 | Stanford | 3 | 3 | Red | Good - Yellow smudge may cause problem with matches… make sure they are correct | Used | Aligned |

**Table S2.** MelanA, MelPro, and SOX10 whole slide image dataset metadata and manual inspection notes, related to STAR Methods. Slides with action column “Used” were included in the calculations; “Not Used” and “Attempted” were discarded.

| TCGA WSI SVS Filename | Tissue Source |
|---|---|
| TCGA-22-4594-01Z-00-DX1.3FCEBC89-8473-4841-87A2-F84AF58A7793.svs | Lung Squamous Cell Carcinoma |
| TCGA-2J-AABA-01Z-00-DX1.93B2B4EF-C302-4D00-ABE3-4862ACC81659.svs | Pancreatic Adenocarcinoma |
| TCGA-2J-AABK-01Z-00-DX1.AF5DE1FD-40EE-4149-8918-B53EC2DF727E.svs | Pancreatic Adenocarcinoma |
| TCGA-3A-A9I7-01Z-00-DX1.23EE4A93-A298-4522-837E-3EE10172D66C.svs | Pancreatic Adenocarcinoma |
| TCGA-3A-A9IB-01Z-00-DX1.77855A18-9E12-4F6A-8FBB-B5057656C493.svs | Pancreatic Adenocarcinoma |
| TCGA-3A-A9IH-01Z-00-DX1.578316D1-186E-4AE4-BD6A-DA426DE87829.svs | Pancreatic Adenocarcinoma |
| TCGA-3A-A9IL-01Z-00-DX1.BEB57CA5-223D-4330-BFFF-8202DCC857F3.svs | Pancreatic Adenocarcinoma |
| TCGA-3A-A9IN-01Z-00-DX1.A4FED037-D993-4F71-B422-14FC4E468B4C.svs | Pancreatic Adenocarcinoma |
| TCGA-3A-A9J0-01Z-00-DX1.322C8475-A1E3-4877-B3B5-921FDDB9698F.svs | Pancreatic Adenocarcinoma |
| TCGA-43-A56V-01Z-00-DX1.AA93FE03-FA7D-42C4-A118-B98C2400D9DA.svs | Lung Squamous Cell Carcinoma |
| TCGA-60-2722-01Z-00-DX1.f3781266-e8dc-4386-9702-5b29e6f2cfa3.svs | Lung Squamous Cell Carcinoma |
| TCGA-66-2742-01Z-00-DX1.8fdd6990-a08c-457b-80e4-586c619a784e.svs | Lung Squamous Cell Carcinoma |
| TCGA-A1-A0SE-01Z-00-DX1.04B09232-C6C4-46EF-AA2C-41D078D0A80A.svs | Breast Invasive Carcinoma |
| TCGA-A2-A04U-01Z-00-DX1.06D17357-46A8-4DC3-A22B-2F4EB6EE3F79.svs | Breast Invasive Carcinoma |
| TCGA-A2-A0CZ-01Z-00-DX1.A433A414-4F1B-4F99-8FD9-E64803F5E042.svs | Breast Invasive Carcinoma |
| TCGA-A8-A09R-01Z-00-DX1.392580F3-0CE5-4EDB-91CF-814AAD0DB649.svs | Breast Invasive Carcinoma |
| TCGA-AO-A0JC-01Z-00-DX1.C8DD421B-9799-4FE7-9224-5EAC6ED1028E.svs | Breast Invasive Carcinoma |
| TCGA-AO-A1KQ-01Z-00-DX1.CAB7D9A5-7030-4A33-BE51-9B04D67A7676.svs | Breast Invasive Carcinoma |
| TCGA-AR-A1AX-01Z-00-DX1.2389D54F-545E-499E-B392-DD731834460A.svs | Breast Invasive Carcinoma |
| TCGA-BH-A0DI-01Z-00-DX1.6A42D535-8842-4C36-8299-A40E9E56759D.svs | Breast Invasive Carcinoma |
| TCGA-D8-A1XS-01Z-00-DX2.ED8BBDB4-CEA6-4E47-8214-4666F3CC6E44.svs | Breast Invasive Carcinoma |
| TCGA-E9-A22D-01Z-00-DX1.b2867437-0add-4b7d-8002-fb09ed961942.svs | Breast Invasive Carcinoma |
| TCGA-F2-A44H-01Z-00-DX1.98C75E19-10DE-434A-AF1B-CDD182F6EDD5.svs | Pancreatic Adenocarcinoma |
| TCGA-F2-A7TX-01Z-00-DX1.2FB4B966-3F76-4BB7-B1E8-D6F651665479.svs | Pancreatic Adenocarcinoma |
| TCGA-FB-A4P5-01Z-00-DX1.D5440110-D217-4B4C-A8D2-7261B430F440.svs | Pancreatic Adenocarcinoma |
| TCGA-FB-A78T-01Z-00-DX1.1DC04A89-2428-489B-A70E-0D9C6D2A5E61.svs | Pancreatic Adenocarcinoma |
| TCGA-H6-A45N-01Z-00-DX1.80D3E1A9-02EB-4897-9632-F6FC00B3FA0F.svs | Pancreatic Adenocarcinoma |
| TCGA-HN-A2OB-01Z-00-DX1.14F1FBFB-4540-43CE-9D79-5BC628640424.svs | Breast Invasive Carcinoma |
| TCGA-HV-A5A4-01Z-00-DX1.00C72860-A4C4-41FB-87BA-7C4381FAF2BD.svs | Pancreatic Adenocarcinoma |

| | |
|---|---|
| TCGA-HZ-8005-01Z-00-DX1.e49bbccf-eab2-4f2f-b882-406b90fb2020.svs | Pancreatic Adenocarcinoma |
| TCGA-HZ-8315-01Z-00-DX1.F6B3F80E-3630-426E-AB2C-7F2EC5B63BFC.svs | Pancreatic Adenocarcinoma |
| TCGA-HZ-8317-01Z-00-DX1.BD28612C-D35D-4664-8B88-A85EF99013AB.svs | Pancreatic Adenocarcinoma |
| TCGA-HZ-8317-01Z-00-DX2.FDB366FF-AAA9-4FDC-A1F4-BA021904ED94.svs | Pancreatic Adenocarcinoma |
| TCGA-HZ-8637-01Z-00-DX1.5943021F-C94B-4CED-B45F-7A288F7188E0.svs | Pancreatic Adenocarcinoma |
| TCGA-HZ-8638-01Z-00-DX1.AD9F30CA-8943-493E-8603-7D1CF41056E6.svs | Pancreatic Adenocarcinoma |
| TCGA-HZ-A77O-01Z-00-DX1.C0F88C8F-C68C-457B-A0FF-3B483FCE7385.svs | Pancreatic Adenocarcinoma |
| TCGA-IB-7644-01Z-00-DX1.A2E77093-90D2-4ED3-90EB-F14A03C3DA57.svs | Pancreatic Adenocarcinoma |
| TCGA-IB-8127-01Z-00-DX1.C7035E56-9D24-4EEA-A09E-8276382193CC.svs | Pancreatic Adenocarcinoma |
| TCGA-IB-A5SS-01Z-00-DX1.899575C7-D239-4A04-8827-044F0D8868C8.svs | Pancreatic Adenocarcinoma |
| TCGA-IB-AAUV-01Z-00-DX1.045691CD-E0F8-4992-BF49-43AF7F83C97A.svs | Pancreatic Adenocarcinoma |
| TCGA-NC-A5HT-01Z-00-DX1.9295B0E3-37FE-4914-AFB3-78B56C893B6D.svs | Lung Squamous Cell Carcinoma |
| TCGA-OL-A97C-01Z-00-DX1.BDEEDEE2-6D07-4046-A8A9-D6FF8F337393.svs | Breast Invasive Carcinoma |
| TCGA-US-A774-01Z-00-DX1.522FF138-153F-488A-BBBC-5EA68EFD80C7.svs | Pancreatic Adenocarcinoma |
| TCGA-UU-A93S-01Z-00-DX1.C4809779-DF5F-4F5D-A78C-B7F95F2D050F.svs | Breast Invasive Carcinoma |
| TCGA-Z5-AAPL-01Z-00-DX1.30371C08-9075-44A9-8ED7-560256D65A7C.svs | Pancreatic Adenocarcinoma |
| **Tissue Source** | **WSI Count** |
| Pancreatic Adenocarcinoma | 27 |
| Breast Invasive Carcinoma | 13 |
| Lung Squamous Cell Carcinoma | 5 |

**Table S3.** TCGA identifiers for SVS whole slide images and summary of tissue sources for negative-control TCGA non-skin H&E tiles used in model training, related to STAR Methods.

| Fold | Train and Validation | | Test | |
|---|---|---|---|---|
| | Positive | Negative | Positive | Negative |
| MelanA 1 | 16,214 | 355,753 | 1,025 | 20,924 |
| MelanA 2 | 15,034 | 354,610 | 800 | 9,422 |
| MelanA 3 | 13,102 | 317,536 | 1,225 | 20,614 |
| MelanA 4 | 15,281 | 383,511 | 855 | 12,648 |
| MelanA 5 | 12,712 | 329,430 | 1,505 | 16,186 |
| Sox10 1 | 8,788 | 282,041 | 1,099 | 20,507 |
| Sox10 2 | 8,758 | 333,967 | 958 | 8,926 |
| Sox10 3 | 8,141 | 283,539 | 489 | 14,778 |
| Sox10 4 | 10,278 | 283,860 | 407 | 5,454 |
| Sox10 5 | 8,432 | 269,898 | 389 | 8,517 |

**Table S4.** Breakdown of positive and negative tile counts for each fold, related to Figures 2 and 3 and STAR Methods.
