# Supplementary figures and images for: Deep learning finds convergent melanocytic morphology despite noisy archival slides (part 2 of 2)
Source: Cell Rep Methods. 2025 Oct 20;5(10):101201. doi: 10.1016/j.crmeth.2025.101201 (PMC12570353; doi:10.1016/j.crmeth.2025.101201)

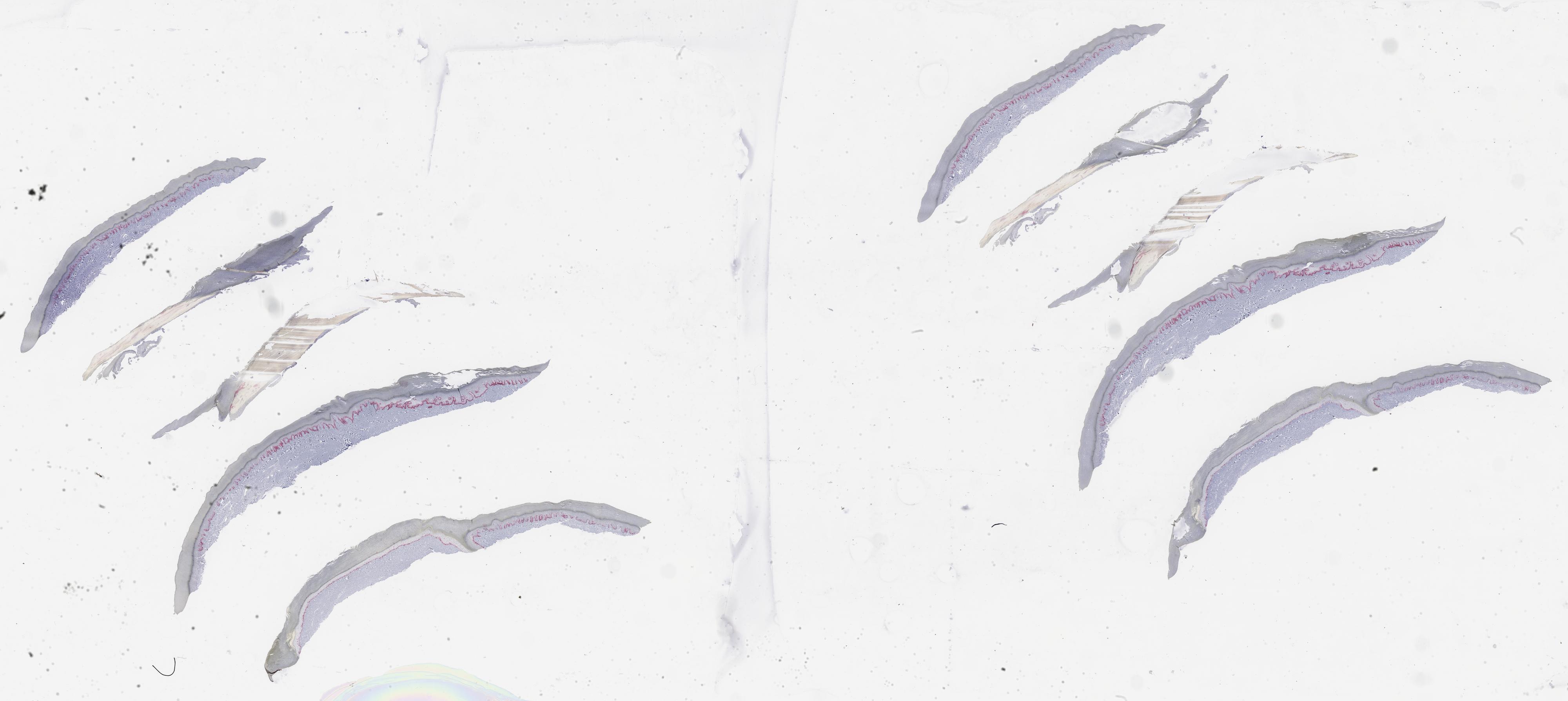

Supplement: Data S1. Illustrative low-resolution summary views of archival H&E-IHC whole slide image pairs, related to STAR Methods and Figure 1 — Details available in Tables S1 and S2. [file mmc2.zip › WSI-17_IHC.jpg]

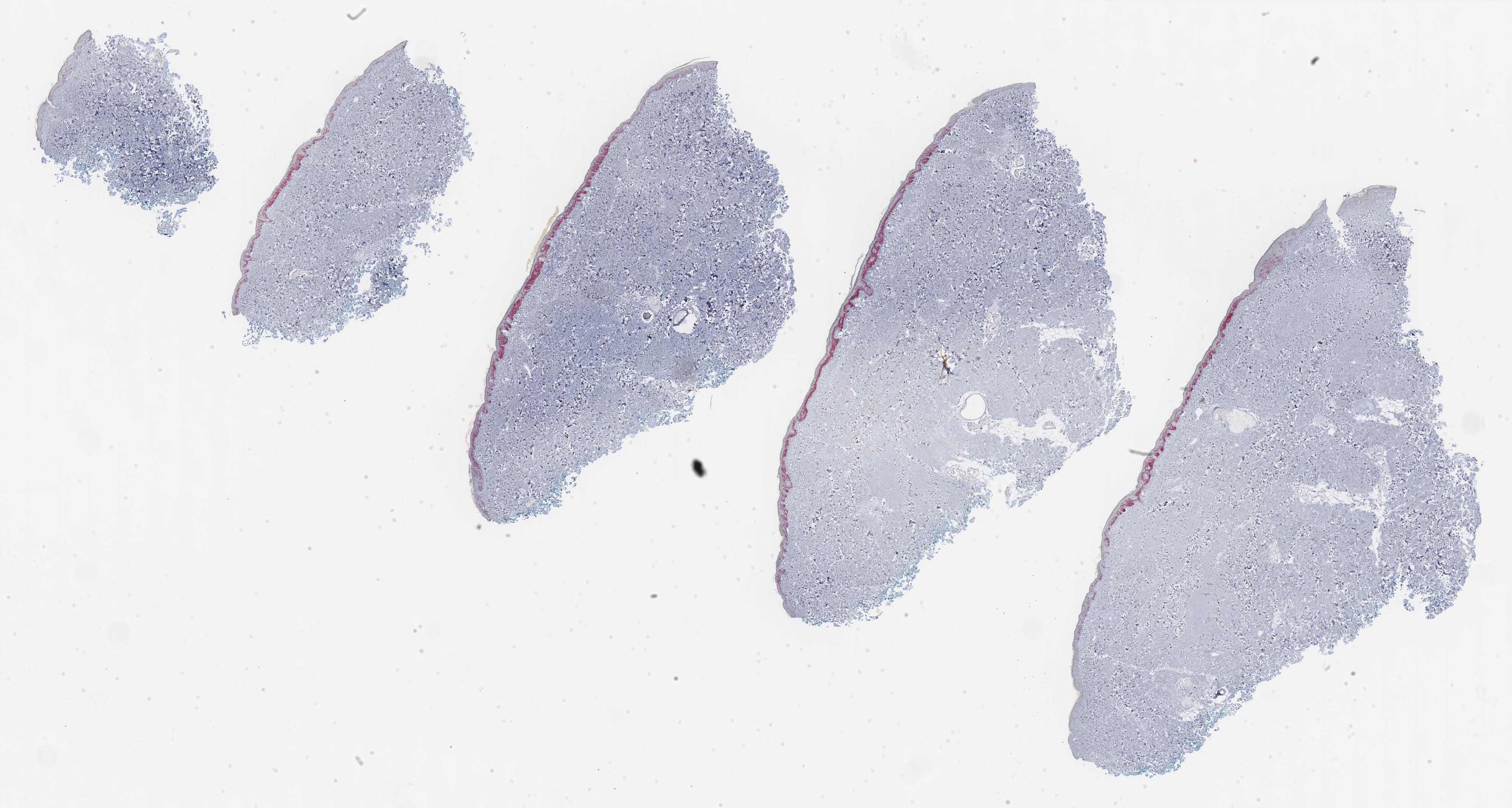

Supplement: Data S1. Illustrative low-resolution summary views of archival H&E-IHC whole slide image pairs, related to STAR Methods and Figure 1 — Details available in Tables S1 and S2. [file mmc2.zip › WSI-07_IHC.jpg]

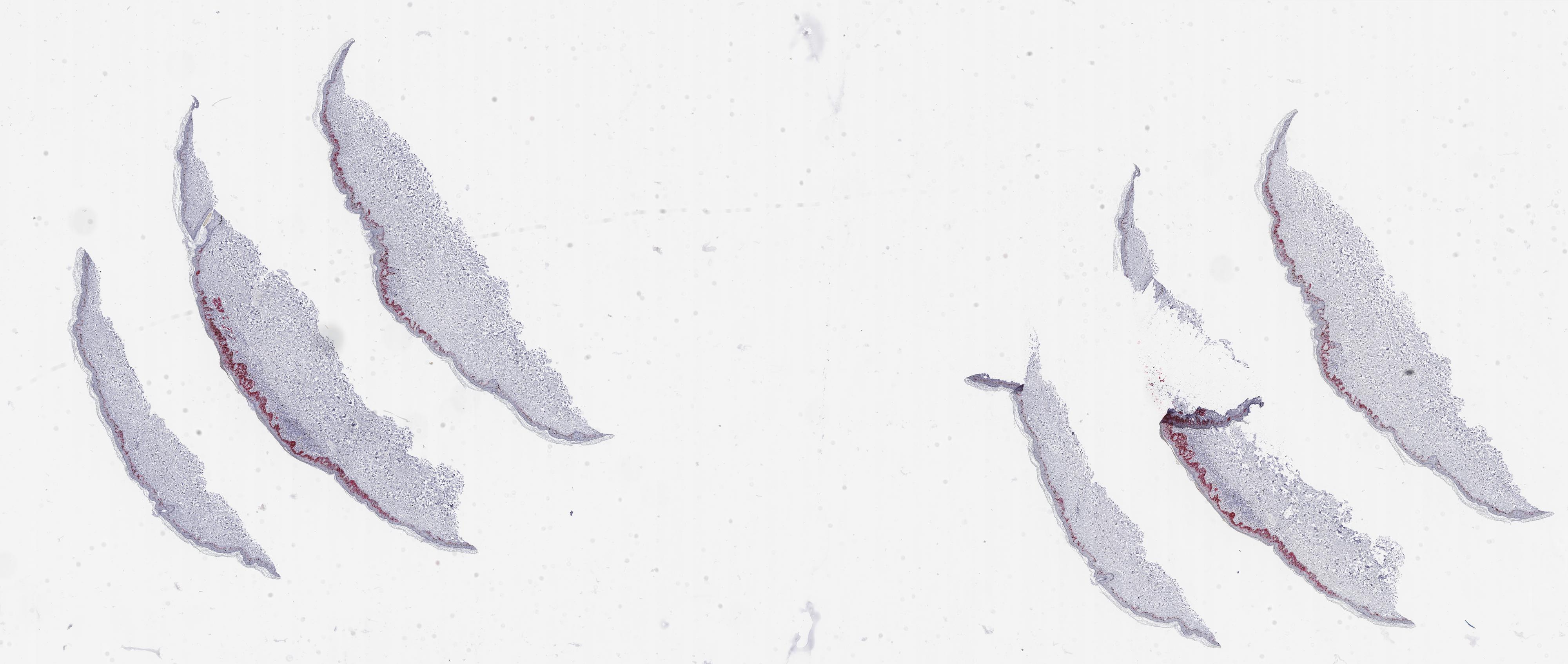

Supplement: Data S1. Illustrative low-resolution summary views of archival H&E-IHC whole slide image pairs, related to STAR Methods and Figure 1 — Details available in Tables S1 and S2. [file mmc2.zip › WSI-28_IHC.jpg]

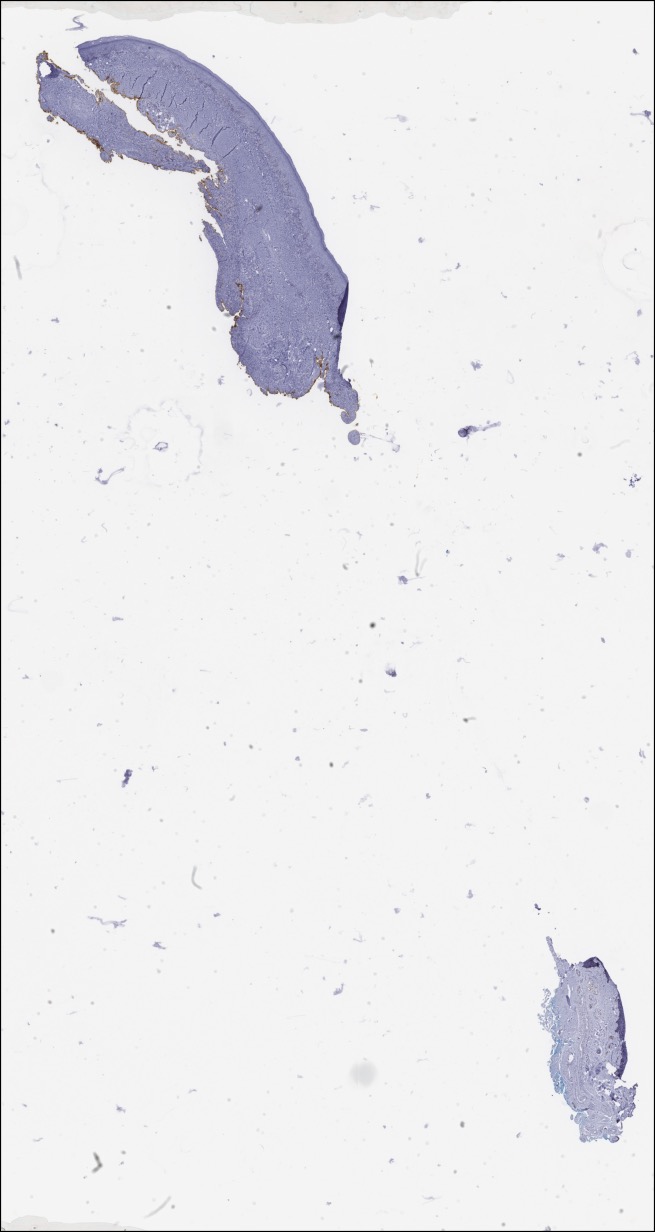

Supplement: Data S1. Illustrative low-resolution summary views of archival H&E-IHC whole slide image pairs, related to STAR Methods and Figure 1 — Details available in Tables S1 and S2. [file mmc2.zip › WSI-38_IHC.jpg]

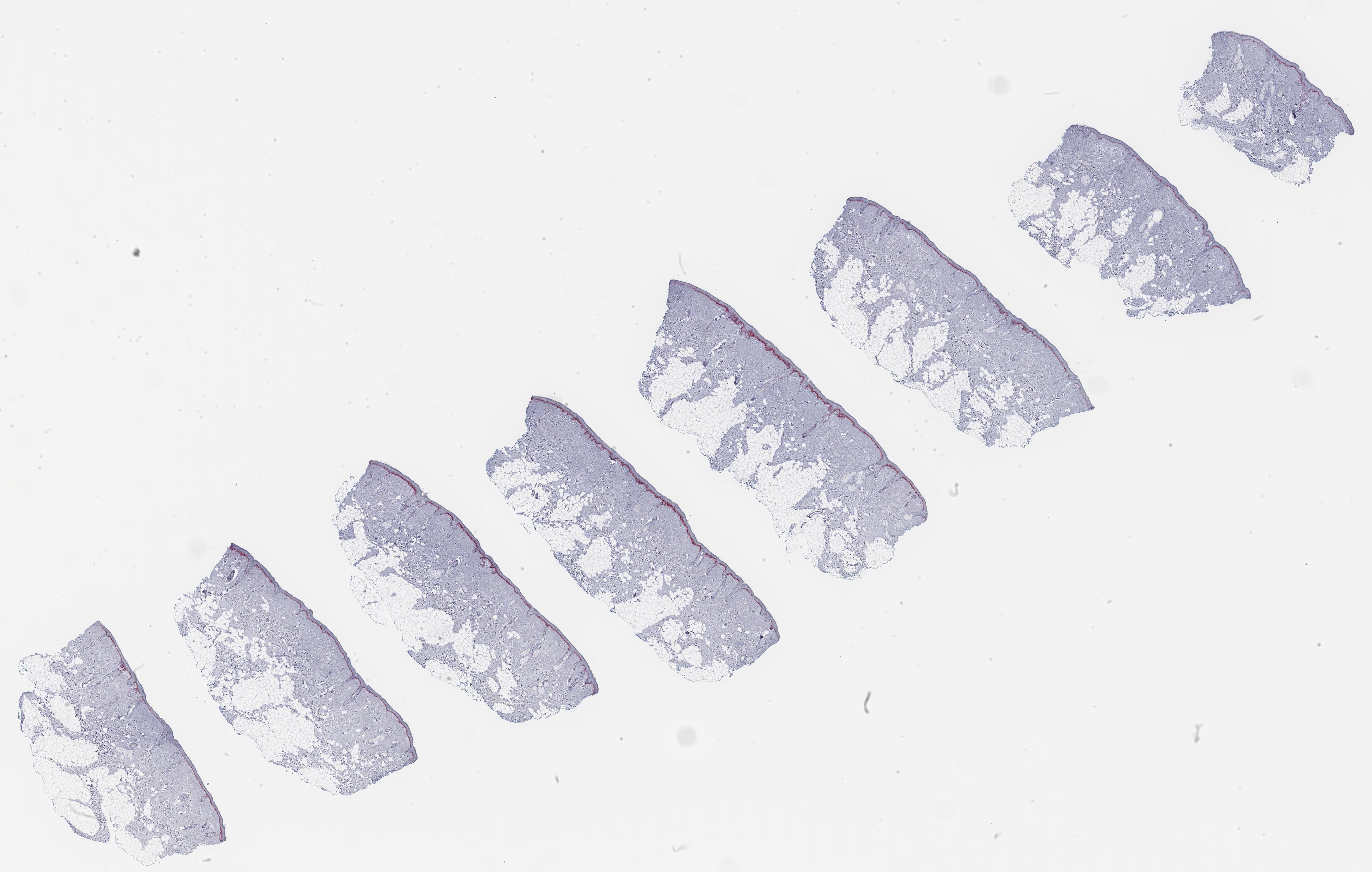

Supplement: Data S1. Illustrative low-resolution summary views of archival H&E-IHC whole slide image pairs, related to STAR Methods and Figure 1 — Details available in Tables S1 and S2. [file mmc2.zip › WSI-06_IHC.jpg]

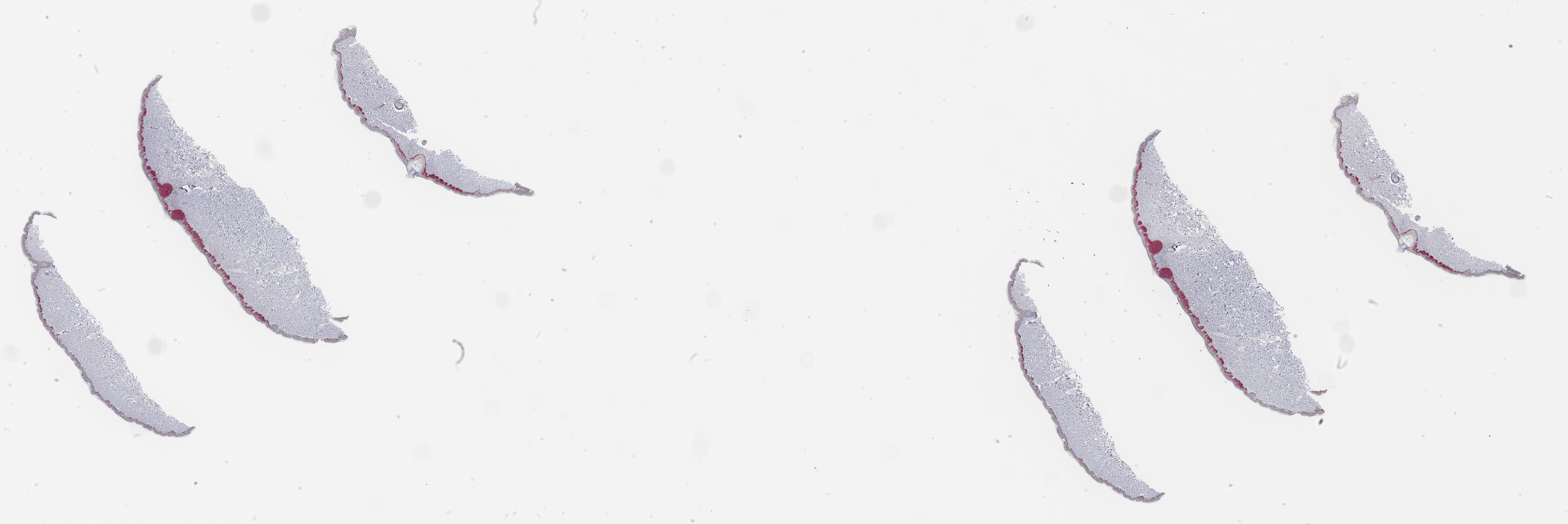

Supplement: Data S1. Illustrative low-resolution summary views of archival H&E-IHC whole slide image pairs, related to STAR Methods and Figure 1 — Details available in Tables S1 and S2. [file mmc2.zip › WSI-16_IHC.jpg]

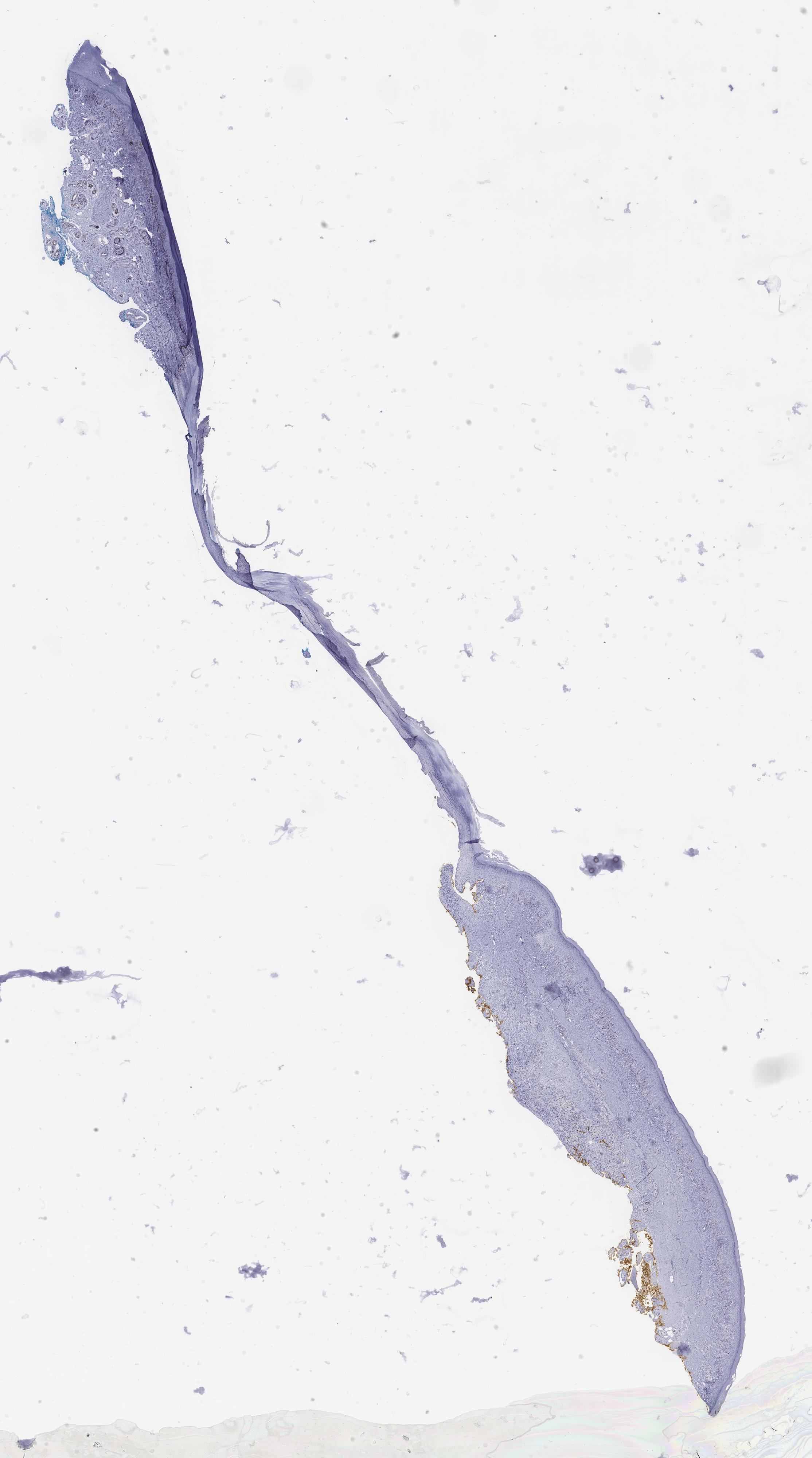

Supplement: Data S1. Illustrative low-resolution summary views of archival H&E-IHC whole slide image pairs, related to STAR Methods and Figure 1 — Details available in Tables S1 and S2. [file mmc2.zip › WSI-39_IHC.jpg]

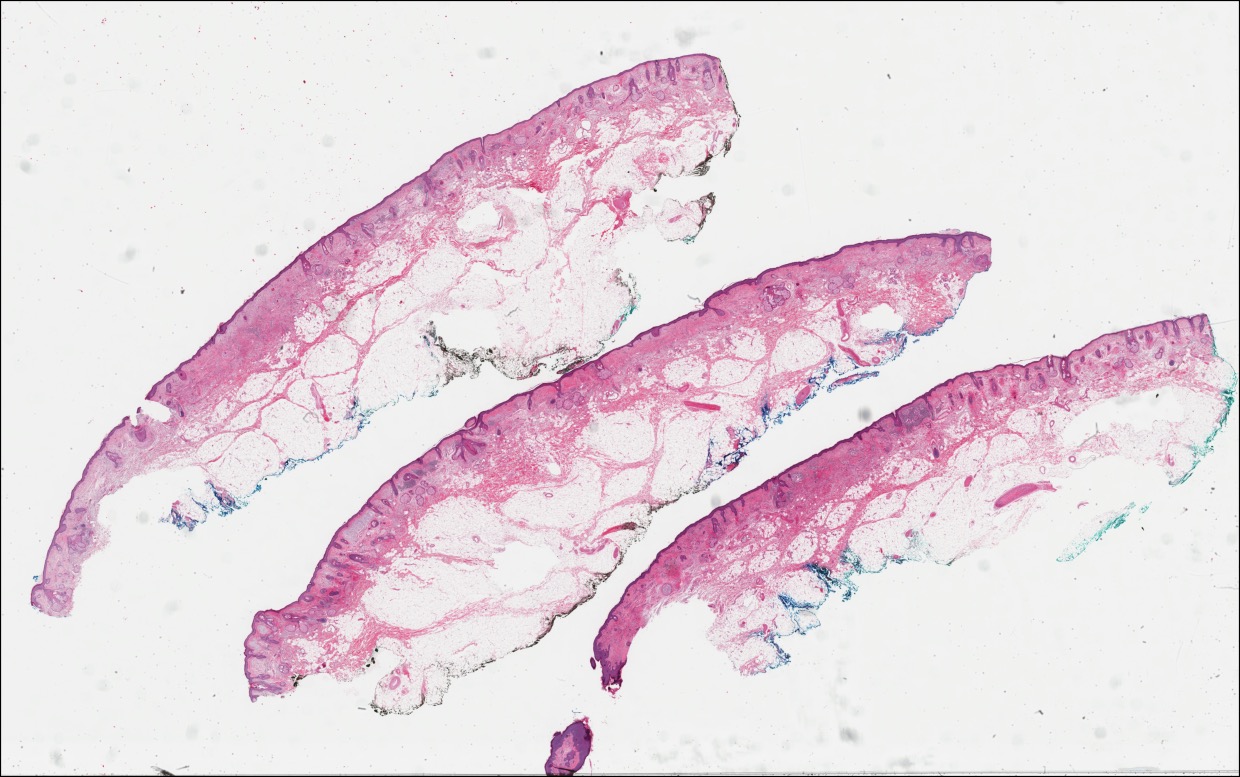

Supplement: Data S1. Illustrative low-resolution summary views of archival H&E-IHC whole slide image pairs, related to STAR Methods and Figure 1 — Details available in Tables S1 and S2. [file mmc2.zip › WSI-51_HE.jpg]

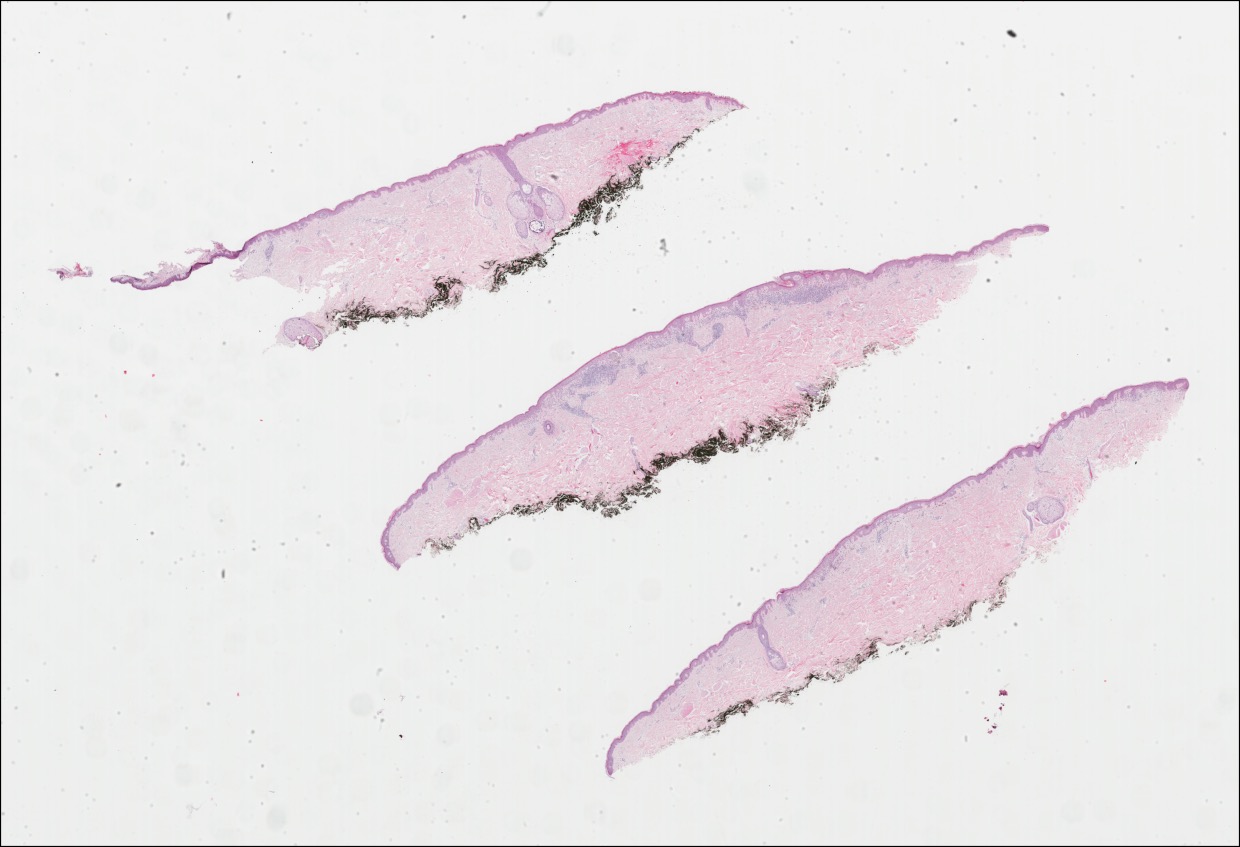

Supplement: Data S1. Illustrative low-resolution summary views of archival H&E-IHC whole slide image pairs, related to STAR Methods and Figure 1 — Details available in Tables S1 and S2. [file mmc2.zip › WSI-32_HE.jpg]

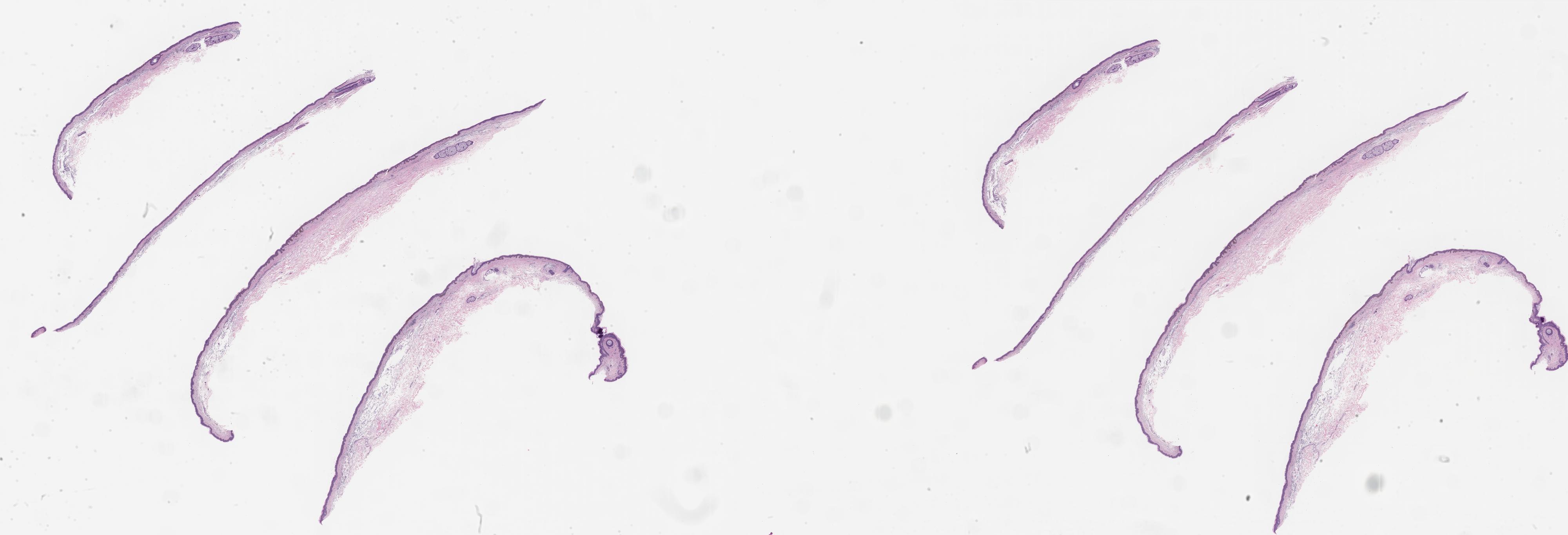

Supplement: Data S1. Illustrative low-resolution summary views of archival H&E-IHC whole slide image pairs, related to STAR Methods and Figure 1 — Details available in Tables S1 and S2. [file mmc2.zip › WSI-43_HE.jpg]

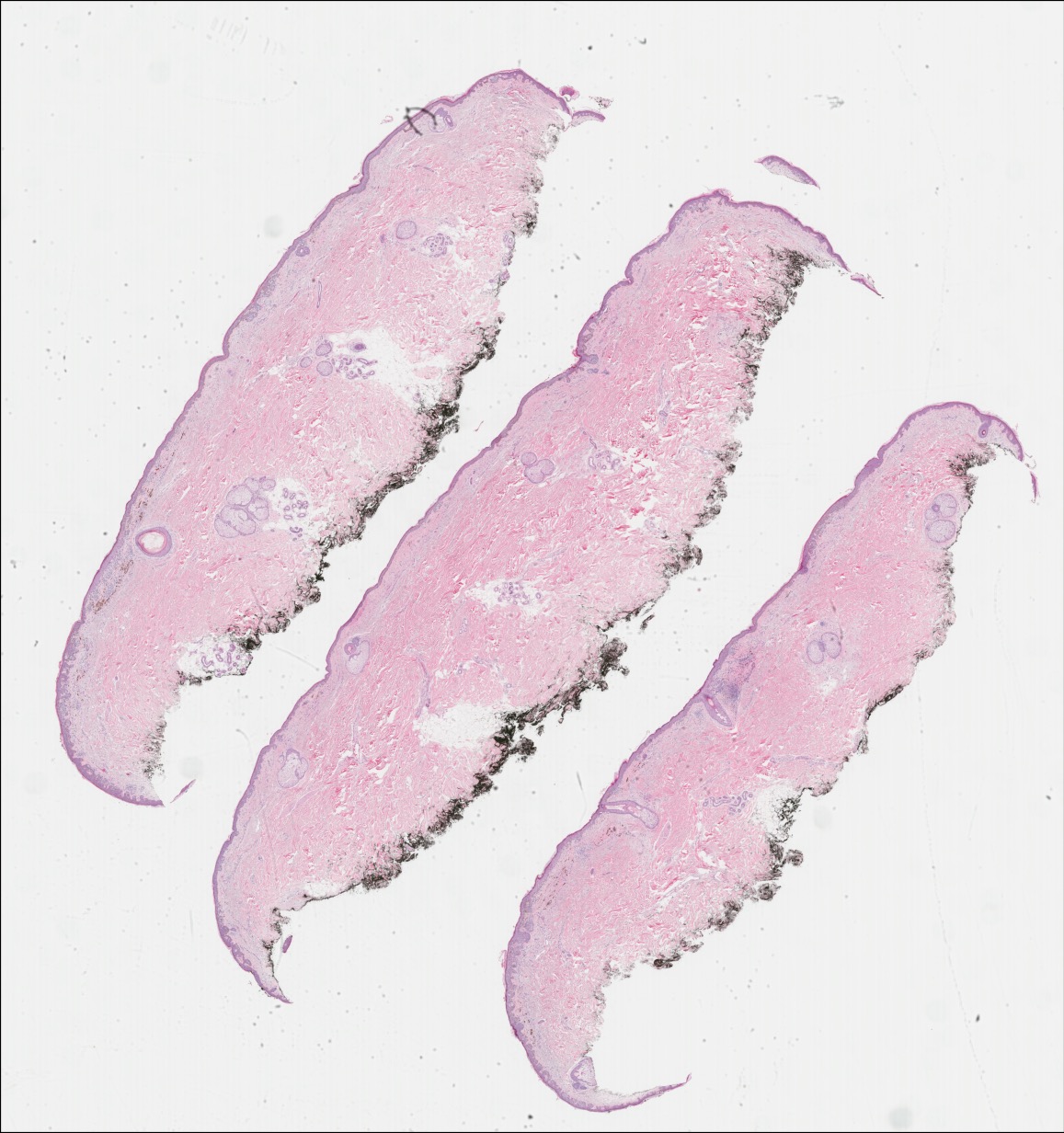

Supplement: Data S1. Illustrative low-resolution summary views of archival H&E-IHC whole slide image pairs, related to STAR Methods and Figure 1 — Details available in Tables S1 and S2. [file mmc2.zip › WSI-20_HE.jpg]

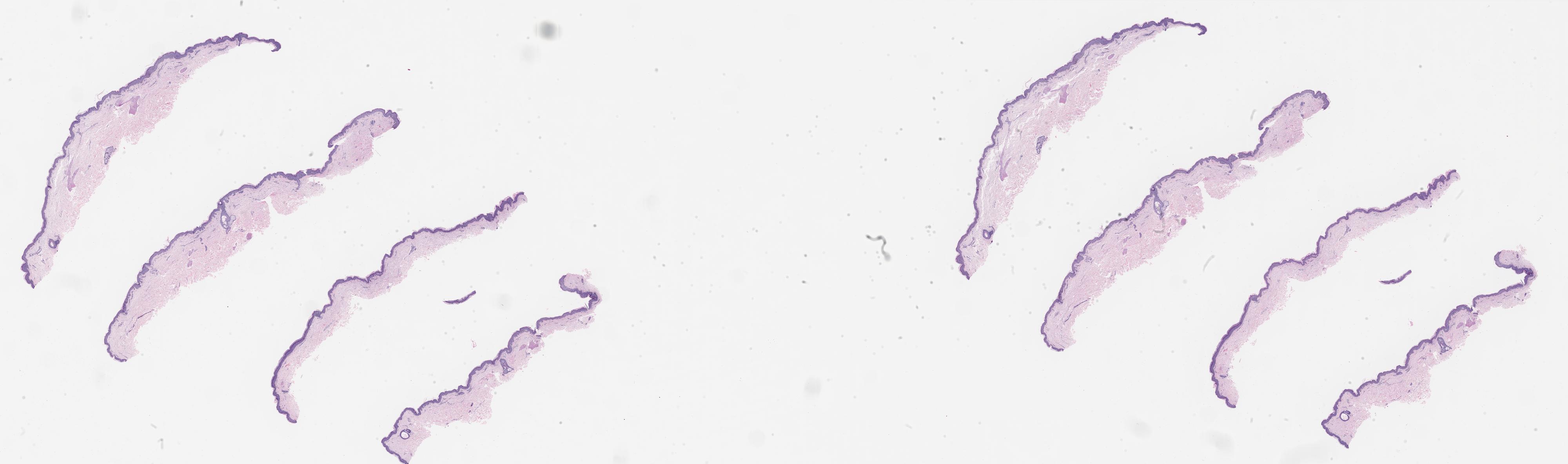

Supplement: Data S1. Illustrative low-resolution summary views of archival H&E-IHC whole slide image pairs, related to STAR Methods and Figure 1 — Details available in Tables S1 and S2. [file mmc2.zip › WSI-08_HE.jpg]

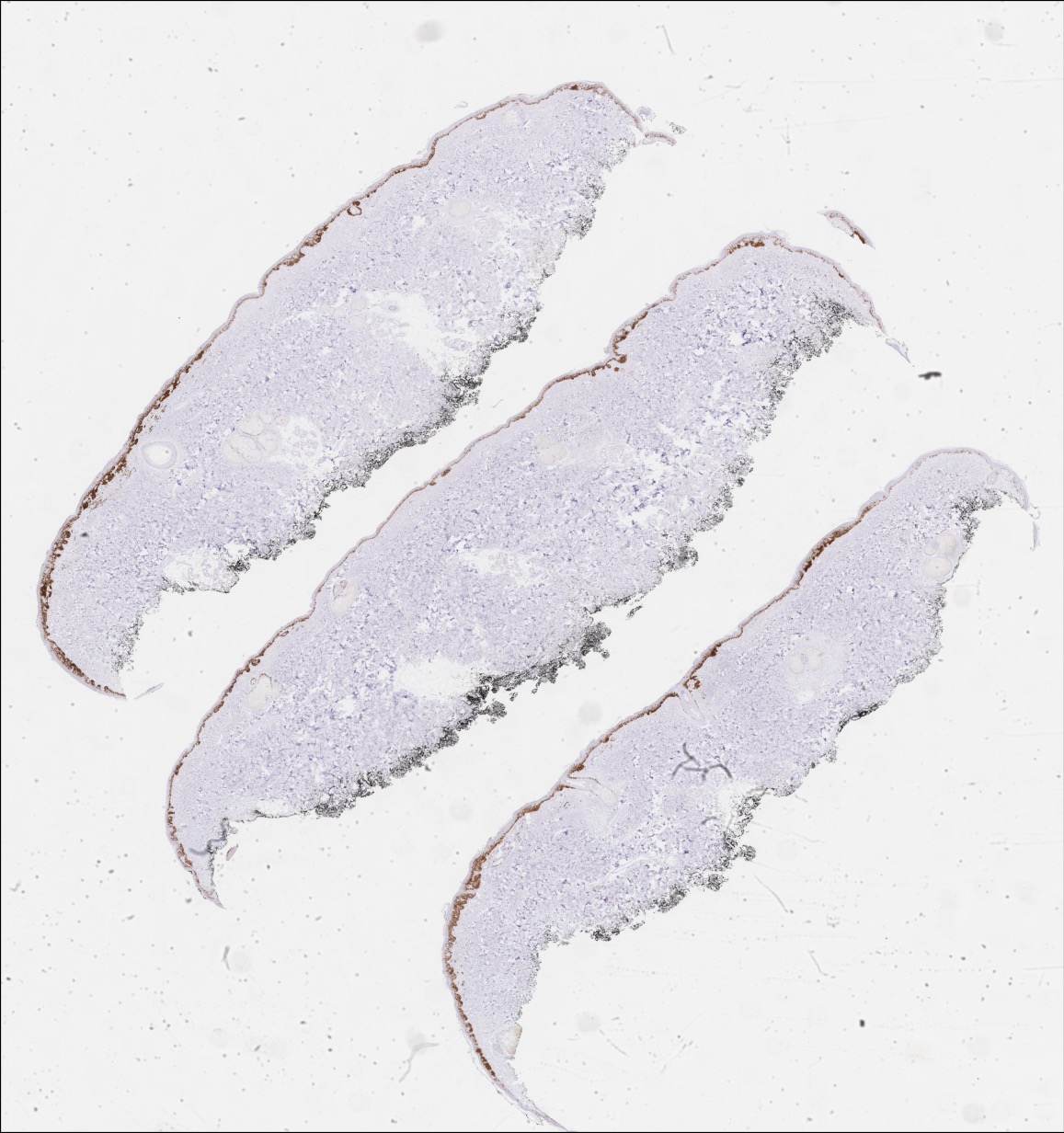

Supplement: Data S1. Illustrative low-resolution summary views of archival H&E-IHC whole slide image pairs, related to STAR Methods and Figure 1 — Details available in Tables S1 and S2. [file mmc2.zip › WSI-20_IHC.jpg]

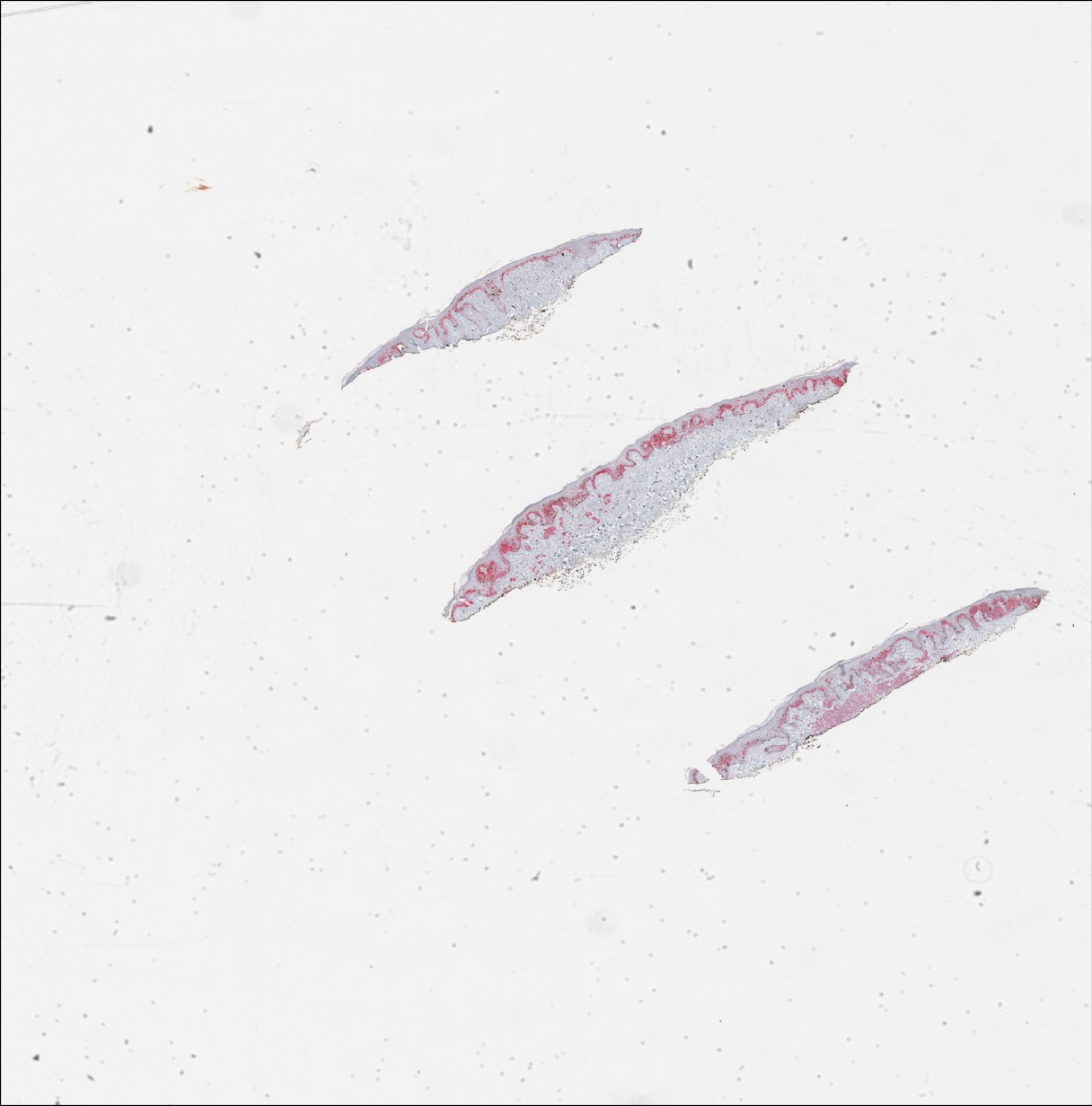

Supplement: Data S1. Illustrative low-resolution summary views of archival H&E-IHC whole slide image pairs, related to STAR Methods and Figure 1 — Details available in Tables S1 and S2. [file mmc2.zip › WSI-30_IHC.jpg]

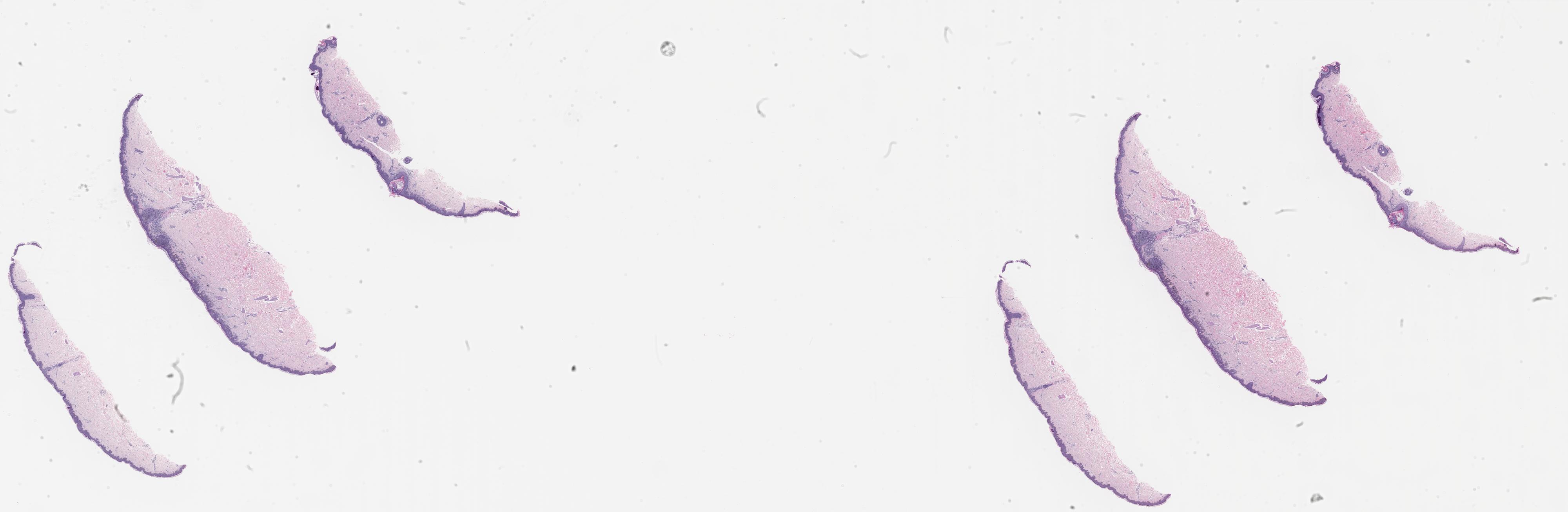

Supplement: Data S1. Illustrative low-resolution summary views of archival H&E-IHC whole slide image pairs, related to STAR Methods and Figure 1 — Details available in Tables S1 and S2. [file mmc2.zip › WSI-16_HE.jpg]

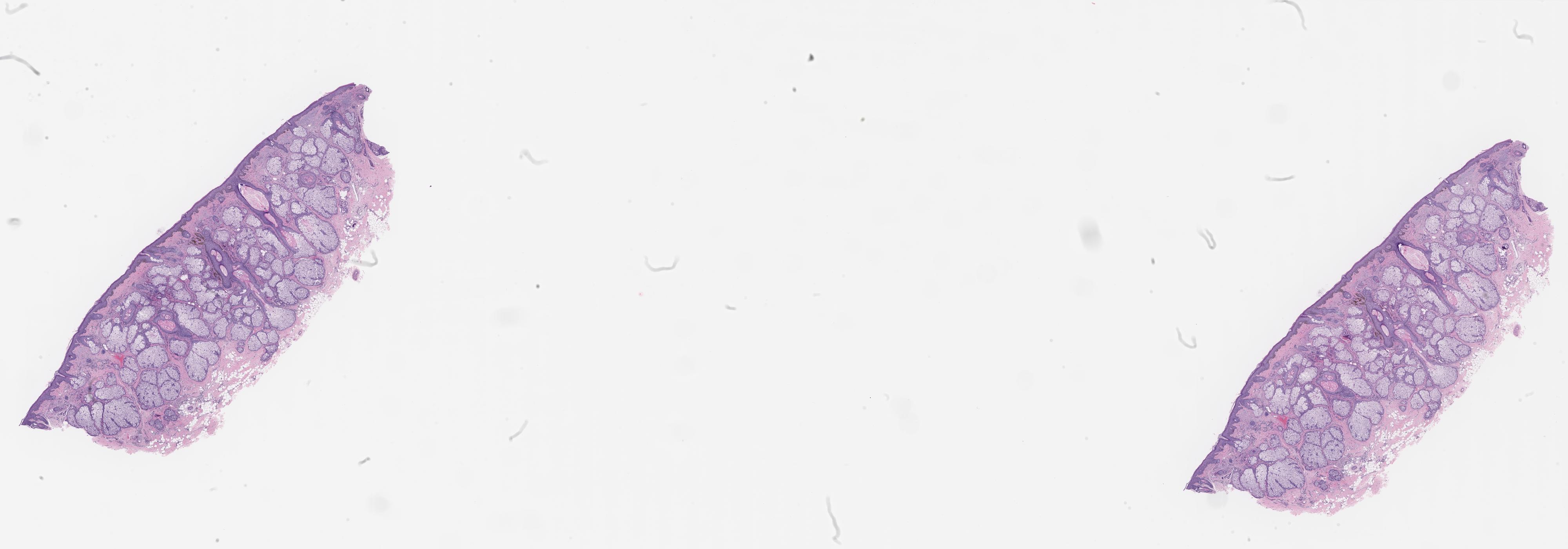

Supplement: Data S1. Illustrative low-resolution summary views of archival H&E-IHC whole slide image pairs, related to STAR Methods and Figure 1 — Details available in Tables S1 and S2. [file mmc2.zip › WSI-04_HE.jpg]

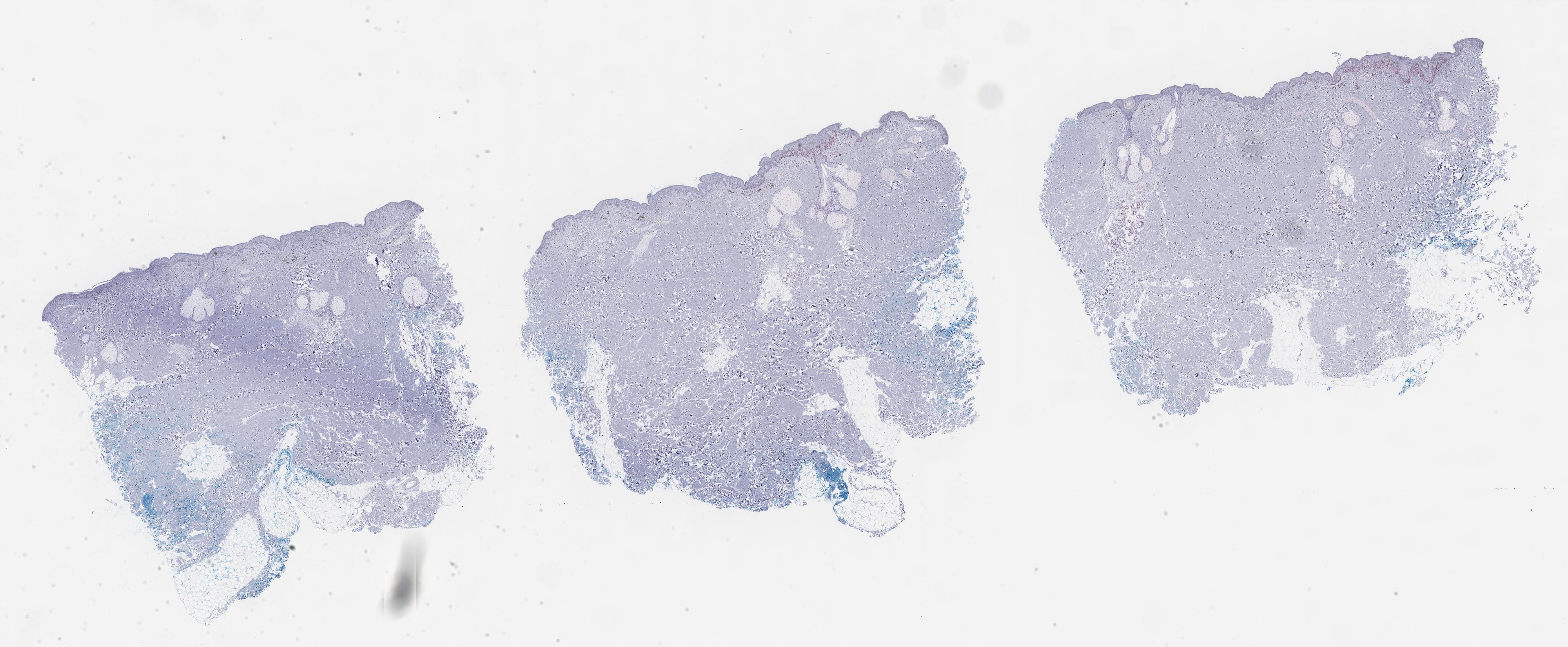

Supplement: Data S1. Illustrative low-resolution summary views of archival H&E-IHC whole slide image pairs, related to STAR Methods and Figure 1 — Details available in Tables S1 and S2. [file mmc2.zip › WSI-42_IHC.jpg]
