# Supplementary figures and images for: Deep learning finds convergent melanocytic morphology despite noisy archival slides (part 1 of 2)
Source: Cell Rep Methods. 2025 Oct 20;5(10):101201. doi: 10.1016/j.crmeth.2025.101201 (PMC12570353; doi:10.1016/j.crmeth.2025.101201)

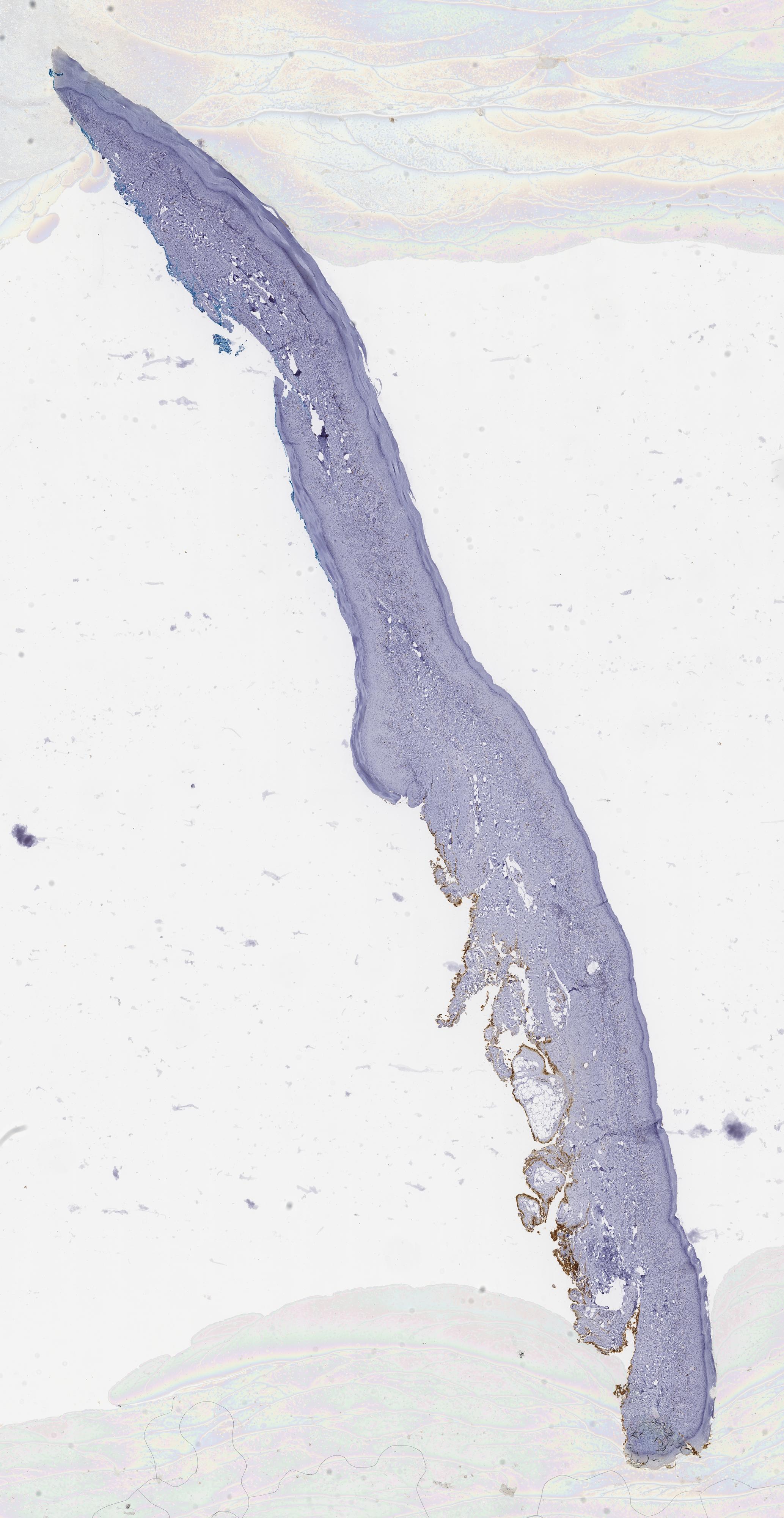

Supplement: Data S1. Illustrative low-resolution summary views of archival H&E-IHC whole slide image pairs, related to STAR Methods and Figure 1 — Details available in Tables S1 and S2. [file mmc2.zip › WSI-35_IHC.jpg]

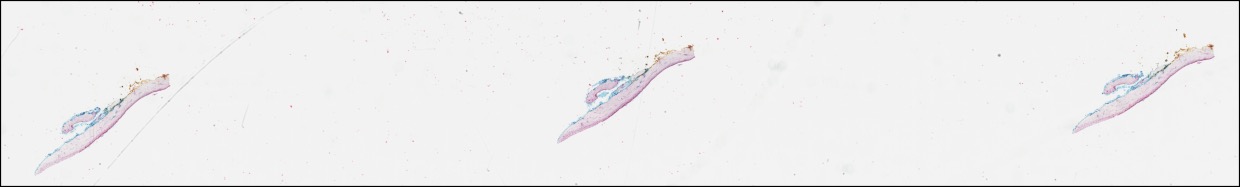

Supplement: Data S1. Illustrative low-resolution summary views of archival H&E-IHC whole slide image pairs, related to STAR Methods and Figure 1 — Details available in Tables S1 and S2. [file mmc2.zip › WSI-25_HE.jpg]

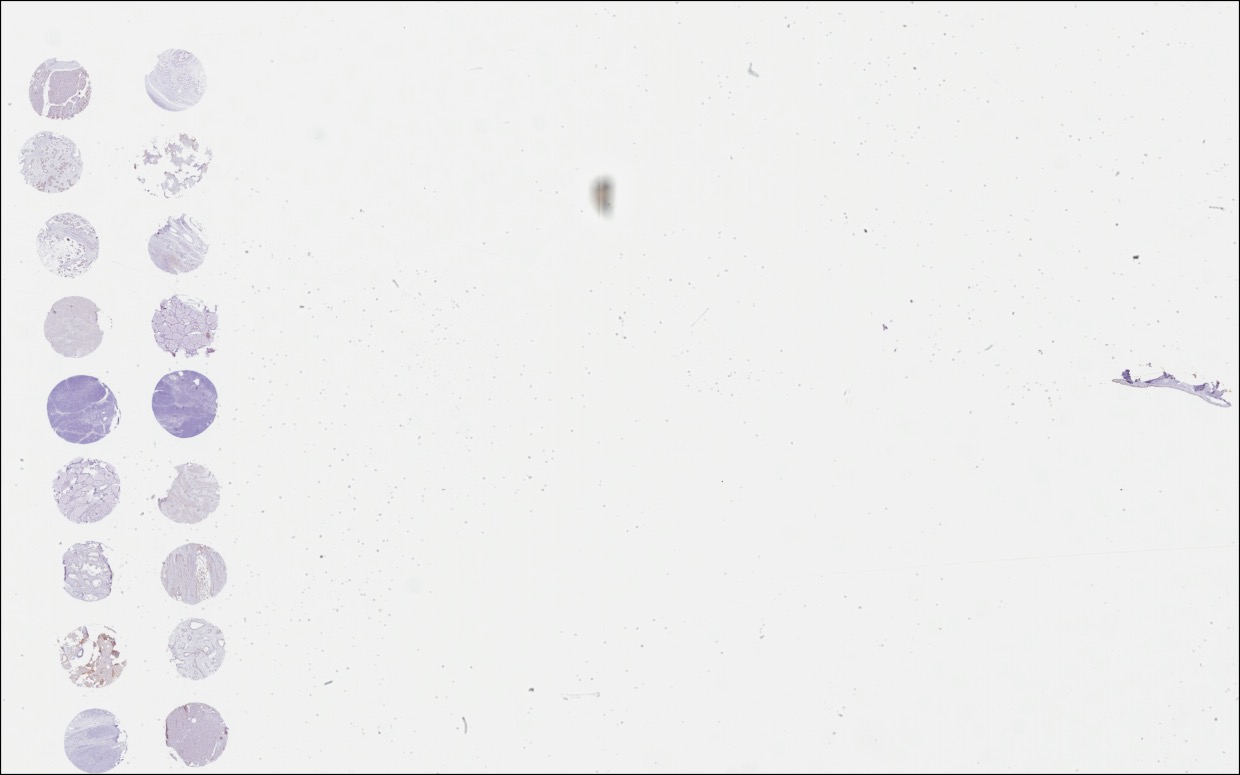

Supplement: Data S1. Illustrative low-resolution summary views of archival H&E-IHC whole slide image pairs, related to STAR Methods and Figure 1 — Details available in Tables S1 and S2. [file mmc2.zip › WSI-25_IHC.jpg]

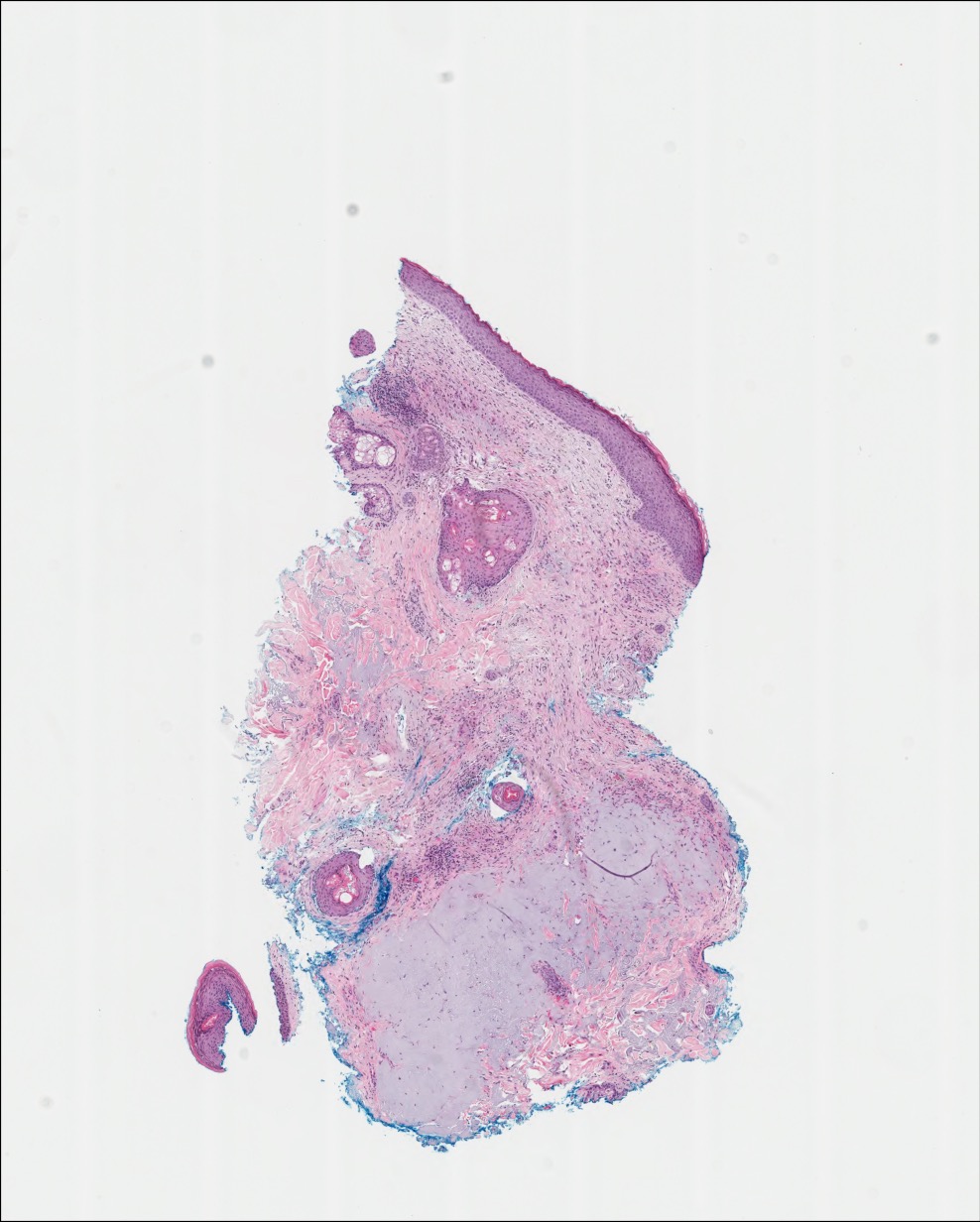

Supplement: Data S1. Illustrative low-resolution summary views of archival H&E-IHC whole slide image pairs, related to STAR Methods and Figure 1 — Details available in Tables S1 and S2. [file mmc2.zip › WSI-58_HE.jpg]

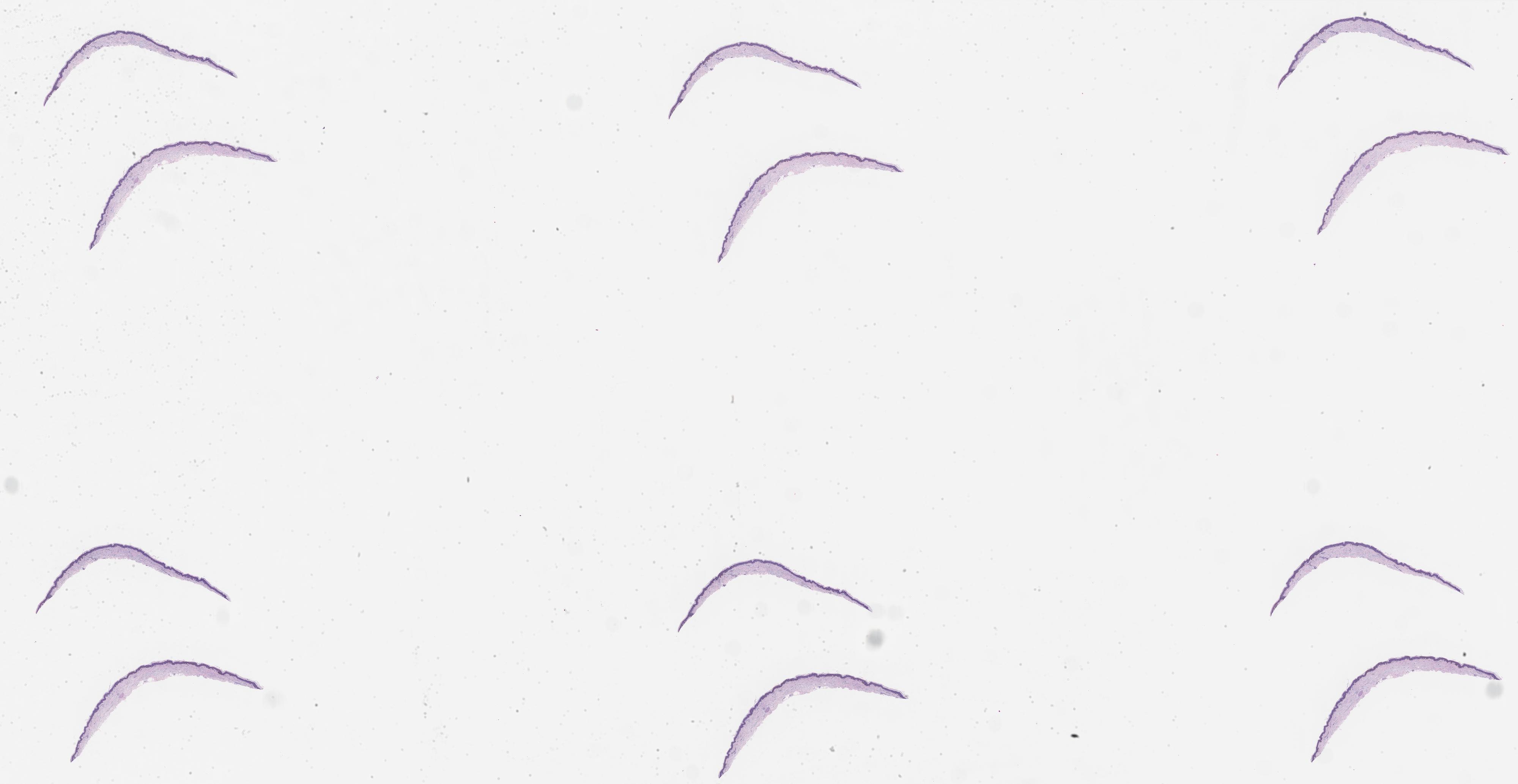

Supplement: Data S1. Illustrative low-resolution summary views of archival H&E-IHC whole slide image pairs, related to STAR Methods and Figure 1 — Details available in Tables S1 and S2. [file mmc2.zip › WSI-46_HE.jpg]

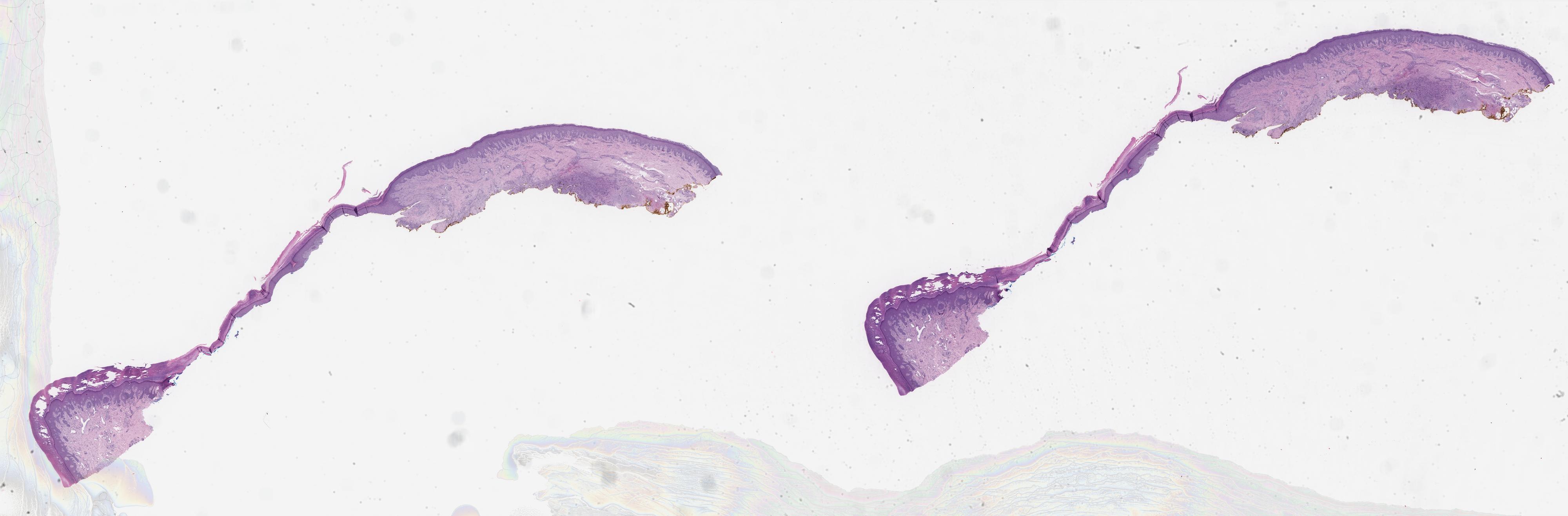

Supplement: Data S1. Illustrative low-resolution summary views of archival H&E-IHC whole slide image pairs, related to STAR Methods and Figure 1 — Details available in Tables S1 and S2. [file mmc2.zip › WSI-37_HE.jpg]

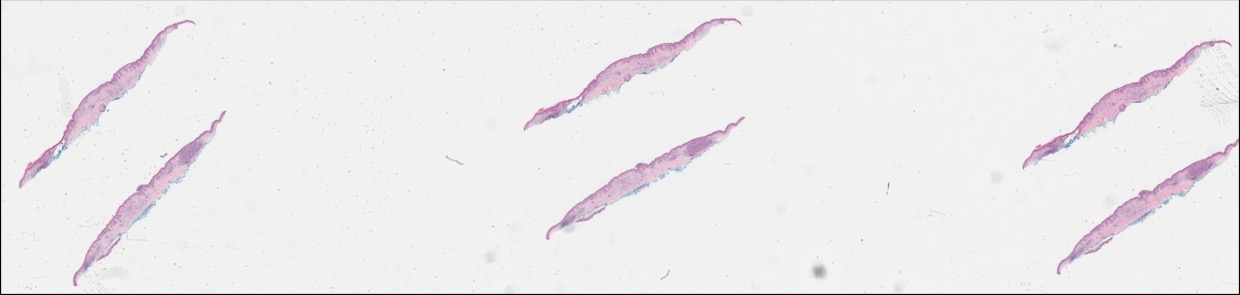

Supplement: Data S1. Illustrative low-resolution summary views of archival H&E-IHC whole slide image pairs, related to STAR Methods and Figure 1 — Details available in Tables S1 and S2. [file mmc2.zip › WSI-29_HE.jpg]

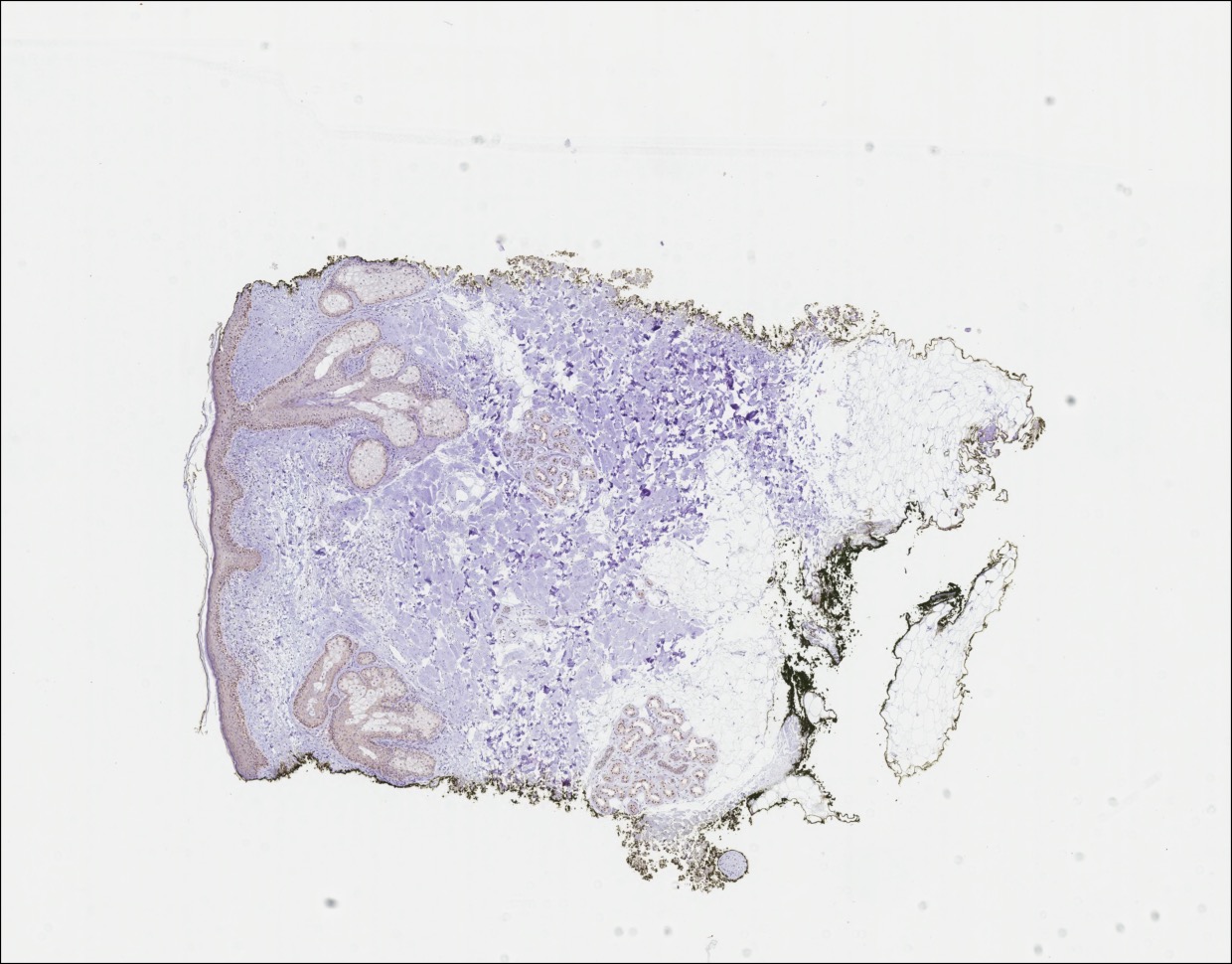

Supplement: Data S1. Illustrative low-resolution summary views of archival H&E-IHC whole slide image pairs, related to STAR Methods and Figure 1 — Details available in Tables S1 and S2. [file mmc2.zip › WSI-57_IHC.jpg]

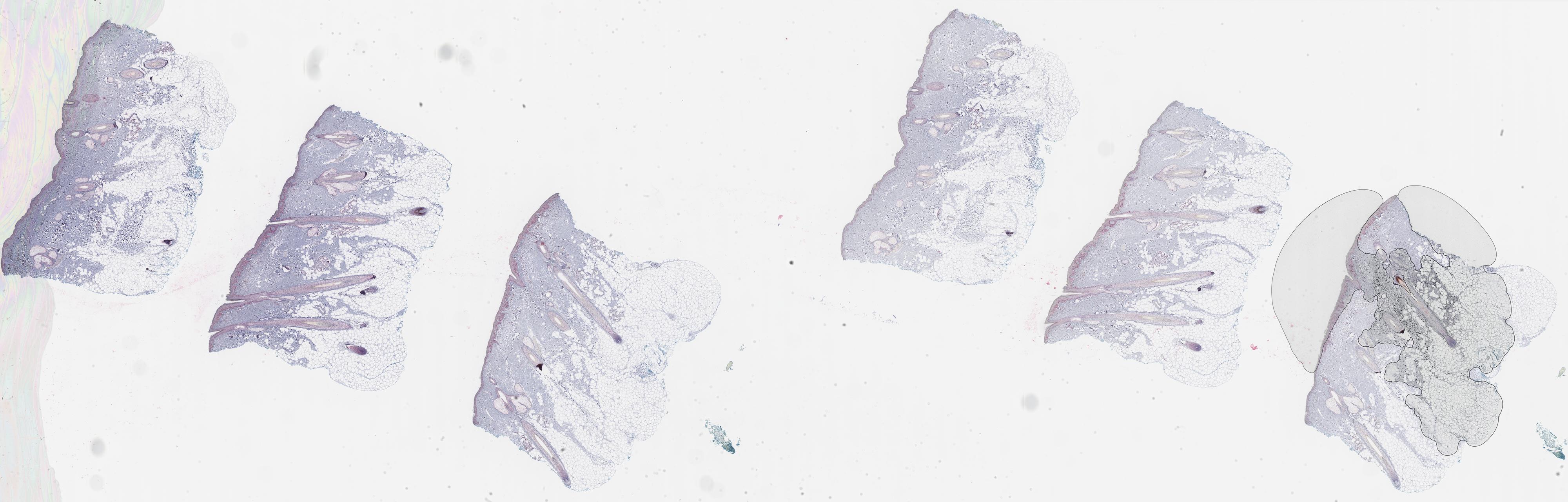

Supplement: Data S1. Illustrative low-resolution summary views of archival H&E-IHC whole slide image pairs, related to STAR Methods and Figure 1 — Details available in Tables S1 and S2. [file mmc2.zip › WSI-47_IHC.jpg]

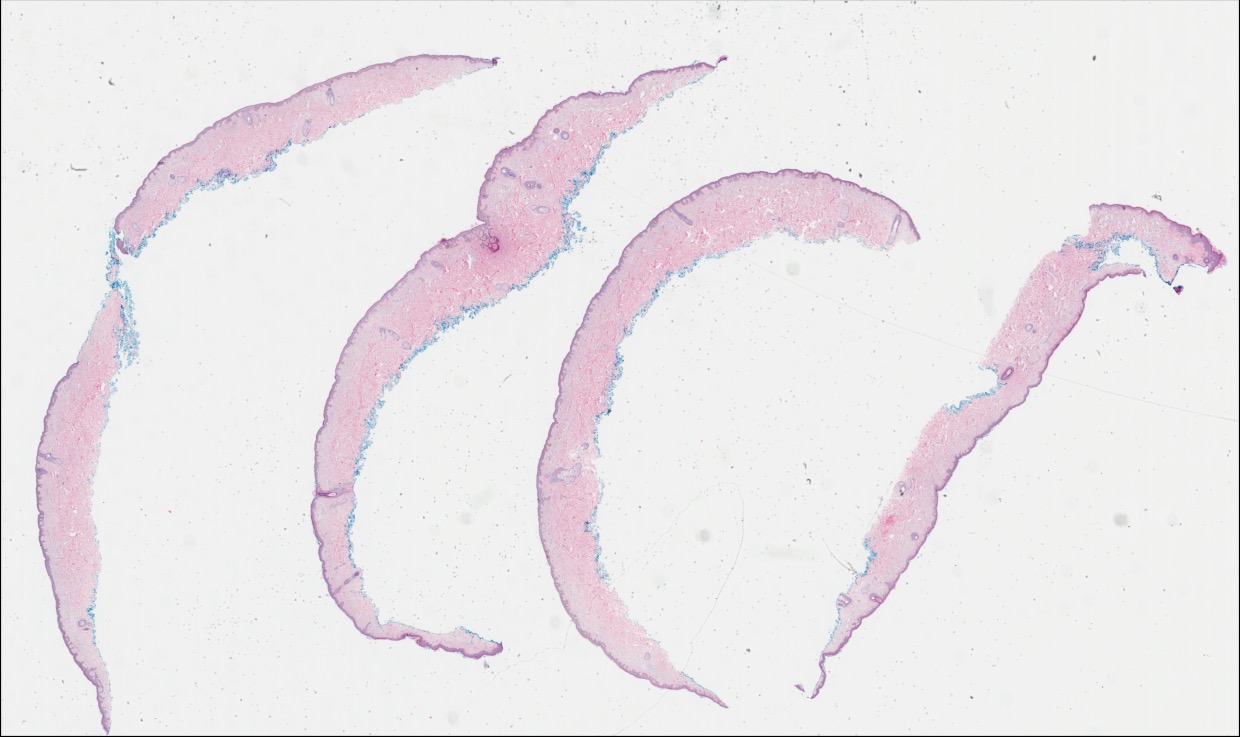

Supplement: Data S1. Illustrative low-resolution summary views of archival H&E-IHC whole slide image pairs, related to STAR Methods and Figure 1 — Details available in Tables S1 and S2. [file mmc2.zip › WSI-54_HE.jpg]

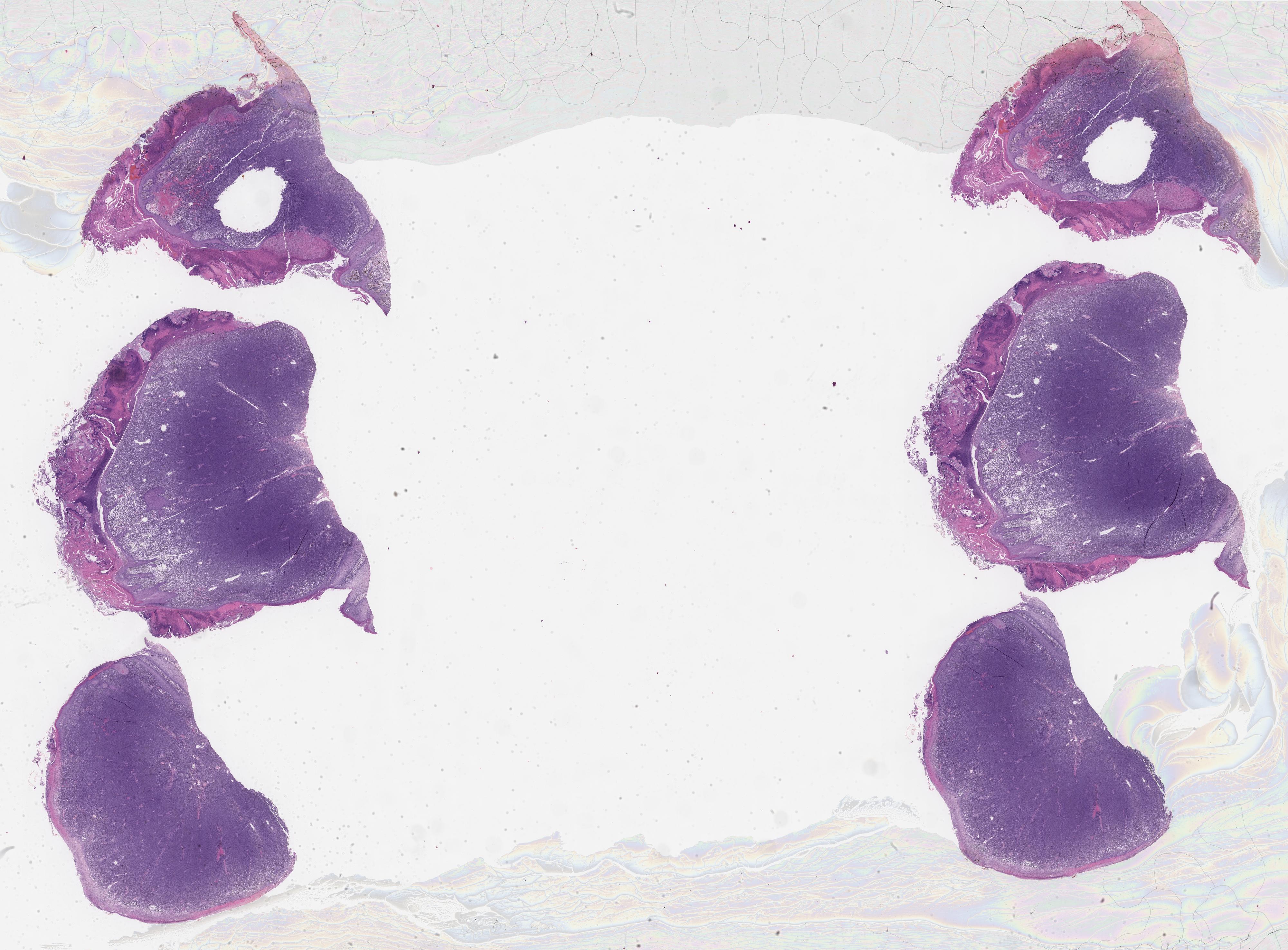

Supplement: Data S1. Illustrative low-resolution summary views of archival H&E-IHC whole slide image pairs, related to STAR Methods and Figure 1 — Details available in Tables S1 and S2. [file mmc2.zip › WSI-01_HE.jpg]

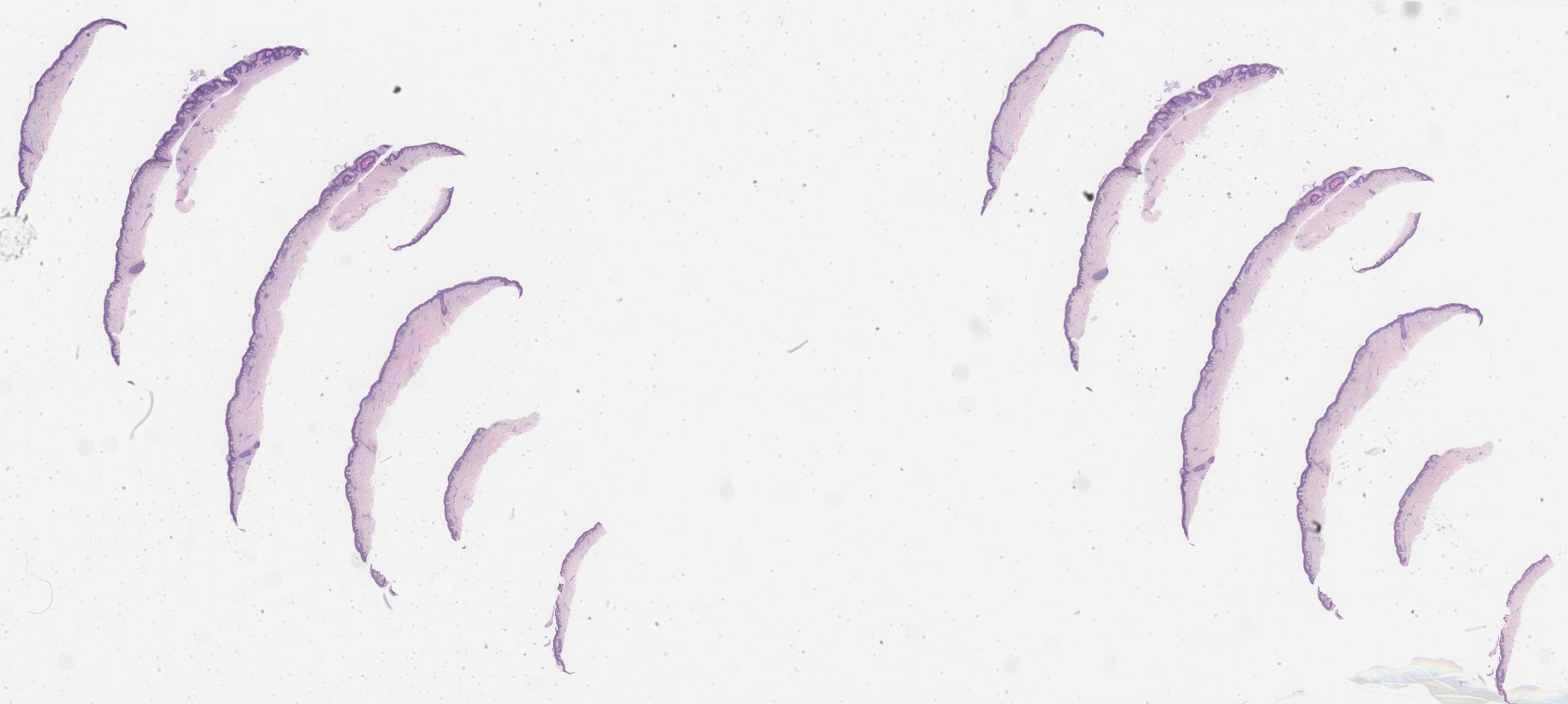

Supplement: Data S1. Illustrative low-resolution summary views of archival H&E-IHC whole slide image pairs, related to STAR Methods and Figure 1 — Details available in Tables S1 and S2. [file mmc2.zip › WSI-13_HE.jpg]

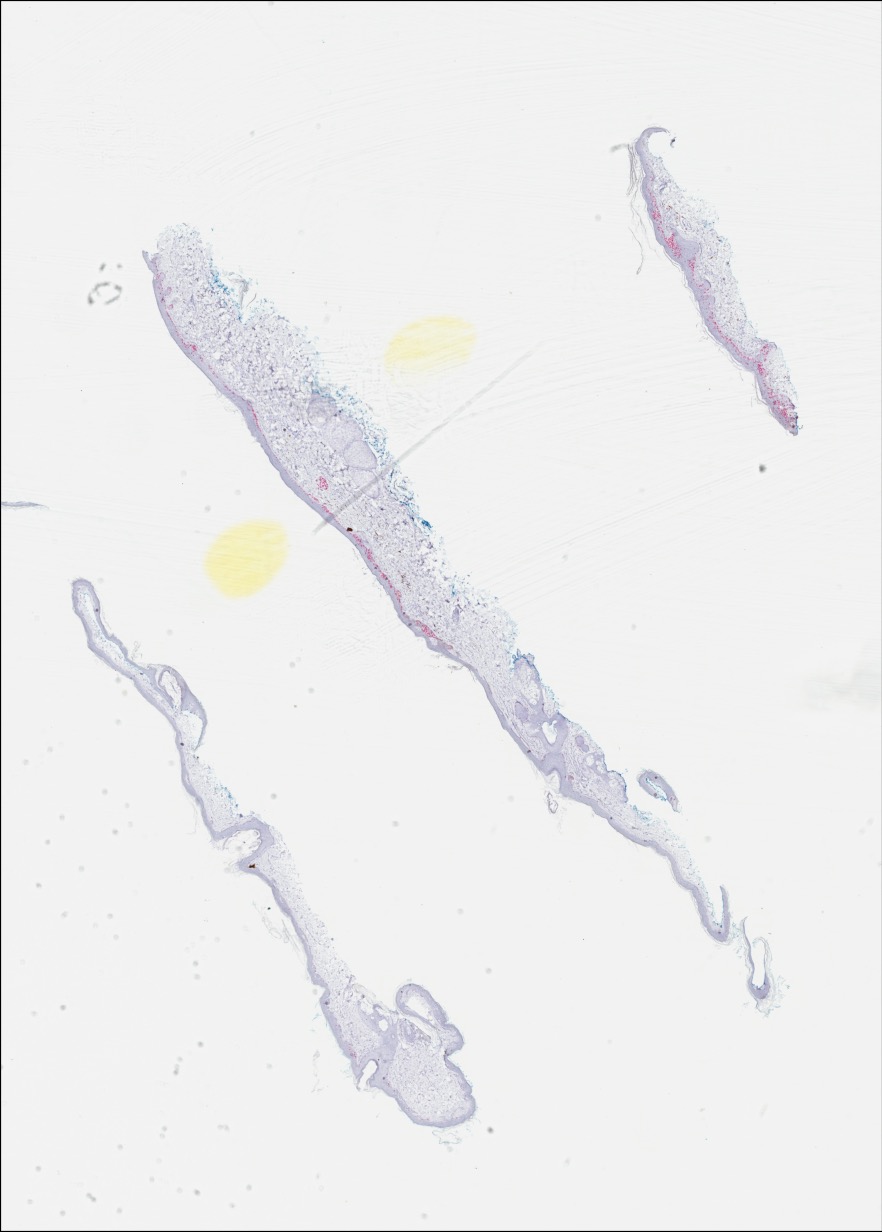

Supplement: Data S1. Illustrative low-resolution summary views of archival H&E-IHC whole slide image pairs, related to STAR Methods and Figure 1 — Details available in Tables S1 and S2. [file mmc2.zip › WSI-61_IHC.jpg]

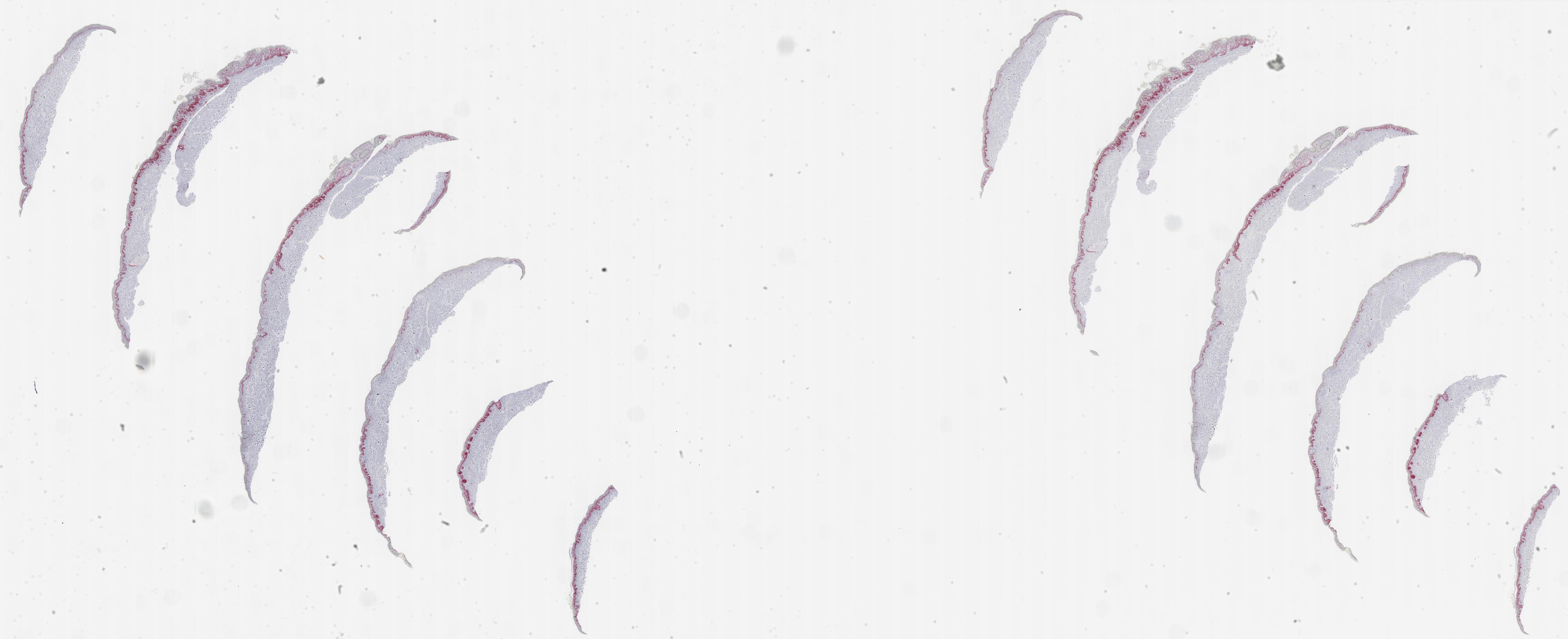

Supplement: Data S1. Illustrative low-resolution summary views of archival H&E-IHC whole slide image pairs, related to STAR Methods and Figure 1 — Details available in Tables S1 and S2. [file mmc2.zip › WSI-13_IHC.jpg]

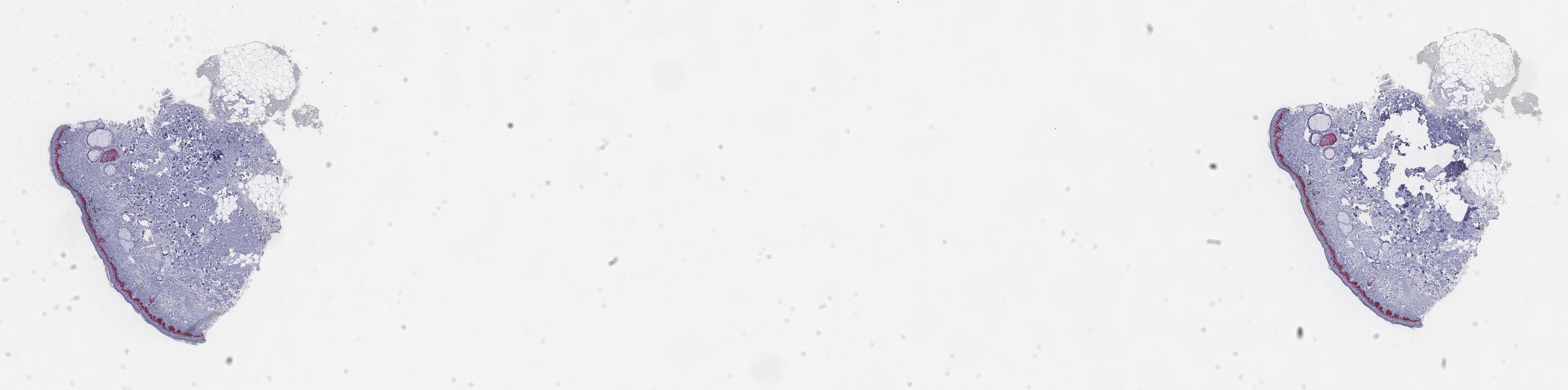

Supplement: Data S1. Illustrative low-resolution summary views of archival H&E-IHC whole slide image pairs, related to STAR Methods and Figure 1 — Details available in Tables S1 and S2. [file mmc2.zip › WSI-03_IHC.jpg]

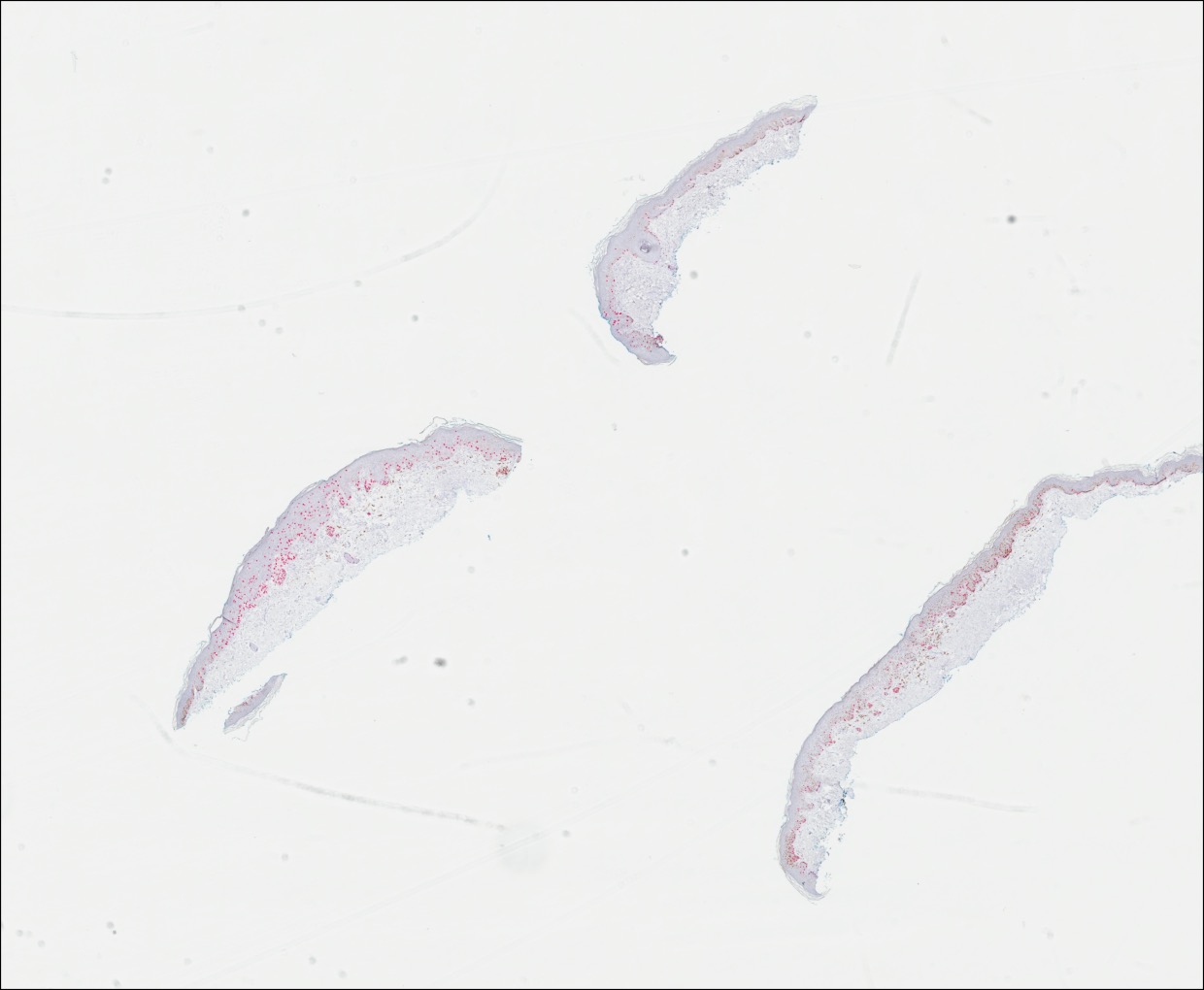

Supplement: Data S1. Illustrative low-resolution summary views of archival H&E-IHC whole slide image pairs, related to STAR Methods and Figure 1 — Details available in Tables S1 and S2. [file mmc2.zip › WSI-60_IHC.jpg]

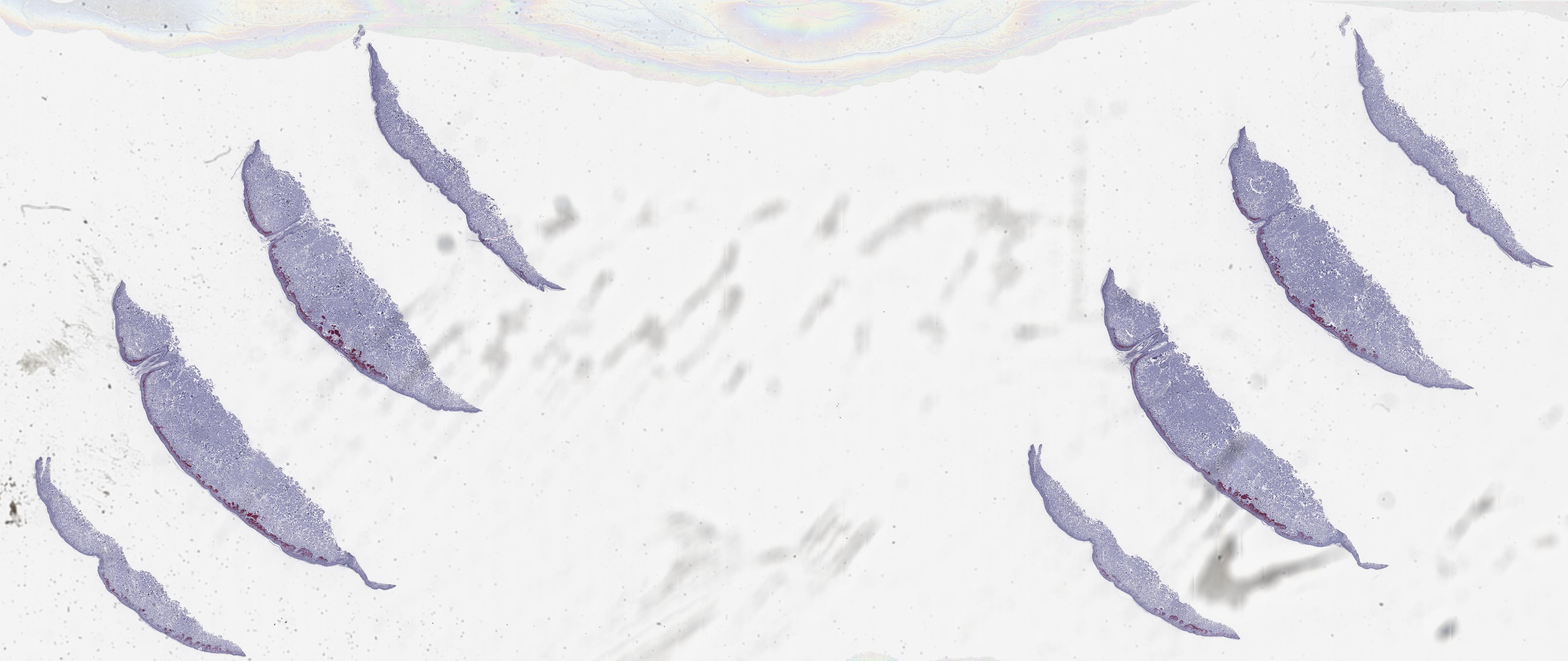

Supplement: Data S1. Illustrative low-resolution summary views of archival H&E-IHC whole slide image pairs, related to STAR Methods and Figure 1 — Details available in Tables S1 and S2. [file mmc2.zip › WSI-02_IHC.jpg]

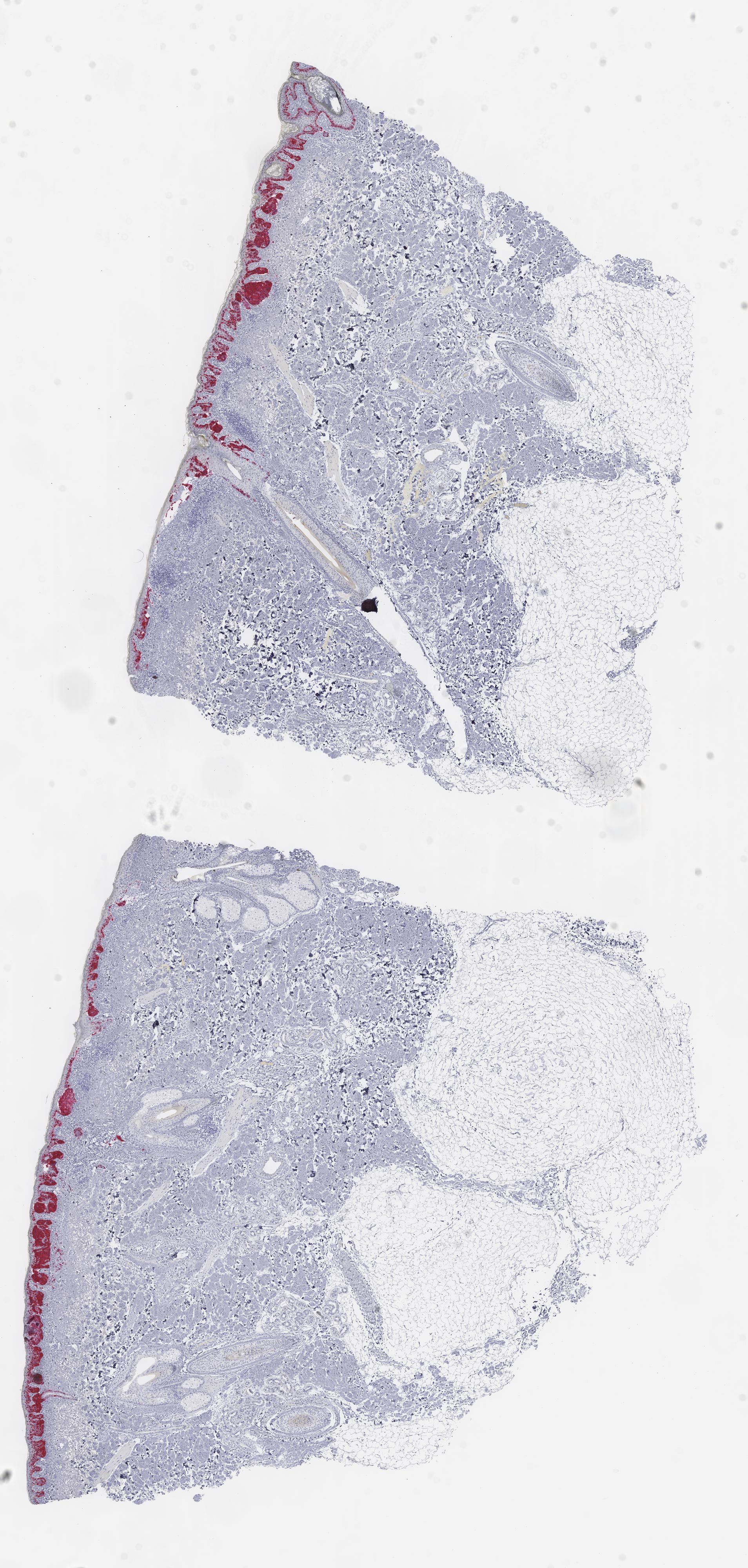

Supplement: Data S1. Illustrative low-resolution summary views of archival H&E-IHC whole slide image pairs, related to STAR Methods and Figure 1 — Details available in Tables S1 and S2. [file mmc2.zip › WSI-12_IHC.jpg]

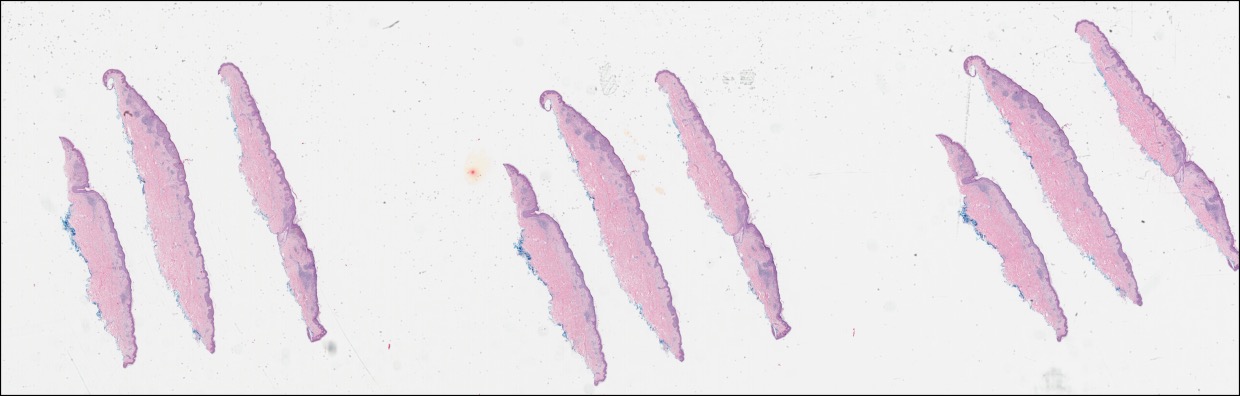

Supplement: Data S1. Illustrative low-resolution summary views of archival H&E-IHC whole slide image pairs, related to STAR Methods and Figure 1 — Details available in Tables S1 and S2. [file mmc2.zip › WSI-56_HE.jpg]

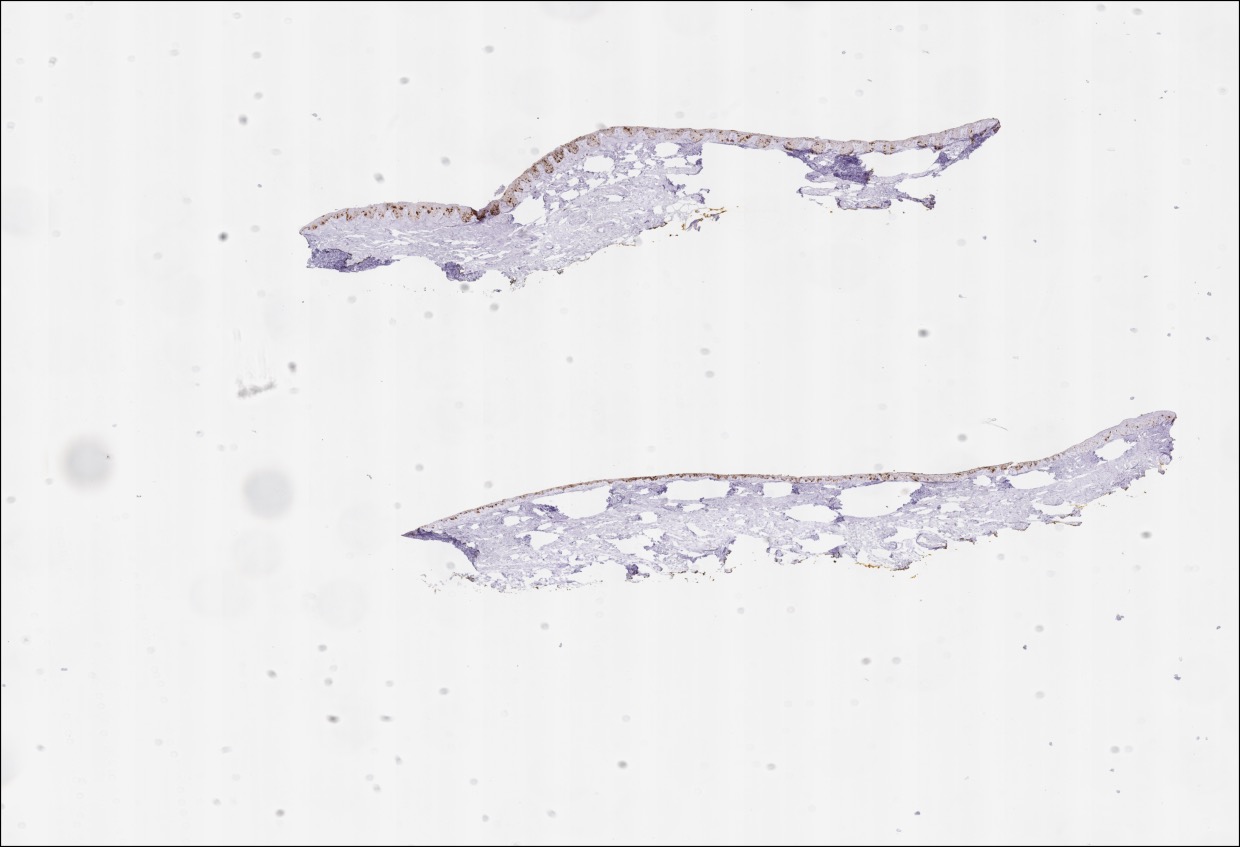

Supplement: Data S1. Illustrative low-resolution summary views of archival H&E-IHC whole slide image pairs, related to STAR Methods and Figure 1 — Details available in Tables S1 and S2. [file mmc2.zip › WSI-24_IHC.jpg]

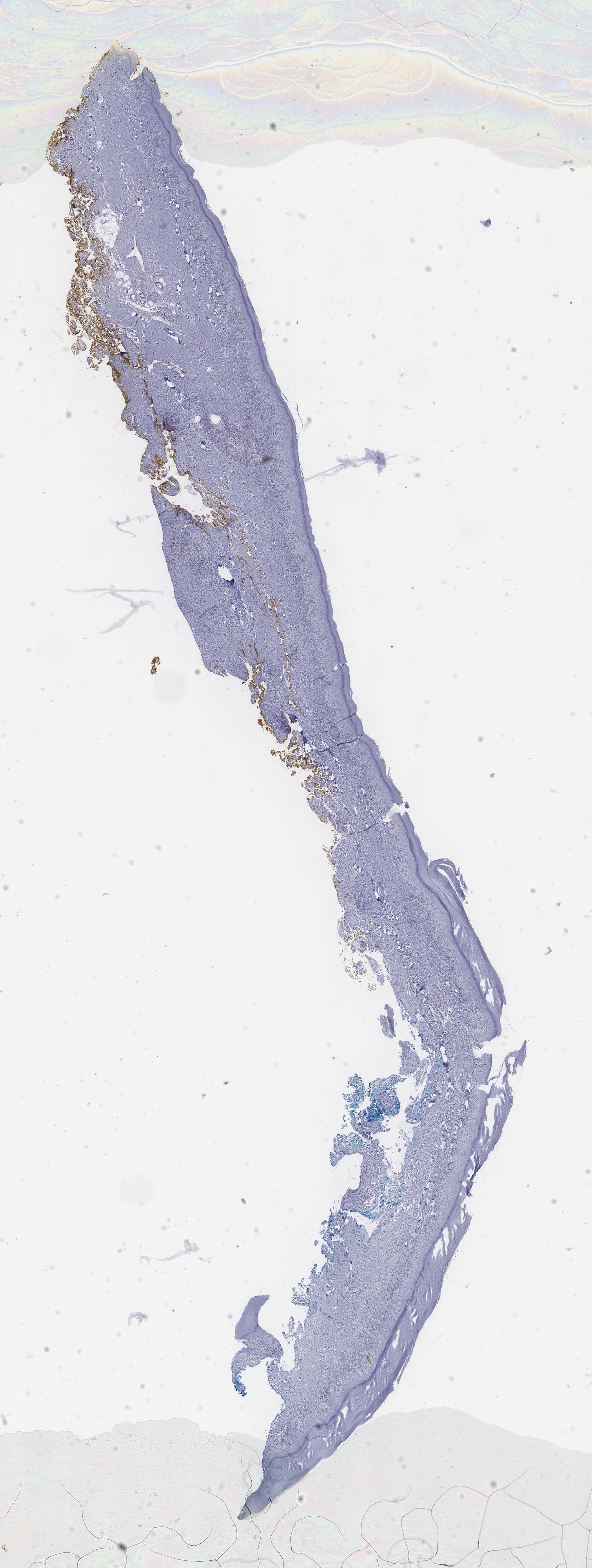

Supplement: Data S1. Illustrative low-resolution summary views of archival H&E-IHC whole slide image pairs, related to STAR Methods and Figure 1 — Details available in Tables S1 and S2. [file mmc2.zip › WSI-34_IHC.jpg]

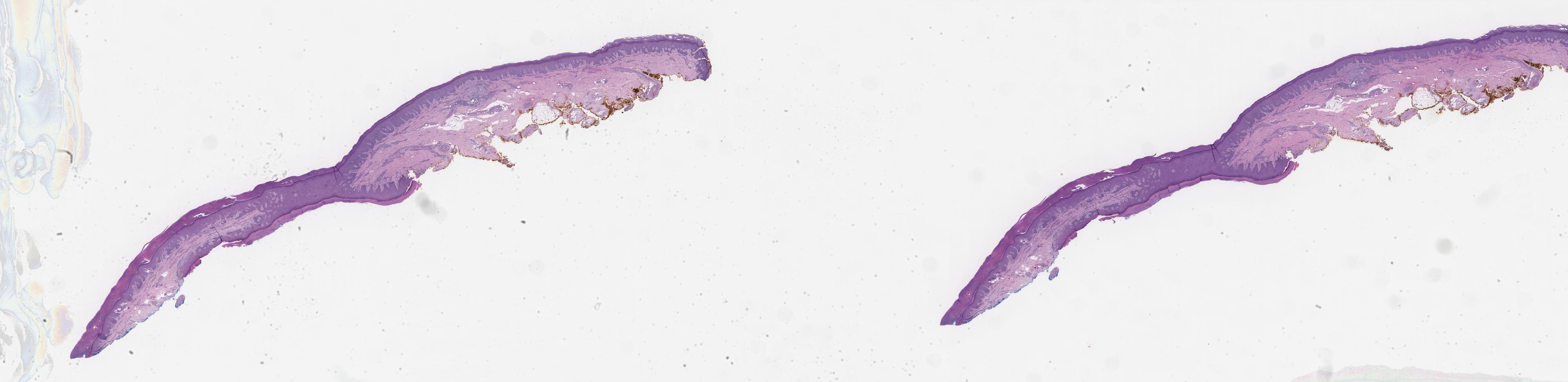

Supplement: Data S1. Illustrative low-resolution summary views of archival H&E-IHC whole slide image pairs, related to STAR Methods and Figure 1 — Details available in Tables S1 and S2. [file mmc2.zip › WSI-35_HE.jpg]

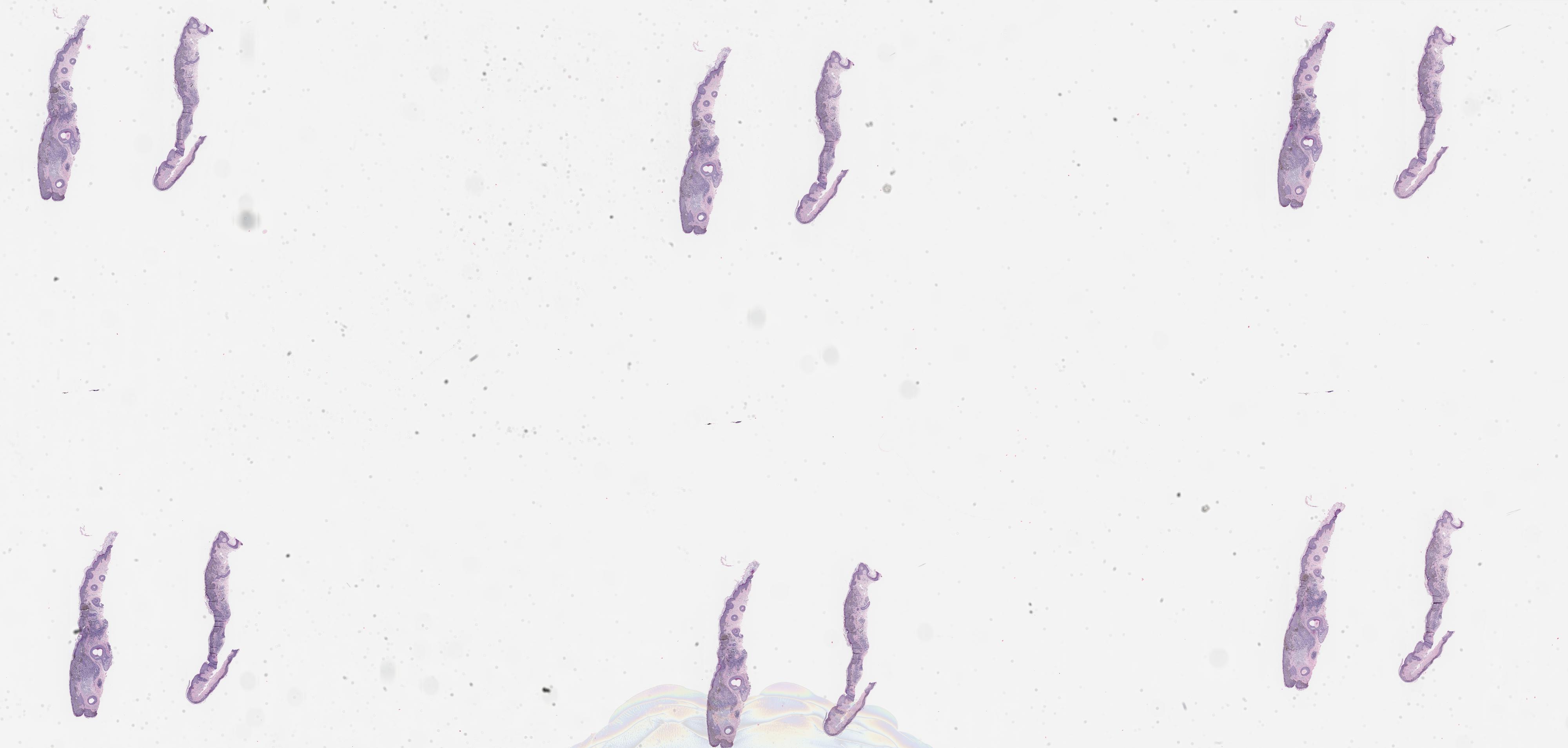

Supplement: Data S1. Illustrative low-resolution summary views of archival H&E-IHC whole slide image pairs, related to STAR Methods and Figure 1 — Details available in Tables S1 and S2. [file mmc2.zip › WSI-48_HE.jpg]

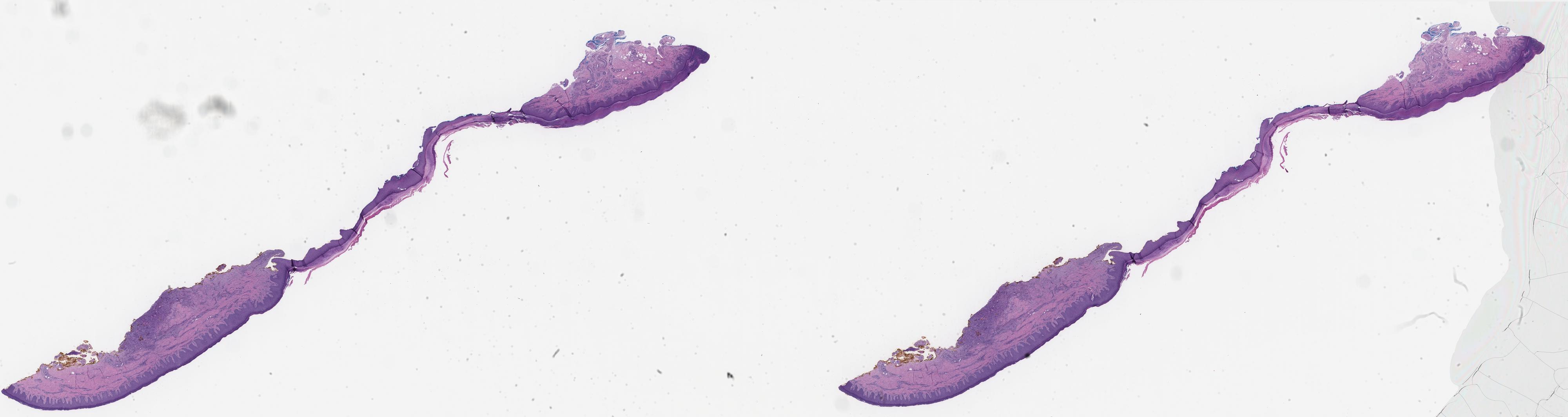

Supplement: Data S1. Illustrative low-resolution summary views of archival H&E-IHC whole slide image pairs, related to STAR Methods and Figure 1 — Details available in Tables S1 and S2. [file mmc2.zip › WSI-39_HE.jpg]

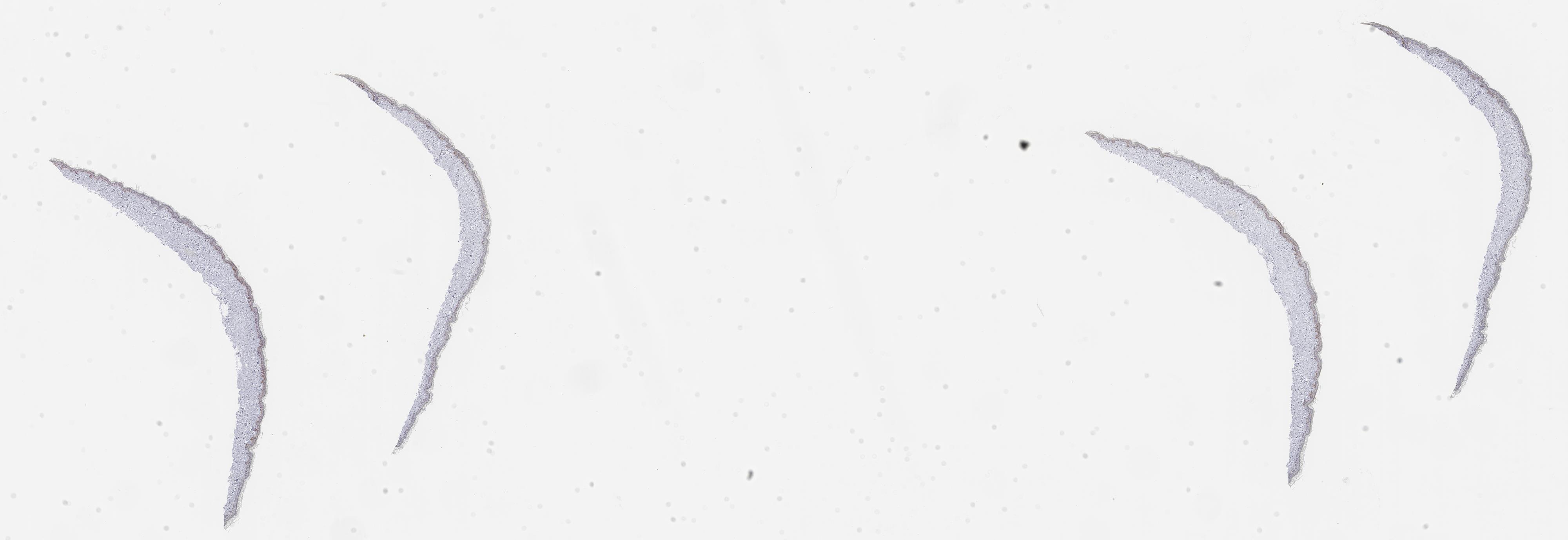

Supplement: Data S1. Illustrative low-resolution summary views of archival H&E-IHC whole slide image pairs, related to STAR Methods and Figure 1 — Details available in Tables S1 and S2. [file mmc2.zip › WSI-46_IHC.jpg]

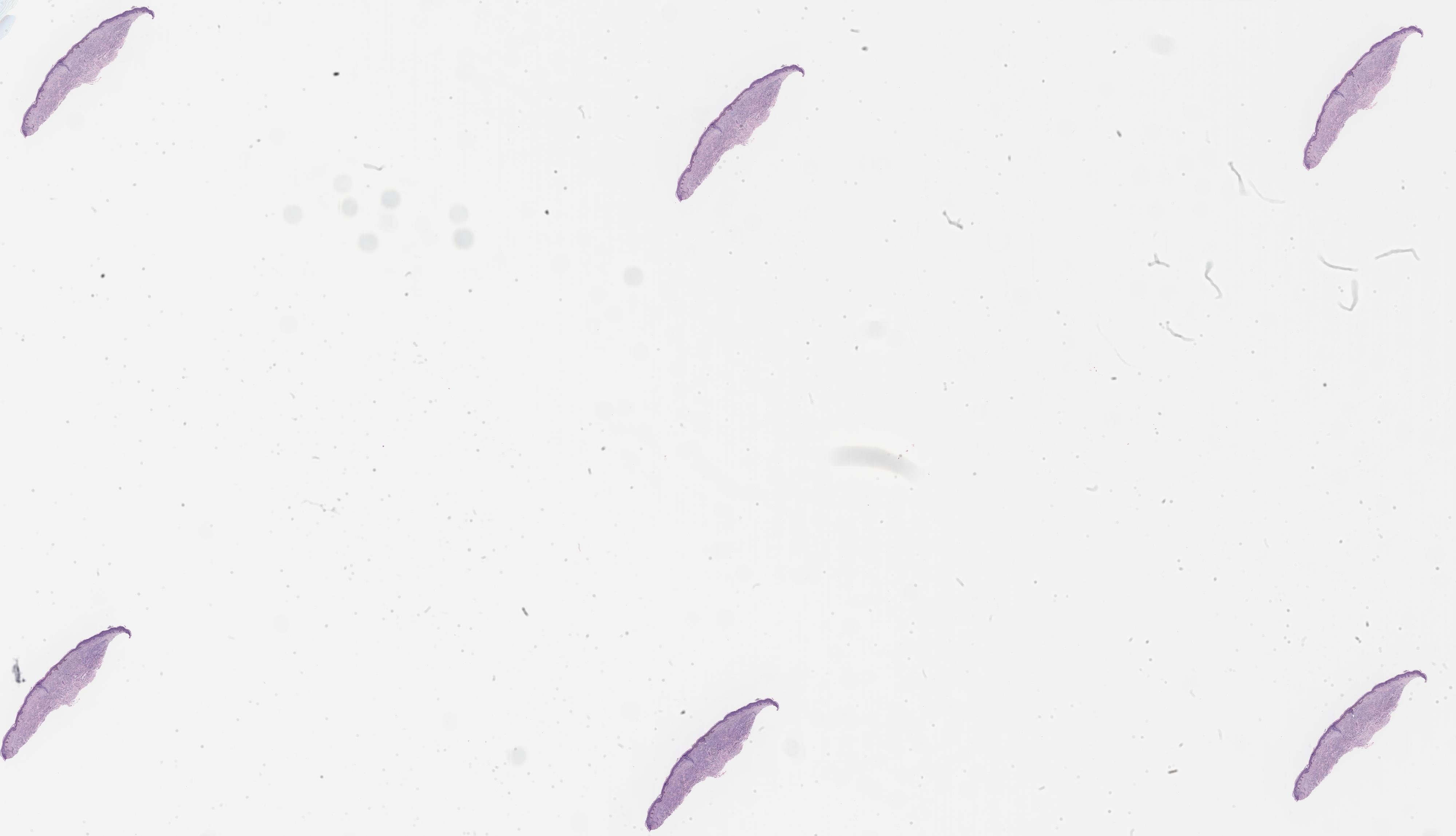

Supplement: Data S1. Illustrative low-resolution summary views of archival H&E-IHC whole slide image pairs, related to STAR Methods and Figure 1 — Details available in Tables S1 and S2. [file mmc2.zip › WSI-44_HE.jpg]

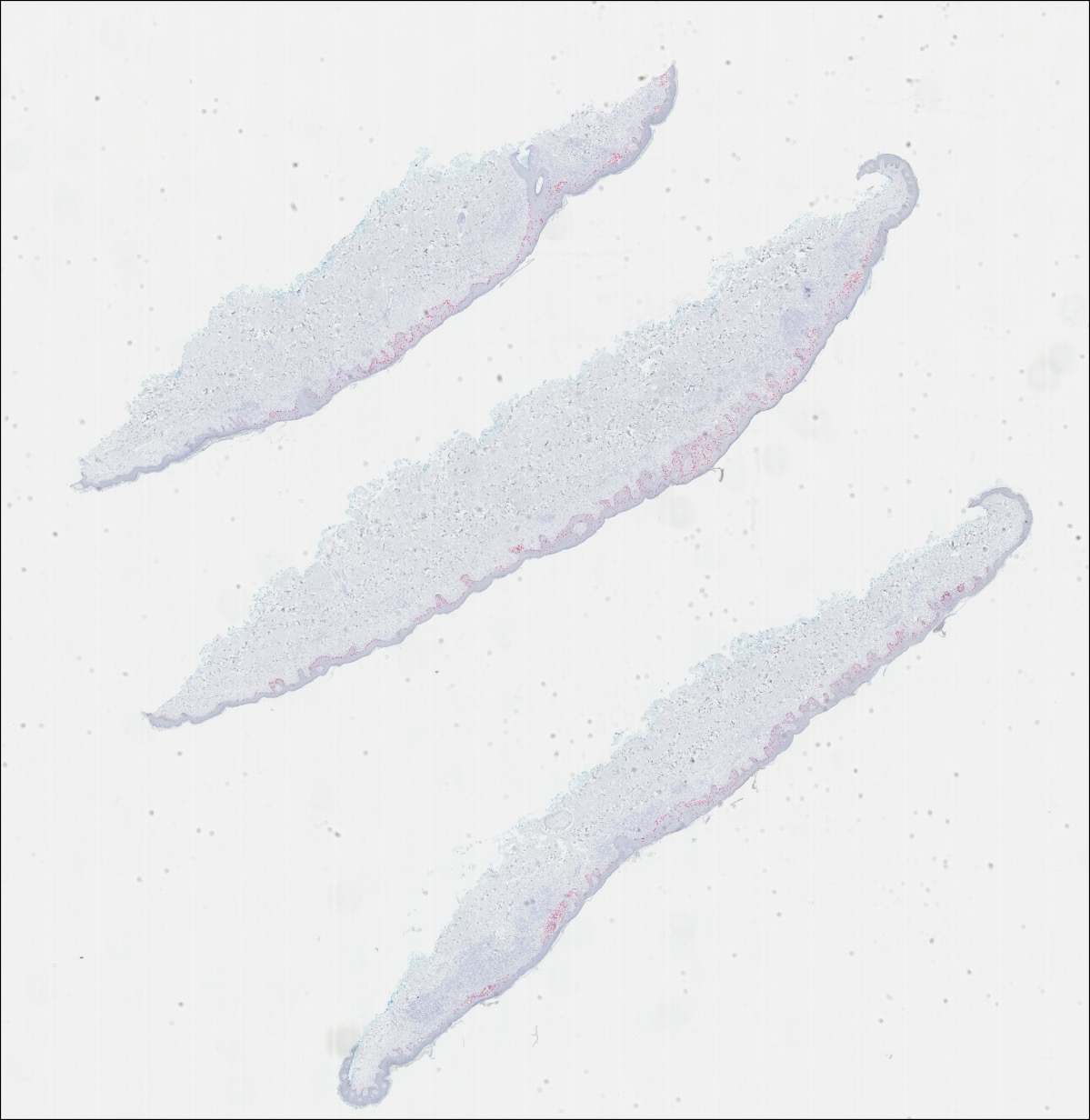

Supplement: Data S1. Illustrative low-resolution summary views of archival H&E-IHC whole slide image pairs, related to STAR Methods and Figure 1 — Details available in Tables S1 and S2. [file mmc2.zip › WSI-56_IHC.jpg]

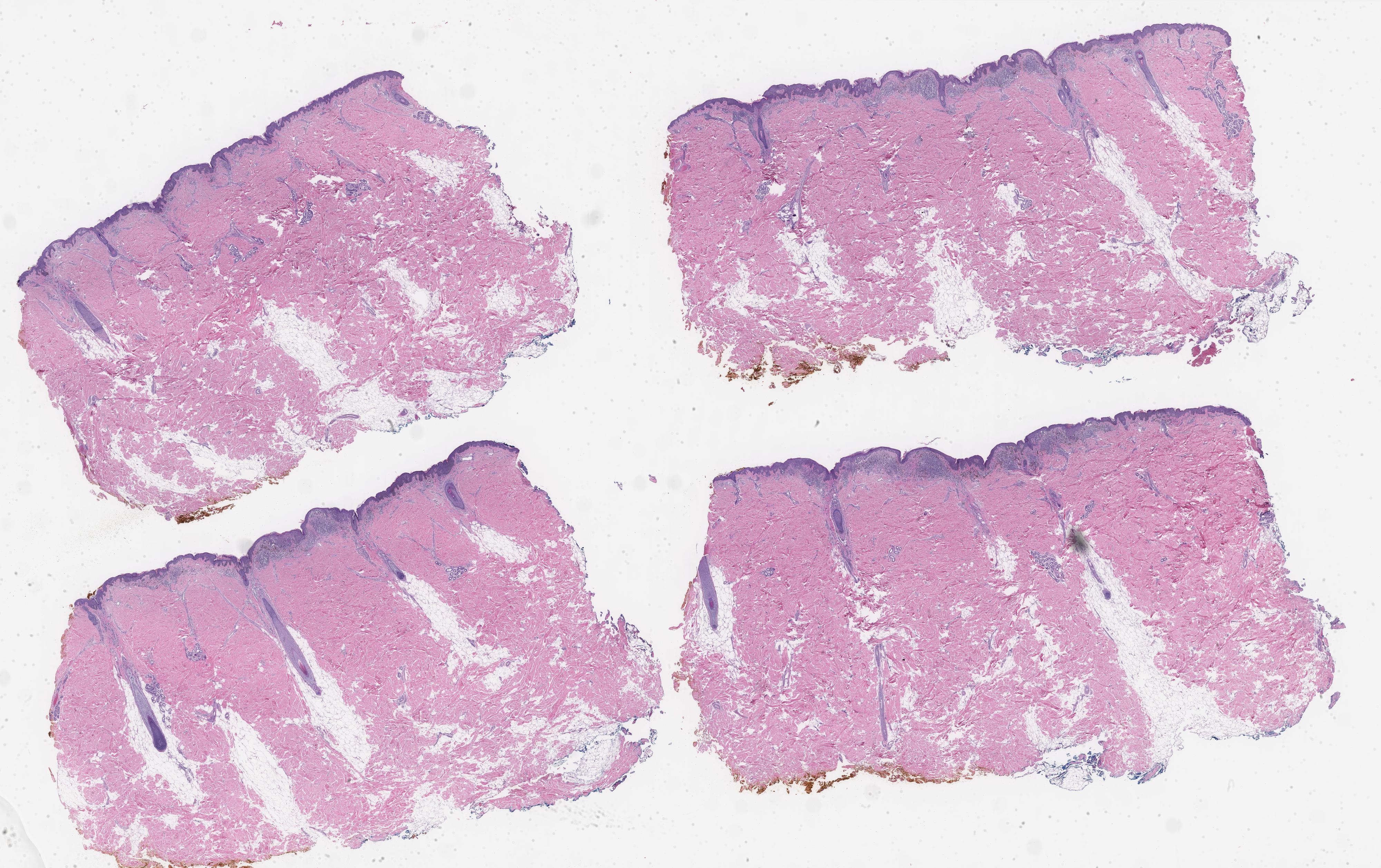

Supplement: Data S1. Illustrative low-resolution summary views of archival H&E-IHC whole slide image pairs, related to STAR Methods and Figure 1 — Details available in Tables S1 and S2. [file mmc2.zip › WSI-11_HE.jpg]

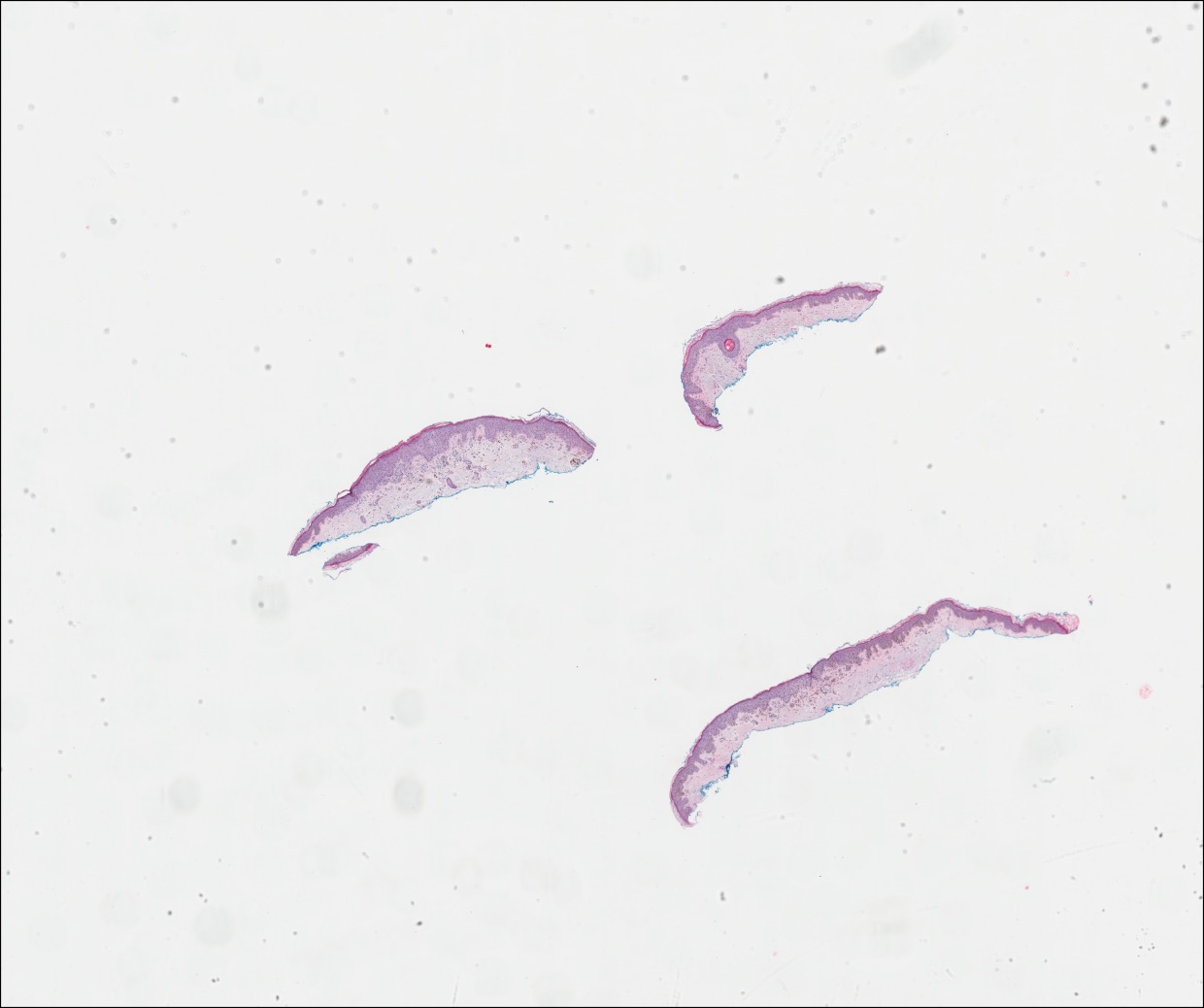

Supplement: Data S1. Illustrative low-resolution summary views of archival H&E-IHC whole slide image pairs, related to STAR Methods and Figure 1 — Details available in Tables S1 and S2. [file mmc2.zip › WSI-60_HE.jpg]

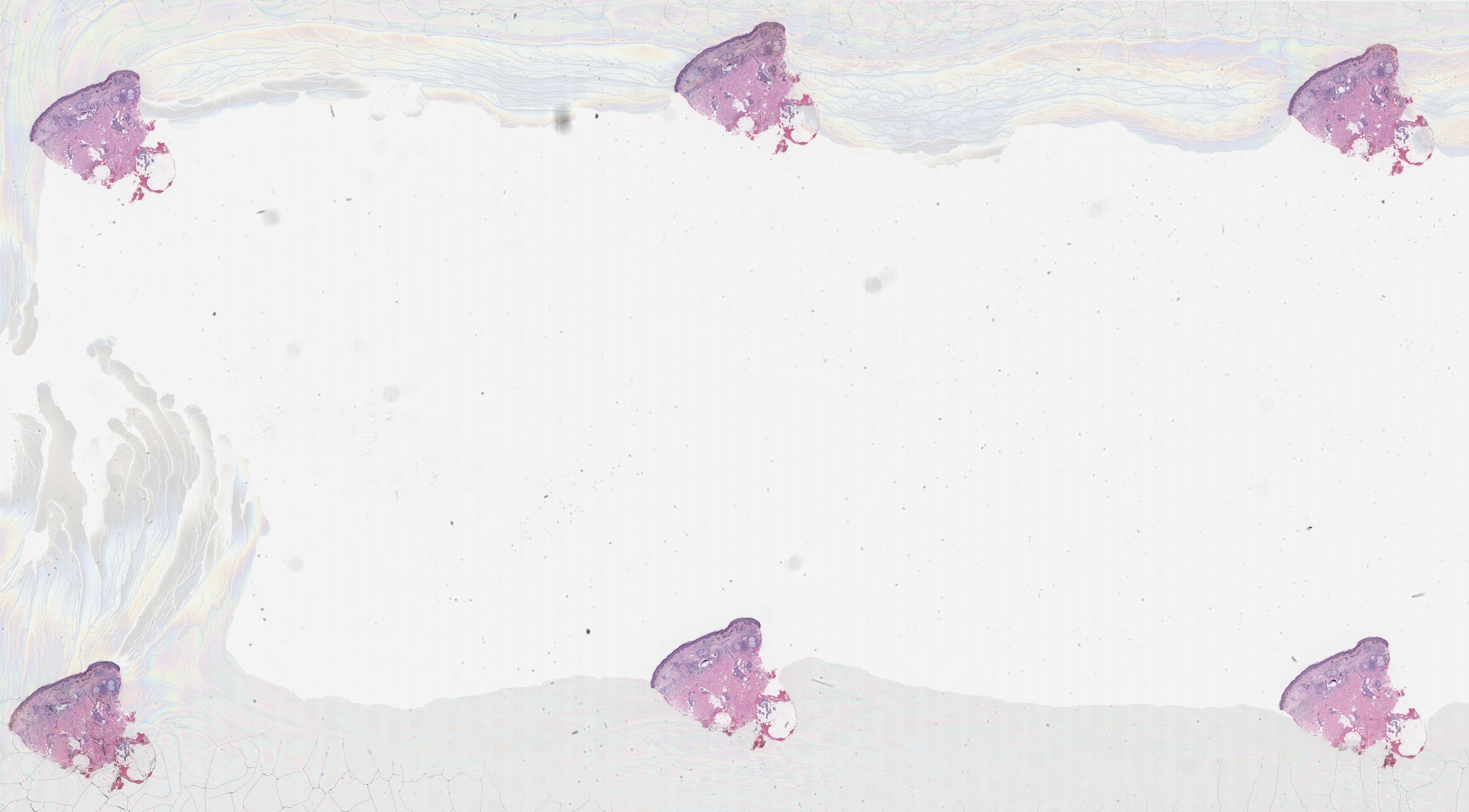

Supplement: Data S1. Illustrative low-resolution summary views of archival H&E-IHC whole slide image pairs, related to STAR Methods and Figure 1 — Details available in Tables S1 and S2. [file mmc2.zip › WSI-03_HE.jpg]

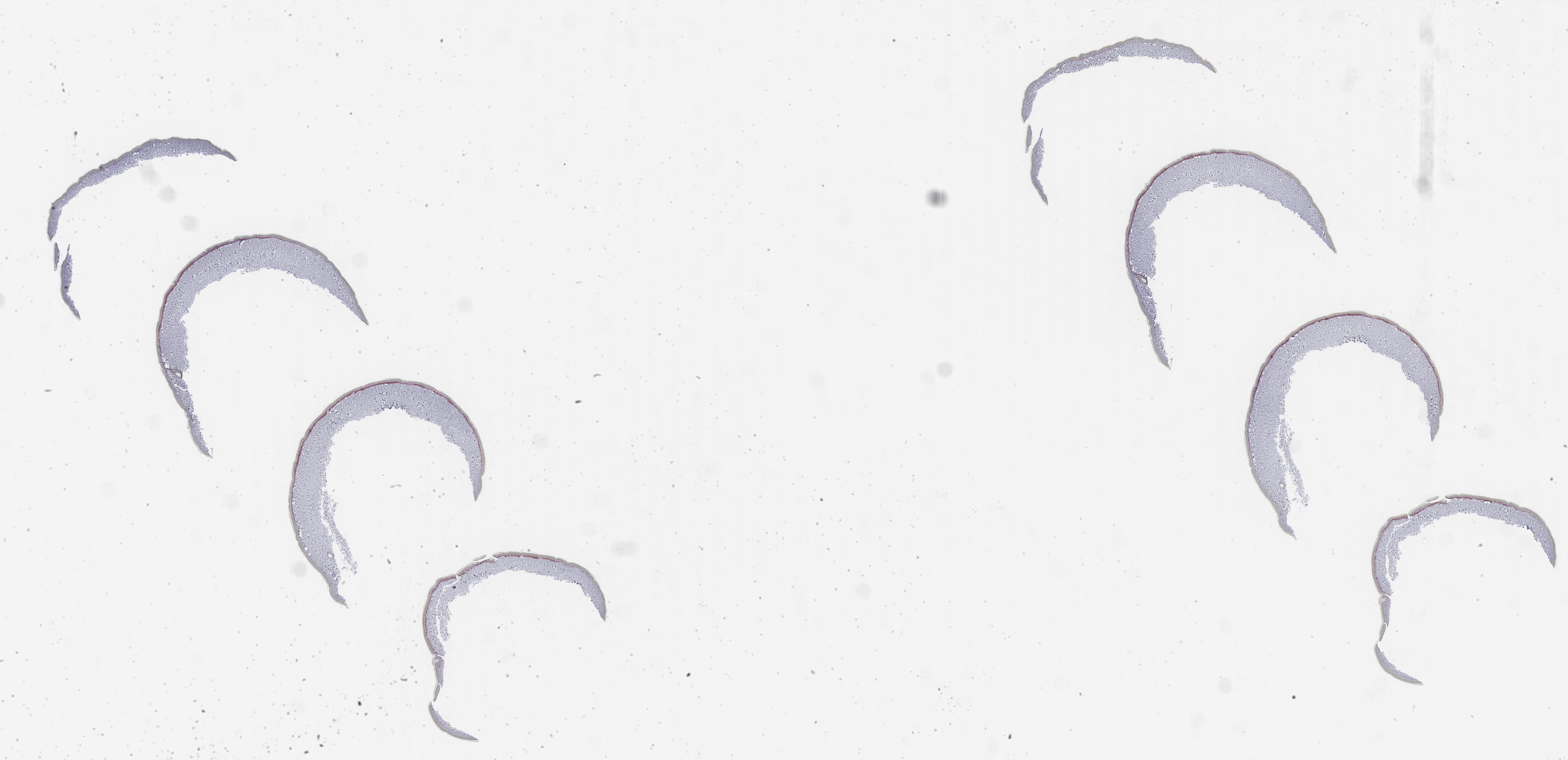

Supplement: Data S1. Illustrative low-resolution summary views of archival H&E-IHC whole slide image pairs, related to STAR Methods and Figure 1 — Details available in Tables S1 and S2. [file mmc2.zip › WSI-10_IHC.jpg]

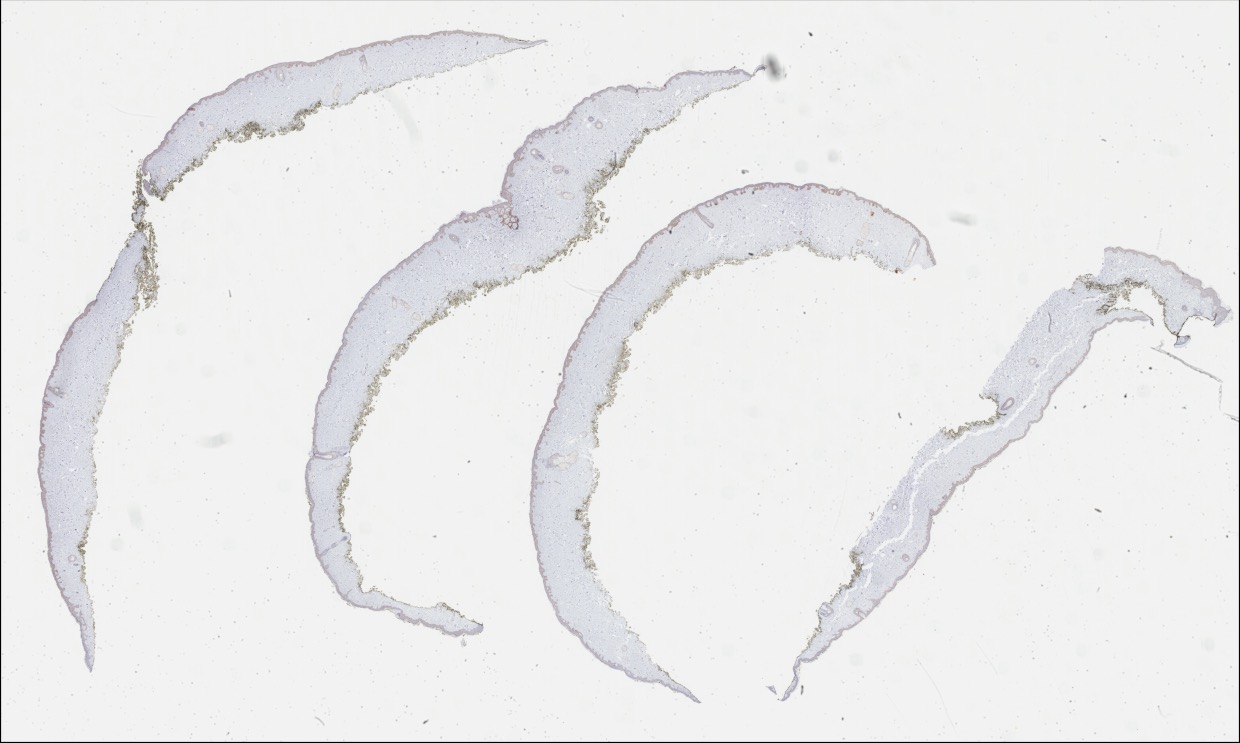

Supplement: Data S1. Illustrative low-resolution summary views of archival H&E-IHC whole slide image pairs, related to STAR Methods and Figure 1 — Details available in Tables S1 and S2. [file mmc2.zip › WSI-54_IHC.jpg]

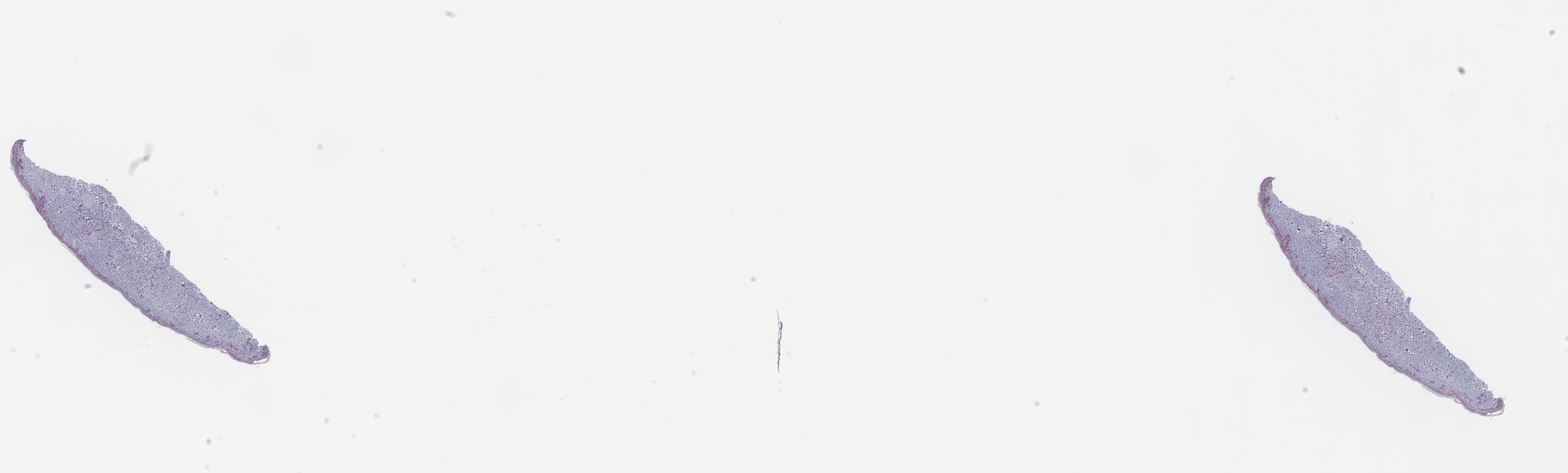

Supplement: Data S1. Illustrative low-resolution summary views of archival H&E-IHC whole slide image pairs, related to STAR Methods and Figure 1 — Details available in Tables S1 and S2. [file mmc2.zip › WSI-44_IHC.jpg]

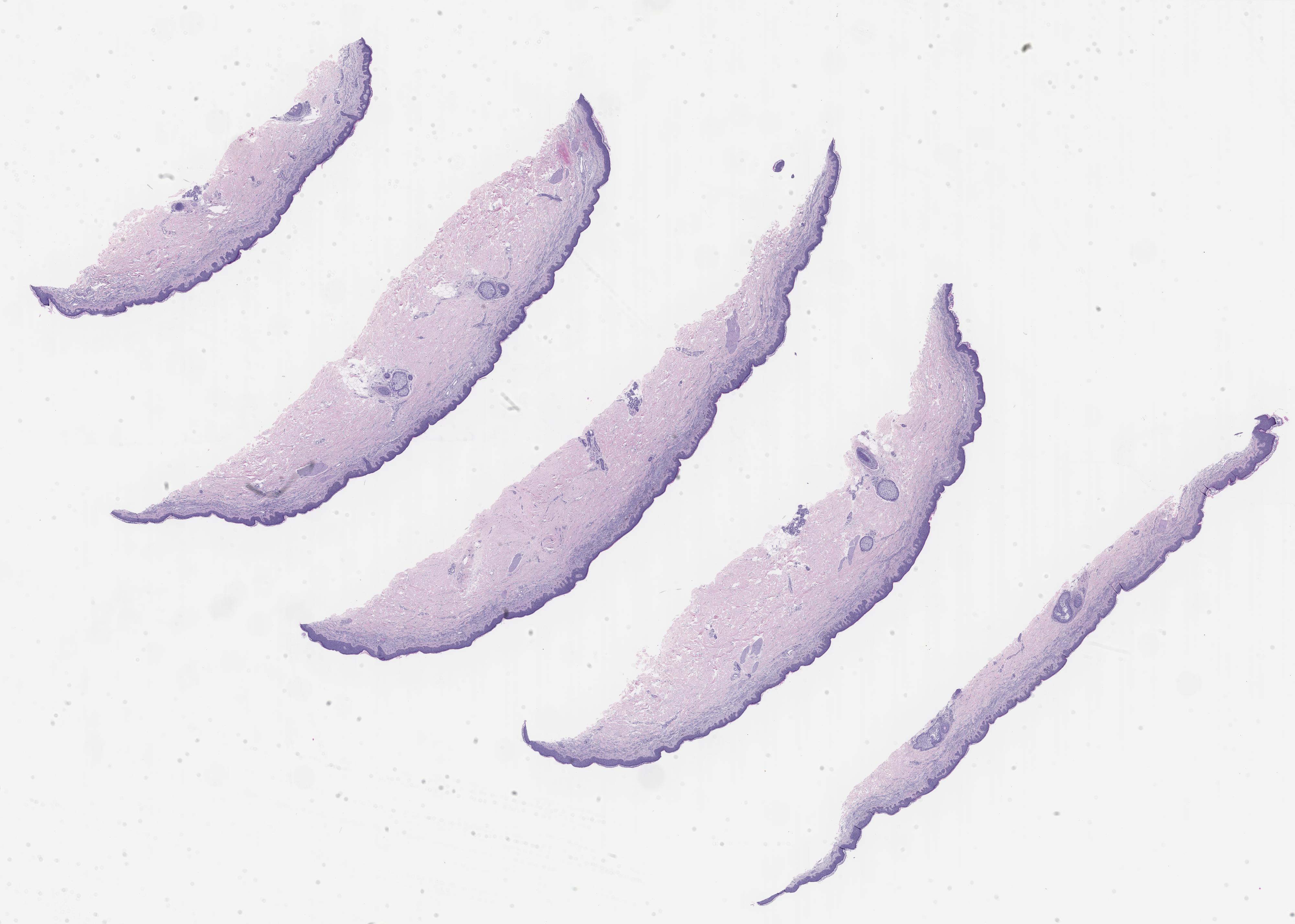

Supplement: Data S1. Illustrative low-resolution summary views of archival H&E-IHC whole slide image pairs, related to STAR Methods and Figure 1 — Details available in Tables S1 and S2. [file mmc2.zip › WSI-19_HE.jpg]

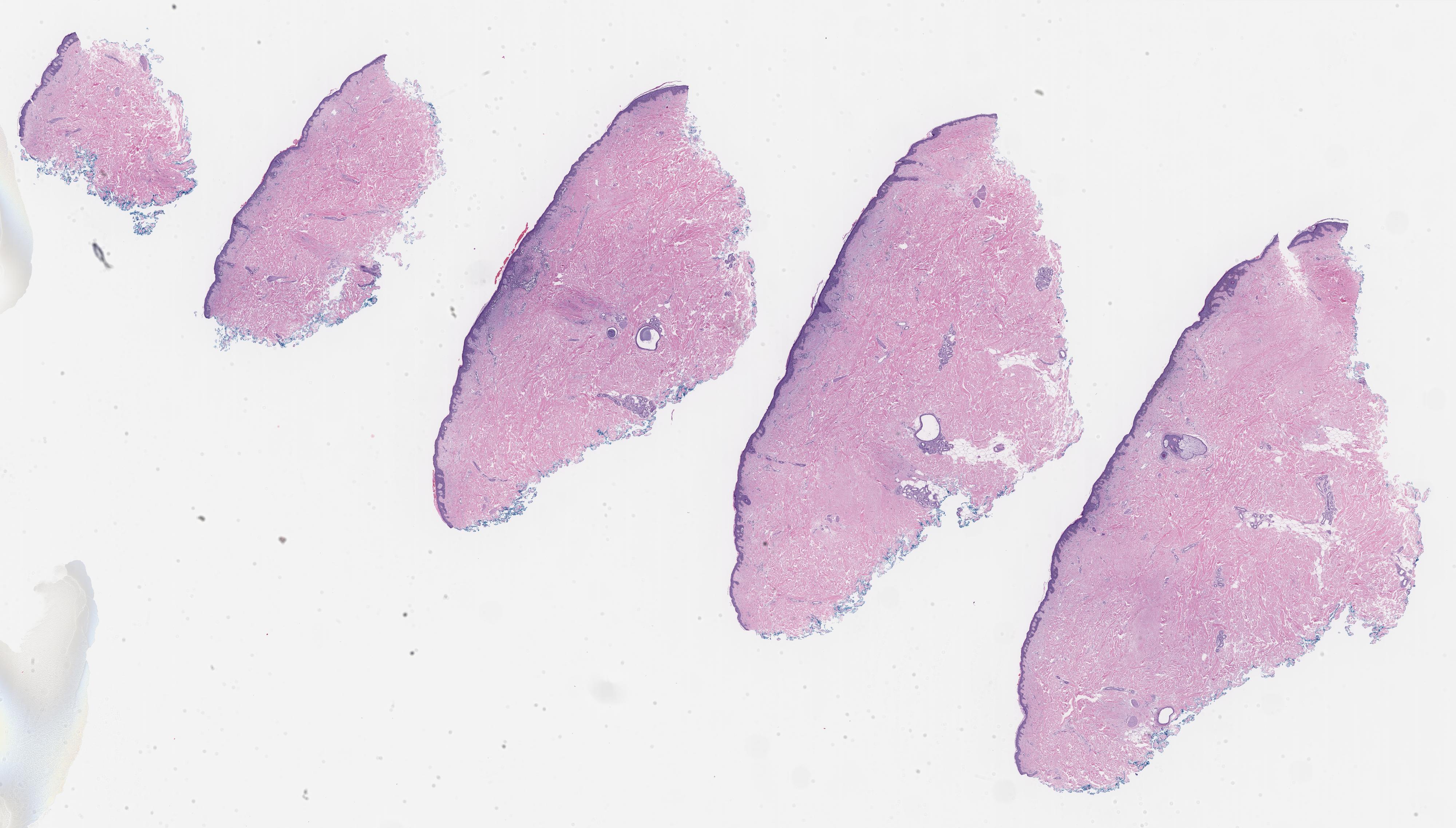

Supplement: Data S1. Illustrative low-resolution summary views of archival H&E-IHC whole slide image pairs, related to STAR Methods and Figure 1 — Details available in Tables S1 and S2. [file mmc2.zip › WSI-07_HE.jpg]

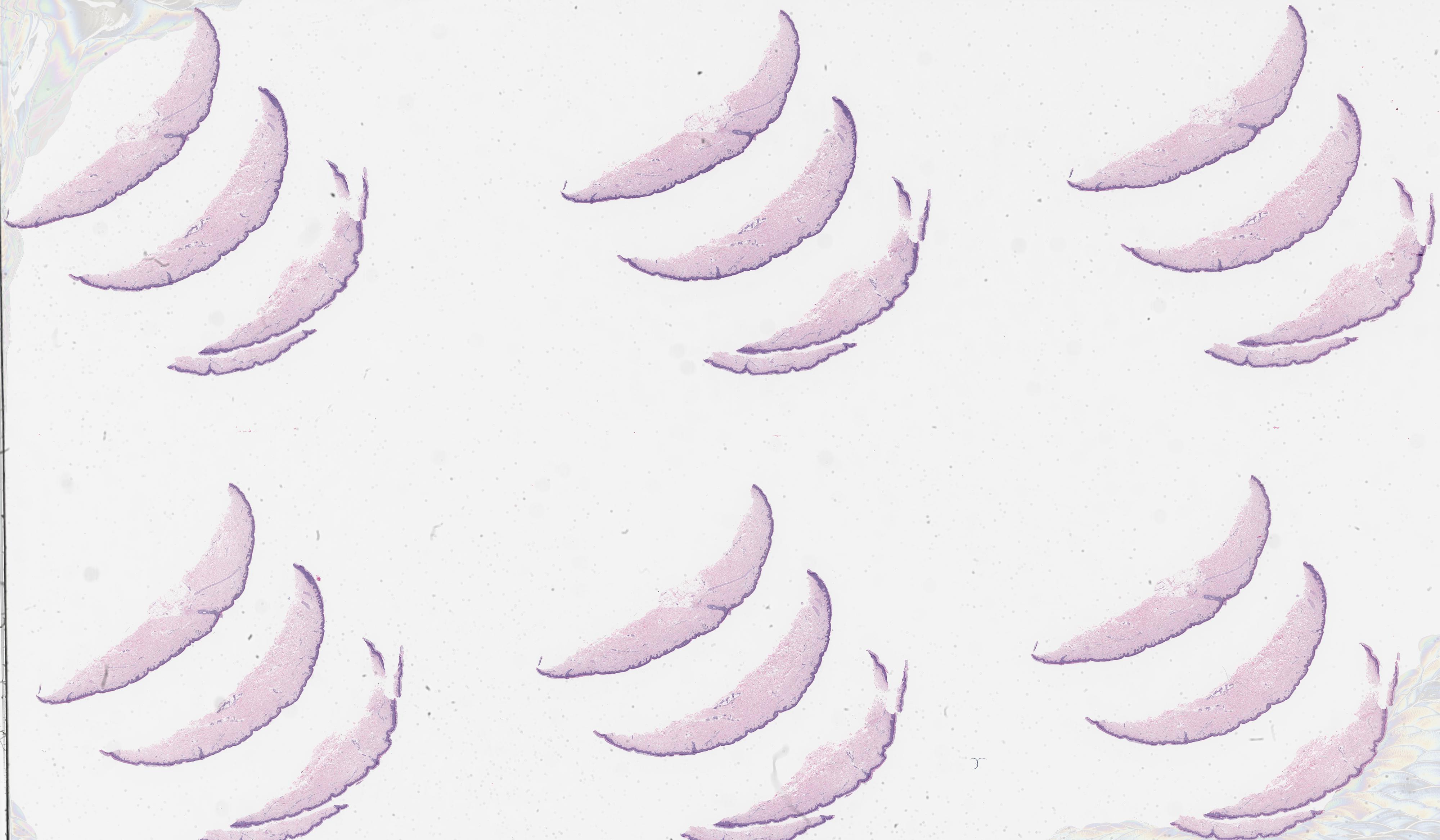

Supplement: Data S1. Illustrative low-resolution summary views of archival H&E-IHC whole slide image pairs, related to STAR Methods and Figure 1 — Details available in Tables S1 and S2. [file mmc2.zip › WSI-15_HE.jpg]

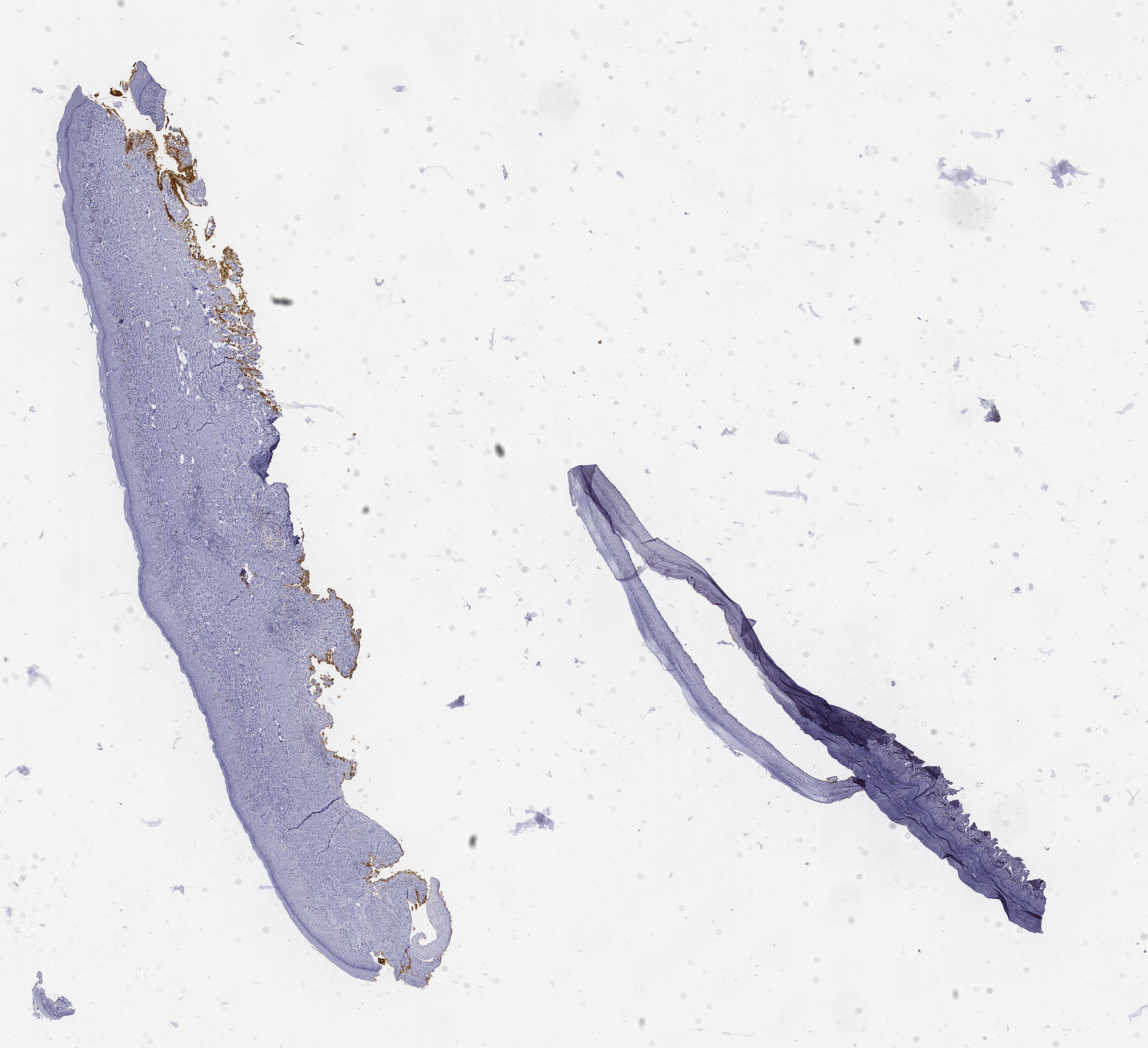

Supplement: Data S1. Illustrative low-resolution summary views of archival H&E-IHC whole slide image pairs, related to STAR Methods and Figure 1 — Details available in Tables S1 and S2. [file mmc2.zip › WSI-36_IHC.jpg]

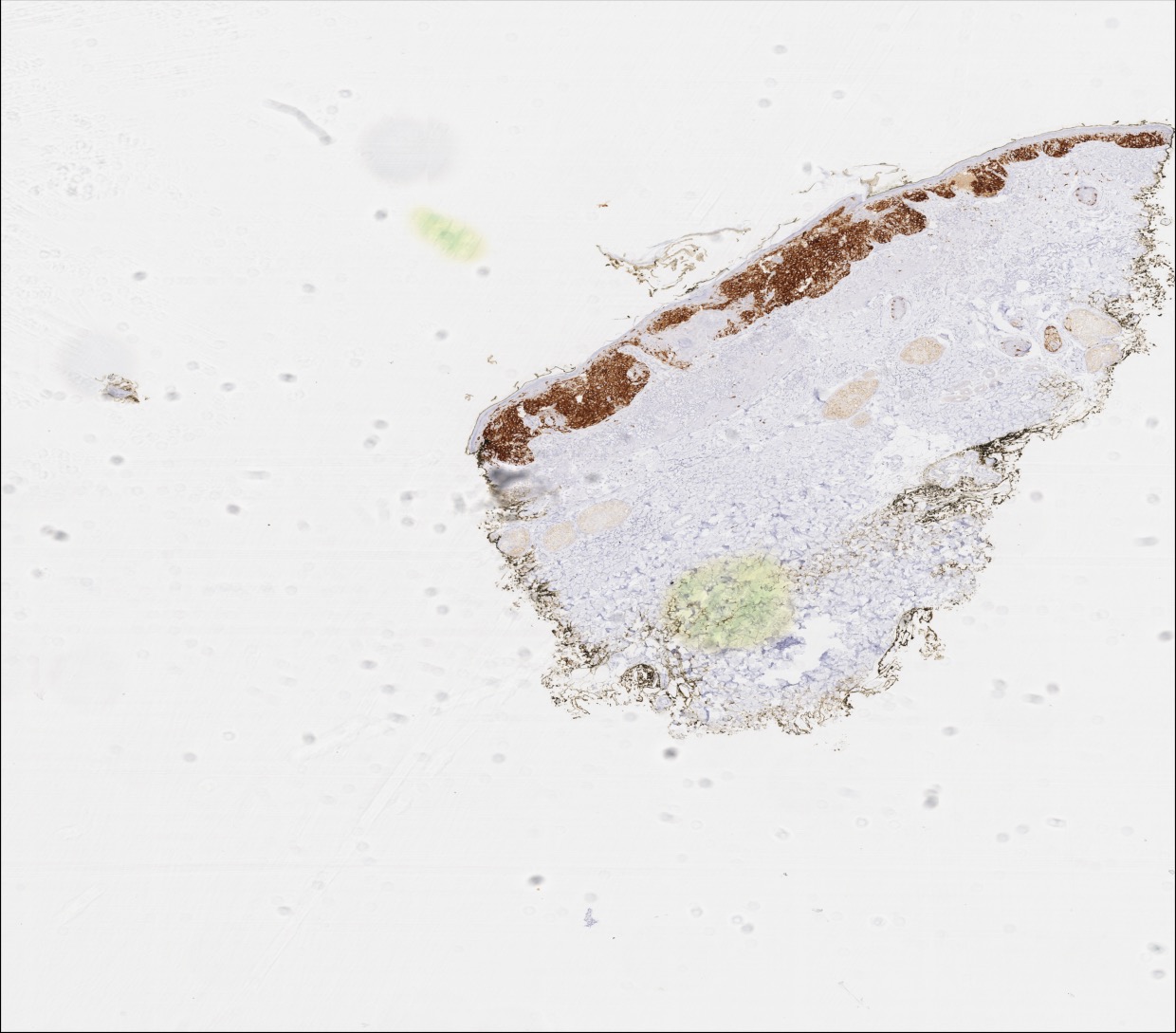

Supplement: Data S1. Illustrative low-resolution summary views of archival H&E-IHC whole slide image pairs, related to STAR Methods and Figure 1 — Details available in Tables S1 and S2. [file mmc2.zip › WSI-26_IHC.jpg]

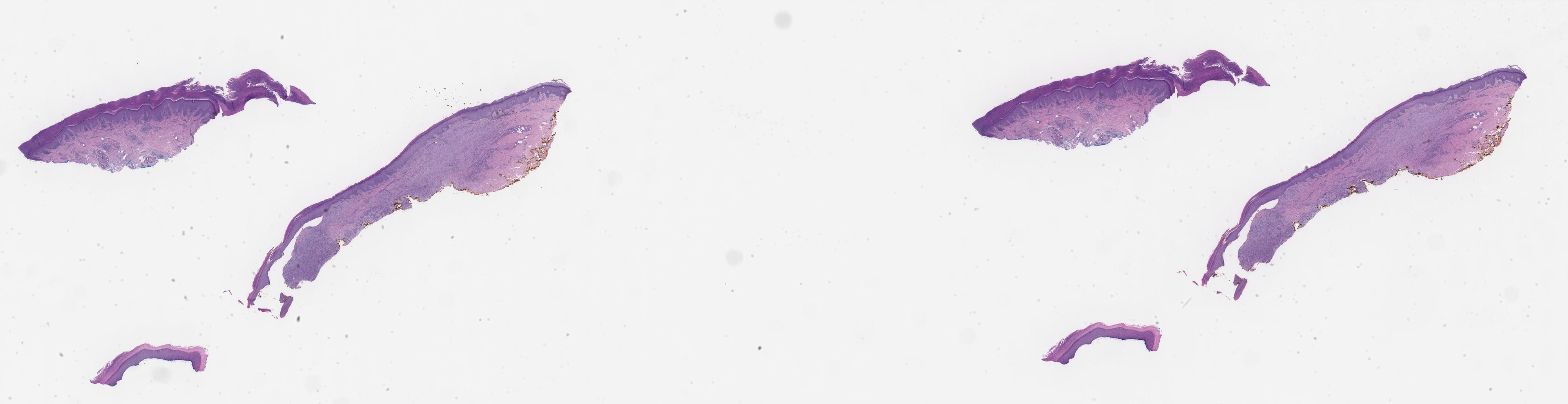

Supplement: Data S1. Illustrative low-resolution summary views of archival H&E-IHC whole slide image pairs, related to STAR Methods and Figure 1 — Details available in Tables S1 and S2. [file mmc2.zip › WSI-40_HE.jpg]

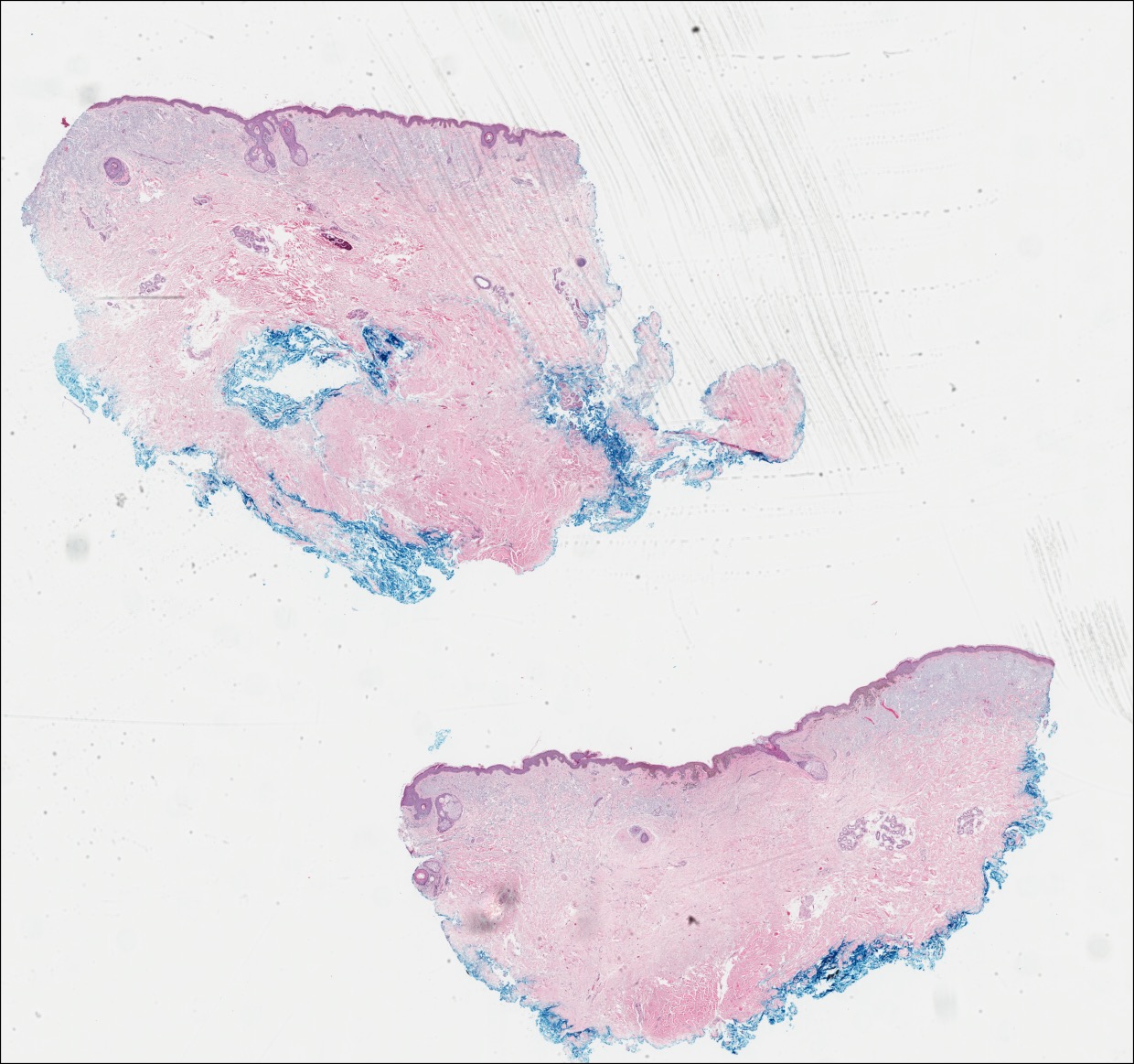

Supplement: Data S1. Illustrative low-resolution summary views of archival H&E-IHC whole slide image pairs, related to STAR Methods and Figure 1 — Details available in Tables S1 and S2. [file mmc2.zip › WSI-23_HE.jpg]

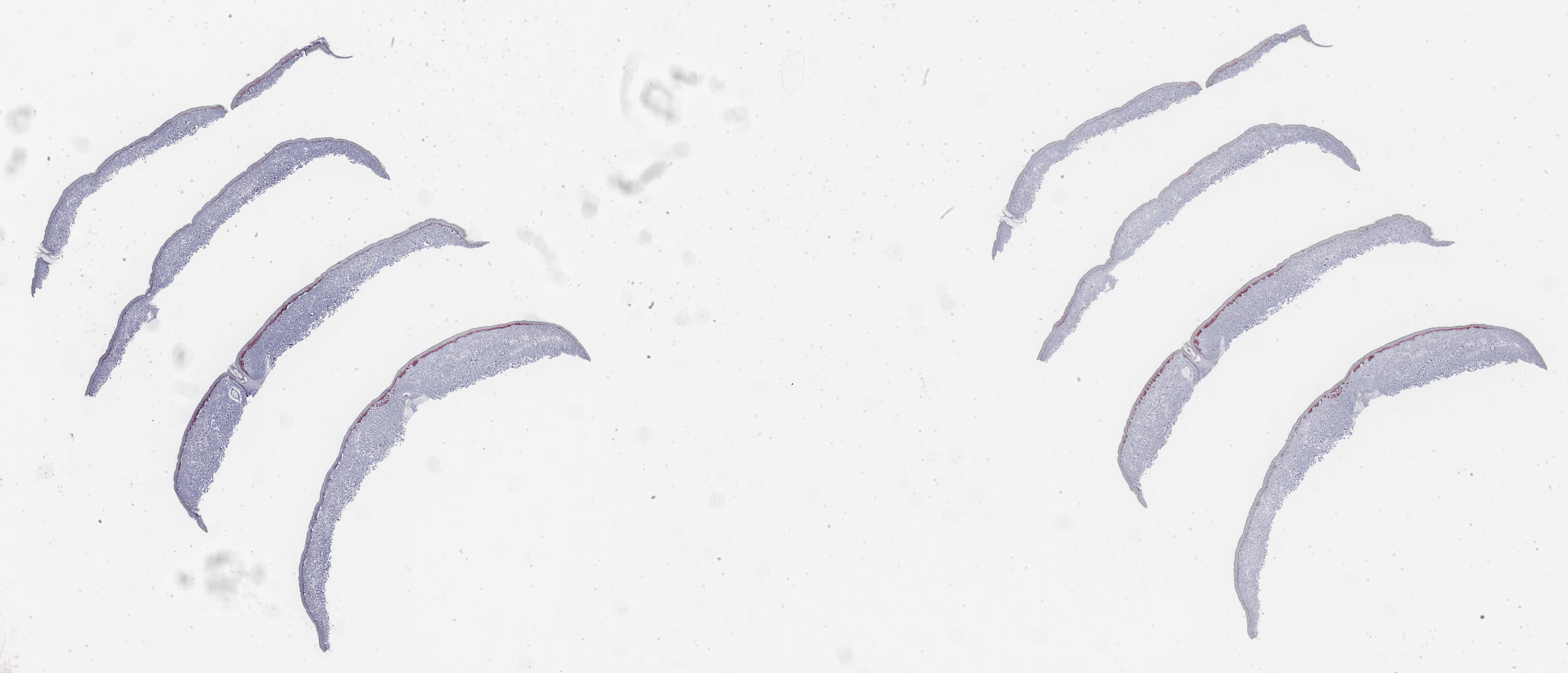

Supplement: Data S1. Illustrative low-resolution summary views of archival H&E-IHC whole slide image pairs, related to STAR Methods and Figure 1 — Details available in Tables S1 and S2. [file mmc2.zip › WSI-09_IHC.jpg]

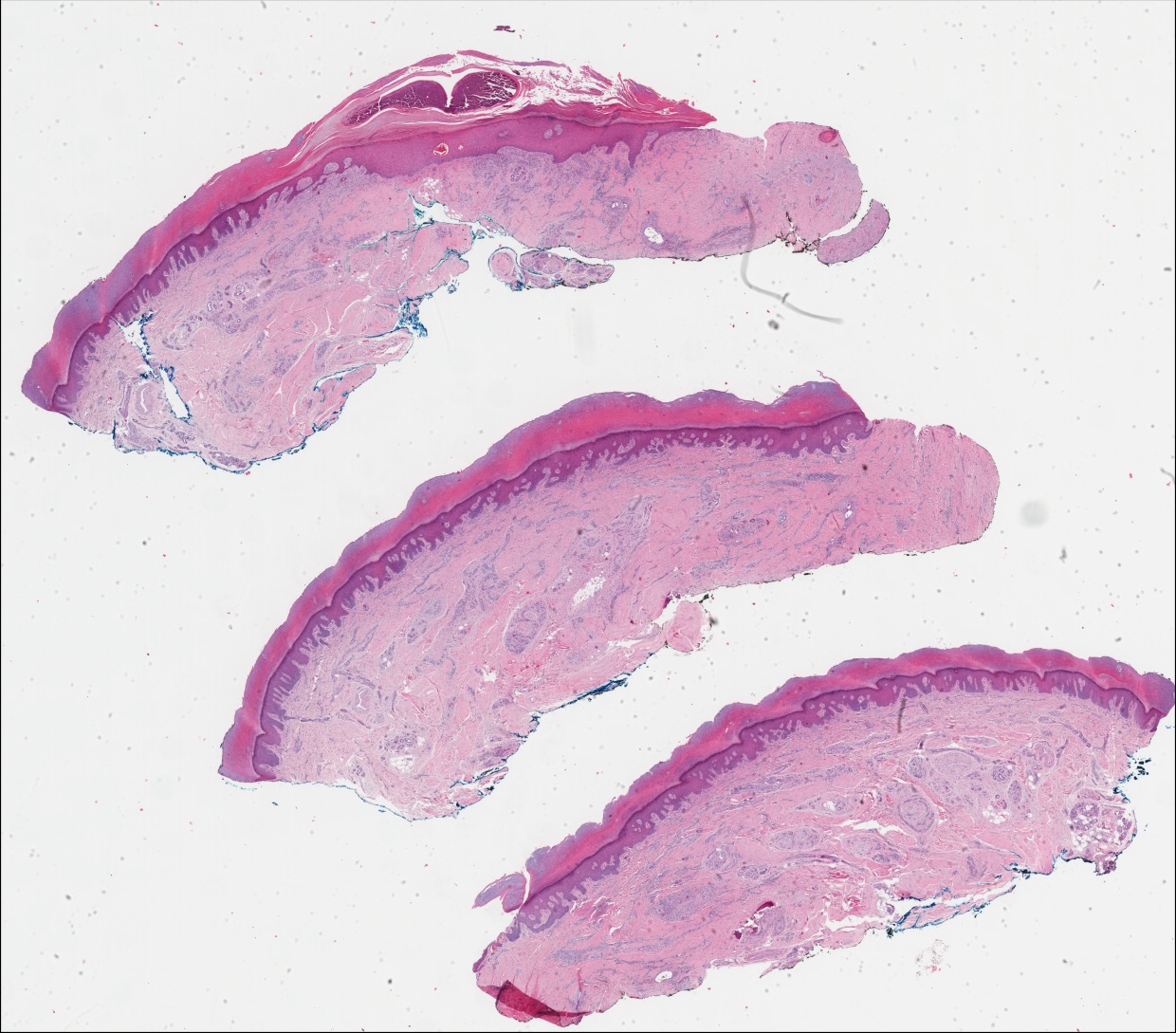

Supplement: Data S1. Illustrative low-resolution summary views of archival H&E-IHC whole slide image pairs, related to STAR Methods and Figure 1 — Details available in Tables S1 and S2. [file mmc2.zip › WSI-52_HE.jpg]

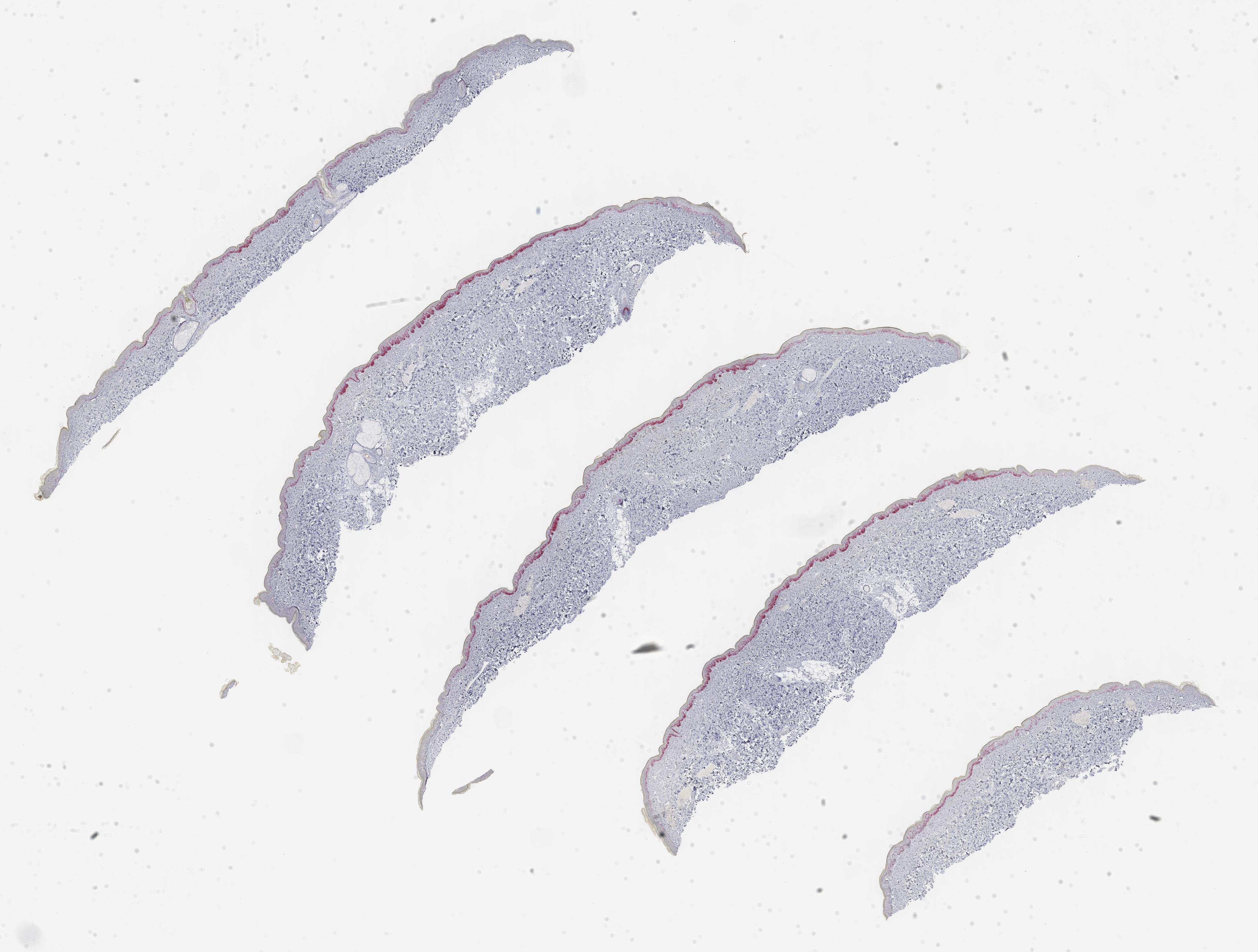

Supplement: Data S1. Illustrative low-resolution summary views of archival H&E-IHC whole slide image pairs, related to STAR Methods and Figure 1 — Details available in Tables S1 and S2. [file mmc2.zip › WSI-19_IHC.jpg]

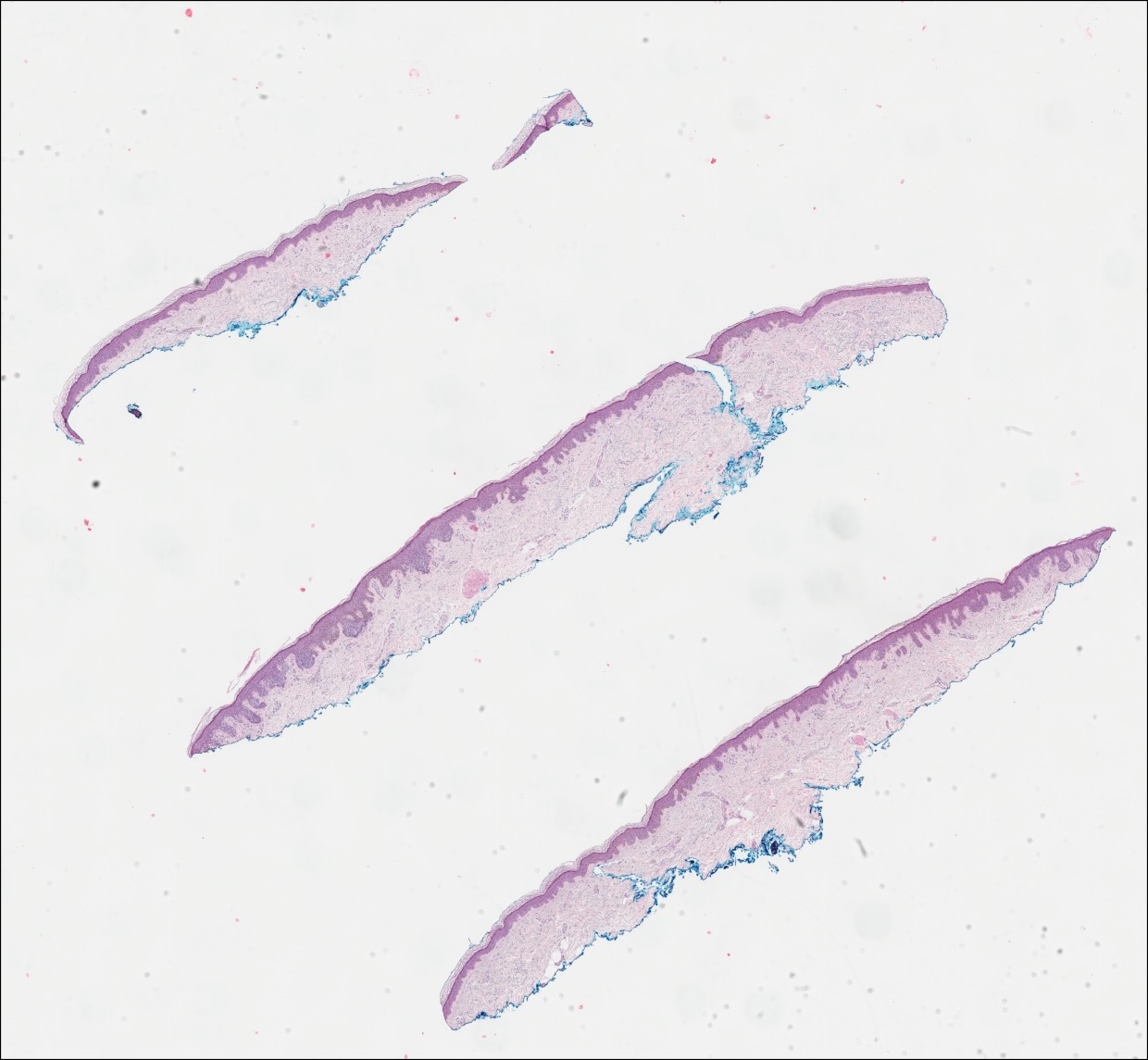

Supplement: Data S1. Illustrative low-resolution summary views of archival H&E-IHC whole slide image pairs, related to STAR Methods and Figure 1 — Details available in Tables S1 and S2. [file mmc2.zip › WSI-31_HE.jpg]

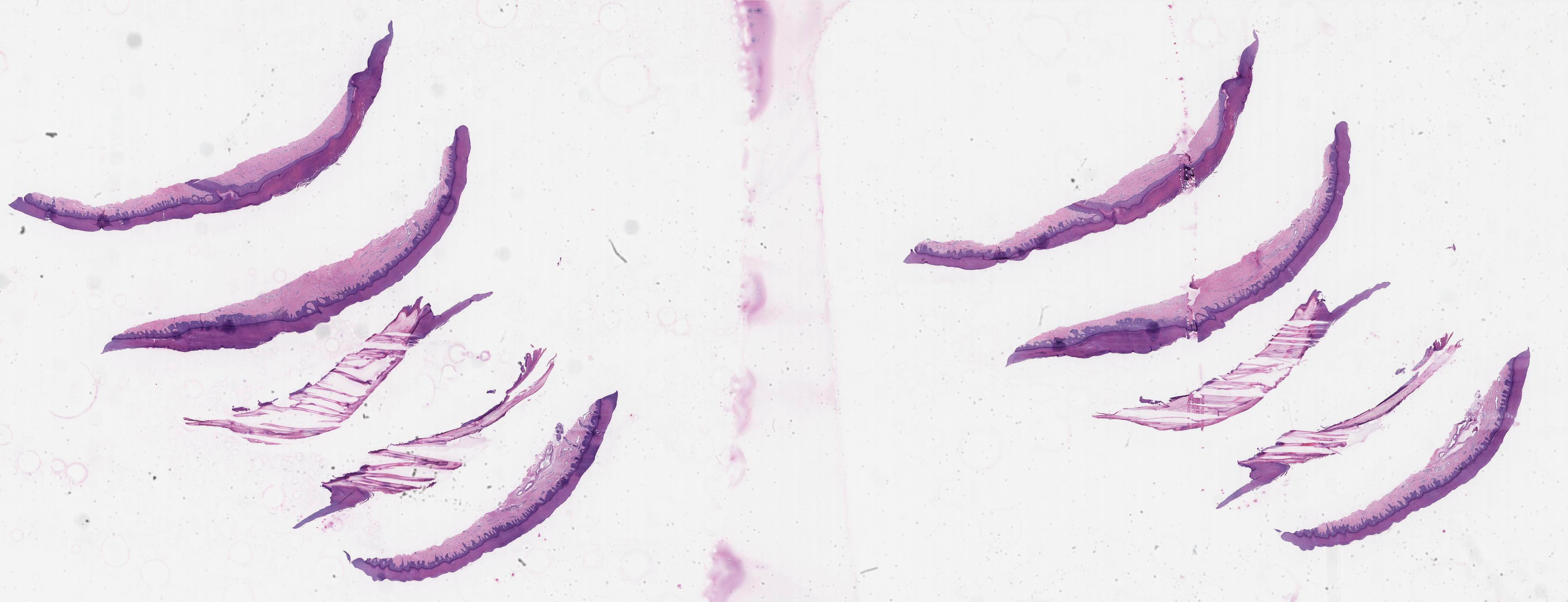

Supplement: Data S1. Illustrative low-resolution summary views of archival H&E-IHC whole slide image pairs, related to STAR Methods and Figure 1 — Details available in Tables S1 and S2. [file mmc2.zip › WSI-17_HE.jpg]

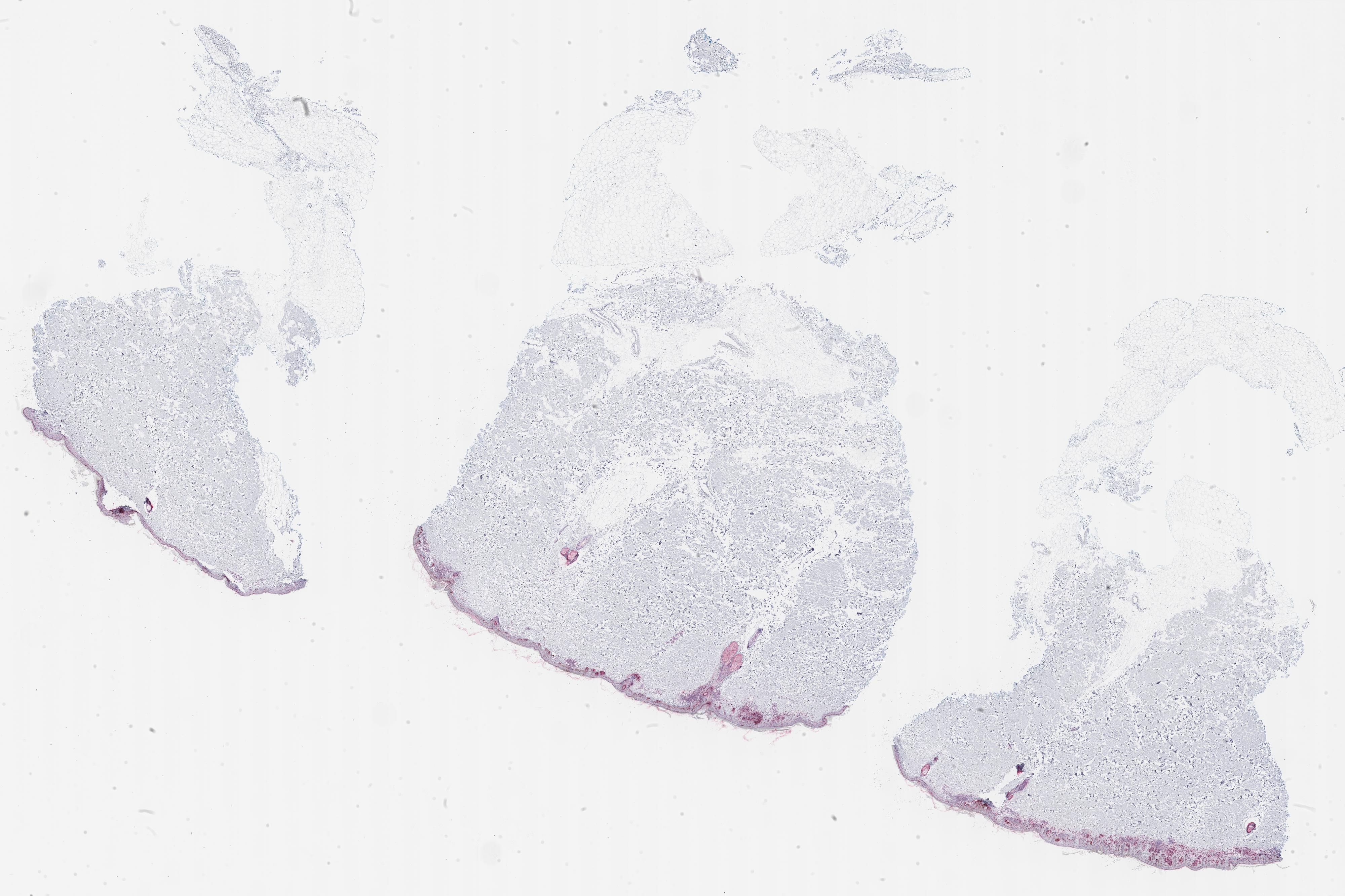

Supplement: Data S1. Illustrative low-resolution summary views of archival H&E-IHC whole slide image pairs, related to STAR Methods and Figure 1 — Details available in Tables S1 and S2. [file mmc2.zip › WSI-45_IHC.jpg]

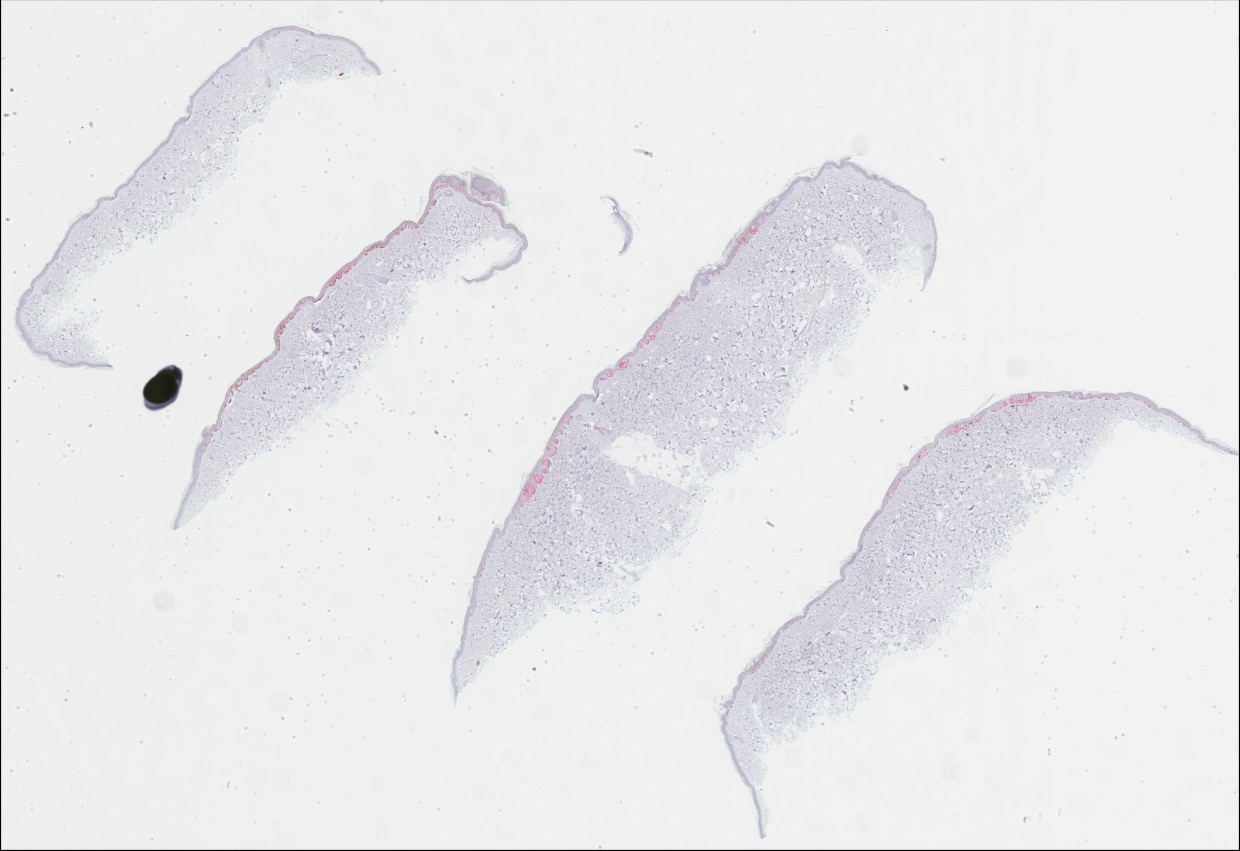

Supplement: Data S1. Illustrative low-resolution summary views of archival H&E-IHC whole slide image pairs, related to STAR Methods and Figure 1 — Details available in Tables S1 and S2. [file mmc2.zip › WSI-55_IHC.jpg]

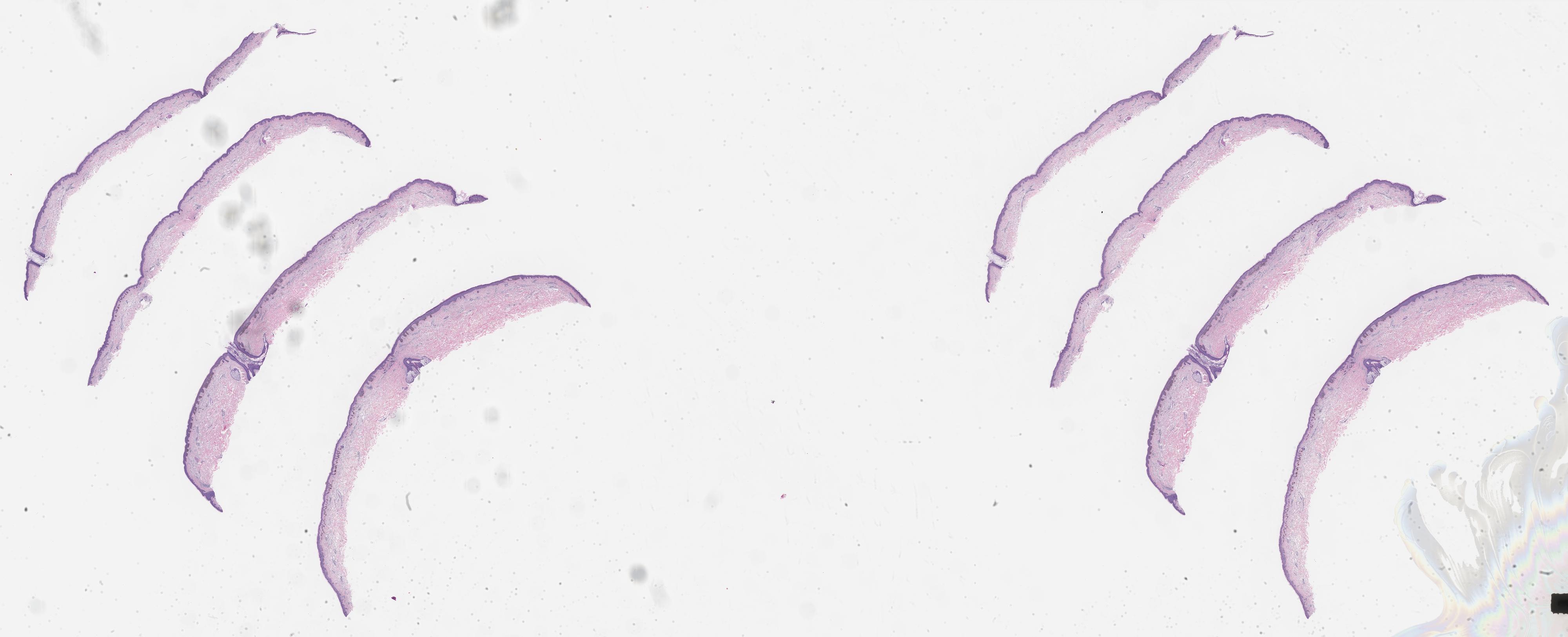

Supplement: Data S1. Illustrative low-resolution summary views of archival H&E-IHC whole slide image pairs, related to STAR Methods and Figure 1 — Details available in Tables S1 and S2. [file mmc2.zip › WSI-09_HE.jpg]

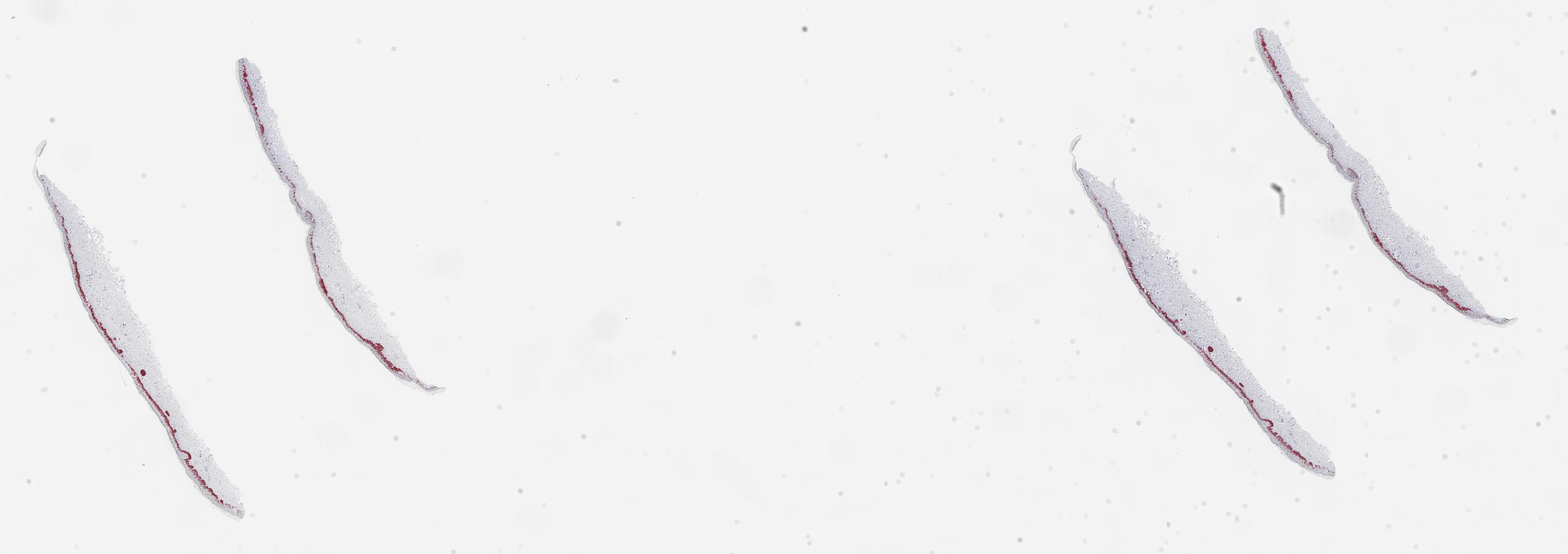

Supplement: Data S1. Illustrative low-resolution summary views of archival H&E-IHC whole slide image pairs, related to STAR Methods and Figure 1 — Details available in Tables S1 and S2. [file mmc2.zip › WSI-27_IHC.jpg]

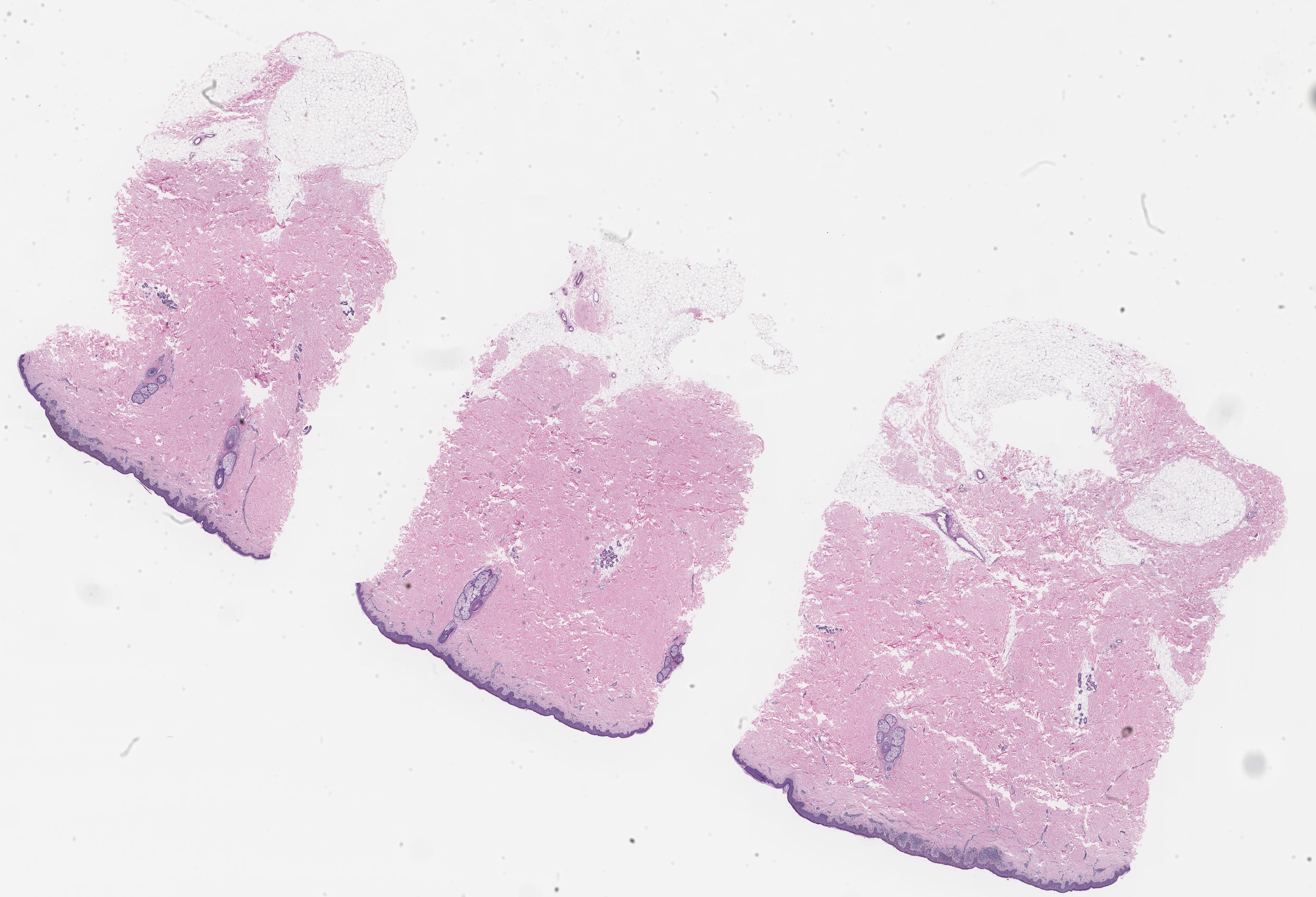

Supplement: Data S1. Illustrative low-resolution summary views of archival H&E-IHC whole slide image pairs, related to STAR Methods and Figure 1 — Details available in Tables S1 and S2. [file mmc2.zip › WSI-05_HE.jpg]

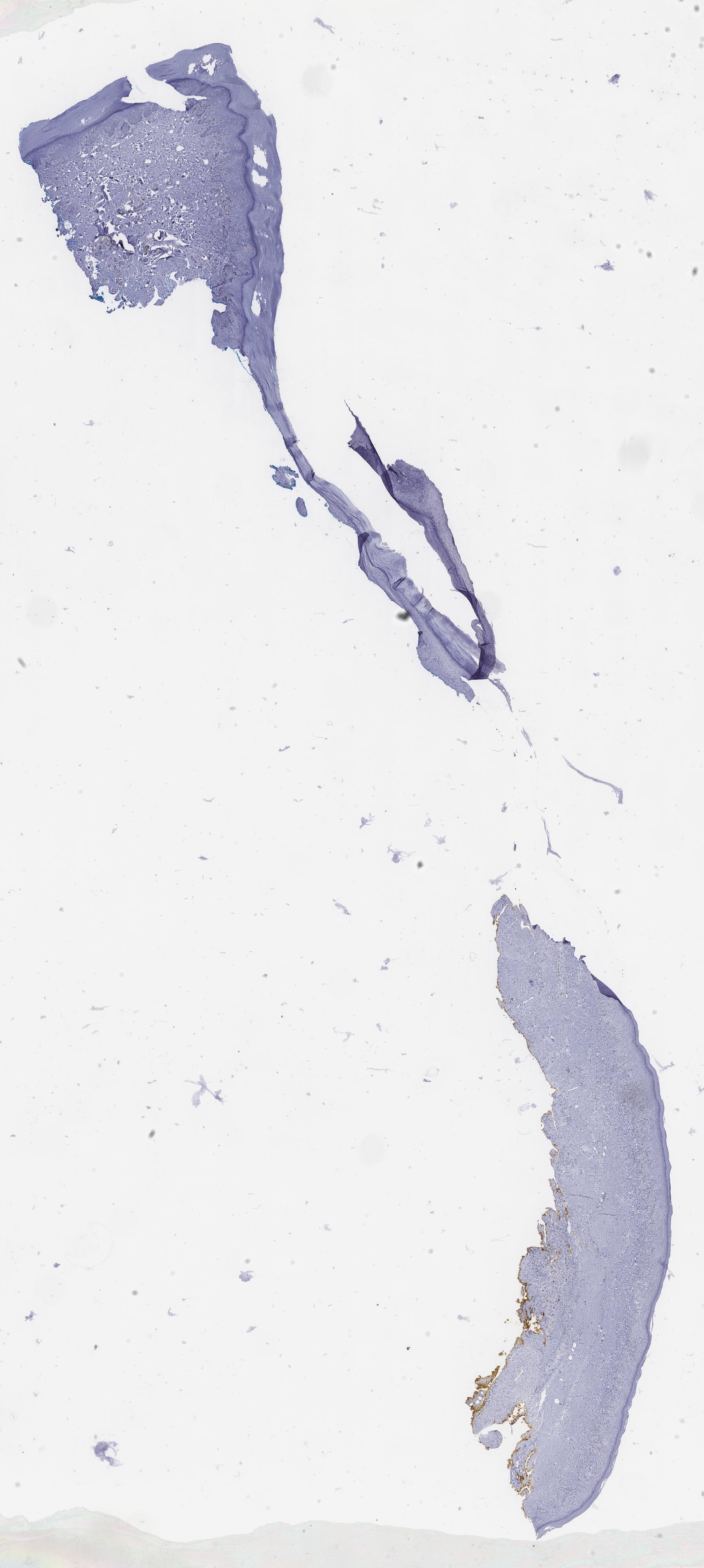

Supplement: Data S1. Illustrative low-resolution summary views of archival H&E-IHC whole slide image pairs, related to STAR Methods and Figure 1 — Details available in Tables S1 and S2. [file mmc2.zip › WSI-37_IHC.jpg]

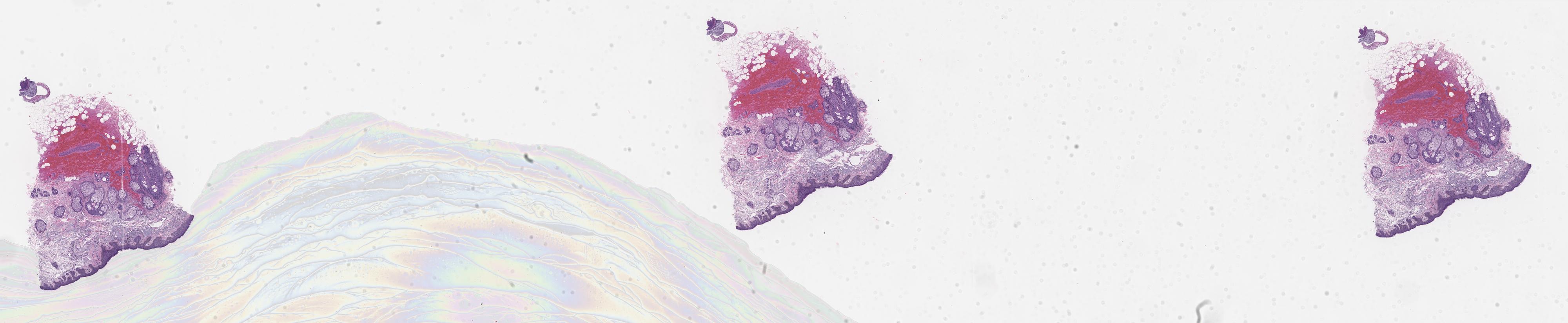

Supplement: Data S1. Illustrative low-resolution summary views of archival H&E-IHC whole slide image pairs, related to STAR Methods and Figure 1 — Details available in Tables S1 and S2. [file mmc2.zip › WSI-33_HE.jpg]

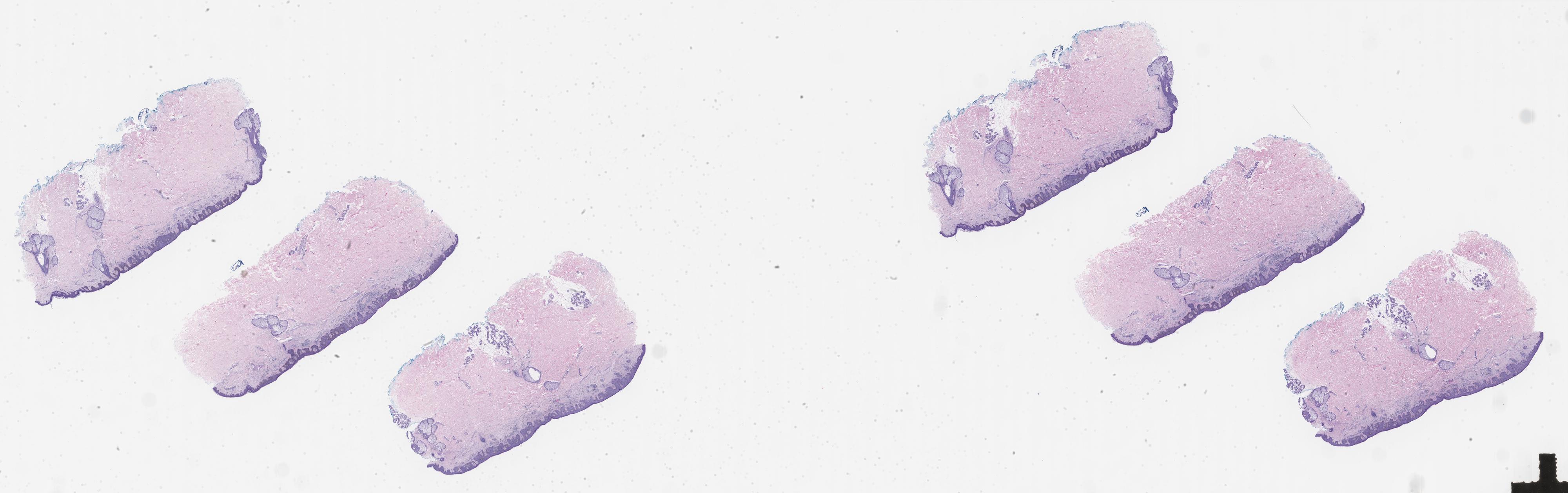

Supplement: Data S1. Illustrative low-resolution summary views of archival H&E-IHC whole slide image pairs, related to STAR Methods and Figure 1 — Details available in Tables S1 and S2. [file mmc2.zip › WSI-50_HE.jpg]

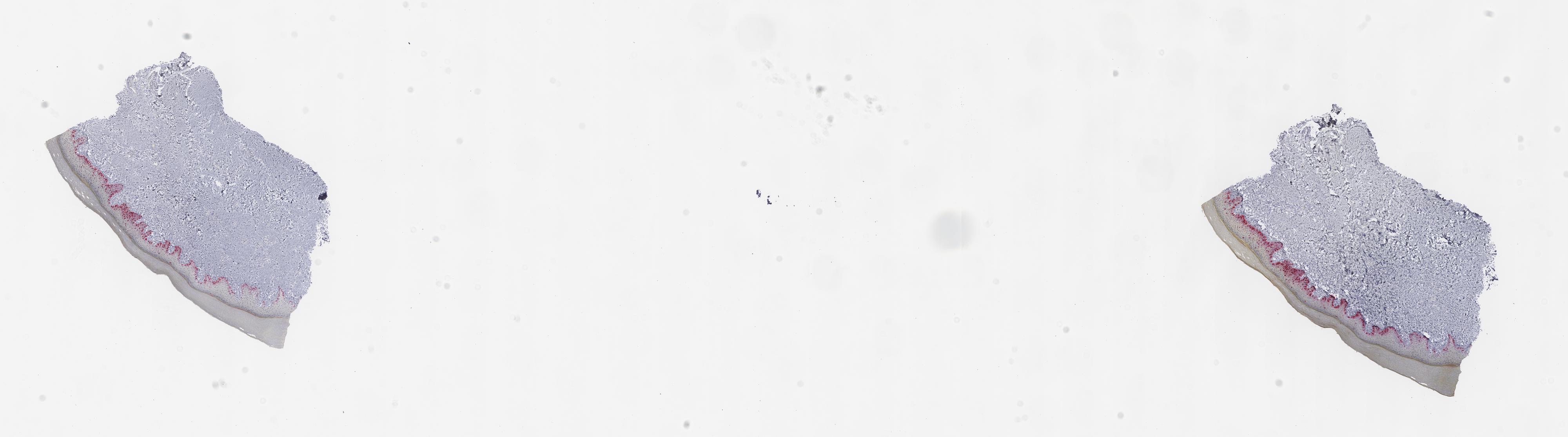

Supplement: Data S1. Illustrative low-resolution summary views of archival H&E-IHC whole slide image pairs, related to STAR Methods and Figure 1 — Details available in Tables S1 and S2. [file mmc2.zip › WSI-18_IHC.jpg]

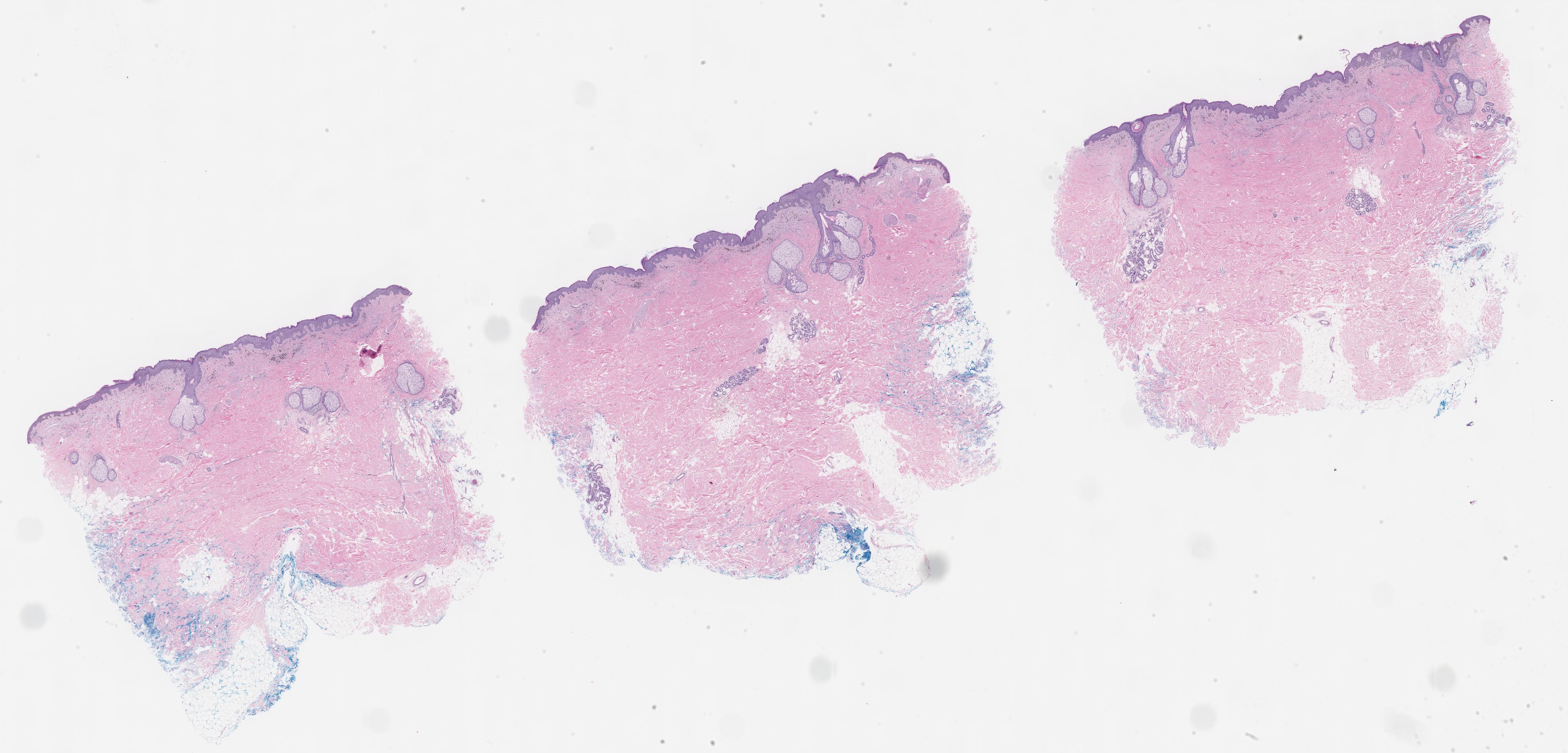

Supplement: Data S1. Illustrative low-resolution summary views of archival H&E-IHC whole slide image pairs, related to STAR Methods and Figure 1 — Details available in Tables S1 and S2. [file mmc2.zip › WSI-42_HE.jpg]

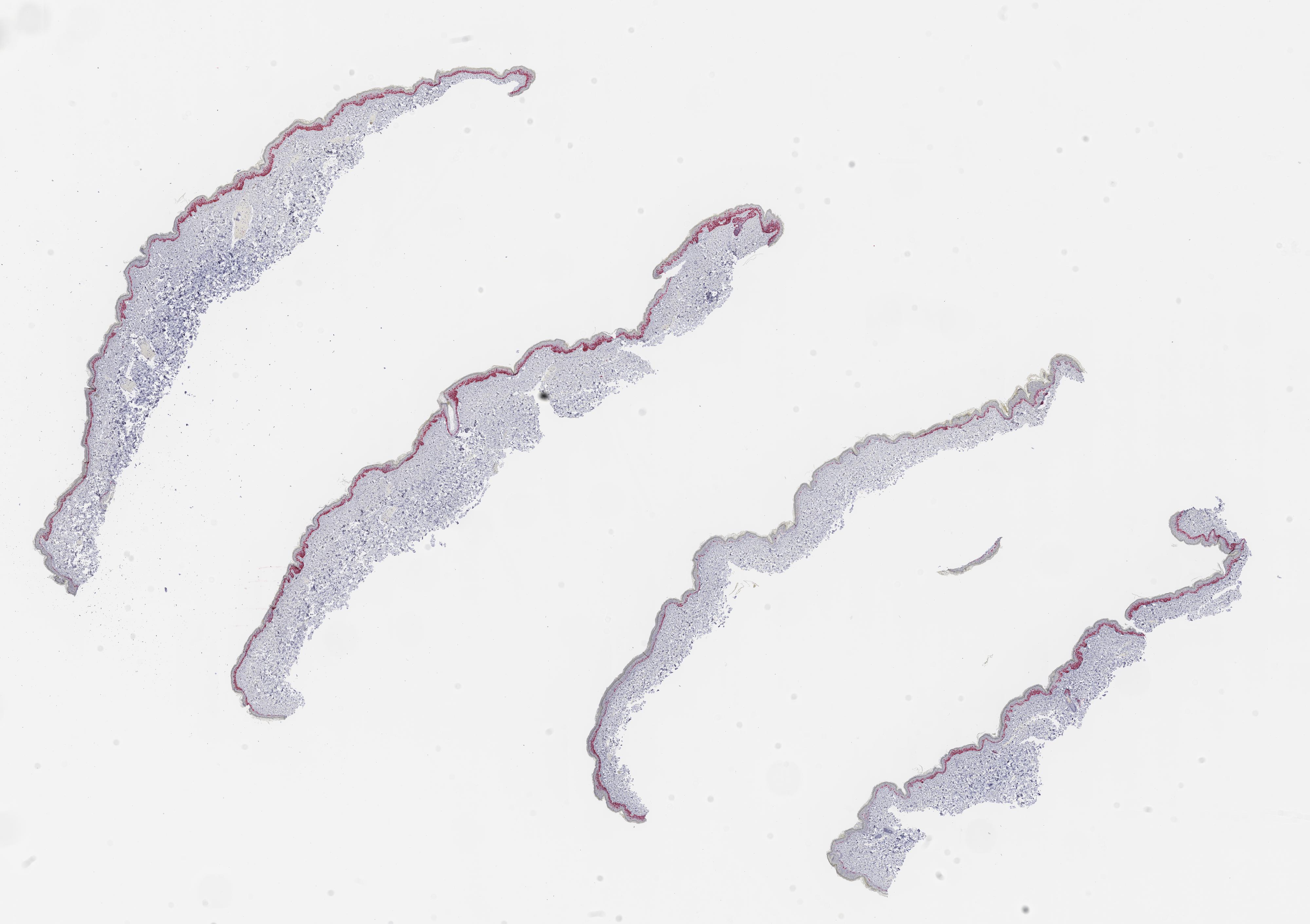

Supplement: Data S1. Illustrative low-resolution summary views of archival H&E-IHC whole slide image pairs, related to STAR Methods and Figure 1 — Details available in Tables S1 and S2. [file mmc2.zip › WSI-08_IHC.jpg]

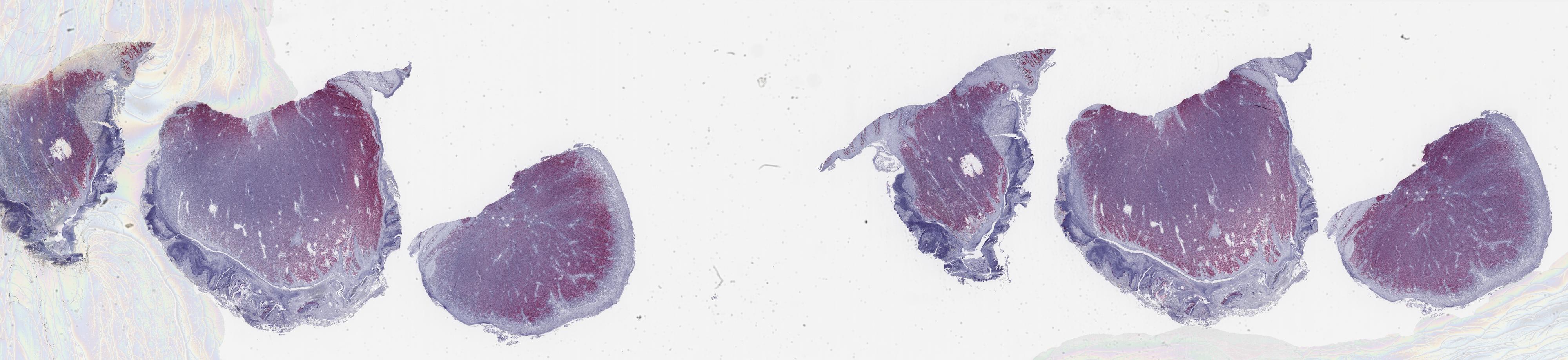

Supplement: Data S1. Illustrative low-resolution summary views of archival H&E-IHC whole slide image pairs, related to STAR Methods and Figure 1 — Details available in Tables S1 and S2. [file mmc2.zip › WSI-01_IHC.jpg]

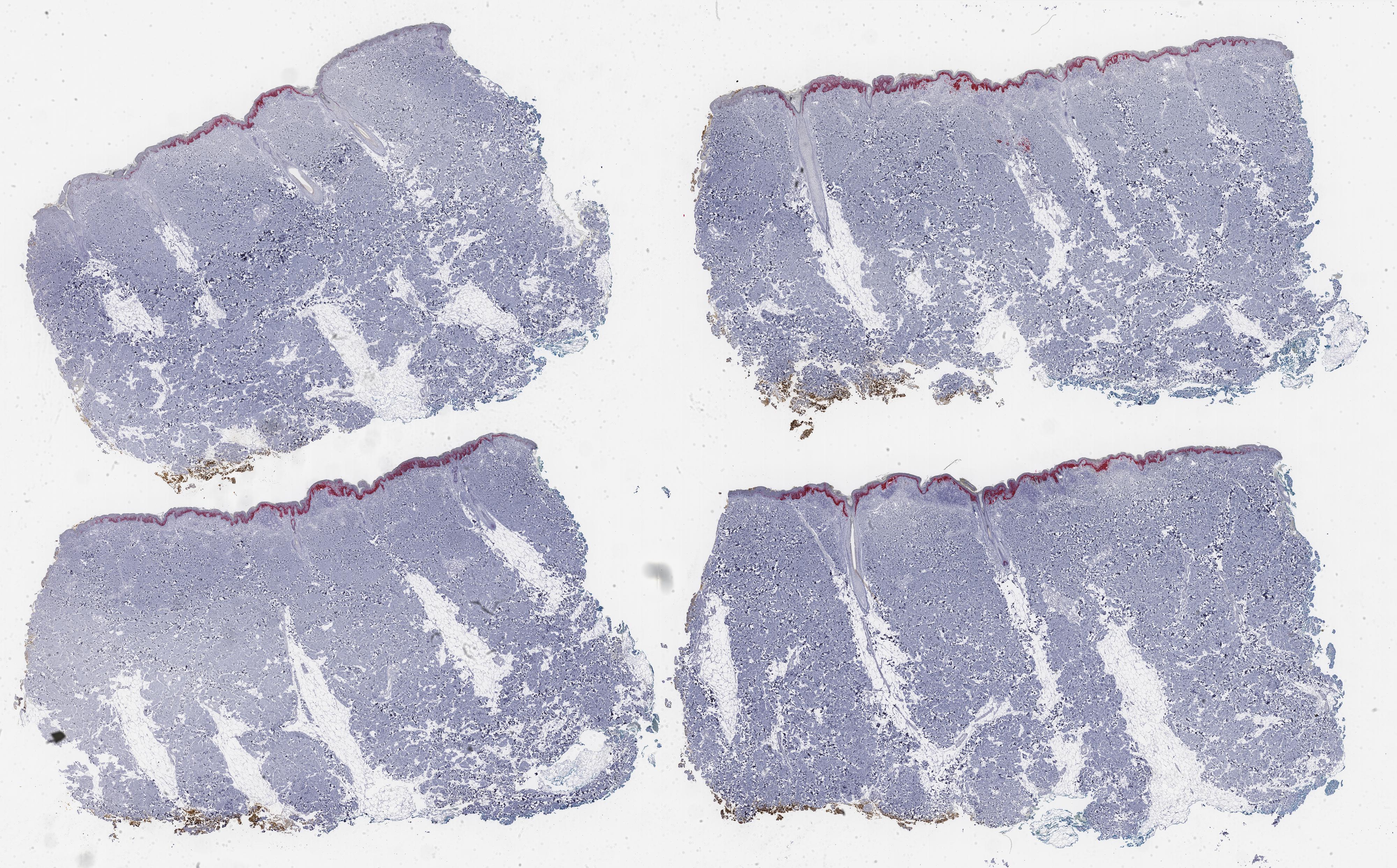

Supplement: Data S1. Illustrative low-resolution summary views of archival H&E-IHC whole slide image pairs, related to STAR Methods and Figure 1 — Details available in Tables S1 and S2. [file mmc2.zip › WSI-11_IHC.jpg]

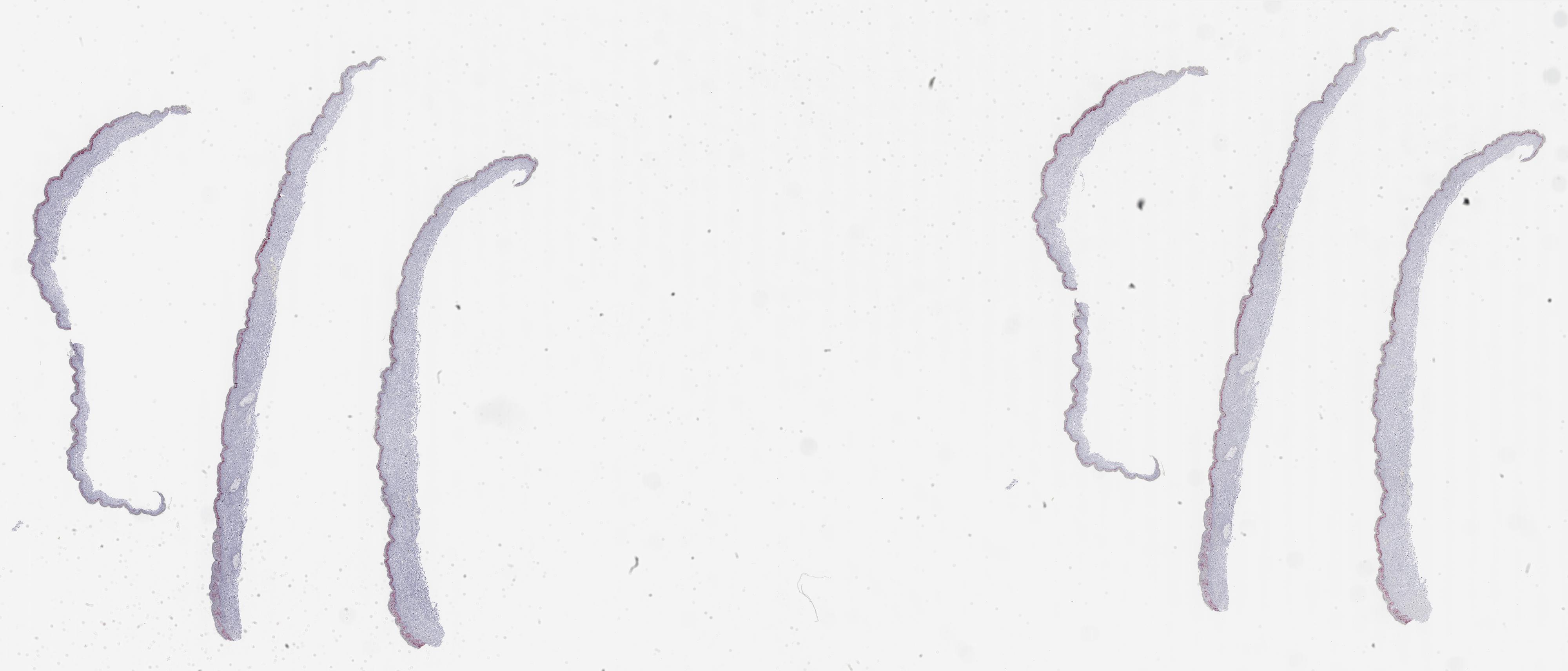

Supplement: Data S1. Illustrative low-resolution summary views of archival H&E-IHC whole slide image pairs, related to STAR Methods and Figure 1 — Details available in Tables S1 and S2. [file mmc2.zip › WSI-14_IHC.jpg]

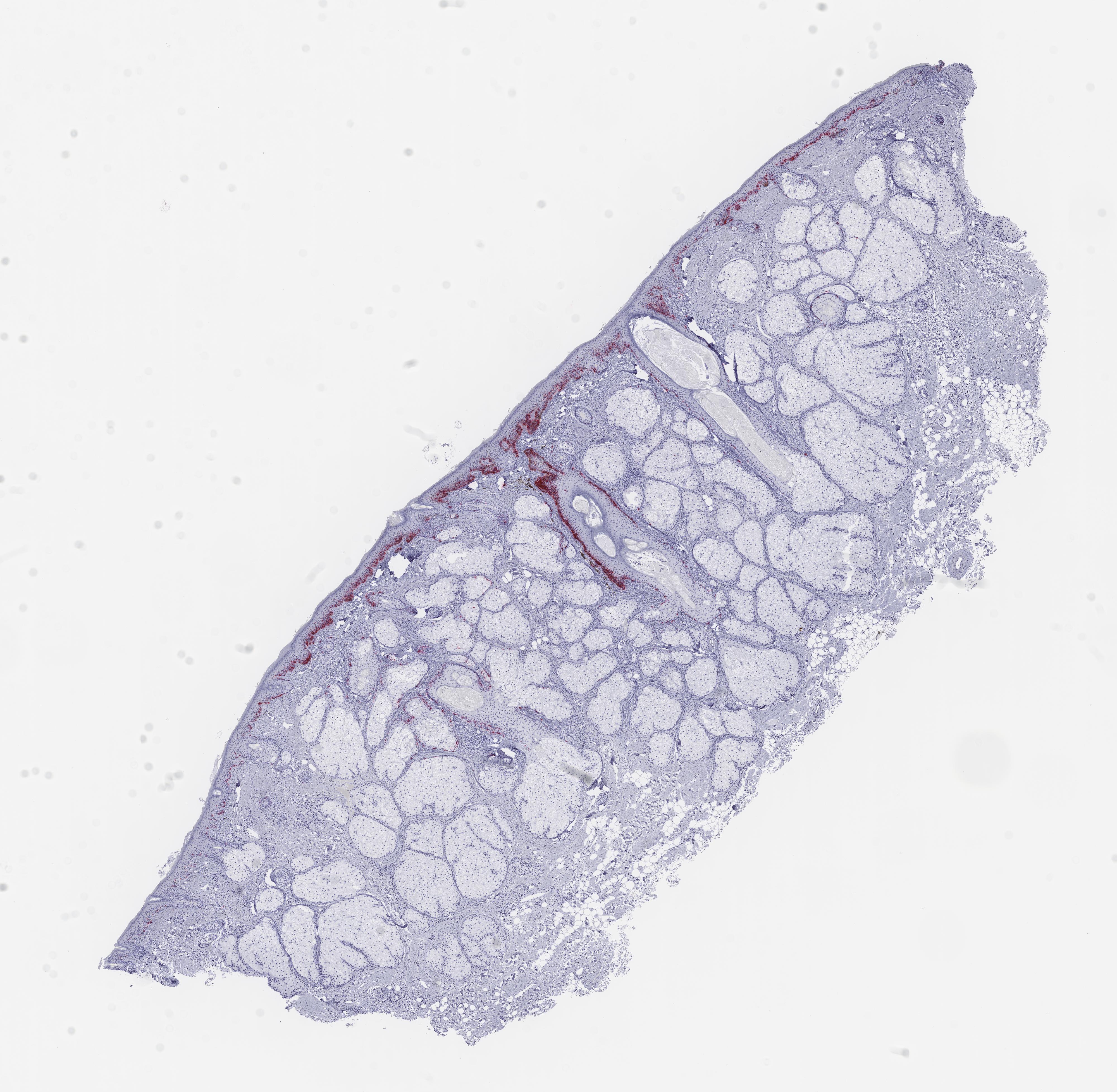

Supplement: Data S1. Illustrative low-resolution summary views of archival H&E-IHC whole slide image pairs, related to STAR Methods and Figure 1 — Details available in Tables S1 and S2. [file mmc2.zip › WSI-04_IHC.jpg]

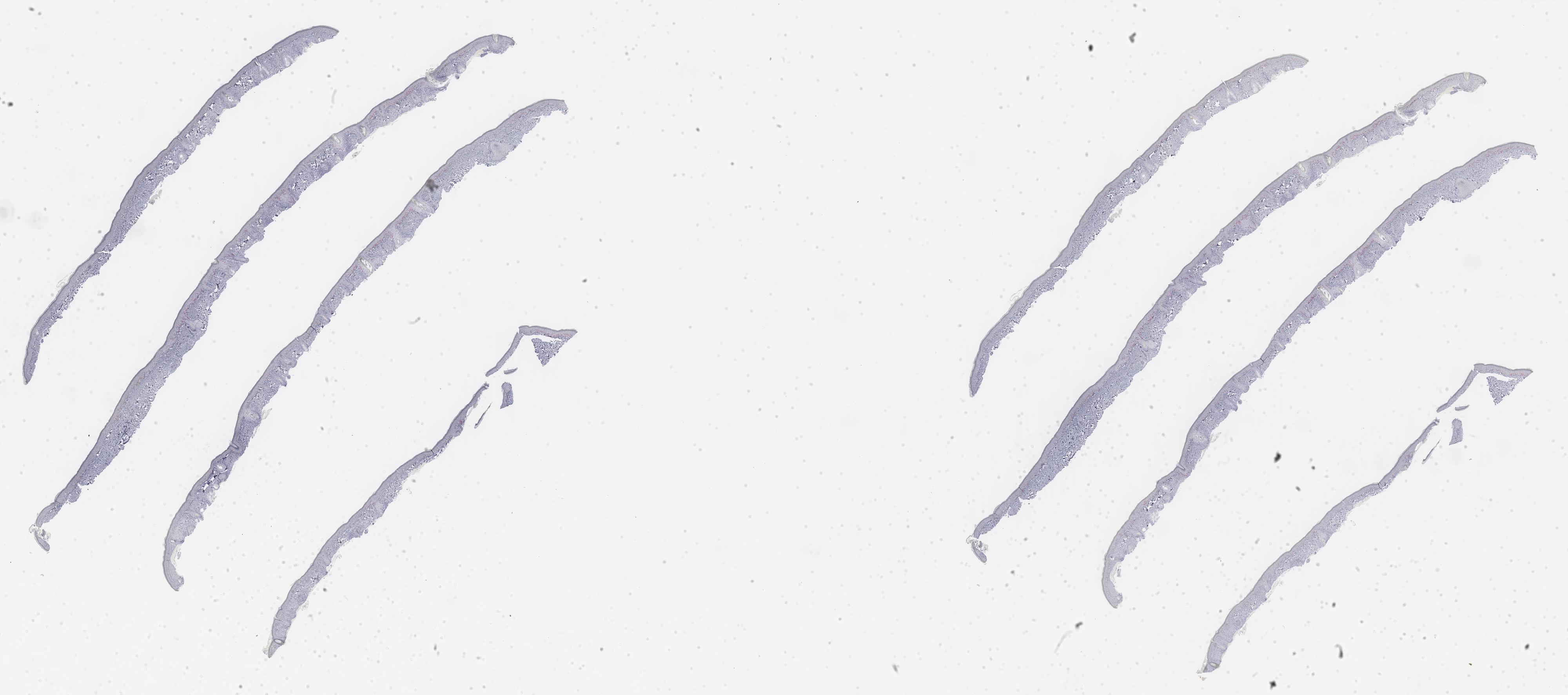

Supplement: Data S1. Illustrative low-resolution summary views of archival H&E-IHC whole slide image pairs, related to STAR Methods and Figure 1 — Details available in Tables S1 and S2. [file mmc2.zip › WSI-49_IHC.jpg]

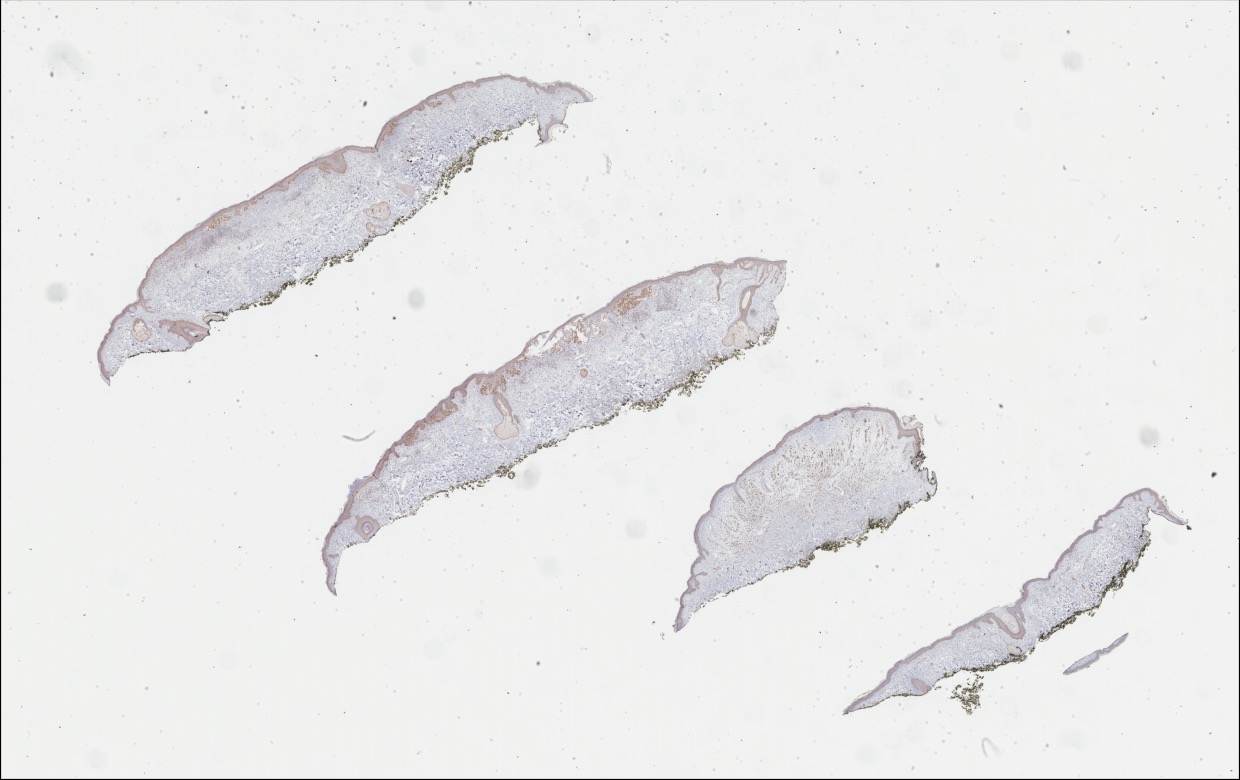

Supplement: Data S1. Illustrative low-resolution summary views of archival H&E-IHC whole slide image pairs, related to STAR Methods and Figure 1 — Details available in Tables S1 and S2. [file mmc2.zip › WSI-59_IHC.jpg]

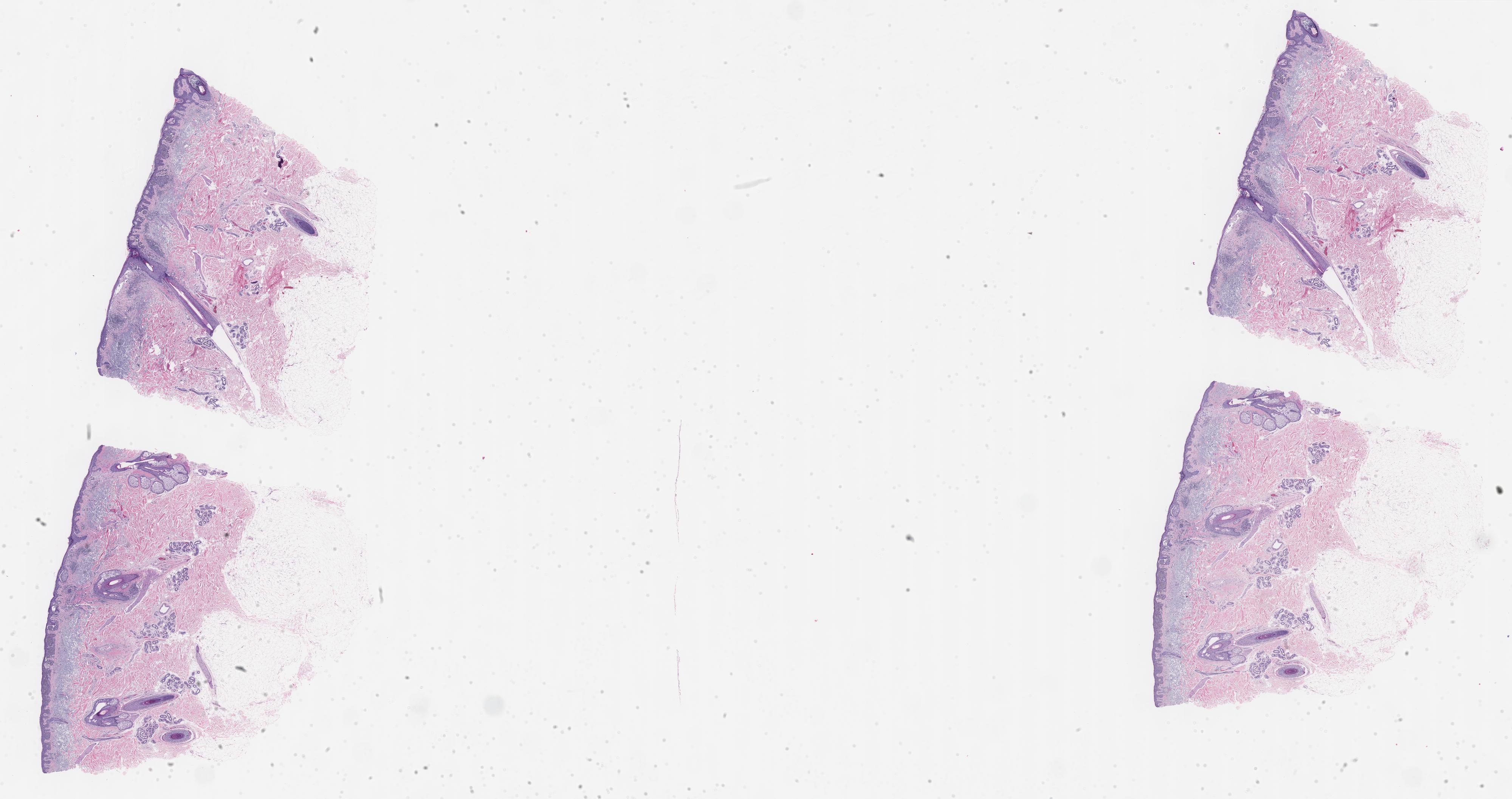

Supplement: Data S1. Illustrative low-resolution summary views of archival H&E-IHC whole slide image pairs, related to STAR Methods and Figure 1 — Details available in Tables S1 and S2. [file mmc2.zip › WSI-12_HE.jpg]

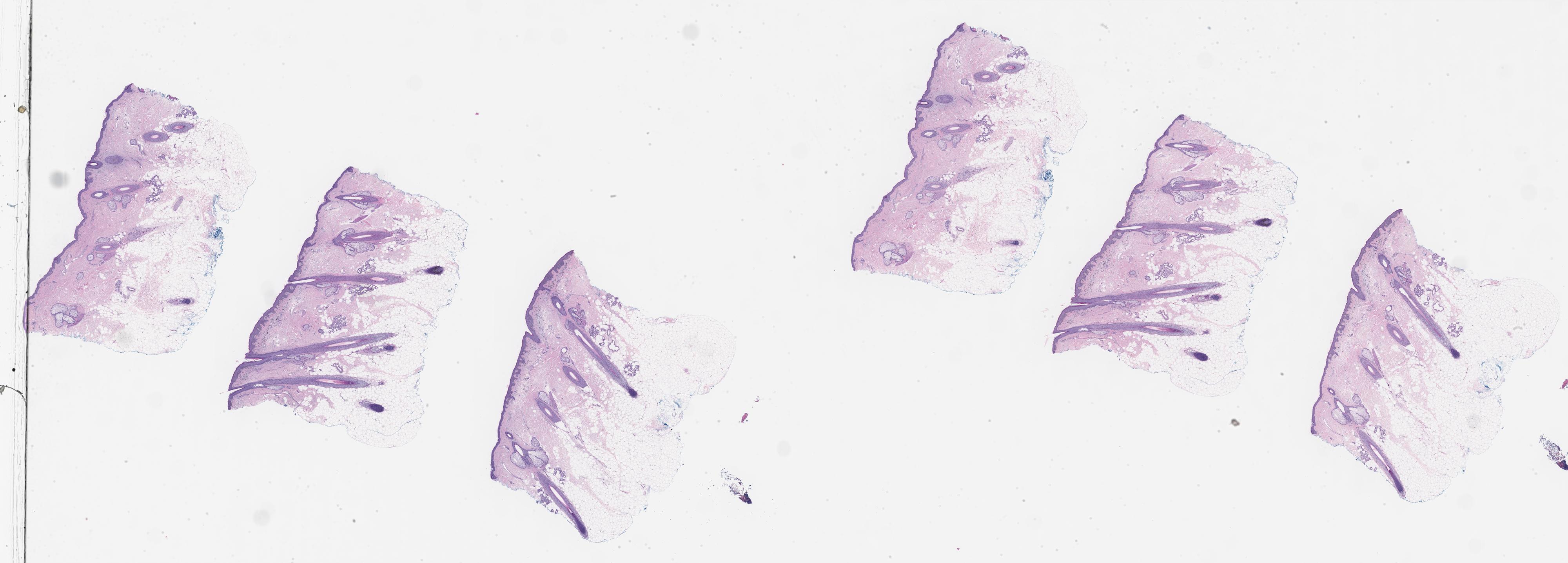

Supplement: Data S1. Illustrative low-resolution summary views of archival H&E-IHC whole slide image pairs, related to STAR Methods and Figure 1 — Details available in Tables S1 and S2. [file mmc2.zip › WSI-47_HE.jpg]

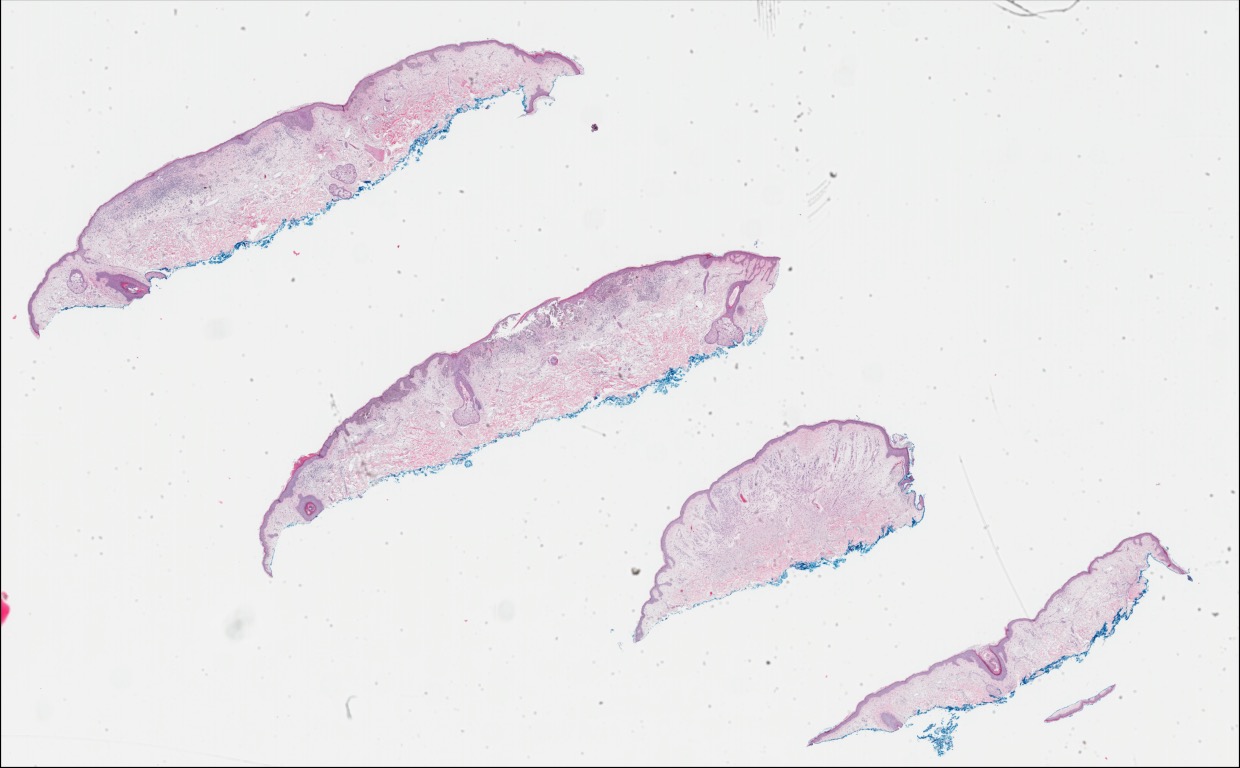

Supplement: Data S1. Illustrative low-resolution summary views of archival H&E-IHC whole slide image pairs, related to STAR Methods and Figure 1 — Details available in Tables S1 and S2. [file mmc2.zip › WSI-59_HE.jpg]

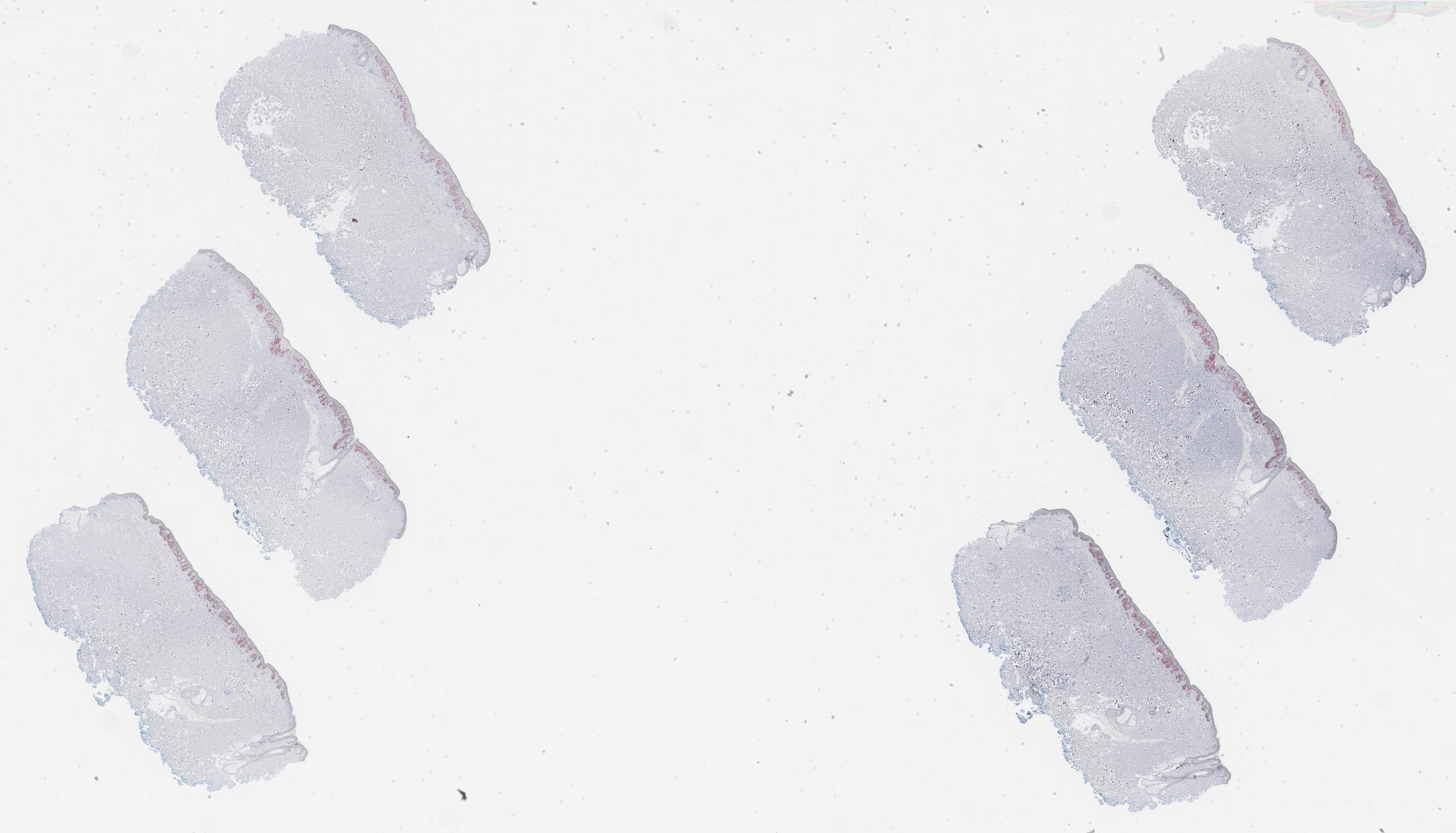

Supplement: Data S1. Illustrative low-resolution summary views of archival H&E-IHC whole slide image pairs, related to STAR Methods and Figure 1 — Details available in Tables S1 and S2. [file mmc2.zip › WSI-50_IHC.jpg]

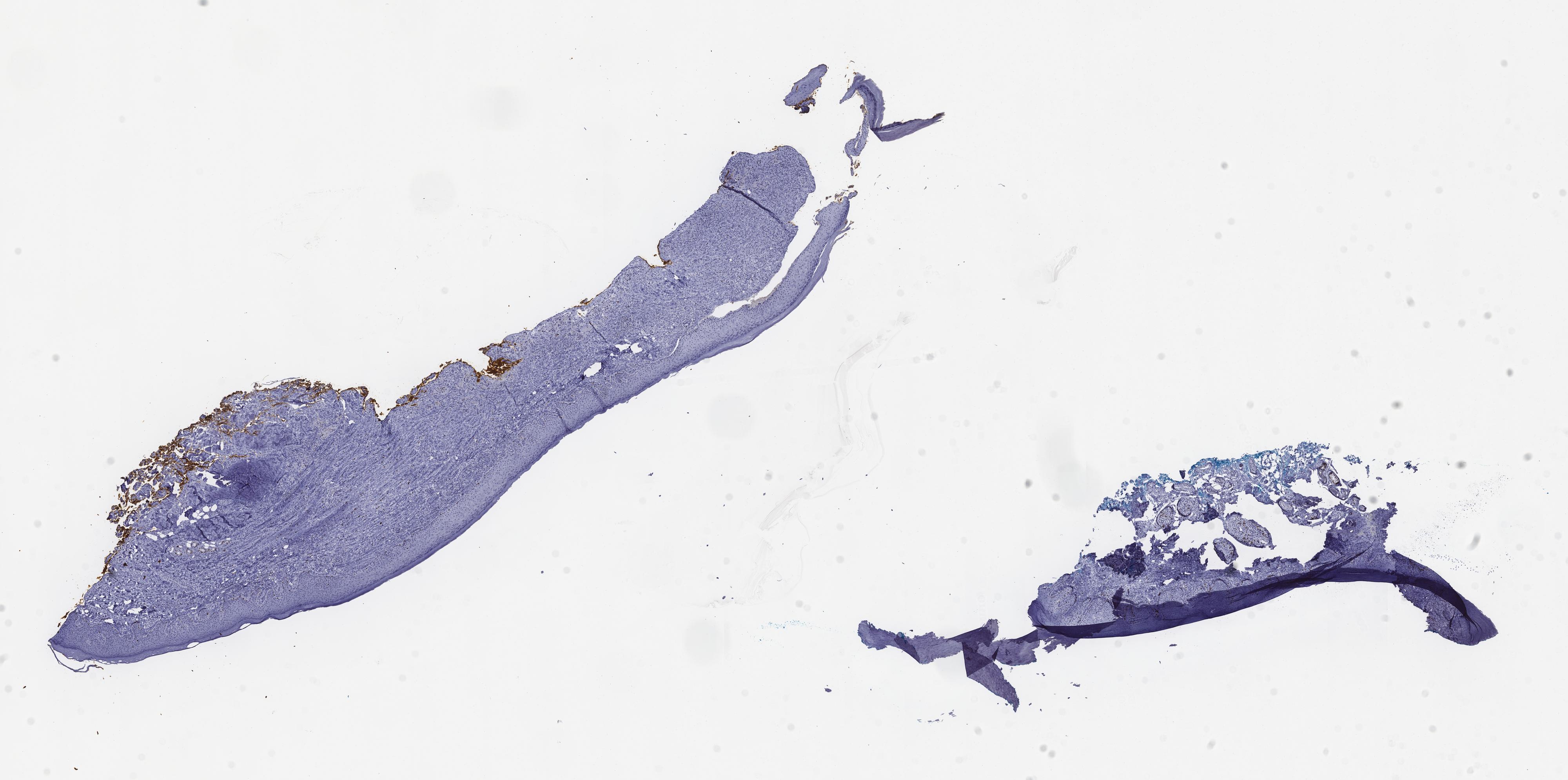

Supplement: Data S1. Illustrative low-resolution summary views of archival H&E-IHC whole slide image pairs, related to STAR Methods and Figure 1 — Details available in Tables S1 and S2. [file mmc2.zip › WSI-40_IHC.jpg]

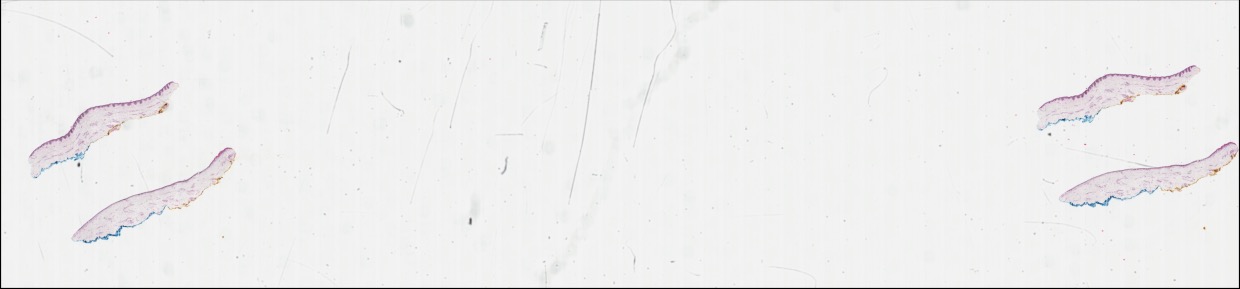

Supplement: Data S1. Illustrative low-resolution summary views of archival H&E-IHC whole slide image pairs, related to STAR Methods and Figure 1 — Details available in Tables S1 and S2. [file mmc2.zip › WSI-24_HE.jpg]

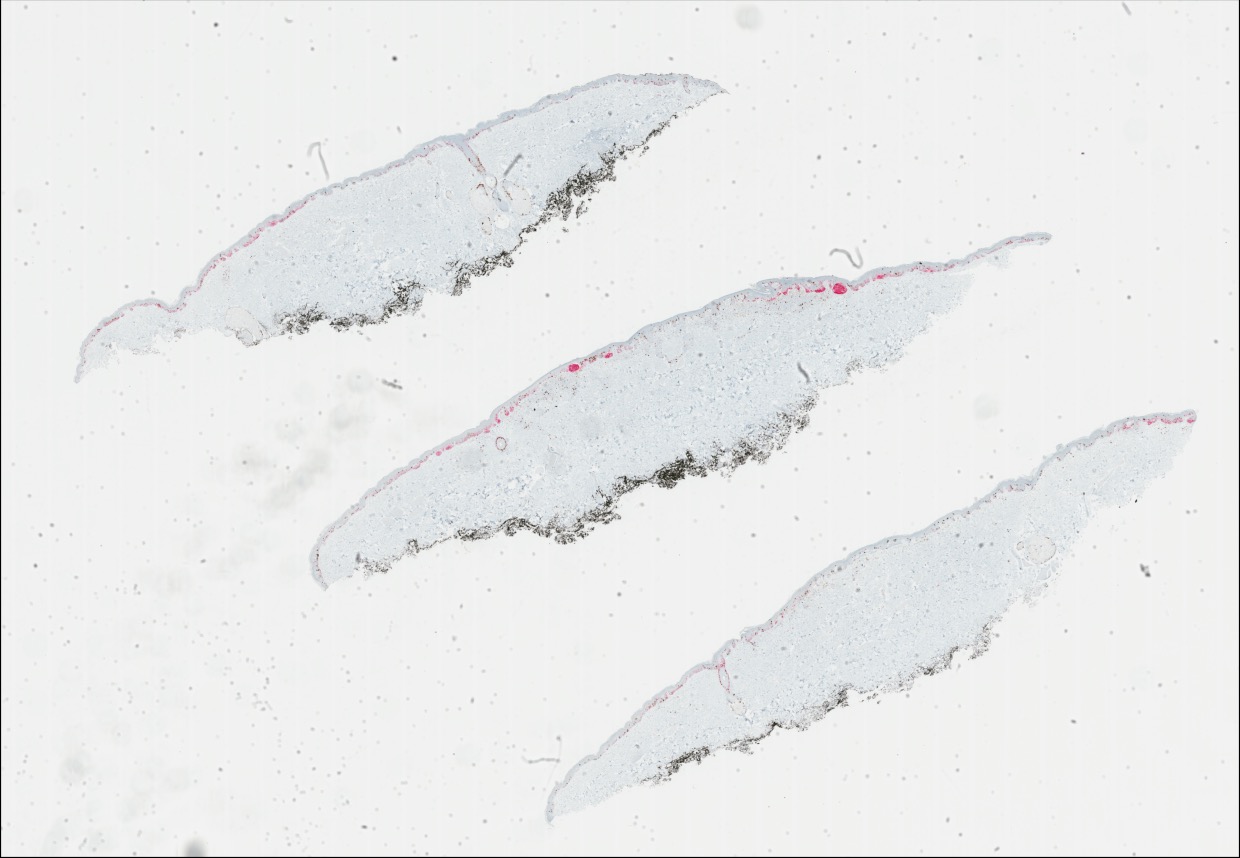

Supplement: Data S1. Illustrative low-resolution summary views of archival H&E-IHC whole slide image pairs, related to STAR Methods and Figure 1 — Details available in Tables S1 and S2. [file mmc2.zip › WSI-32_IHC.jpg]

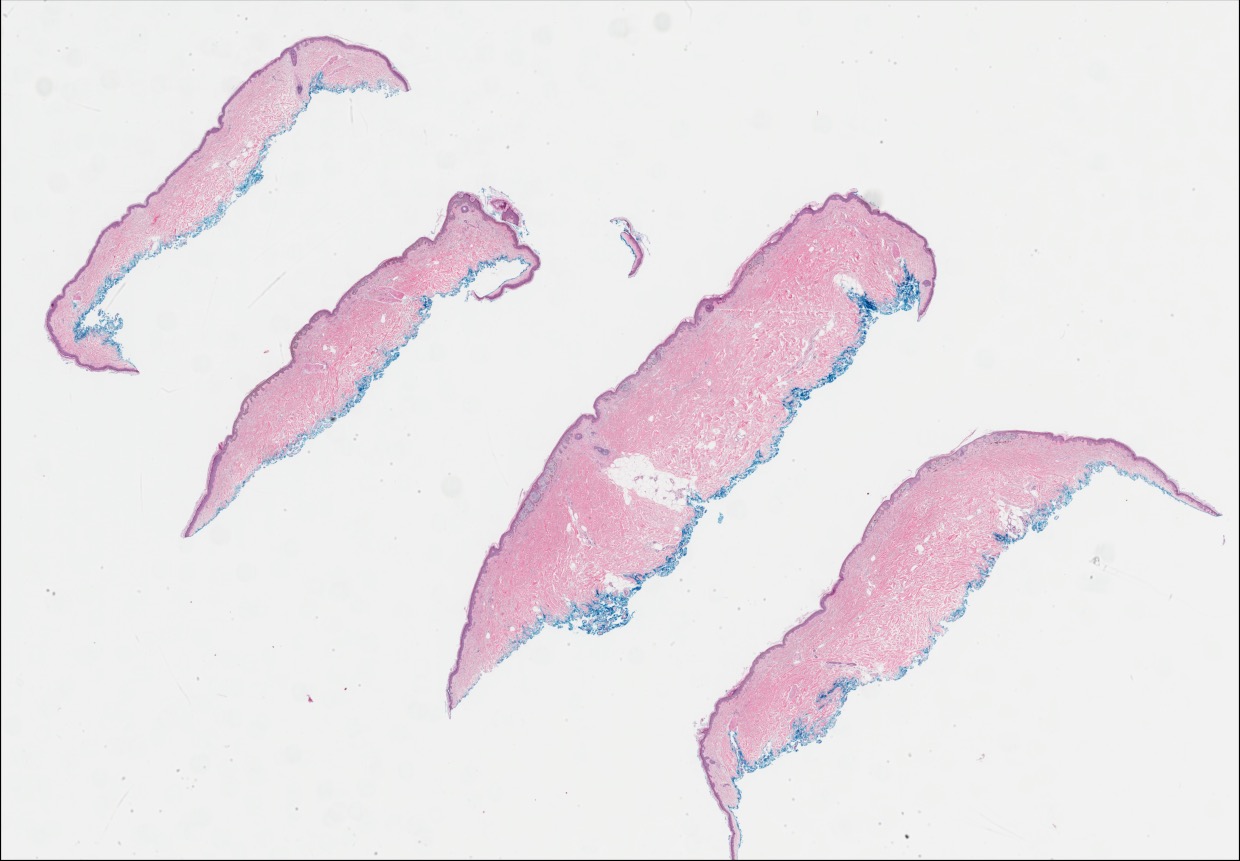

Supplement: Data S1. Illustrative low-resolution summary views of archival H&E-IHC whole slide image pairs, related to STAR Methods and Figure 1 — Details available in Tables S1 and S2. [file mmc2.zip › WSI-55_HE.jpg]

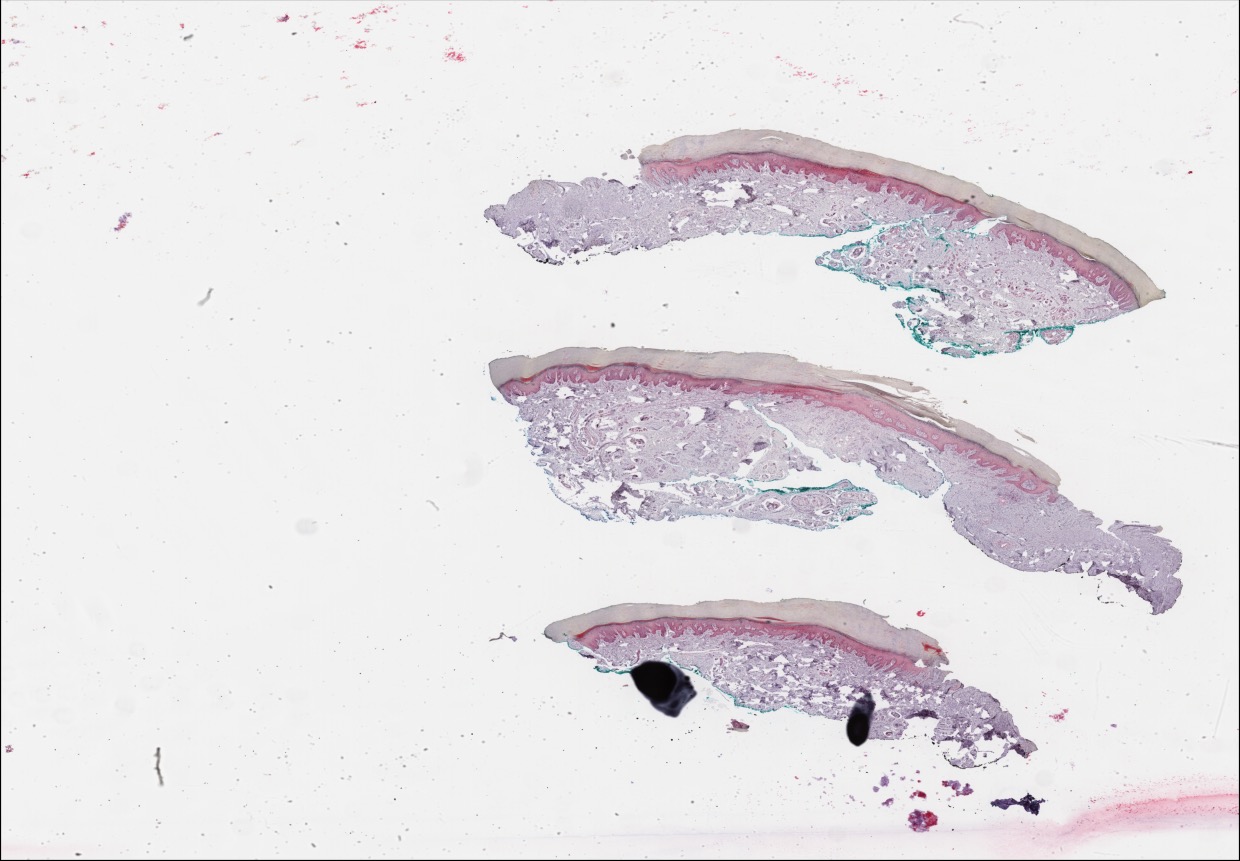

Supplement: Data S1. Illustrative low-resolution summary views of archival H&E-IHC whole slide image pairs, related to STAR Methods and Figure 1 — Details available in Tables S1 and S2. [file mmc2.zip › WSI-22_IHC.jpg]

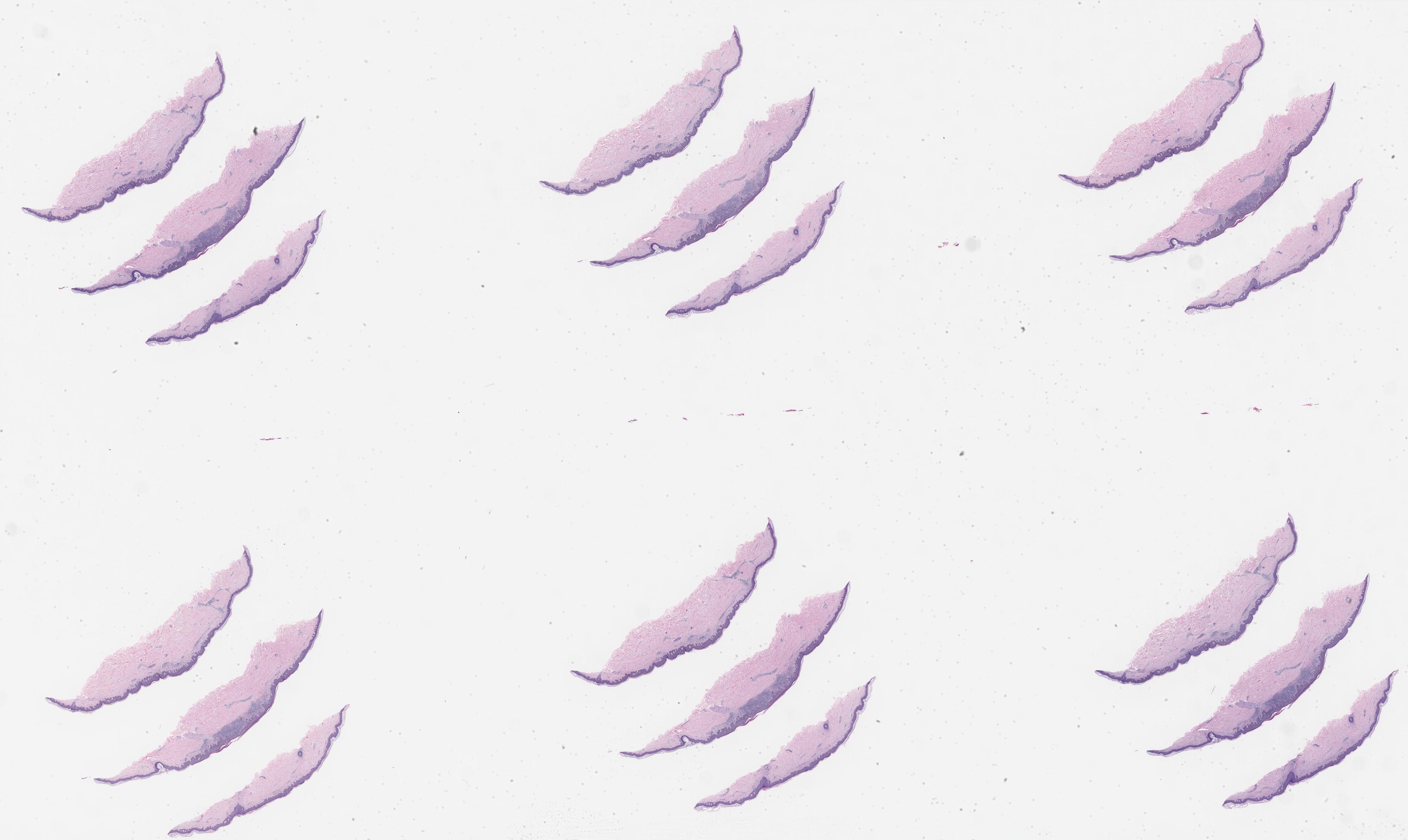

Supplement: Data S1. Illustrative low-resolution summary views of archival H&E-IHC whole slide image pairs, related to STAR Methods and Figure 1 — Details available in Tables S1 and S2. [file mmc2.zip › WSI-28_HE.jpg]

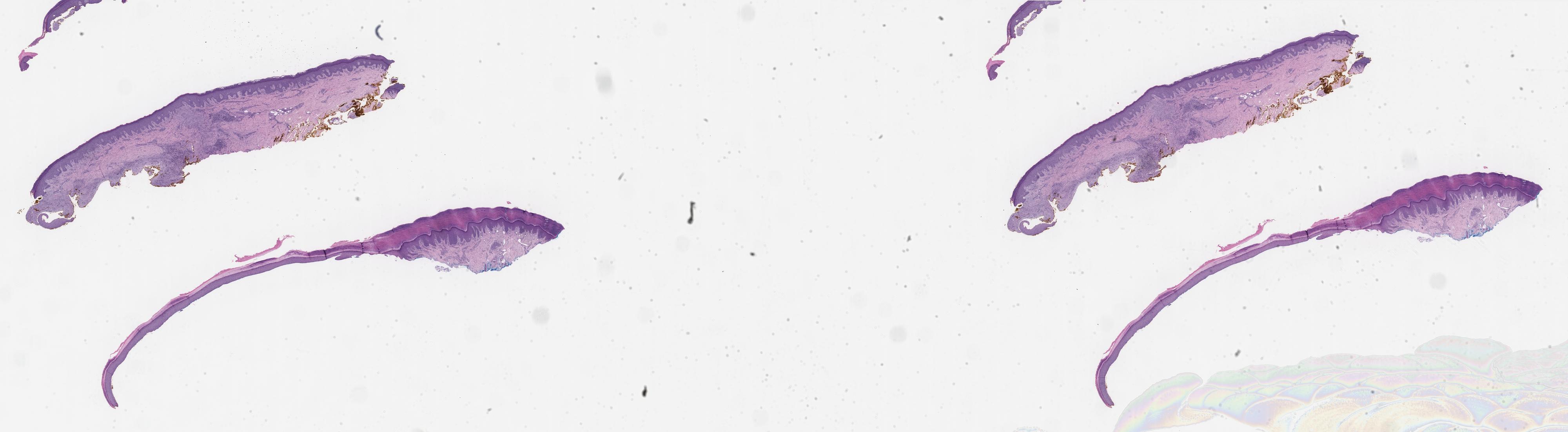

Supplement: Data S1. Illustrative low-resolution summary views of archival H&E-IHC whole slide image pairs, related to STAR Methods and Figure 1 — Details available in Tables S1 and S2. [file mmc2.zip › WSI-36_HE.jpg]

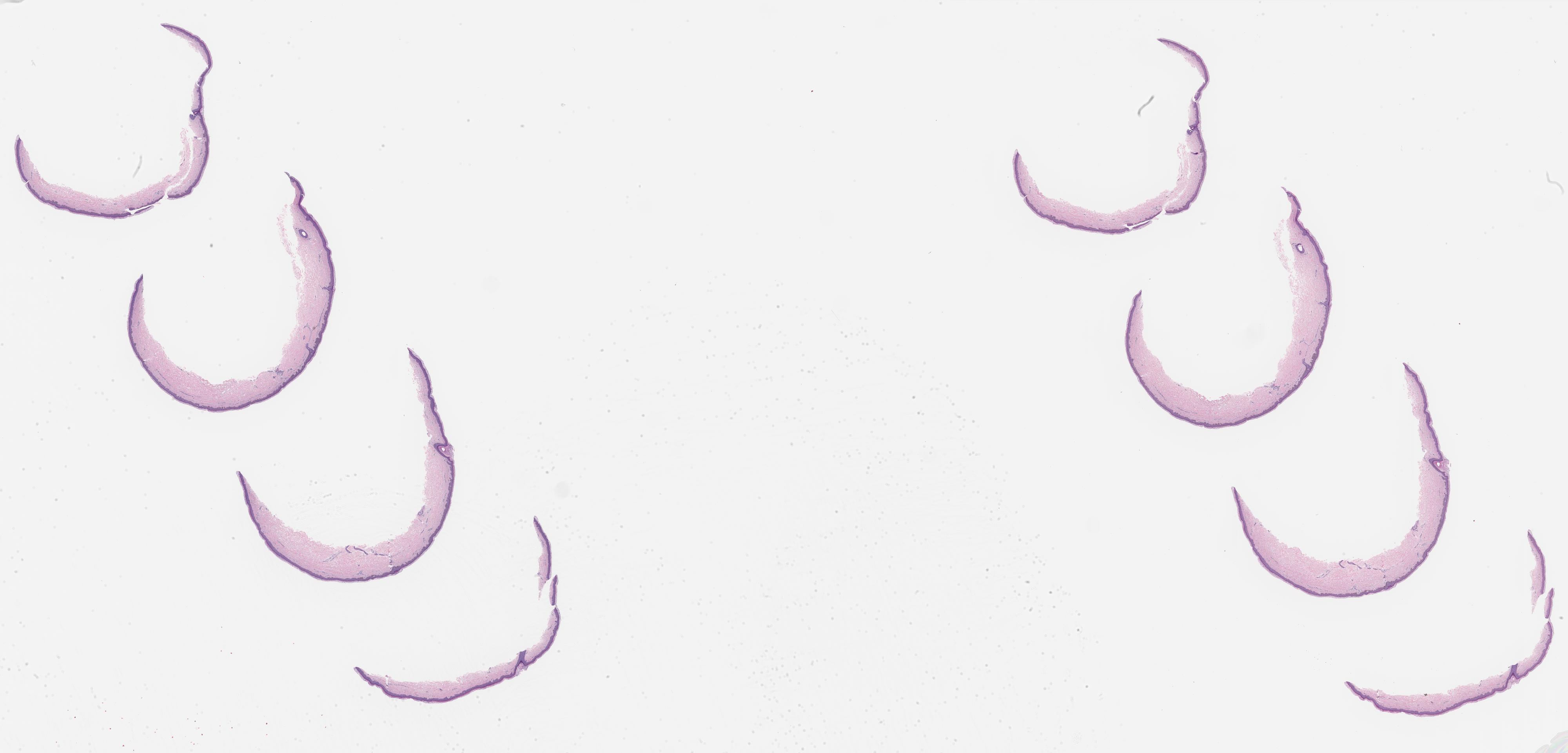

Supplement: Data S1. Illustrative low-resolution summary views of archival H&E-IHC whole slide image pairs, related to STAR Methods and Figure 1 — Details available in Tables S1 and S2. [file mmc2.zip › WSI-10_HE.jpg]

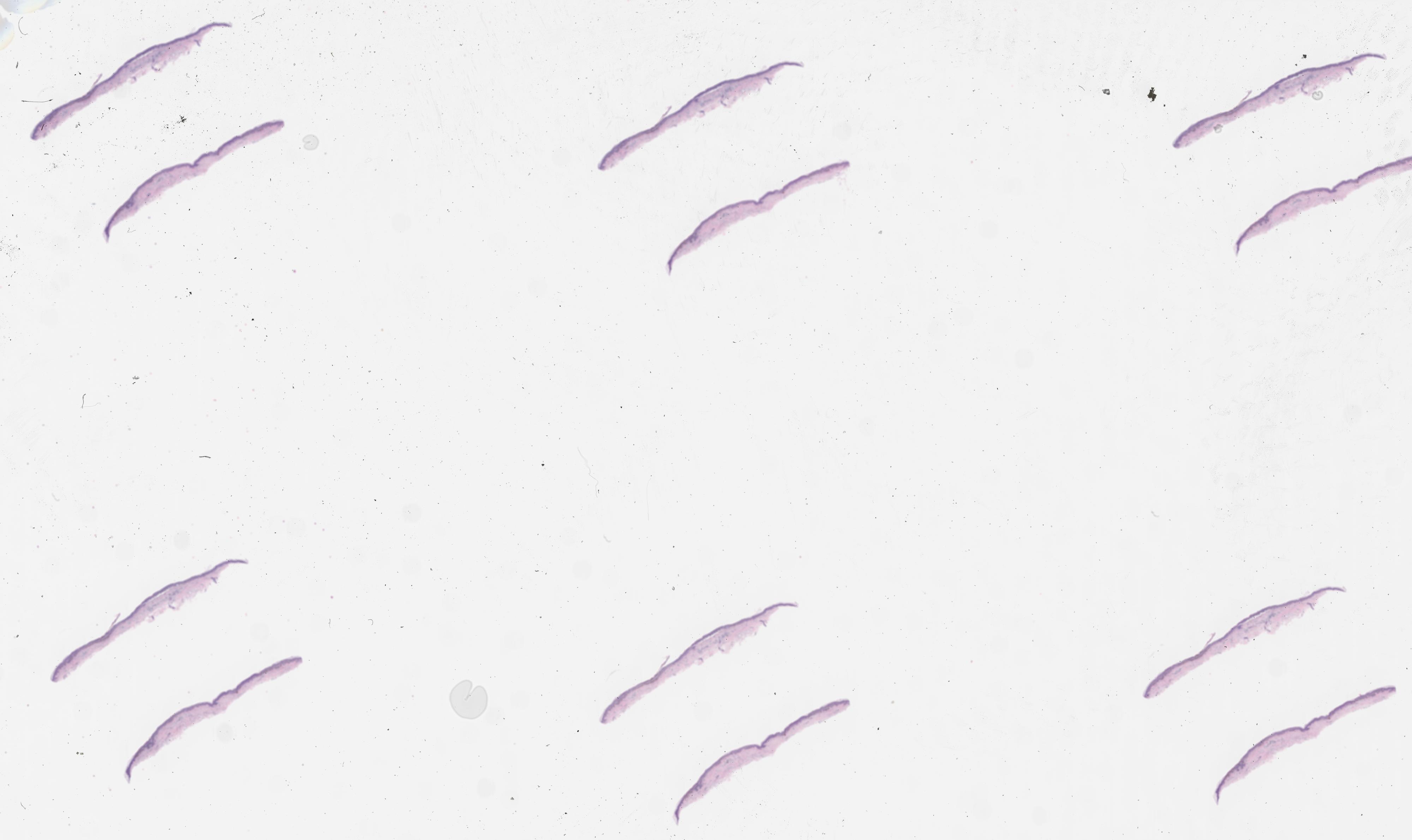

Supplement: Data S1. Illustrative low-resolution summary views of archival H&E-IHC whole slide image pairs, related to STAR Methods and Figure 1 — Details available in Tables S1 and S2. [file mmc2.zip › WSI-27_HEjpg.jpg]

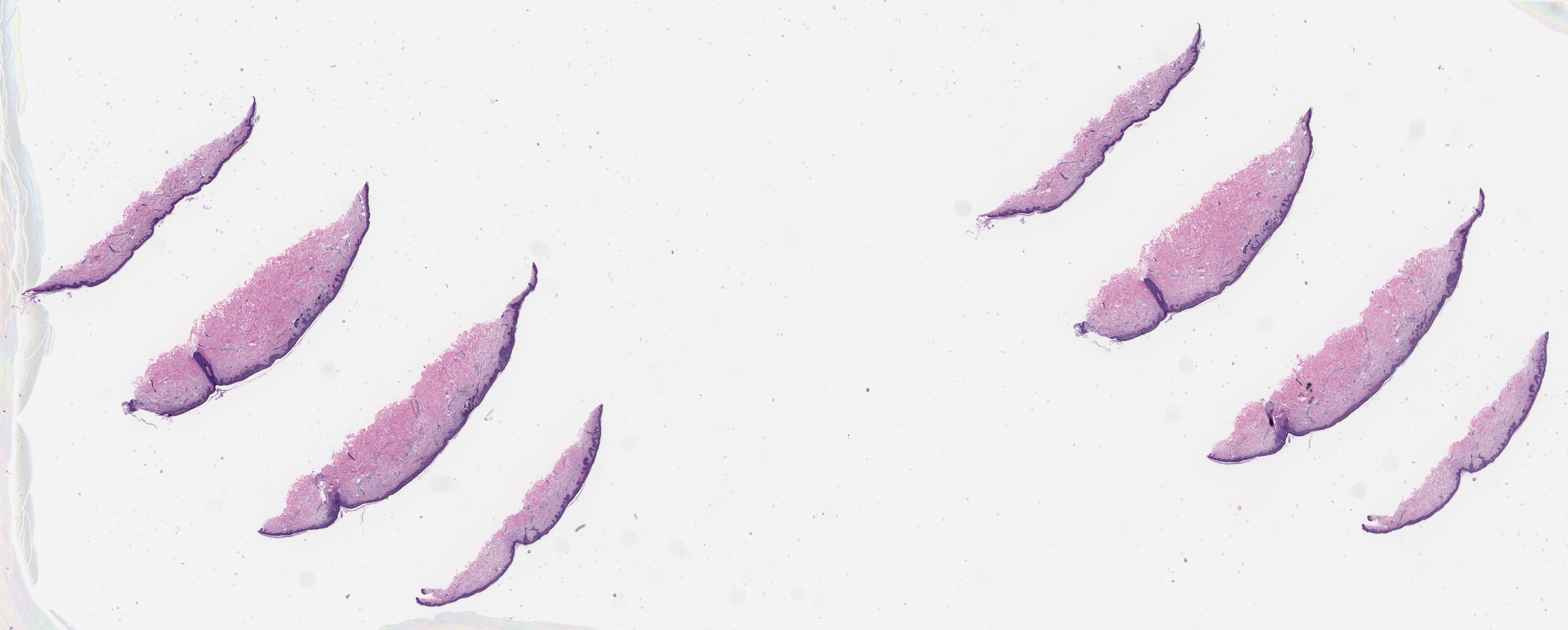

Supplement: Data S1. Illustrative low-resolution summary views of archival H&E-IHC whole slide image pairs, related to STAR Methods and Figure 1 — Details available in Tables S1 and S2. [file mmc2.zip › WSI-02_HE.jpg]

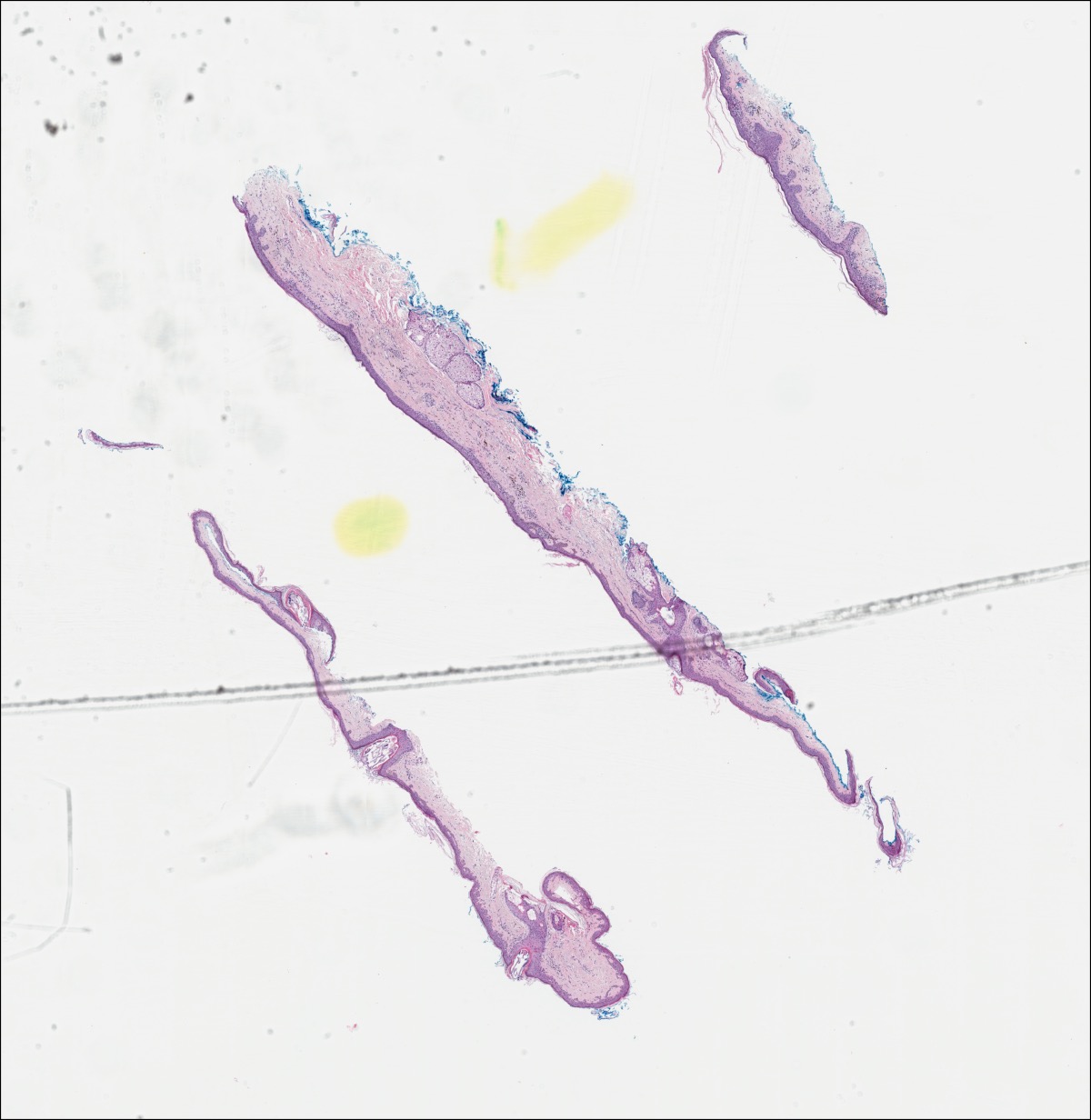

Supplement: Data S1. Illustrative low-resolution summary views of archival H&E-IHC whole slide image pairs, related to STAR Methods and Figure 1 — Details available in Tables S1 and S2. [file mmc2.zip › WSI-61_HE.jpg]

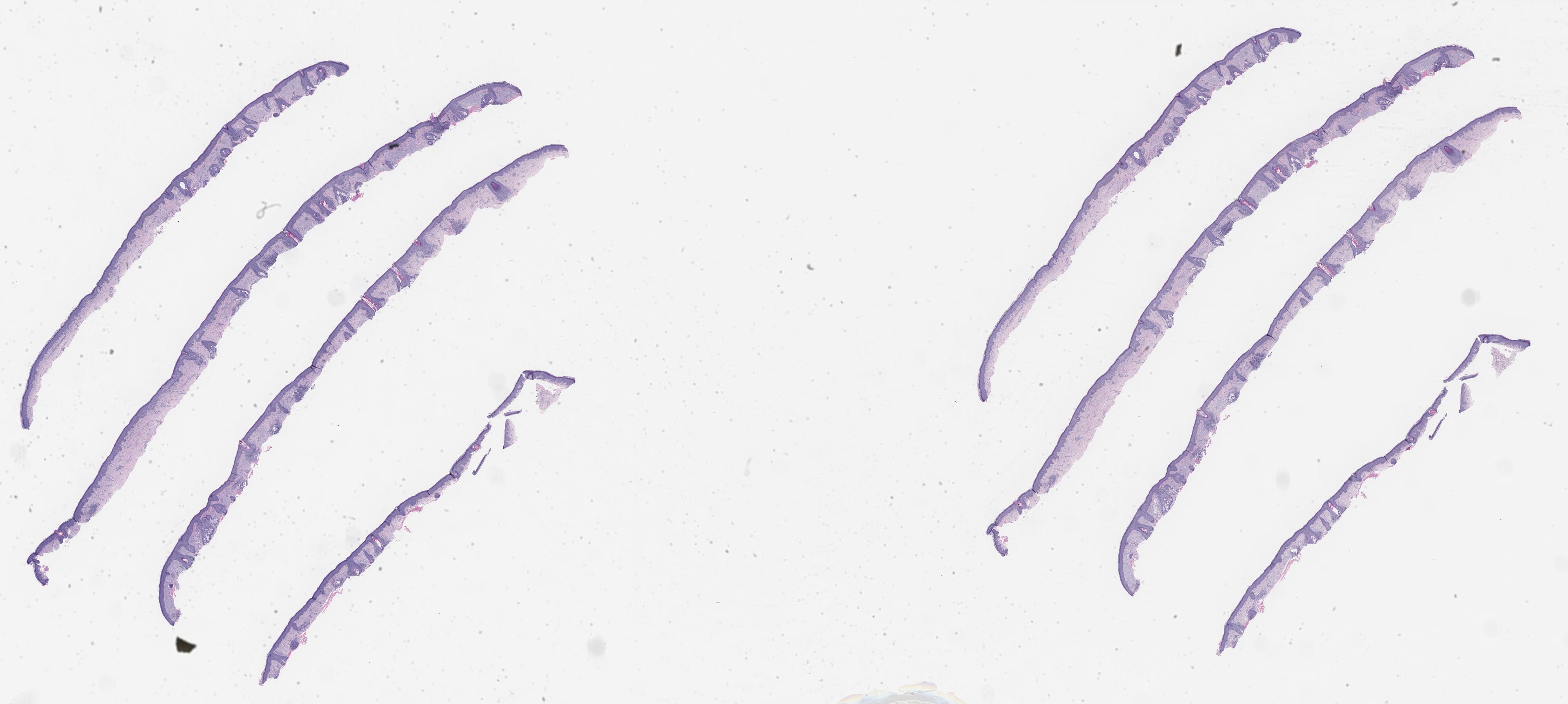

Supplement: Data S1. Illustrative low-resolution summary views of archival H&E-IHC whole slide image pairs, related to STAR Methods and Figure 1 — Details available in Tables S1 and S2. [file mmc2.zip › WSI-49_HE.jpg]

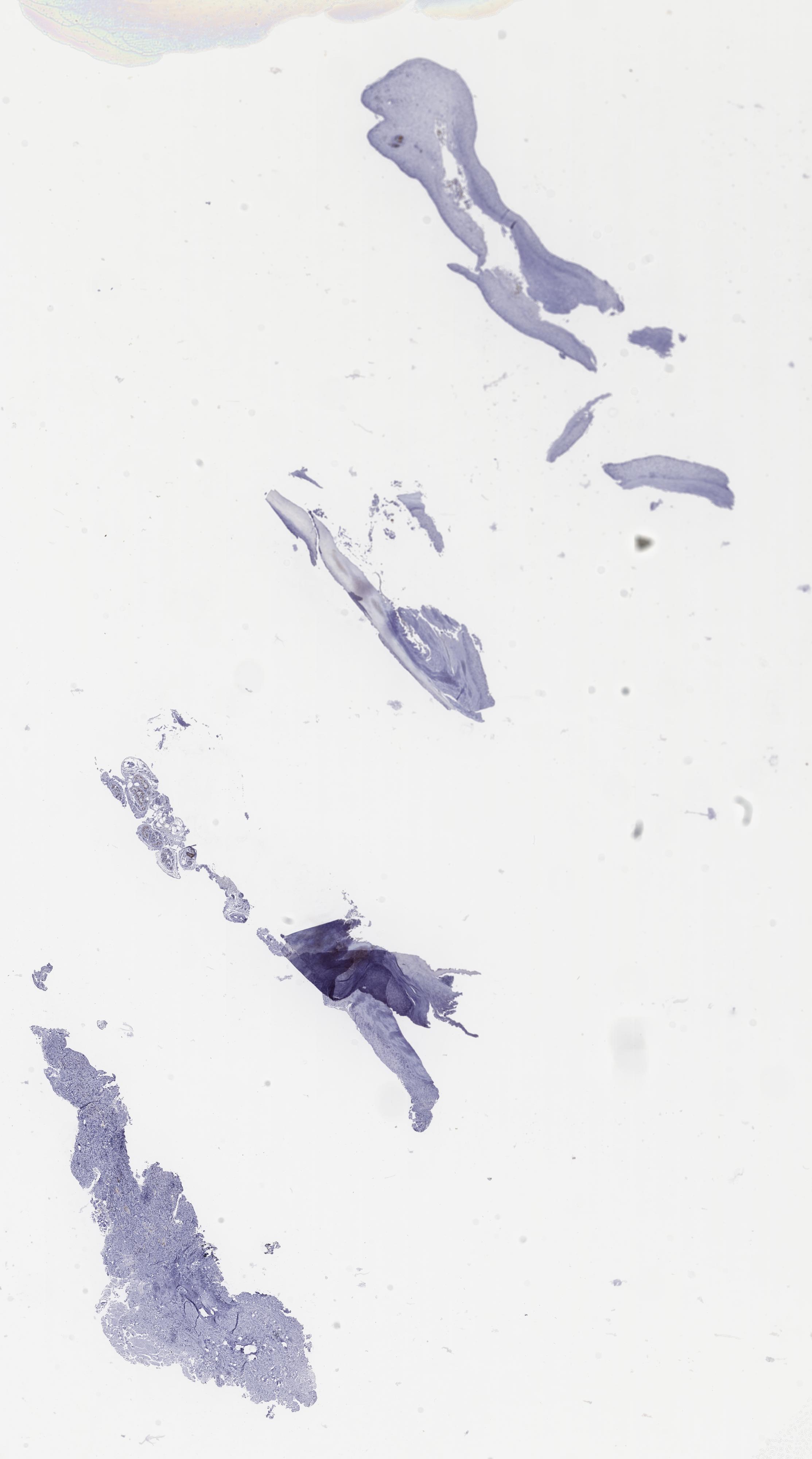

Supplement: Data S1. Illustrative low-resolution summary views of archival H&E-IHC whole slide image pairs, related to STAR Methods and Figure 1 — Details available in Tables S1 and S2. [file mmc2.zip › WSI-41_IHC.jpg]

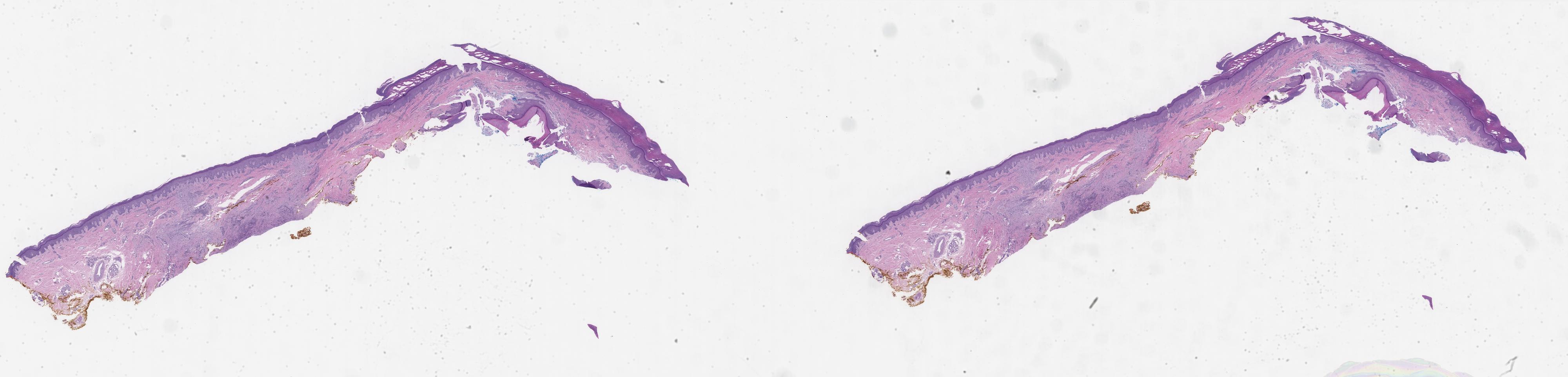

Supplement: Data S1. Illustrative low-resolution summary views of archival H&E-IHC whole slide image pairs, related to STAR Methods and Figure 1 — Details available in Tables S1 and S2. [file mmc2.zip › WSI-34_HE.jpg]

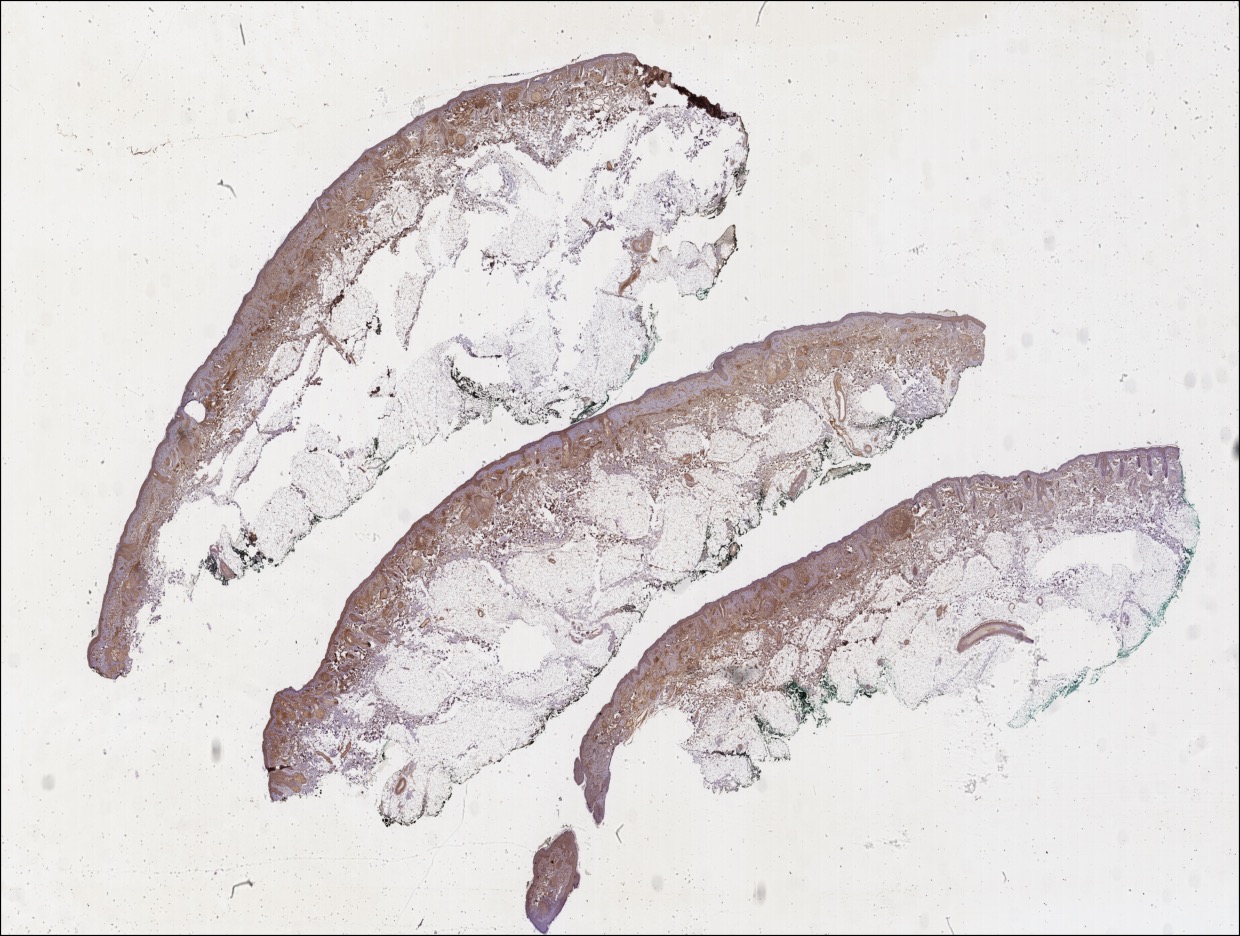

Supplement: Data S1. Illustrative low-resolution summary views of archival H&E-IHC whole slide image pairs, related to STAR Methods and Figure 1 — Details available in Tables S1 and S2. [file mmc2.zip › WSI-51_IHC.jpg]

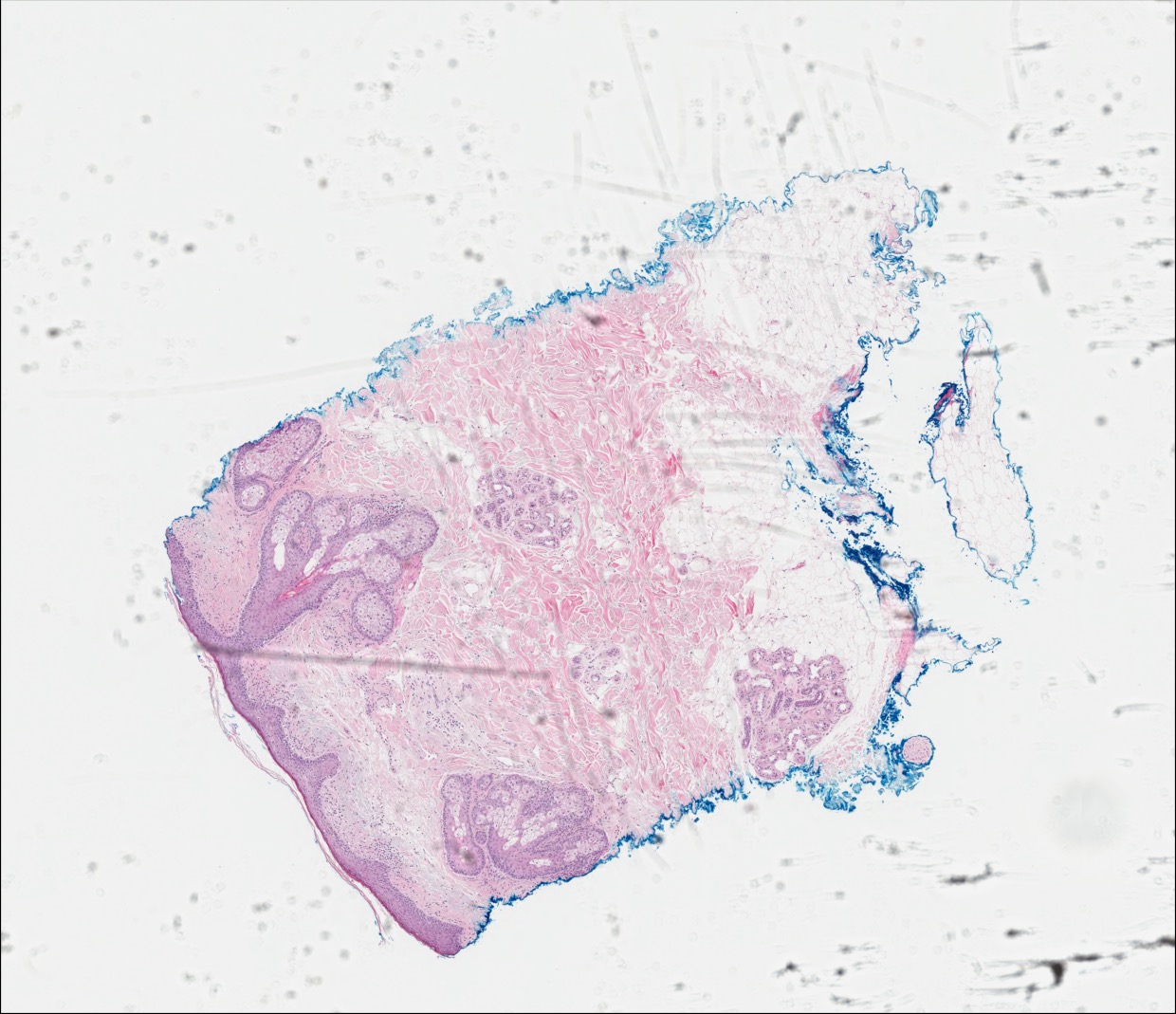

Supplement: Data S1. Illustrative low-resolution summary views of archival H&E-IHC whole slide image pairs, related to STAR Methods and Figure 1 — Details available in Tables S1 and S2. [file mmc2.zip › WSI-57_HE.jpg]

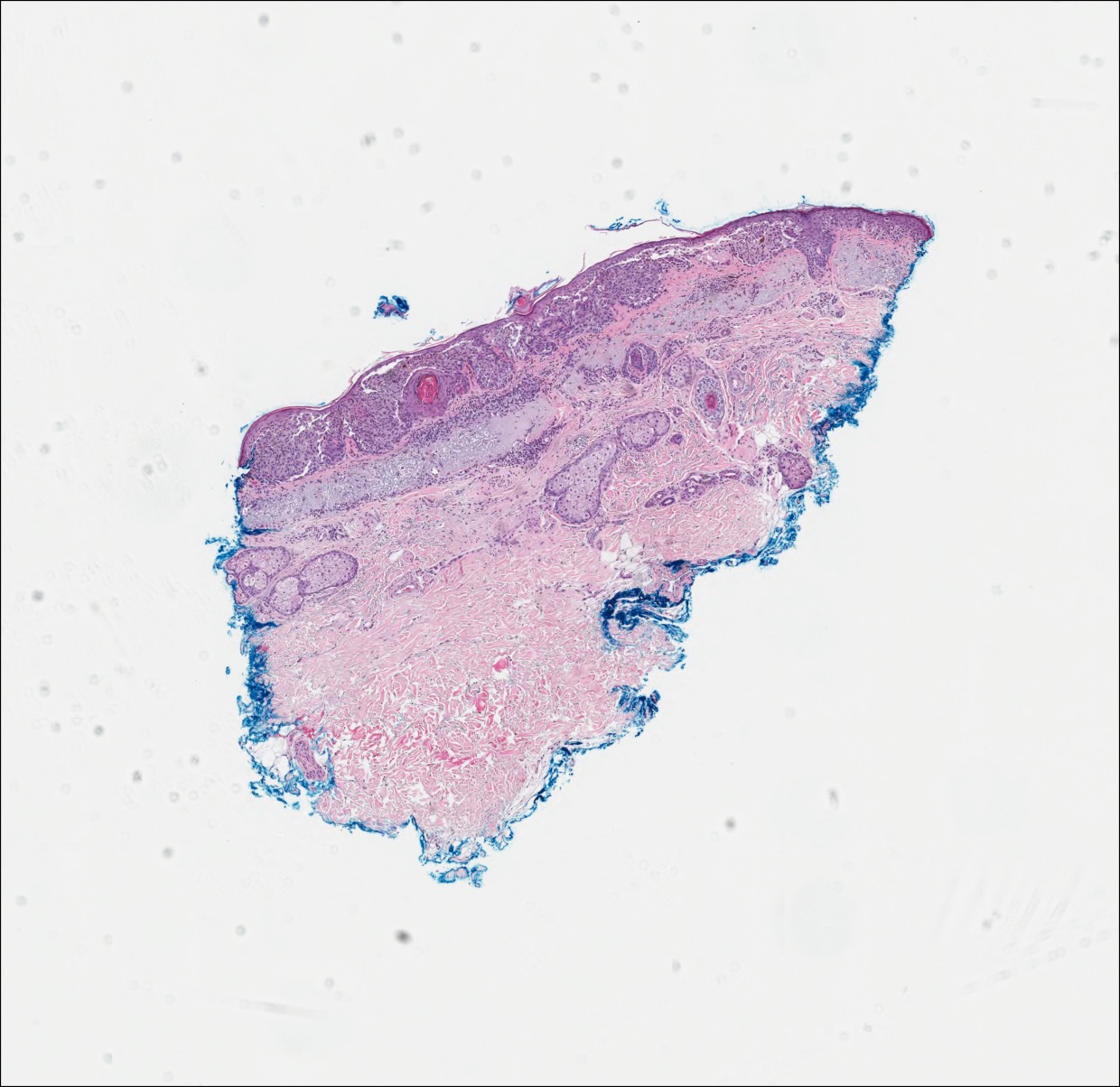

Supplement: Data S1. Illustrative low-resolution summary views of archival H&E-IHC whole slide image pairs, related to STAR Methods and Figure 1 — Details available in Tables S1 and S2. [file mmc2.zip › WSI-26_HE.jpg]

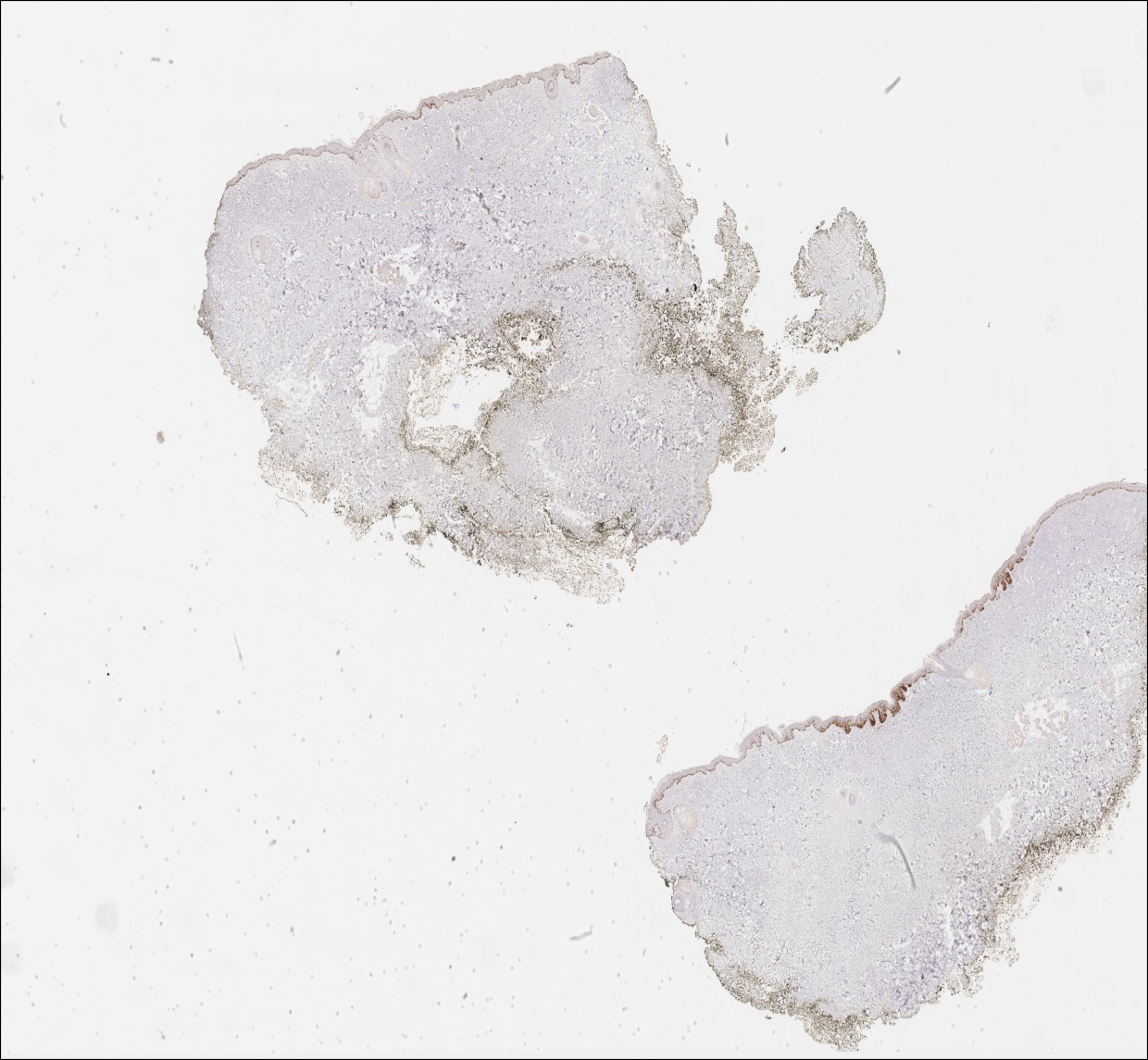

Supplement: Data S1. Illustrative low-resolution summary views of archival H&E-IHC whole slide image pairs, related to STAR Methods and Figure 1 — Details available in Tables S1 and S2. [file mmc2.zip › WSI-23_IHC.jpg]

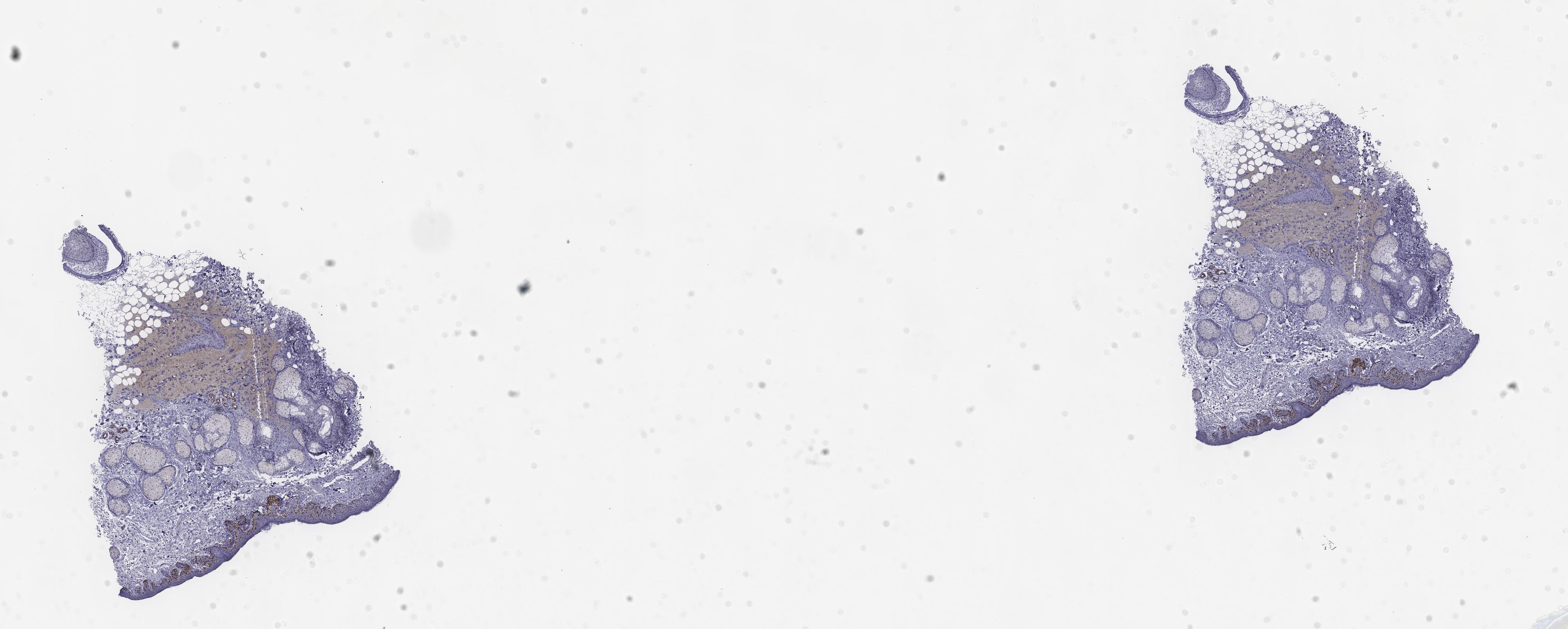

Supplement: Data S1. Illustrative low-resolution summary views of archival H&E-IHC whole slide image pairs, related to STAR Methods and Figure 1 — Details available in Tables S1 and S2. [file mmc2.zip › WSI-33_IHC.jpg]

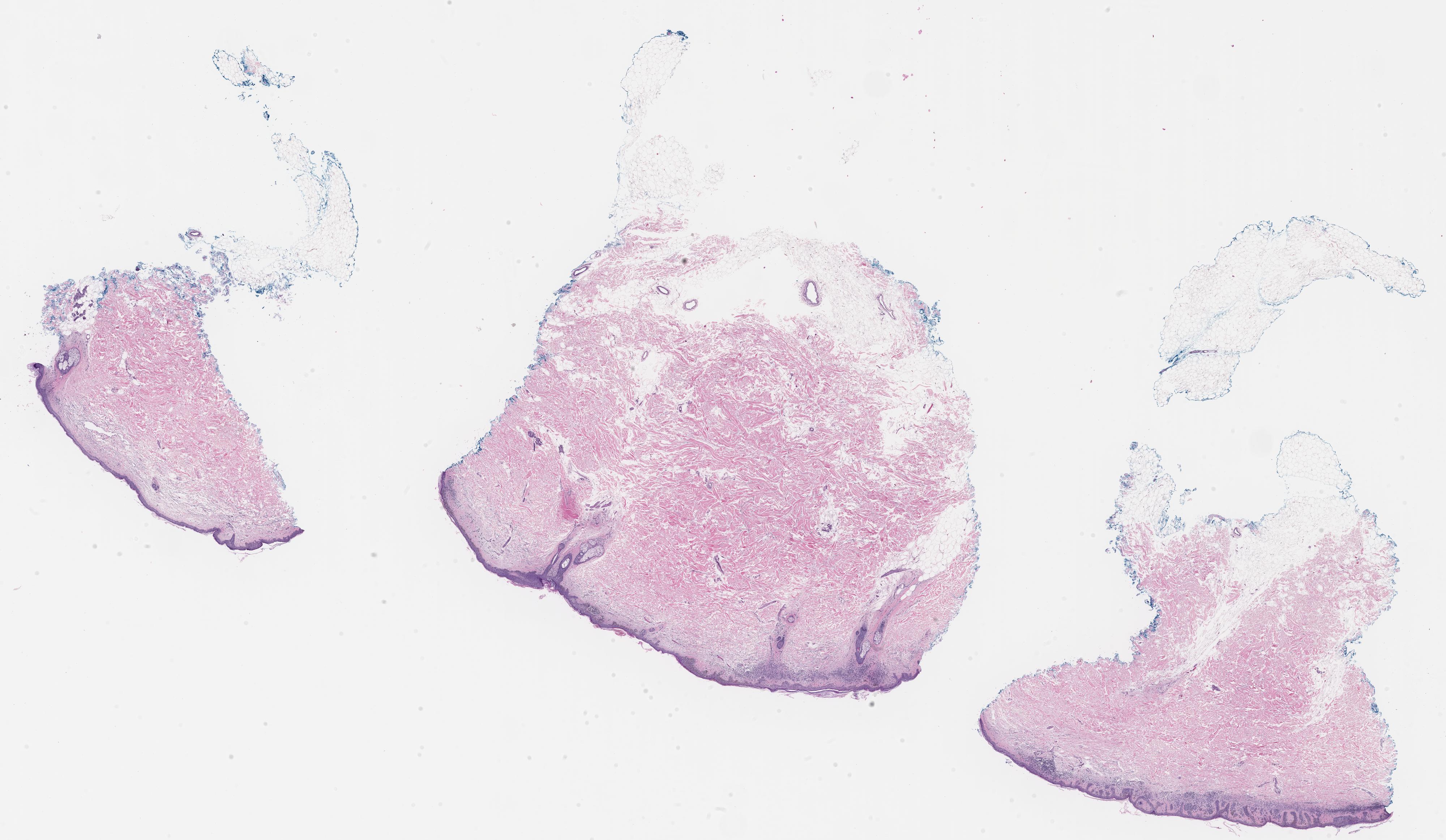

Supplement: Data S1. Illustrative low-resolution summary views of archival H&E-IHC whole slide image pairs, related to STAR Methods and Figure 1 — Details available in Tables S1 and S2. [file mmc2.zip › WSI-45_HE.jpg]

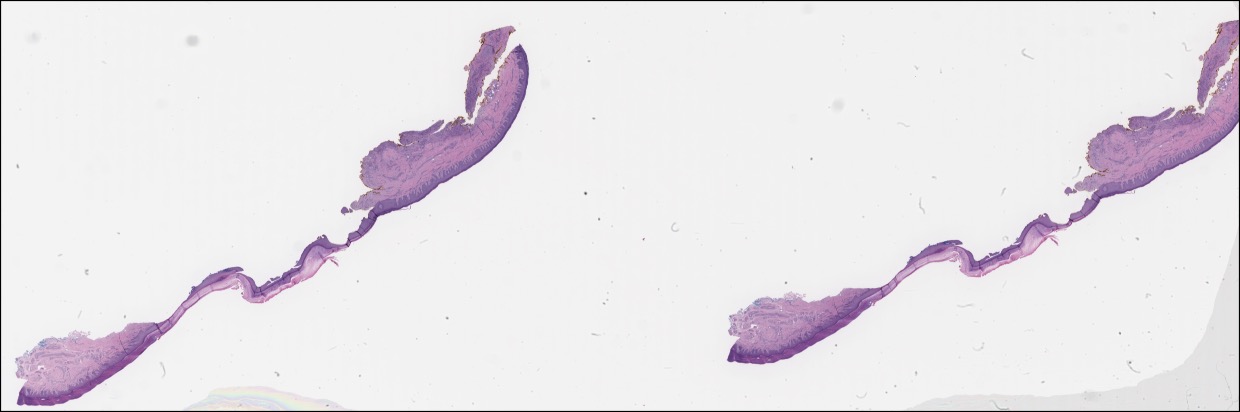

Supplement: Data S1. Illustrative low-resolution summary views of archival H&E-IHC whole slide image pairs, related to STAR Methods and Figure 1 — Details available in Tables S1 and S2. [file mmc2.zip › WSI-38_HE.jpg]

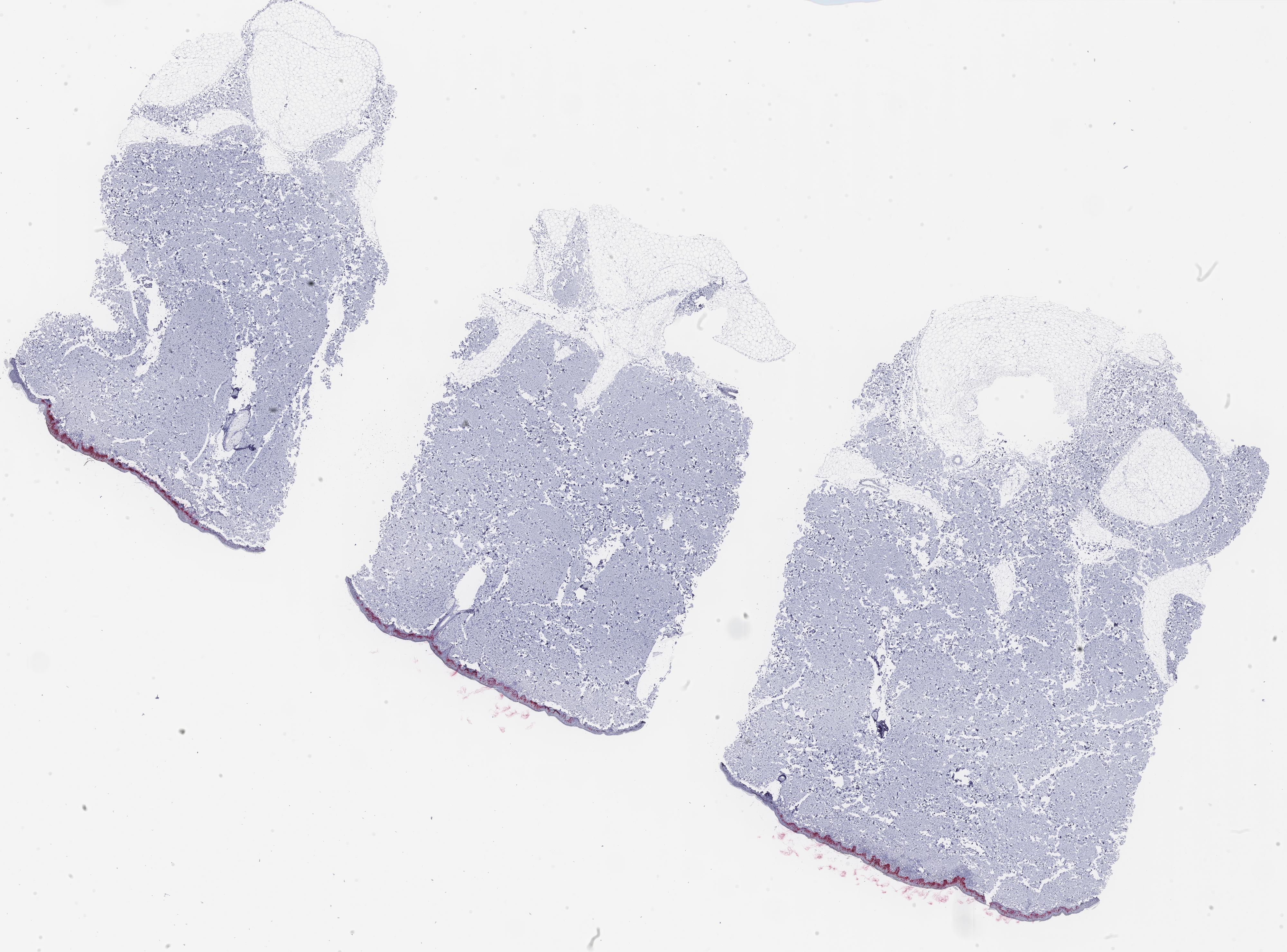

Supplement: Data S1. Illustrative low-resolution summary views of archival H&E-IHC whole slide image pairs, related to STAR Methods and Figure 1 — Details available in Tables S1 and S2. [file mmc2.zip › WSI-05_IHC.jpg]

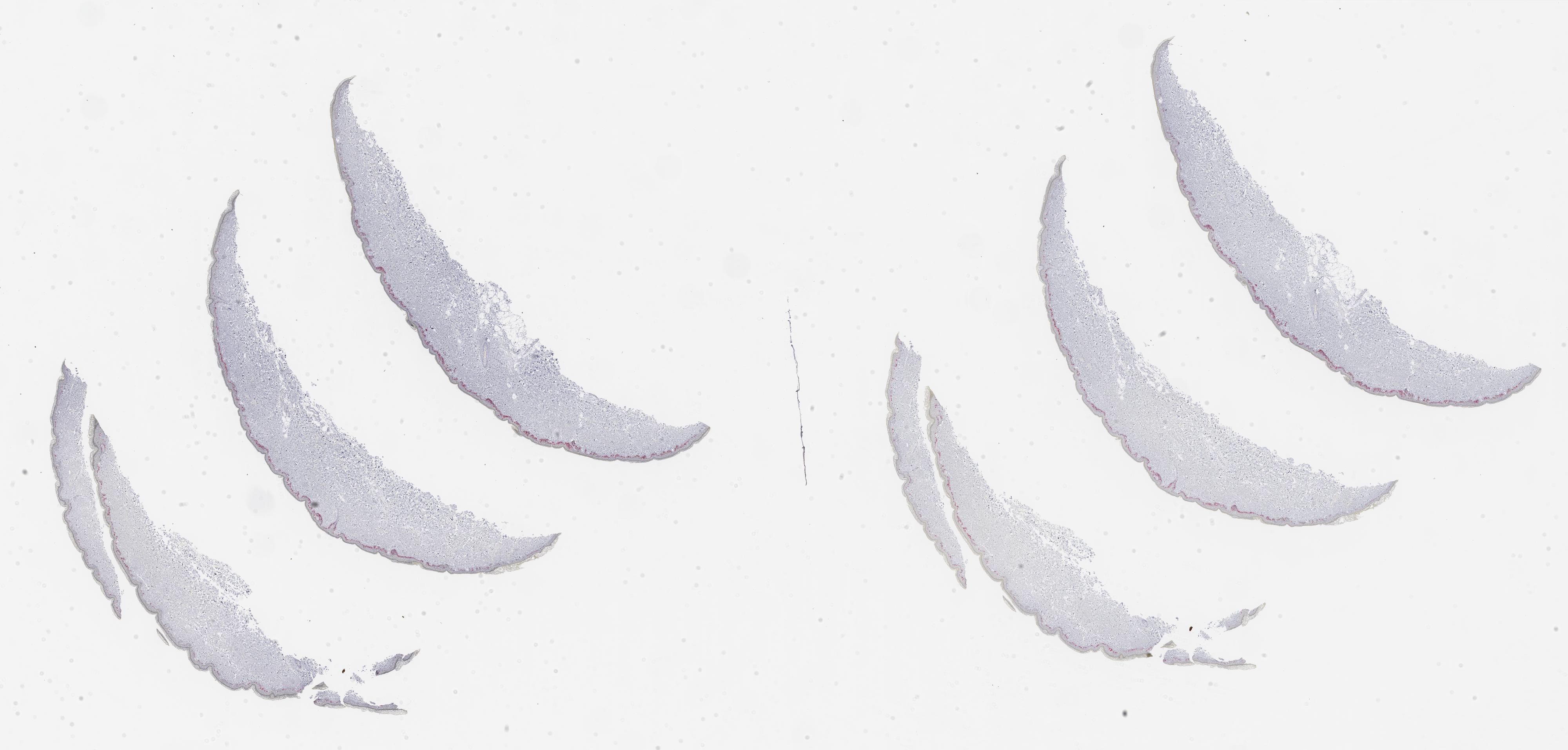

Supplement: Data S1. Illustrative low-resolution summary views of archival H&E-IHC whole slide image pairs, related to STAR Methods and Figure 1 — Details available in Tables S1 and S2. [file mmc2.zip › WSI-15_IHC.jpg]

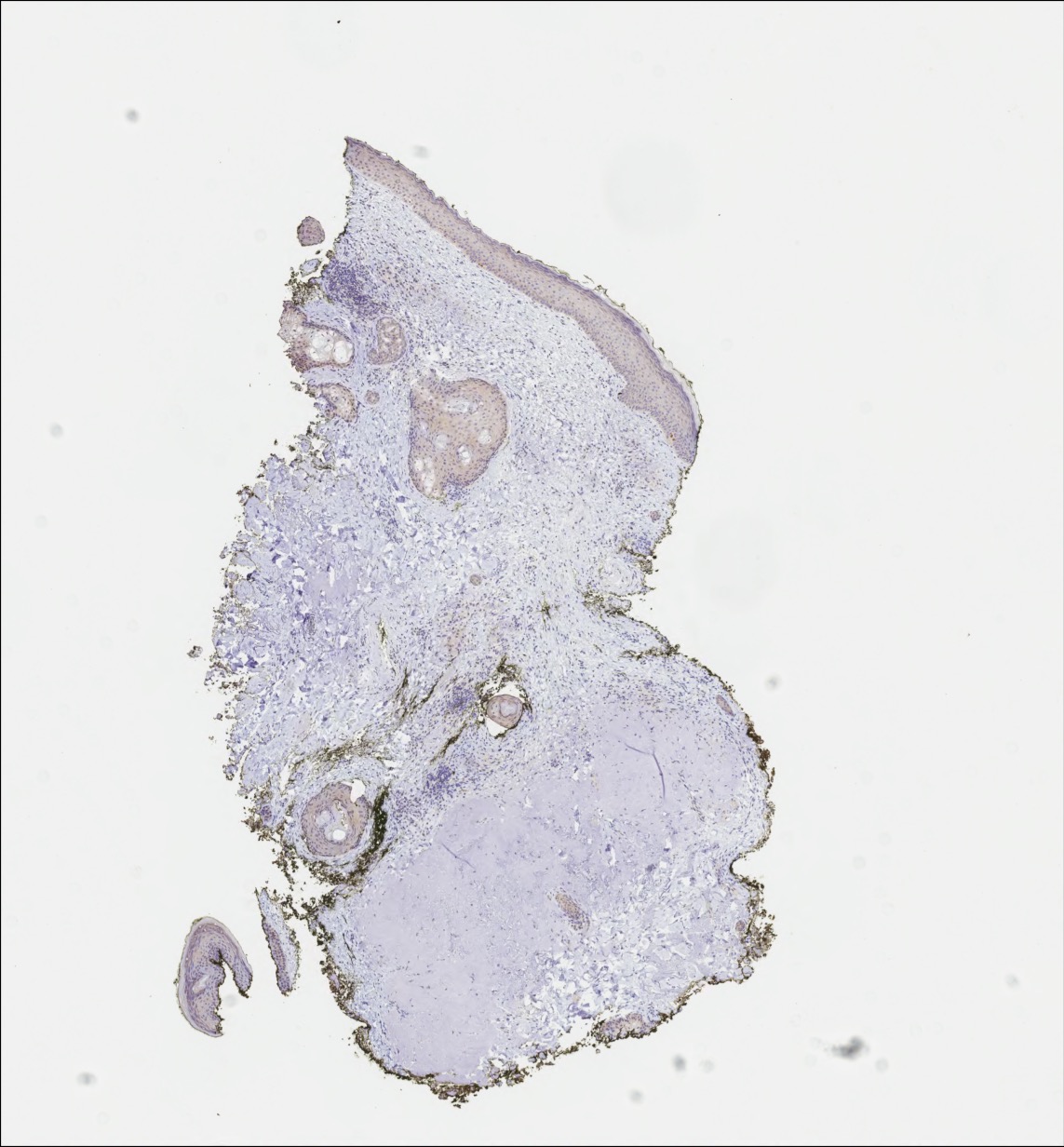

Supplement: Data S1. Illustrative low-resolution summary views of archival H&E-IHC whole slide image pairs, related to STAR Methods and Figure 1 — Details available in Tables S1 and S2. [file mmc2.zip › WSI-58_IHC.jpg]

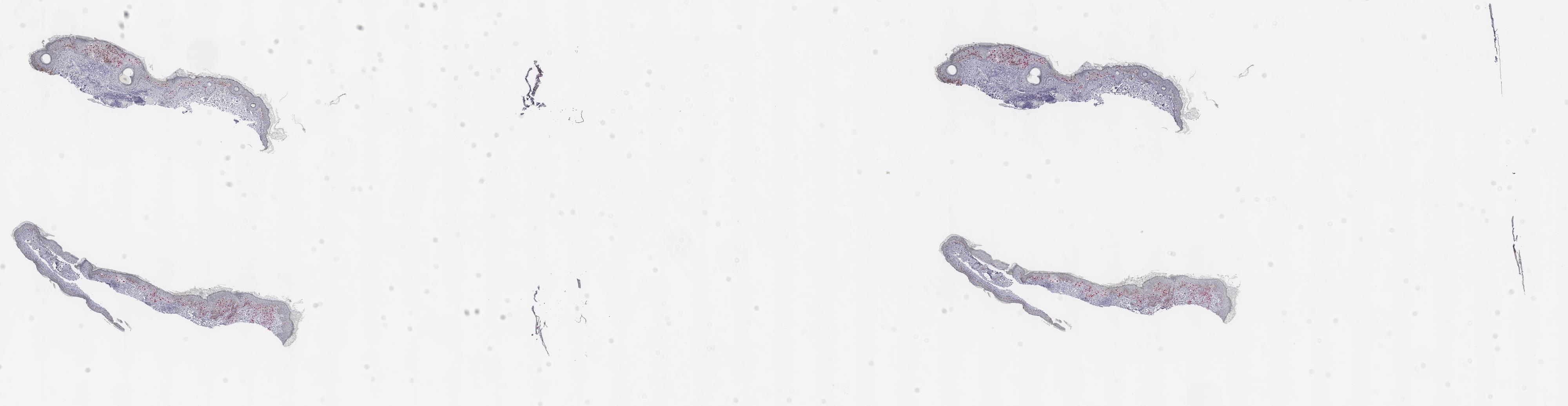

Supplement: Data S1. Illustrative low-resolution summary views of archival H&E-IHC whole slide image pairs, related to STAR Methods and Figure 1 — Details available in Tables S1 and S2. [file mmc2.zip › WSI-48_IHC.jpg]

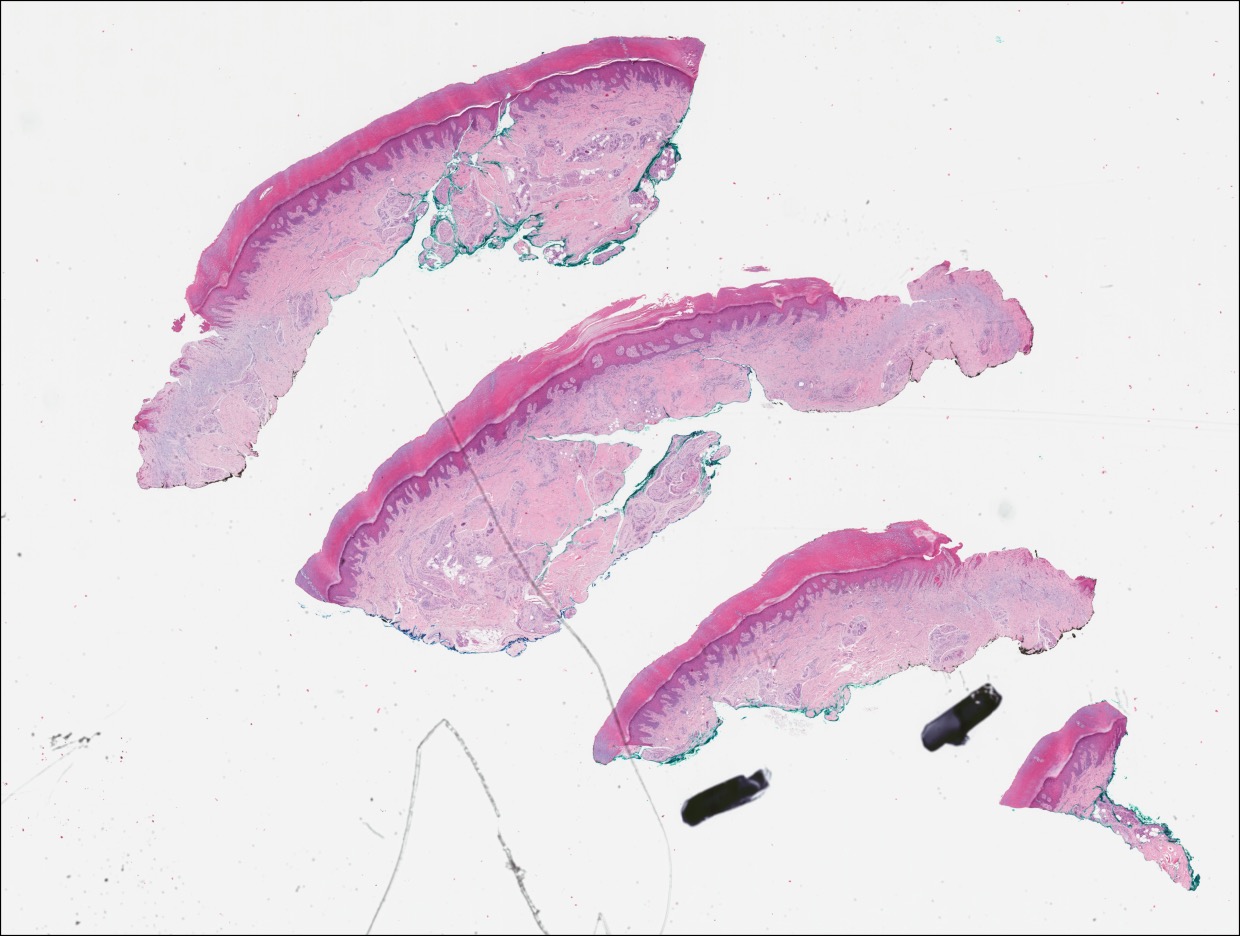

Supplement: Data S1. Illustrative low-resolution summary views of archival H&E-IHC whole slide image pairs, related to STAR Methods and Figure 1 — Details available in Tables S1 and S2. [file mmc2.zip › WSI-22_HE.jpg]

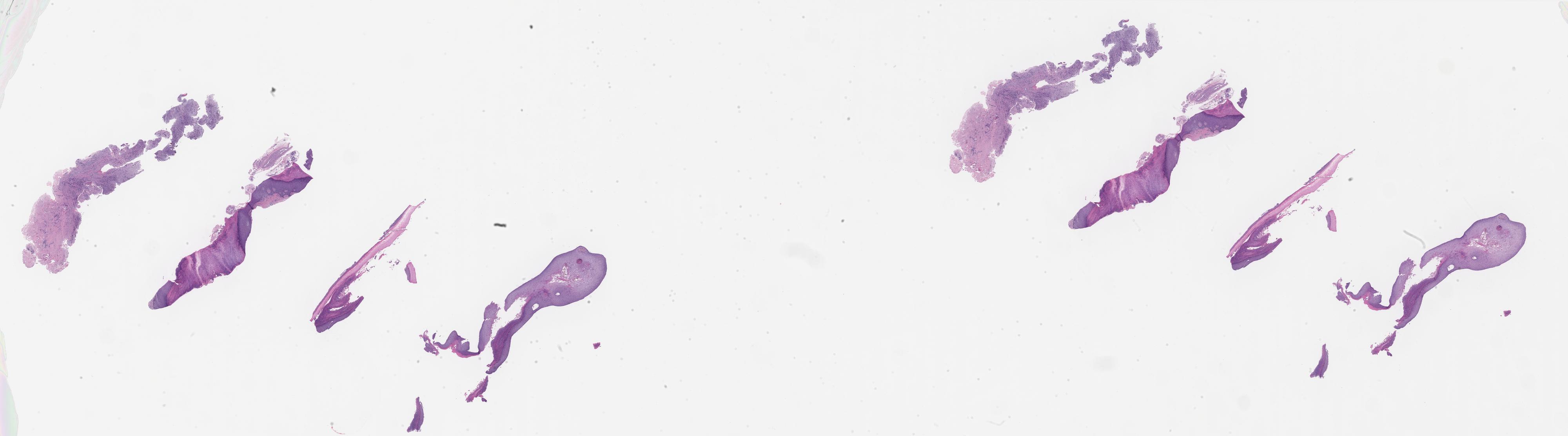

Supplement: Data S1. Illustrative low-resolution summary views of archival H&E-IHC whole slide image pairs, related to STAR Methods and Figure 1 — Details available in Tables S1 and S2. [file mmc2.zip › WSI-41_HE.jpg]

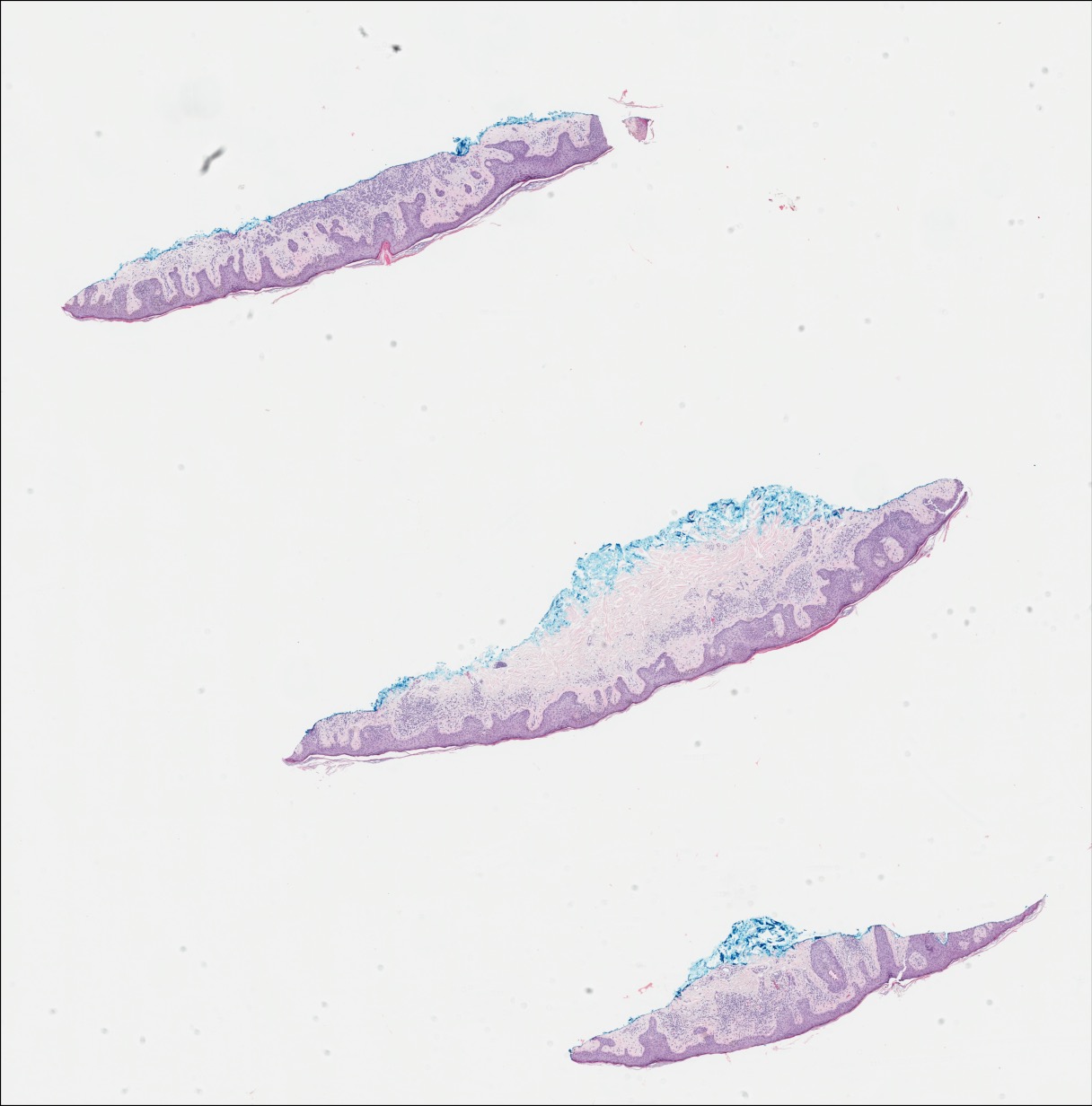

Supplement: Data S1. Illustrative low-resolution summary views of archival H&E-IHC whole slide image pairs, related to STAR Methods and Figure 1 — Details available in Tables S1 and S2. [file mmc2.zip › WSI-30_HE.jpg]

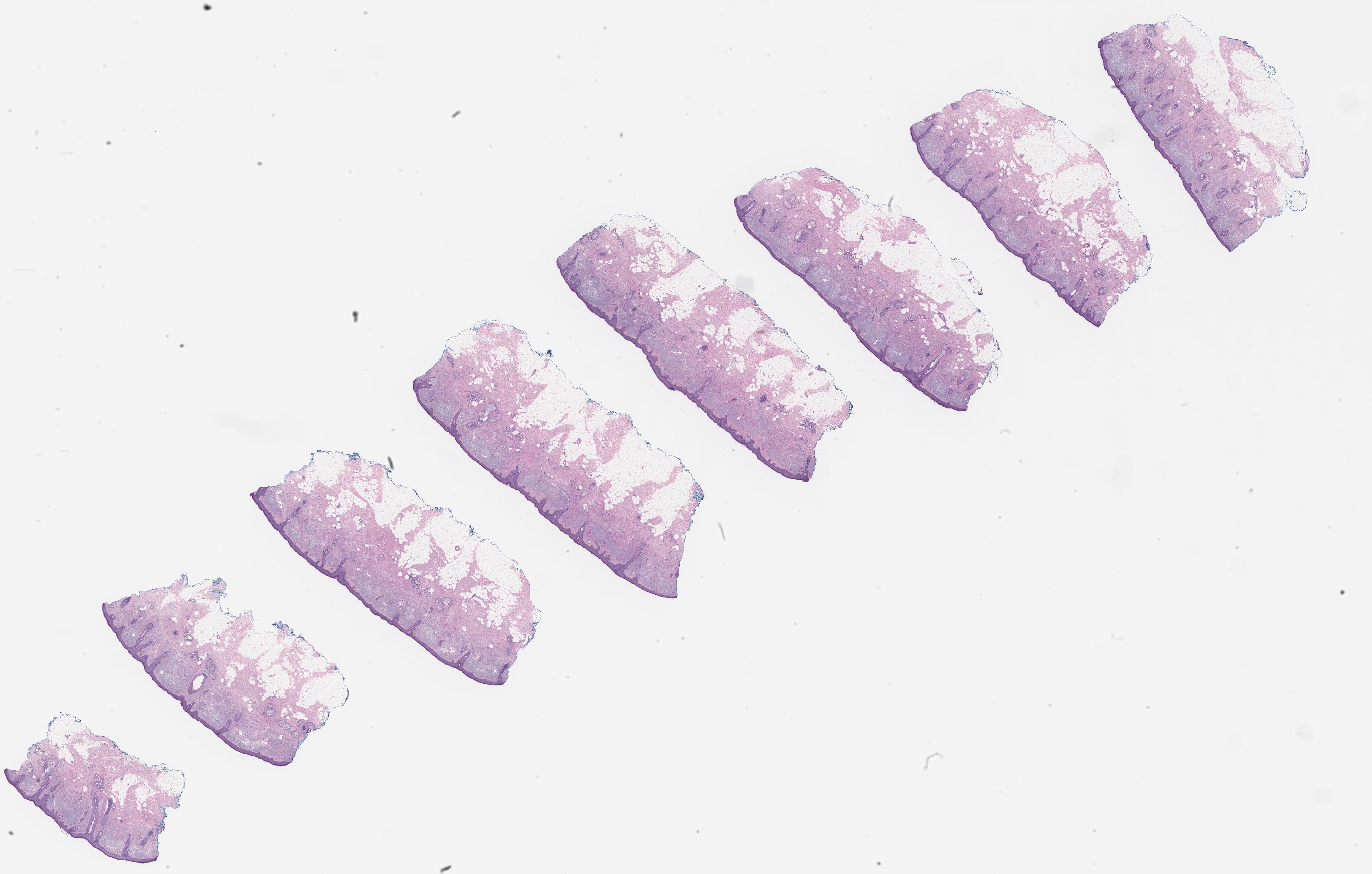

Supplement: Data S1. Illustrative low-resolution summary views of archival H&E-IHC whole slide image pairs, related to STAR Methods and Figure 1 — Details available in Tables S1 and S2. [file mmc2.zip › WSI-06_HE.jpg]

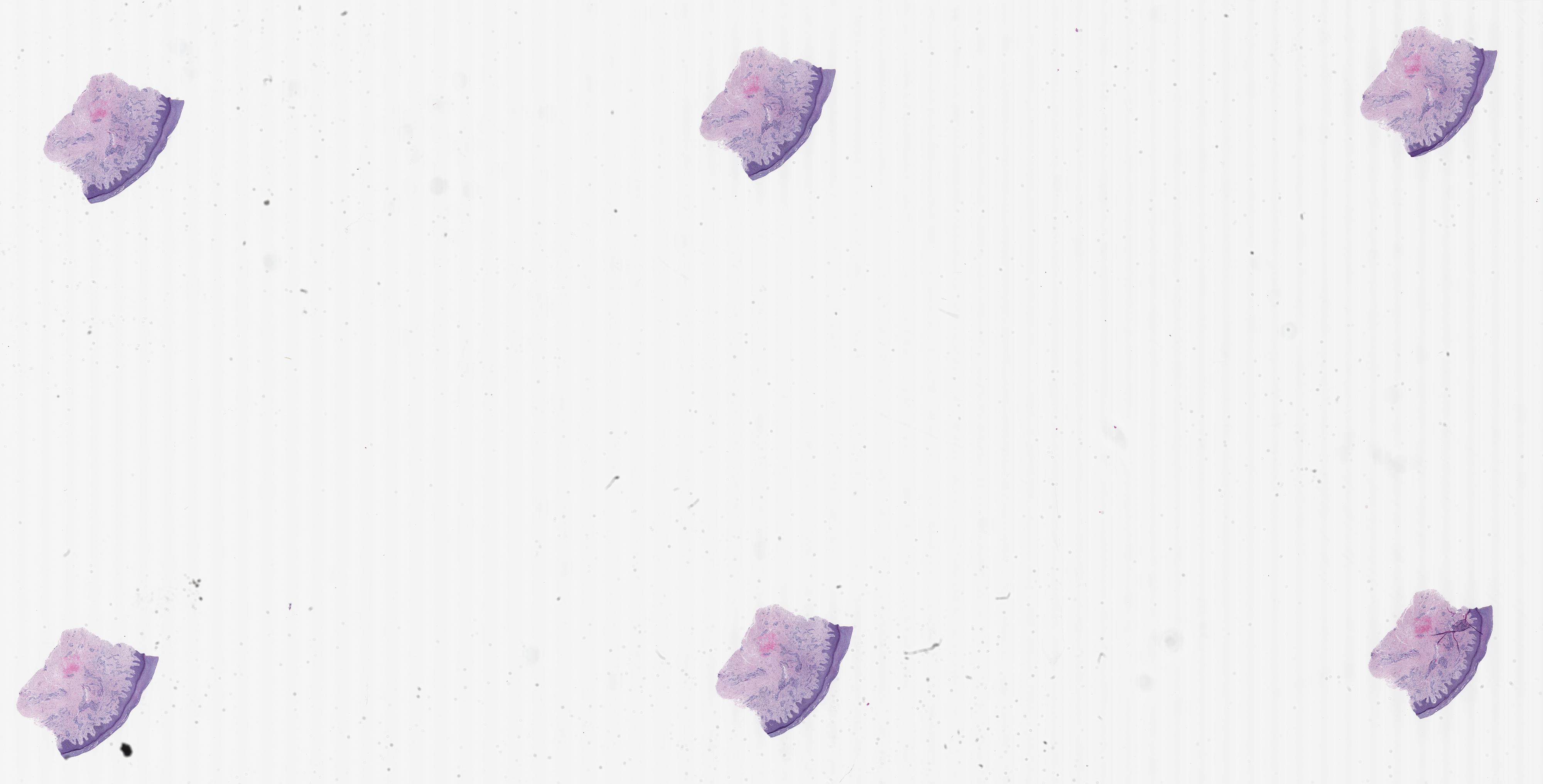

Supplement: Data S1. Illustrative low-resolution summary views of archival H&E-IHC whole slide image pairs, related to STAR Methods and Figure 1 — Details available in Tables S1 and S2. [file mmc2.zip › WSI-18_HE.jpg]

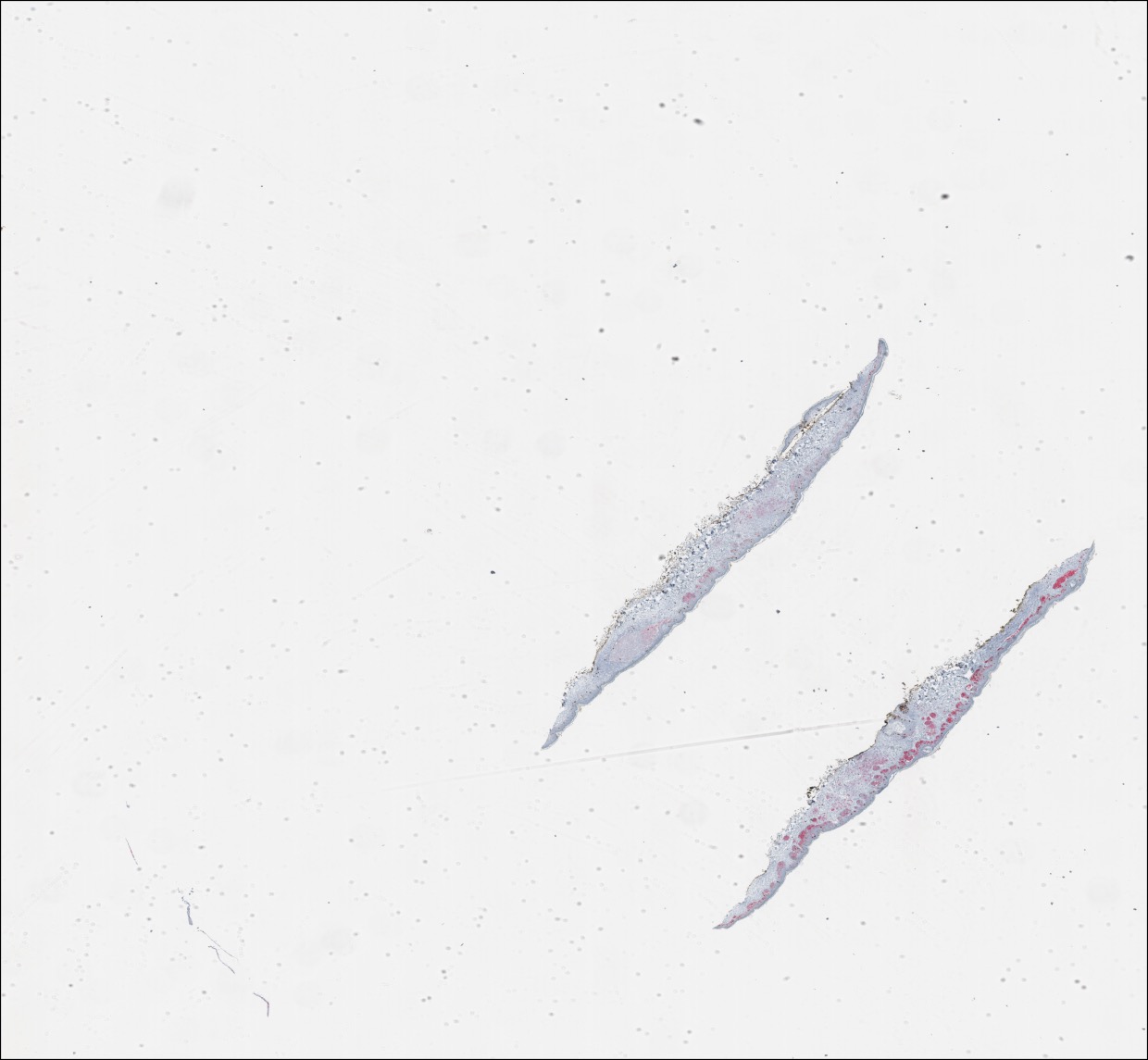

Supplement: Data S1. Illustrative low-resolution summary views of archival H&E-IHC whole slide image pairs, related to STAR Methods and Figure 1 — Details available in Tables S1 and S2. [file mmc2.zip › WSI-31_IHC.jpg]

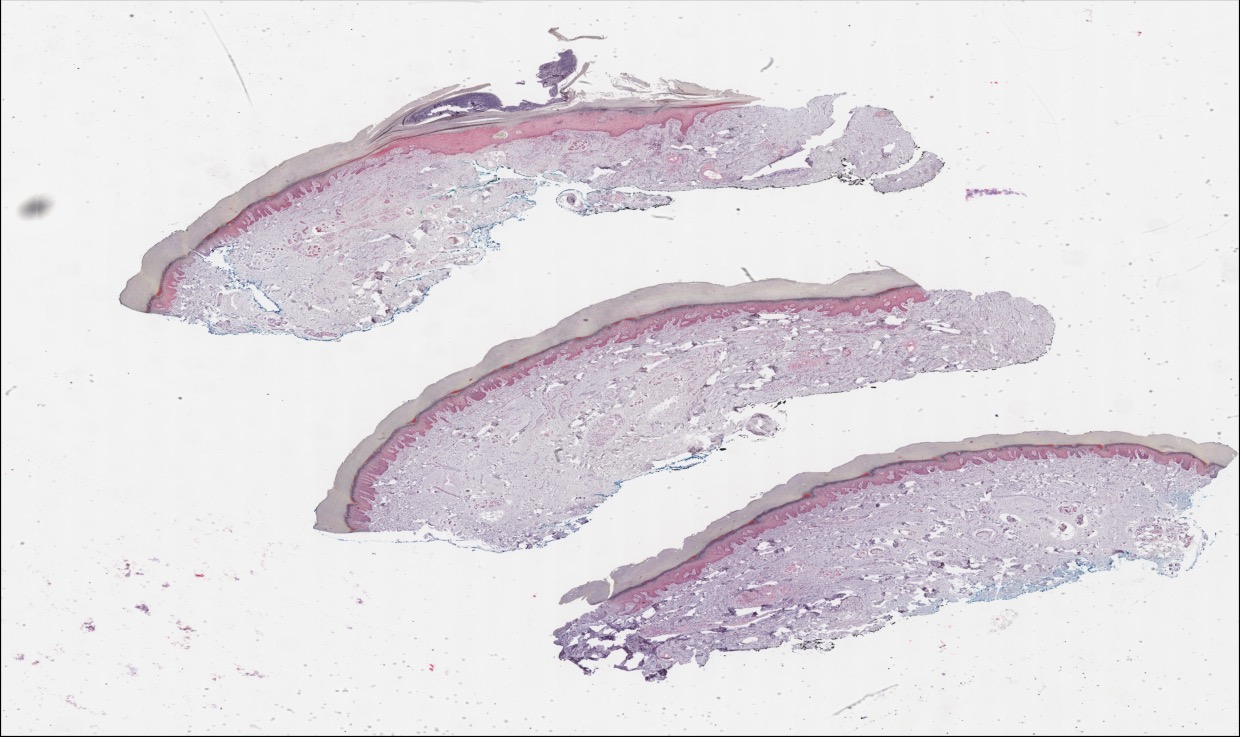

Supplement: Data S1. Illustrative low-resolution summary views of archival H&E-IHC whole slide image pairs, related to STAR Methods and Figure 1 — Details available in Tables S1 and S2. [file mmc2.zip › WSI-21_IHC.jpg]

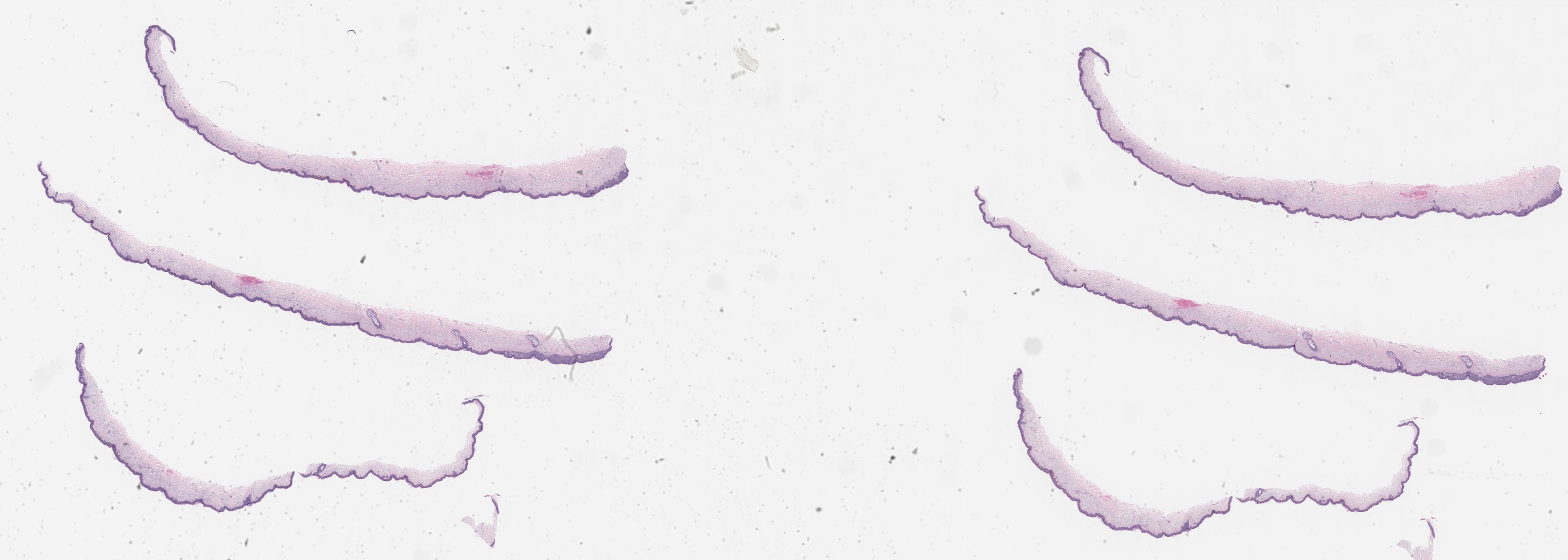

Supplement: Data S1. Illustrative low-resolution summary views of archival H&E-IHC whole slide image pairs, related to STAR Methods and Figure 1 — Details available in Tables S1 and S2. [file mmc2.zip › WSI-14_HE.jpg]

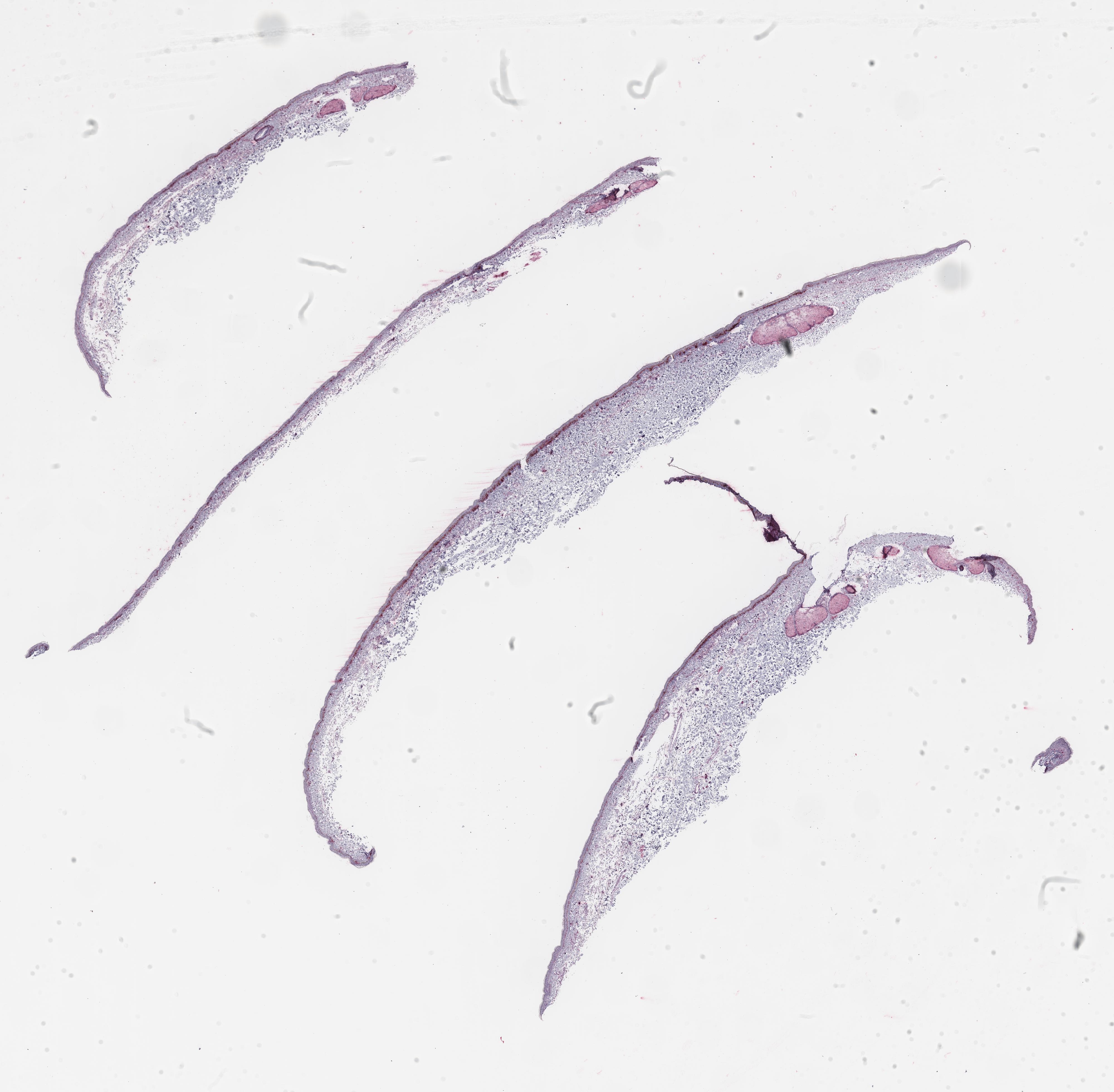

Supplement: Data S1. Illustrative low-resolution summary views of archival H&E-IHC whole slide image pairs, related to STAR Methods and Figure 1 — Details available in Tables S1 and S2. [file mmc2.zip › WSI-43_IHC.jpg]
