# Supplementary material for: Deep learning finds convergent melanocytic morphology despite noisy archival slides
Source: Cell Rep Methods. 2025 Oct 20;5(10):101201. doi: 10.1016/j.crmeth.2025.101201 (PMC12570353; doi:10.1016/j.crmeth.2025.101201)
Supplement: Document S2. Article plus supplemental information [file mmc3.pdf]

Cell Reports Methods

Article

Deep learning finds convergent melanocytic morphology despite noisy archival slides

Graphical abstract

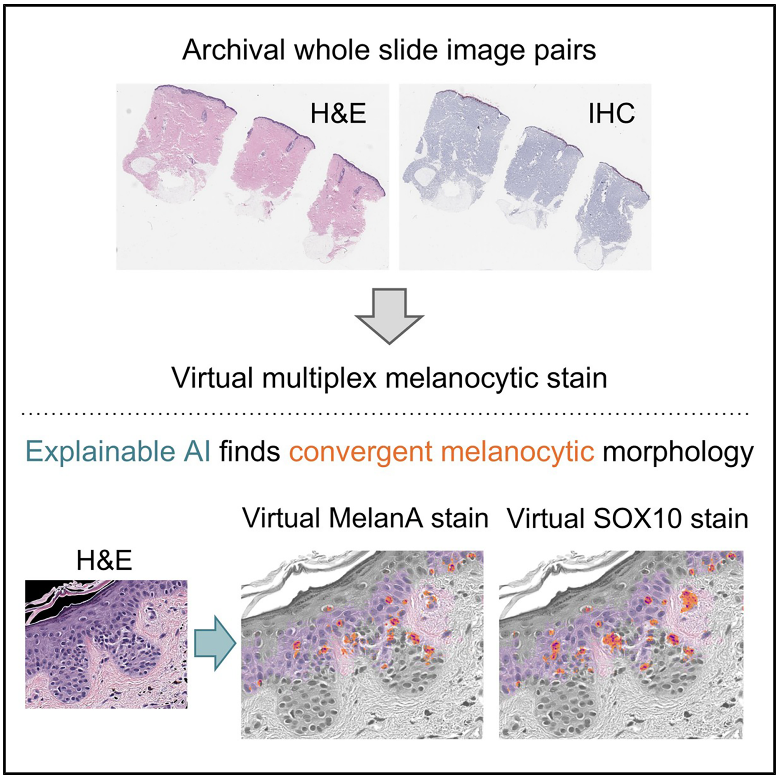

Authors

Mikio Tada, Garrett Gaskins, Sina Ghandian, Nicholas Mew, Michael James Keiser, Elizabeth Sarah Keiser

Correspondence

keiser@keiserlab.org (M.J.K.), e.keiser@gmail.com (E.S.K.)

In brief

Tada et al. develop virtual multiplexed immunohistochemical (IHC) stains for melanocytic atypia by learning from real-world H&E and IHC archival slide pairs. Despite noisy training from adjacent tissues, digital pathology models leveraging different subcellular melanocytic markers converge on consistent patterns by explainable AI, streamlining melanoma assessment workflows.

Highlights

- Deep learning creates virtual IHC stains from imperfect H&E-to-IHC slide pairs
- Models achieve AUROC = 0.948 (MelanA) and 0.867 (SOX10) despite noisy training data
- CNNs trained on different IHC stains converge on similar melanocyte morphology
- Saliency maps enable pixel-level multiplexed virtual IHC for melanocyte features

Tada et al., 2025, Cell Reports Methods *5*, 101201
October 20, 2025 © 2025 The Author(s). Published by Elsevier Inc.
https://doi.org/10.1016/j.crmeth.2025.101201

CellPress

Cell Reports Methods

CellPress
OPEN ACCESS

Article

Deep learning finds convergent melanocytic morphology despite noisy archival slides

Mikio Tada,[1,2,7] Garrett Gaskins,[1,2,7] Sina Ghandian,[1,2] Nicholas Mew,[1,2] Michael James Keiser,[1,2,3,4,8,*] and Elizabeth Sarah Keiser[5,6,*]
[1]Institute for Neurodegenerative Diseases, University of California, San Francisco, San Francisco, CA 94158, USA
[2]Bakar Computational Health Sciences Institute, University of California, San Francisco, San Francisco, CA 94158, USA
[3]Department of Pharmaceutical Chemistry, University of California, San Francisco, San Francisco, CA 94158, USA
[4]Department of Bioengineering and Therapeutic Sciences, University of California, San Francisco, San Francisco, CA 94158, USA
[5]Department of Pathology, University of California, San Francisco, San Francisco, CA 94143, USA
[6]Department of Pathology, San Diego Veterans Health Care System, San Diego, CA 92161, USA
[7]These authors contributed equally
[8]Lead contact
*Correspondence: keiser@keiserlab.org (M.J.K.), e.keiser@gmail.com (E.S.K.)
https://doi.org/10.1016/j.crmeth.2025.101201

MOTIVATION Despite great advances in computational pathology, deep learning models designed to identify pixel-level cytologic and architectural features typically rely on extensive pixel-level annotated training data. Manual annotation is a time-consuming task that limits scalability and introduces the risk of human error and annotator bias. We address this gap by introducing a scalable, open-source, weakly supervised method to create virtual immunohistochemical (IHC) stains. This deep learning approach leverages real-world archival H&E and melanocytic IHC stains to learn a precise pixel-level rationale for detecting melanocytes in cutaneous biopsies and excisions, without the need for manual annotations or large training datasets.

SUMMARY

Melanocytic atypia often leads to diagnostic discordance, complicating its prediction by machine learning models. To overcome this, we paired H&E-stained histology images with contiguous or serial sections immunohistochemically (IHC) stained for melanocytic cells via antibodies for MelanA, MelPro, or SOX10. We developed a melanocytic atypia deep learning pipeline from real-world archives of 122 paired whole slide images from 61 confirmed melanoma *in situ* (MIS) cases at two institutions. Only 37.7% of pairs matched well enough for deep learning; nonetheless, MelanA+MelPro models achieved an average area under the receiver-operating characteristic (AUROC) = 0.948 and area under the precision-recall curve (AUPRC) = 0.611 (9.3× baseline) and SOX10 models achieved AUROC = 0.867 and AUPRC = 0.433 (7.3× baseline). Despite learning from biologically different nuclear versus cytoplasmic IHC stains, convolutional neural network models exhibited a convergent explainable AI rationale. The resulting multi-antibody virtual stains identified cytologic and small-scale architectural features directly from H&E images, supporting pathologists in assessing cutaneous MIS.

INTRODUCTION

Whole slide scanned formalin-fixed paraffin-embedded (FFPE) tissue image analysis was only accepted in a research context[1–8] before the US Food and Drug Administration (FDA) approved the first whole slide imaging system for histopathology primary diagnosis in 2017.[9] The first FDA-approved machine learning-enabled medical device was also in the field of anatomic pathology for automated assessment of cervical cytology slides in 1995,[10] but radiology and several other medical subspecialties quickly surpassed pathology in the number of FDA-approved machine learning-enabled medical devices.[11] Currently, less than 1% of the 882 FDA-approved devices are specific to FFPE tissue pathology, of which a single device is FDA approved for automated histomorphologic organ-specific analysis.[12] Here, we develop an approach to making FFPE skin tissue histomorphologic machine learning algorithms for researchers and practicing dermatopathologists.

Convolutional neural networks[13] effectively identify and diagnose a range of pathologies,[14] including skin disease,[15–18] with diagnostic performance approaching that of a general practice pathologist in some cases.[19,20] Most of these achievements

© 2025 The Author(s). Published by Elsevier Inc.
This is an open access article under the CC BY license (http://creativecommons.org/licenses/by/4.0/).

rely on a paradigm of supervised learning, in which networks learn from a corpus of well-labeled, human-curated images. These models can generate predicted virtual stains meant to approximately replicate particular immunohistochemical (IHC) stains directly from H&E images.[21–23] Although new techniques are emerging to relax the resolution of labeling required,[15,24,25] pathologist-labeled images remain the currently accepted computational "ground truth," synonymous with the clinical "gold standard." However, if there is uncertainty regarding histopathologic ground-truth labels, the classification task becomes muddled and the model becomes less generalizable.

Melanocytic atypia is a particularly challenging field of pathology, with a spectrum of melanocytic atypia seen in benign (nevus), atypical (dysplastic nevus), and malignant (melanoma) lesions. Cytologically, melanocytes show a range of atypia, sometimes mimicking benign epidermal keratinocytes. Architecturally, atypical melanocytes display anywhere from benign-appearing to highly atypical growth patterns. Thus, pathologist interobserver agreement for melanocytic atypia in standard (H&E) histology images ranges between 33% and 68%, with atypical nevus versus malignant melanoma cases accounting for much of the diagnostic discordance.[26,27]

Various conventional classification methods for melanocytic atypia exist, with diagnostic top-line terminology ranging from eponymous labels to descriptive diagnoses with up to nine permutations of architectural and cytologic atypia. Currently, accepted diagnostic terminology includes "melanocytic atypia with mild/moderate/severe architectural disorder and mild/moderate/severe cytologic atypia," which some diagnostic laboratories have shortened to "mild/moderate/severe dysplastic nevus," or rarely just "dysplastic nevus," "atypical nevus," or "Clark's nevus," with a comment recommending surveillance or excision.[28,29] More recently, the World Health Organization proposed a two-grade system for dysplastic nevi with size criteria and intermittently ("low-CSD" [cumulative sun damage]) versus chronically ("high-CSD") sun-exposed diagnostic pathways for melanoma classification.[30] At times, it is not possible to differentiate between a moderately dysplastic nevus and one that requires excision due to incomplete biopsy or unknown therapeutic history. Such cases often receive the diagnosis "atypical melanocytic neoplasm" or "atypical melanocytic proliferation" to communicate the need for additional clinical correlation, including further sampling or excision.

Severely dysplastic nevi and melanoma *in situ* (MIS) undergo similar surgical treatment, but the diagnoses impart crucial "atypical" versus "malignant/cancer" implications to the patient. While researchers could collapse these two diagnoses into a single category for a supervised learning task, it would be clinically misleading. In supervised machine learning (ML), the utility of a learned function is dependent upon the accuracy and reliability of the labels used to train it. To circumvent the issue of diagnostic discordance negatively contributing to our "ground-truth" labels, we apply a pathologist-agnostic method to identify melanocytic atypia in patient tissue sections.

Pathologists employ IHC stains to highlight specific cell types in diagnostically challenging cases. Correspondingly, our computational method uses the melanocytic IHC-stained sections from archival patient cases to auto-label (notate) approximate melanocyte location within corresponding H&E tissue sections of diagnosed cases of MIS. A convolutional neural network (CNN) ingests 64 $\times$ 64 micron tiles (high-power views) of image data from the H&E section for training, with the tile labels extracted from the paired but unseen IHC section as the source of the melanocyte-specific labeling. While IHC stains highlight specific cells, the field of view visible in a "tile" in these models is intentionally larger than the size of a single cell. Thus, a computational algorithm requires a binary "tile-level label" of "*increased* melanocytes present" vs. "*few to no* melanocytes present," which is determined by a fixed stain-specific cutoff within each IHC-stained model. Just as pathologists do not study and diagnose melanoma by solely viewing a melanocytic IHC stain without an H&E paired section, we do not train our CNN on IHC tissue samples directly. Instead, we assemble a custom dataset composed of adjacently sliced and paired tissue sections. Each set of paired tissue consists of at least one tissue level that is IHC stained and a complementary level that is H&E stained. We refer to both tissue profiles collectively as a "sample pair."

The computational extraction of IHC stain information for each sample pair allows the method to incorporate information regarding melanocyte location and morphology (Figure 1) while only training on H&E data with known pathologist-assigned diagnoses, e.g., MIS in this study. Ideally, the CNN learns features indicative of melanocytic atypia that are generalizable to new H&E images. While others have also adopted this paired-stain deep learning training strategy, their approach required laborious custom tissue restaining or creation of new "back-to-back" H&E and IHC paired slides instead of leveraging existing clinical archival slides of nonidentical tissue sections.[23,31] The method relies on a critical assumption: that sequential tissue sections from the same tissue block (some "contiguously" cut "back to back" by the same histotechnologist at the same point in time, and others cut "serially" or "discontinuously" at two different points in time with the tissue block re-faced for the IHC stain) are similar enough in morphology and location that IHC staining from one slice can serve as a proxy label for its adjacent (H&E) pair. As we leverage archival slide pairs from historical cases, we further benefit from the observation that these slide pairs were sufficiently well matched for dermatopathologists to make diagnoses in clinical practice. To ensure optimal morphological congruence among paired H&E and IHC images, we align sample pairs at their native 40$\times$ resolution. We then train a CNN, specifically DenseNet121,[32] to identify melanocytic atypia in H&E images at the tile level. Although precise individual cell assignment even among entrapped or MIS-neighboring melanocytes can be difficult to determine without genetic profiling,[33] we refer to melanocytes within MIS as "atypical melanocytes" due to their association with malignant lesions for simplicity.

We assess the performance of the (H&E) trained networks by their ability to classify tiled images from validation of H&E patient tissue sections as either melanocyte containing or non-melanocyte containing, using unseen aligned IHC-stained tissue as ground truth. We generate high-resolution prediction heatmaps on validation tissue sections (H&E) for slide-level interpretation. We also perform an AI/ML explainability technique called saliency mapping to generate fine-grained maps highlighting which

CellPress
OPEN ACCESS

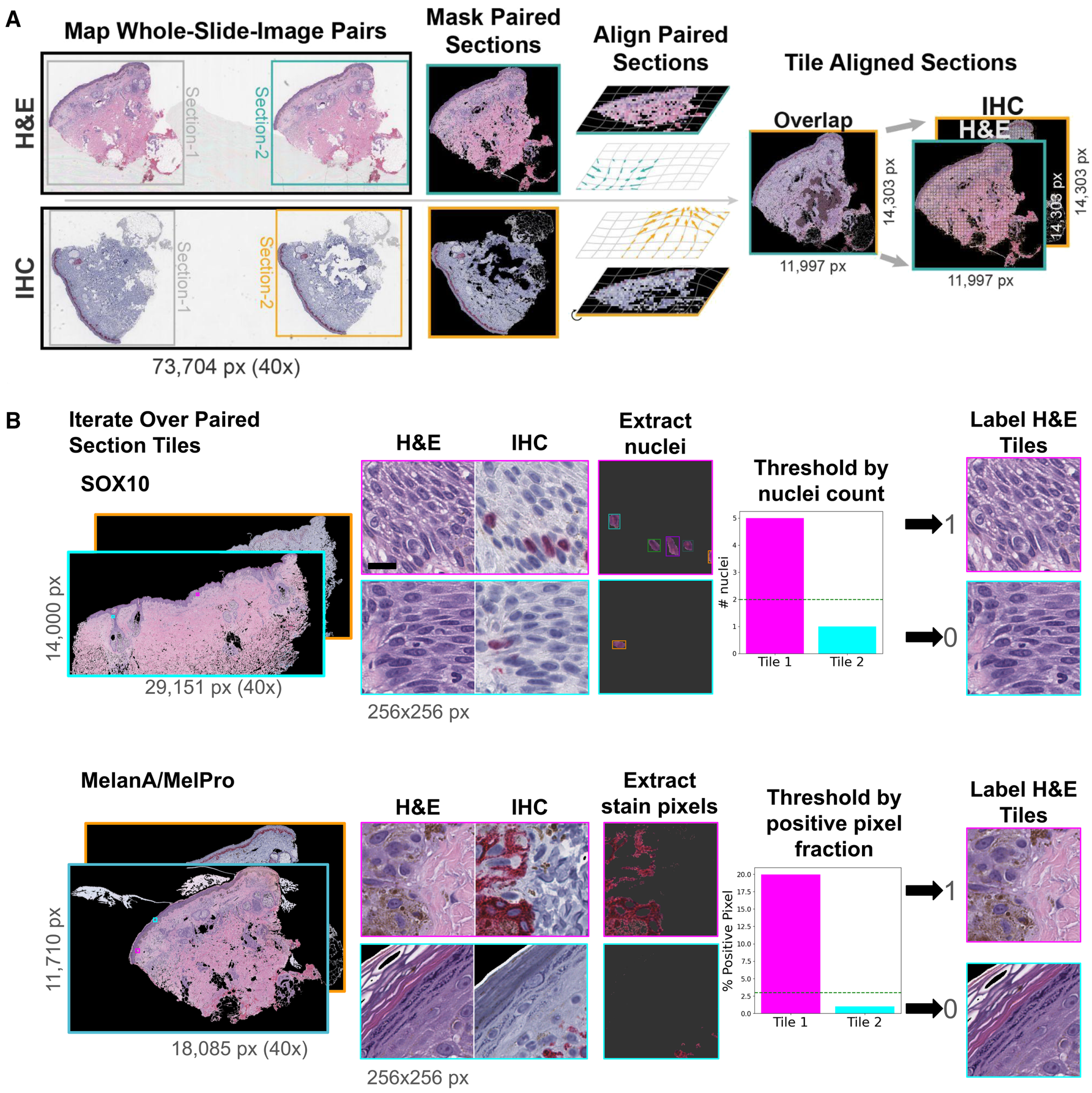

Figure 1. Whole-slide image processing pipeline

(A) Tissue section preparation and alignment: We extract and mask matching tissue sections from separate H&E- and IHC-stained WSIs, forming a pair. We align tissue sections using ImageJ's b-spline alignment algorithm (bUnwarpJ) to ensure optimal congruence at the tile level. Following alignment, we independently tile tissue sections.

(B) Tissue tile labeling: We label H&E tiles based on the associated IHC stain. For tiles corresponding to SOX10 stain, labeling is based on the count of nuclei, while for those with MelanA stain, it is based on the proportion of positive pixels. Scale bars, 16 μm.

tissue regions contribute the most to the neural network's detection of melanocytic tissue.

Teaching a supervised model to learn where melanocytes are present in an H&E tissue section, with confirmation solely from approximate information from a corresponding IHC stain, helps to reduce the necessity, cost, and time of manual dermatopathologist labeling and avoids the issues of human bias and noisy labeling.[26,27] We hope this "virtual stain" method will assist the general clinical pathologist in rapidly and effectively identifying cutaneous melanocytes, improving turnaround times, and reducing healthcare costs for new cutaneous MIS diagnoses.

RESULTS

MelanA and SOX10 IHC stains are among the most common in melanocytic dermatopathology. We created two separate CNNs, trained each on a specific stain, and evaluated how they agreed and differed.

We algorithmically generated stain labels from IHC sections aligned to H&E sections

We developed a stain-agnostic melanocyte presence labeling pipeline that annotated whether a small (64 × 64 microns;

256 × 256 pixels) H&E tile contained melanocytic cells using sequentially sliced matched nuclear and cytoplasmic IHC-stained tissue images. Using adjacent IHC-stained tissue as an approximate answer or "label" complements studies that rely on collecting training data from pathologists' manual annotations. Despite the physical differences between different tissue sections, we demonstrated a computationally efficient method to label melanocytic cells in archival H&E and IHC whole slide images (WSIs) accurately. Because our dataset is archival, the H&E sections were not always "back to back" or cut strictly adjacent to the IHC sections and may vary slightly in architecture and morphology. Consequently, a raw IHC WSI file, spanning tens of thousands of pixels in height and width, could not serve as a proxy to label for melanocytic cell presence to a matched archival H&E without further image processing. Accordingly, we first separated the WSI into individual tissue sections to remove slide white space and to account for variations in tissue section placement or orientation (see tissue extraction and filtering, STAR Methods). Next, we tackled coarse-grained H&E-to-IHC section dissimilarity by algorithmically aligning the position, orientation, and scale of the IHC section to match a paired H&E section using publicly available software, bUnwarpJ[34] (Figure 1A). This tool also computes limited "warping" corrections, such as adjusting for minor shearing or bunching of the tissue samples. High-magnification differences typically persisted between H&E and IHC tissue sections, as evident when comparing alignment fidelity within 256 × 256 pixels H&E and IHC tiles (Figure 1B). However, the exact variation in tissue structures across tiles became insignificant when we consolidated the IHC tile's signal into a single binary annotation per H&E tile (Figure 1B, rightmost columns). Consequently, the neural networks never received within-tile label information during training. However, this inherent limitation to the slide processing methods did restrict our pipeline to processing WSIs at a maximum optical magnification of 40× (0.25 microns per pixel [MPP]), as the alignment limitations did not support a finer-grained training label.

We developed separate methods to label H&E tiles by paired SOX10-stained tiles versus MelanA- and MelPro-stained tiles, tailored to the IHC stain's biological characteristics and to facilitate downstream *in silico* multiplex melanocytic staining. SOX10 is a nuclear stain, so we counted stained nuclei. Since MelanA and MelPro are cytoplasmic stains, we followed a more cytoplasmic logic for these stains (hereafter simplified to "MelanA") by calculating the proportion of stain-positive pixels within an IHC tile. We set positive-label thresholds by dermatopathologist review (E.S.K.), requiring at least two stained nuclei for SOX10 and 3% of the pixel area to be stain positive for MelanA (Figure 1B). As expected, increasing these thresholds reduces the total count of positively labeled tiles, while lowering them has the opposite effect (Figure S3). We systematically checked the impact of different training label threshold choices on model performance to ensure the robustness of our approach (Figure S3). We tested pixel proportion thresholds ranging from 1% to 5% for MelanA and MelPro, while for SOX10, we evaluated nuclei count thresholds from 1 to 5. When evaluated on a fixed test set (minimum 3% for MelanA and MelPro and ≥2 nuclei for SOX10), the models demonstrated stable area under the receiver-operating characteristic (AUROC) and area under the precision-recall curve (AUPRC) across the ranges. The MelanA/MelPro model achieved consistently high AUROC (0.94–0.95) with minimal variation, while the AUPRC scores remained steady from 0.58 to 0.61 (Figure S3). Similarly, the SOX10 model achieved AUROC between 0.78 and 0.87 and AUPRC between 0.39 and 0.43 (Figure S3). The models were robust to these biologically reasonable variations in training threshold choice.

Models detected melanocyte cells in H&E-stained tissue

We created *in silico* multiplex IHC stains from archival H&E tissue by developing CNN models that assessed the likelihood of melanocytic cell presence one H&E tile at a time. We trained DenseNet121 CNN models[32] on H&E tiled images labeled from the MelanA and SOX10 datasets. Many of the archival WSIs we initially collected failed in the data processing step due to insurmountable tissue structure differences between H&E vs. IHC sections or digitization artifacts such as blurriness—we discarded 62% of the original 61 H&E-IHC WSI pairs (Table S2 "Action" column). Consequently, a case-stratified static hold-out test set strategy was not feasible at this dataset size. To evaluate the models reliably, we performed 5-fold cross-validation and reported the average performance among the folds.

MelanA and SOX10 models achieved strong AUROCs (0.948 ± 0.023 and 0.867 ± 0.091) with good AUPRCs (0.611 ± 0.091 and 0.433 ± 0.165) (Figure 2A). We also constructed "label maps" directly on the hidden IHC tissue sections to visually evaluate this numerical performance. The logic was that the training pipeline, by definition, could not generate cell-level melanocyte predictions because we could only train the CNNs to generate binary yes/no predictions at the level of 256 × 256-pixel (64 × 64 microns) fields of view. While we could progressively "slide" the CNN's field of view one pixel at a time across the entire H&E section, we still needed to average the resulting overlapping predictions to generate the "prediction heatmap" (Figure 2B, middle column). Consequently, even a "perfect" CNN could not generate heatmaps more fine grained than the analogous overlapping sliding-window procedure on the hidden paired IHC, which we termed "label maps" (Figure 2B, right column). Whole-tissue prediction heatmaps achieved excellent fidelity to the IHC label maps, particularly in the high-confidence (dark purple overlay) regions (top row, Figures 2B and 3B). In the zoomed insets (bottom row, Figures 2B and 3B), both models appear slightly oversensitive in their melanocytic cell area prediction, consistent with the trade-offs shown in their precision-recall curves (right, Figures 2A and 3A). Naive 1-pixel-at-a-time ("1-pixel stride") heatmaps require $10{,}000^2$ = 100 million CNN predictions per 10k-by-10k pixel WSI, so we generated whole-tissue heatmaps using a 6-pixel stride and reported dataset-wide performance metrics using a 256-pixel stride. Performance correlated closely between 6-pixel and 256-pixel strides (Figure S4).

To bolster generalizability, we trained models with non-cutaneous H&E training images from the The Cancer Genome Atlas (TCGA) as additional negative examples. We assessed this strategy against two other training regimes: (1) zero TCGA tiles and (2)

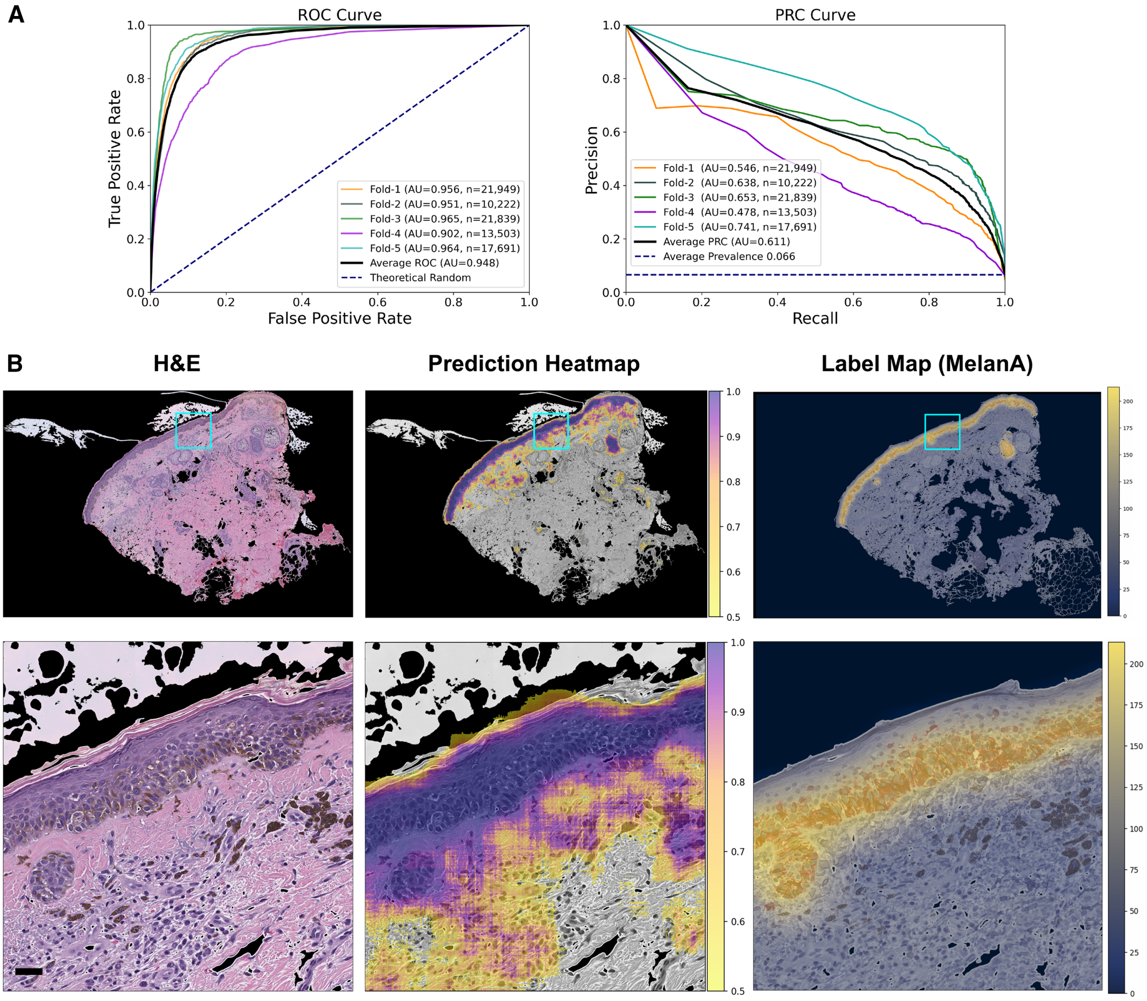

Figure 2. MelanA melanocyte-detector evaluation

(A) Performance metrics: We evaluate the models, labeled based on either MelanA or MelPro IHC stains, using five distinct test sets from a 5-fold cross-validation. Each fold splits patients into train vs. testing categories. The AUROC (left) and AUPRC (right) curves display the performances across these test sets.

(B) Visualizing melanocytes predictions: The top row compares the original H&E image, the prediction heatmap, and the corresponding ground-truth IHC label map. The bottom row shows a zoomed-in, higher resolution segment of the top row image, highlighted in blue. Scale bars, 32 μm.

half as many TCGA tiles injected into the negative training data. Incorporating non-cutaneous TCGA H&E tiles did not harm model performance (Figure S1); we observed robust AUROC and AUPRC scores across conditions. A user may elect to exclude TCGA-derived negative training if conceptually preferred. Interestingly, however, including TCGA tiles in the training data resulted in a better model by some criteria. For instance, training on the chosen TCGA fraction achieved lower standard deviation across 5-fold cross-validation for both MelanA+MelPro and SOX10 models compared to models trained on zero and half TCGA conditions.

Models accurately identified melanocytes with biologically sensible edge cases

We used validation tissue tiles of H&E-stained sections paired with IHC-stained sections to test the MelanA and SOX10 models. Overall, the models accurately and reliably identified H&E-stained tiles with increased numbers of melanocytes (Figure 4).

Melanocytes show a spectrum of morphologies, from small hyperchromatic nuclei with perinuclear clearing to enlarged nuclei with nucleoli and characteristic “battleship gray” cytoplasm.[35] Some melanocyte cytologic features overlap with keratinocyte cytologic features, making cell-specific identification particularly challenging in H&E-stained images. We wondered whether the SOX10 and MelanA CNN models had learned to leverage these characteristic morphological features or instead relied on an unrelated numerical logic. To assess this, we examined prediction successes and failures at the single-tile level (256 × 256 pixels tile with 0.25 MPP, so 64 × 64 microns, Figure 4). We display a representative subset of true-positive, true-negative, false-positive, and false-negative tiles for each model, arising from tile-level predictions of whether predicted melanocyte counts exceeded a minimum threshold. In the

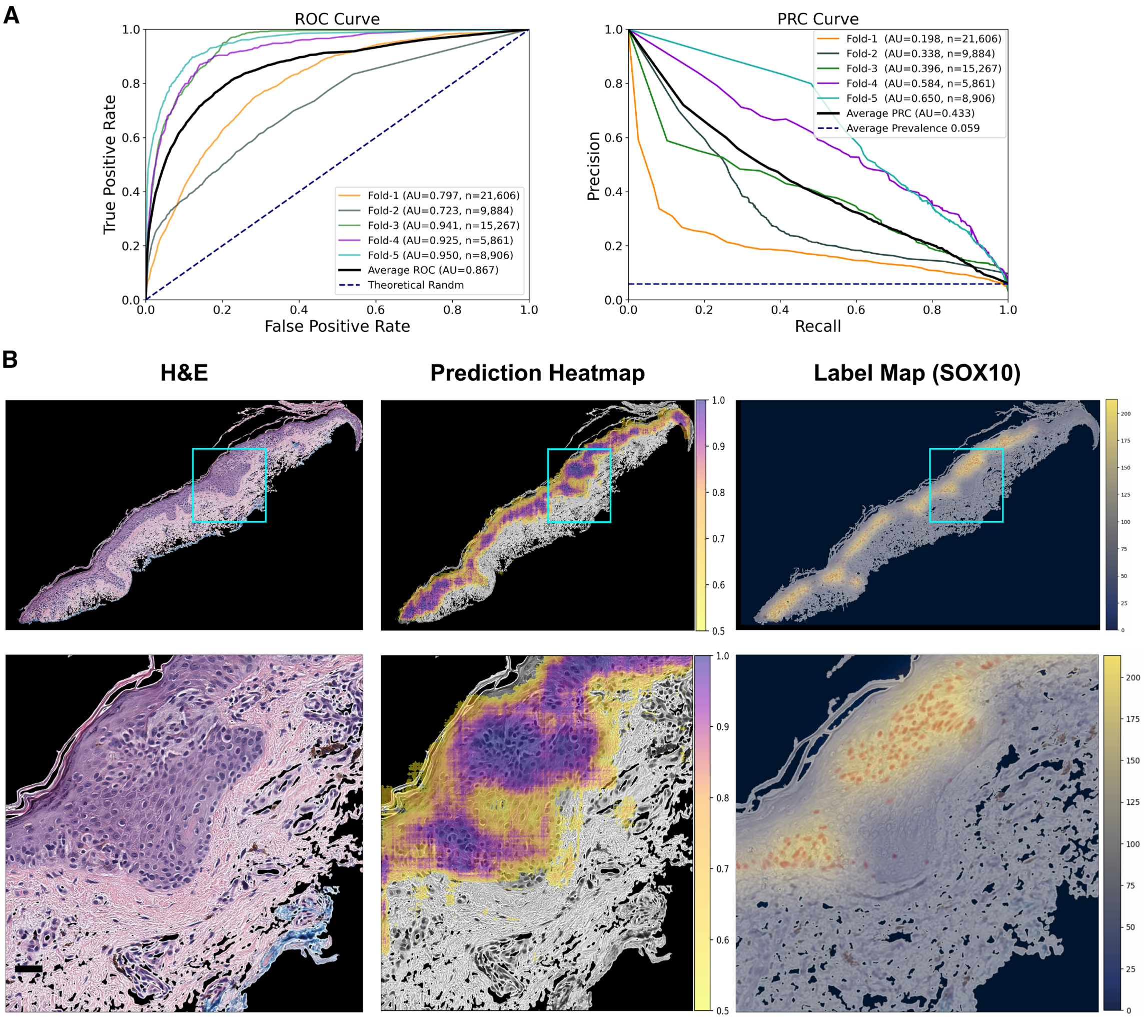

Figure 3. SOX10 melanocyte-detector evaluation

We prepared (A) and (B) as in Figure 2, but using SOX10 IHC-stained whole slide images. Scale bars, 32 μm.

previous section, the prediction heatmaps (Figures 2B and 3B, top row) visualize averaged prediction confidences by overlapping individual predictions for these high-power (40×) but small field-of-view (64 × 64 microns) tiles to simulate a low-magnification view of the entire tissue. MelanA and SOX10 models accurately identified tiles with increased numbers of melanocytes, including tiles with subtle melanocytes interspersed within the epidermal keratinocytes (e.g., "true-positives" in Figures 4A and 4B).

Differentiating eccrine glands from melanocytic nests was more challenging for the models than it typically would be for pathologists. Eccrine glands display small hyperchromatic nuclei and amphophilic cytoplasm, similar to some melanocytes, but their duct and gland architecture plus deep dermal location are generally clear non-melanocytic indicators to pathologists. SOX10 is known to stain eccrine myoepithelial and secretory cells, whereas MelanA does not. The MelanA model incorrectly identified some eccrine glands as melanocytes (e.g., "false-positive" tiles *xix*, *xxii*, and *xxvi* of Figure 4A) but still appropriately identified other "true-negative" eccrine gland tiles (e.g., *xii*, *xv*, and *xviii* of Figure 4A). Since the SOX10 model inherits the limitations of the SOX10 antibody by its training, it is unsurprising that this model also identified some "true-positive" tiles that contain eccrine glands instead of melanocytes. Consequently, numerical performance scores for the SOX10 model (e.g., Figure 3A) formally reflect prediction of positive IHC signal instead of melanocyte-specific signal. However, the model explainability methods (Figure 5) reveal that both models, nonetheless, assign similar importance to nuclear and cytoplasmic features in eccrine glands in H&E-stained images despite their positive SOX10 and negative MelanA expression (false-positive tile sets *iv* and *vi* in Figure 5B and true-negative tile sets *ii* and *iii* in Figure 5C).

The SOX10 model incorrectly rejected some tiles with at least three melanocytes in the H&E image despite the image pairs being back to back, with the same cells in both images. In these scenarios, the algorithm did not correctly identify the small hyperchromatic melanocytes (e.g., "false-negative" tile *xxx*,

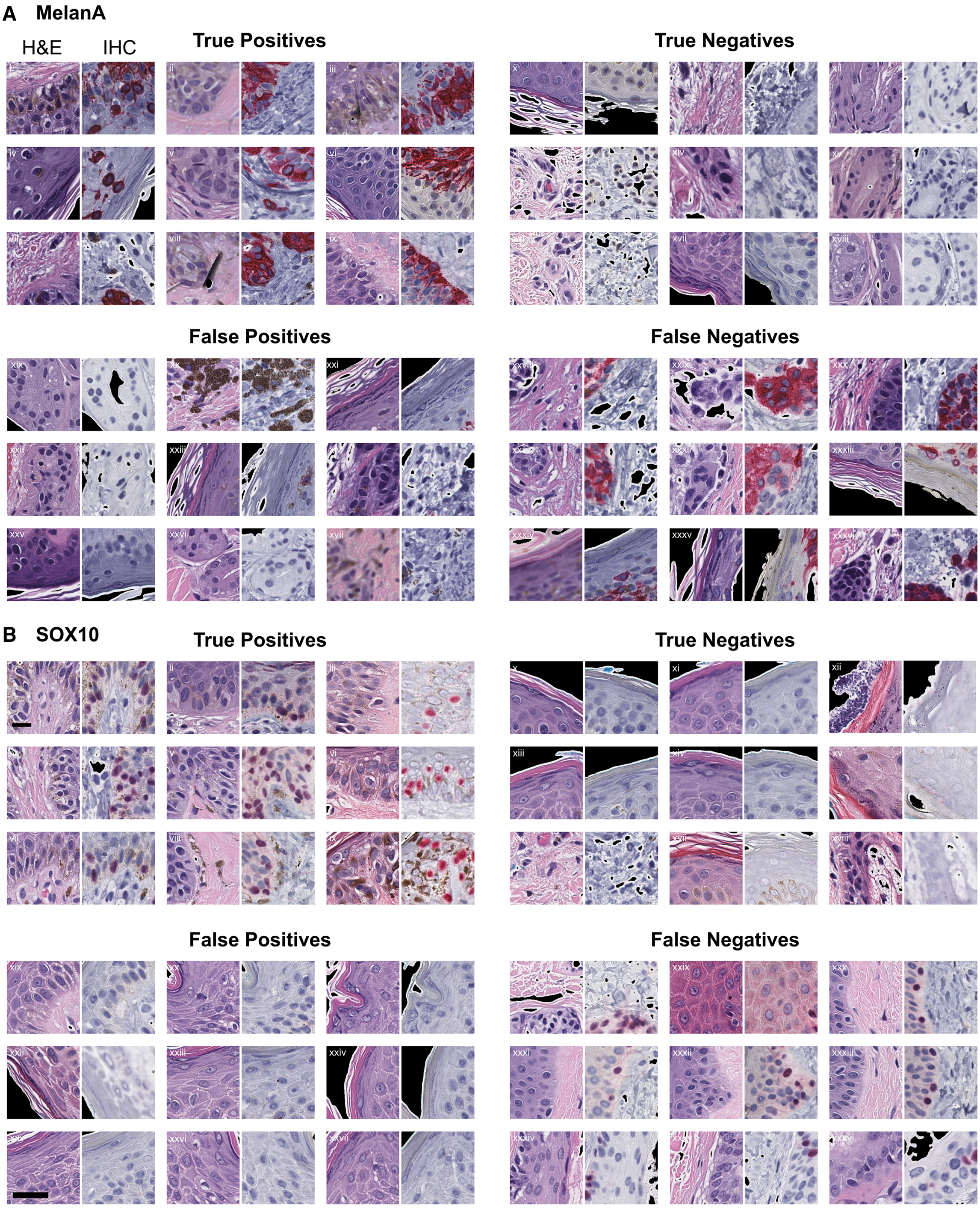

Figure 4. Assessing model accuracy and reliability in identifying melanocytes in validation H&E- and IHC-stained tiles
(A and B) Truth table prediction examples: Predictions of MelanA and SOX10 melanocyte-detectors. Both panels demonstrate the four prediction outcomes: true-positive, true-negative, false-positive, and false-negative. For each outcome category, nine paired examples contain a paired H&E tile and its corresponding IHC tile for the MelanA (A) and SOX10 (B) models. Scale bars, 32 μm.

Figure 4B). However, other false-negative image pairs appear to be cut discontinuously, with either fewer or absent melanocytes seen in the H&E half of the tile pair or a blurry H&E image (false-negative tiles *xxxi*, *xxxii*, *xxxiv*, and *xxxvi* Figure 4B). These discontinuous cases inflate the false-negative count, as the model reliably evaluated the H&E tile. In contrast, the MelanA model, on average, rejects tiles with more melanocytes than the SOX10 tiles, but there appears to be more processing

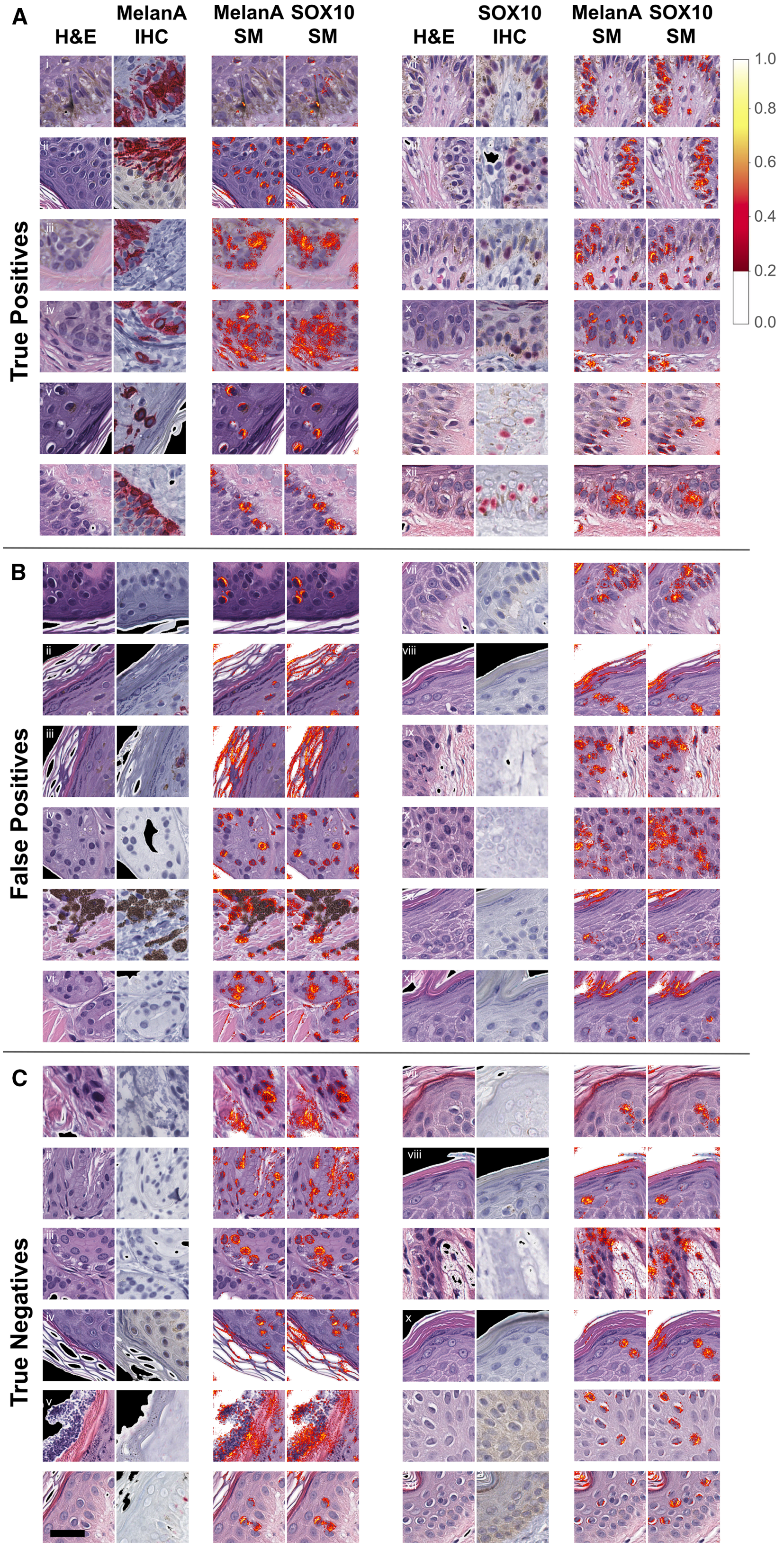

Figure 5. Example saliency maps for MelanA and SOX10 models

(A–C) Example of saliency map on true-positive (A), false-negative tiles (B), and true-negative example tiles (C) using the MelanA and SOX10 models. Scale bars, 32 µm.

CellPress
OPEN ACCESS

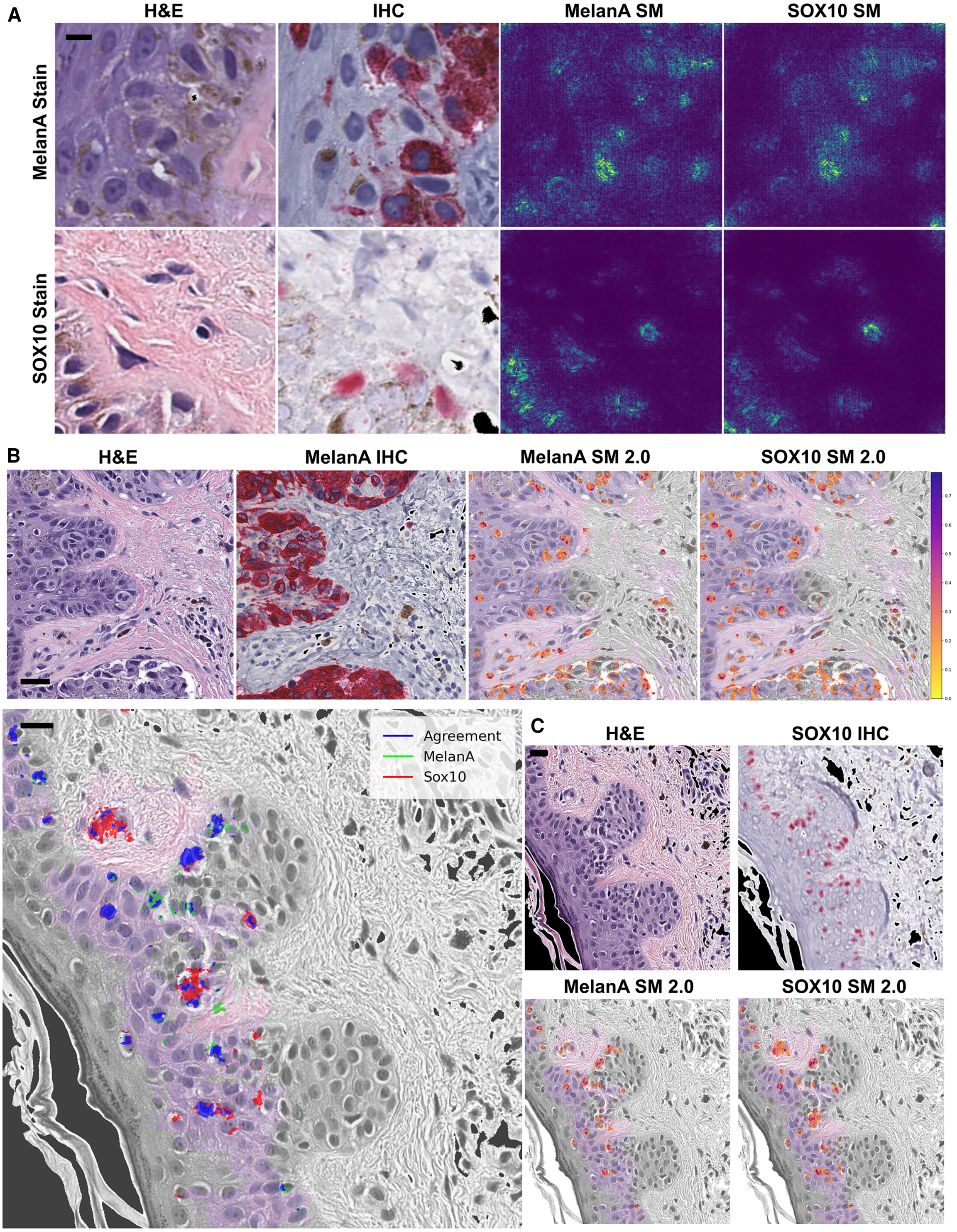

Figure 6. Interpreting model predictions via saliency mapping

(A) Columns display H&E tiles, IHC, MelanA model saliency map attributions, and SOX10 model saliency map attributions. All tiles are size 256 × 256 pixels; scale bars, 8 μm.

(B) A stitched view of attributions across multiple tiles (1,024 × 1,024 pixels) on a MelanA-stained tissue. We used Guided Grad-CAM to calculate attributions and overlaid them onto the portion of the tissue in the image predicted to have melanocytes (the color-saturated area; using prediction heatmap, stride = 6, confidence threshold = 0.9). The desaturated area represents tissue without strong melanocyte prediction. Scale bars, 32 μm.

(legend continued on next page)

artifact within the tissue in these images and non-contiguous section "mismatch" in some cross-tissue tile alignment (e.g., false-negative tiles *xxxi* and *xxxvi* in Figure 4A). Therefore, some false-negative MelanA examples may be misleading.

MelanA and SOX10 models both appeared to select tiles with basketweave orthokeratosis and few to no melanocytes in their false-positives (e.g., false-positive tiles *xxi* and *xxiii* in Figure 4A; false-positive tiles *xxii* and *xxiv* in Figure 4B). Some melanocytic lesions may show pigmented parakeratosis, such as in acral sites or particularly irritated or "traumatized" melanocytic lesions, but basketweave or compact orthokeratosis is not conventionally independently correlated with the presence or absence of melanocytes. Further, the stratum corneum in false-positive tiles without melanocytes appears to lack obvious pigmentation. While the basketweave orthokeratosis could be coincidentally present in these false-positive tiles, its saliency highlighted in Figure 5 argues against mere chance (false-positive tiles *ii*, *iii*, *viii*, *xi*, and *xii* in Figure 5B).

Cases where the SOX10 model falsely identified keratinocytes as melanocytes show H&E tiles with perinuclear clearing of keratinocytes, particularly in cases with angulated and hyperchromatic keratinocytic nuclei (e.g., false-positive tiles *xx* and *xxvi* in Figure 4B). These cases also show epidermal "spongiosis," signifying increased intercellular fluid, creating visible desmosomes between cells and "reactive" keratinocytes. The selection of tiles with keratinocyte desmosomes is a surprising finding, given that melanocytes lack desmosomes. However, these reactive keratinocytes contain more prominent nucleoli than their neighbors, and some cells show nuclear pallor mimicking melanocyte pseudo-inclusions, suggesting the SOX10 model may be prioritizing nuclear features and immediate perinuclear features of the cytoplasm rather than the intercellular bridging seen elsewhere in the image (see saliency in false-positive tiles *vii*, *ix*, and *x* in Figure 5B).

Interpreting the models' rationales for melanocytic morphology

Intuitively, a saliency map illustrates a model's rationale for predicting melanocyte presence or absence in the tiles. Specifically, the maps highlight a tile's most salient features (pixels), whose deletion or intensification would change the model's prediction most. For the clinically minded, these salient features are pertinent positives *and* negatives, with the saliency maps highlighting features in the image that can also explain why something *is not* a melanocyte. Unlike the heatmaps showing the probability of increased melanocytes in tiles (e.g., Figures 2B and 3B), the saliency maps highlight the specific pixels the algorithm uses to decide whether or not increased numbers of melanocytes are present. In some cases, the algorithm identifies features inside a melanocyte as contributory. In other cases, the model identifies important discriminatory features elsewhere in the image, such as within keratinocytes or the stratum corneum.

Saliency mapping techniques calculate a numerical contribution strength, or "attribution," for each pixel (feature) within a model's prediction *context*. Thus, a salient pixel will have a high attribution value by a particular model in the context of a particular prediction. The same pixel may lose salience in the context of a different model (e.g., a model trained on a different IHC stain) or a prediction on an overlapping but different tile. We used Guided Grad-CAM (see STAR Methods) to calculate and visualize attributions on the H&E input tile using SOX10 and MelanA models for true-positive and false-positive outcomes (Figure 5; Saliency Maps, SM). We scaled attribution magnitudes between 0 and 1, with attribution strength proceeding from red (weak, but >0.2) to yellow (strong). For comparison, Figure 6A displays the raw output of Guided Grad-CAM alongside its H&E instead of by overlay (rightmost two columns, titled "SM"). We generated independent saliency maps for the MelanA- and SOX10-trained models (Figure 5, rightmost two columns of each image group). Despite meaningful differences in how we calculated H&E tile labels when training each model, the saliency maps, nonetheless, appear surprisingly similar. The SOX10 model relied on a count of stained positive nuclei within the hidden paired IHC, whereas the MelanA model learned from labels based on the proportion of stained positive cytoplasmic areas. Both models learned to assign attention to similar cell morphologies of the H&E-stained input tissue.

The MelanA model identifies portions of the stratum corneum and dense dermal melanin deposition as salient features, albeit incorrectly in some cases (e.g., tile sets *ii*, *iii*, and *v* in Figure 5B). However, it correctly disregards increased intracytoplasmic melanin pigment in other less densely pigmented cases, instead focusing on the nuclear membrane and perinuclear salient features (e.g., tile sets *vii*–*xii* in Figure 5A). In instances where the models correctly identify a tile as lacking increased melanocytes, it identifies bizarrely enlarged nuclei and correctly identifies keratinocytic intracytoplasmic processes (desmosomes) as specifically contributing to its correct tile interpretation (e.g., tile sets *vii* and *viii* in Figure 5C). Interestingly, both models appropriately identify nuclear membrane and perinuclear features within eccrine glands as salient reasons for the tile *not* containing increased numbers of melanocytes (e.g., tile sets *ii* and *iii* in Figure 5C).

Nuclear and perinuclear features are identifying features of melanocytes in both models

Prediction heatmaps (e.g., Figures 2B and 3B) show areas of increased melanocytes, while saliency maps (e.g., Figures 5 and 6A) show the most essential pixels underlying the predictions. We posited that combining these maps into a single view would help researchers and clinicians visualize neural network predictions without requiring that they accept all model predictions in a "black box" way. To achieve this, we generated an "explained-heatmap" that highlights increased-melanocyte regions (colored areas vs. grayscale) and calls out the pixels motivating

(C) We constructed a multi-antibody "agreement map" view to compare the virtual stains (left), indicating how the rationales of the two models compare. Both models independently assign high attribution to blue pixels, whereas the MelanA-model-only attributions are green and SOX10-model-only attributions are red. We include corresponding H&E, IHC (top right), and separated saliency maps (bottom right) for reference. The desaturated regions again correspond to the region lacking strong SOX10 prediction (using prediction heatmap, stride = 6, confidence threshold = 0.9). Scale bars, 16 μm.

the positive prediction for those regions (orange) (Figure 6B). By conditioning the saliency map (yellow-orange) on positive prediction-only regions, the explained-heatmap focuses on areas and visual morphology reasoning where the model predicts positive outcomes with above 0.9 confidence. Intriguingly, this pixel-level reasoning extracted from the saliency mapping techniques is a by-product of the neural network training process, which never received training labels at a resolution more fine-grained than yes or no labels for the entire 256 × 256-pixel input images of H&E tiles.

On inspection, explained-heatmap views for MelanA- and SOX10-based models appeared unexpectedly similar (e.g., Figures 6A and 5). To distinguish subtle differences between the models trained on labels from these different IHC stains, we also generated a virtual-IHC "agreement map" comparison view (Figure 6B). Green and red overlays call out pixels exclusively salient to MelanA or SOX10 model predictions on the H&E image. Where both models agreed a pixel was salient, the color is blue. Although MelanA is a cytoplasmic stain and SOX10 is a nuclear stain, the models' attributions "converge" on salient features of the nucleus and the nuclear-cytoplasmic interface, as well as immediate perinuclear cytoplasmic features. Although stratum corneum and intercellular features were salient in negative predictions (e.g., Figure 5C), neither category appears to predict the presence of melanocytes in these positive-area examples (Figure 6C). Quantitative analysis showed substantial overlap (Intersection of Union [IoU]: 0.428 ± 0.286 and Dice: 0.540 ± 0.301) between two saliency maps for the region of interest (Figure S6). This pattern of convergence was consistent across the entire 5-fold test set (IoU: 0.565 ± 0.136 and Dice: 0.712 ± 0.114). To contextualize these results, null-baseline experiments using 500 random *mismatched* but melanocyte-positive tile pairs consistently resulted in IoU and Dice scores of 0 ± 0. Additionally, we tested various minimum pixel-area thresholds for detecting convergent blobs and found that the IoU and Dice scores remained stable across a range of threshold values (Figure S6).

DISCUSSION

CNNs trained on a small real-world dataset of approximate and noisy H&E- and IHC-stained archival slide pairs, nonetheless, learned robust and consistent visual reasoning (pixel salience) for melanocyte cell presence. Although the CNN models never received training information more granular than a simple "melanocytic" or "not" label for 64 × 64 micron H&E tiles, they learned to ascribe precise and fine-grained salience to specific cells (Figure 5). In evaluating the CNNs' reasoning for melanocyte-positive area predictions, we found the salient cells were morphologically plausible (Figures 5A, 6A, and 6B). Strikingly, independent CNNs trained on different IHC stains, nonetheless, independently converged on similar visual reasoning (e.g., Figure 6C) despite the stains themselves being cytoplasmic (MelanA) vs. nuclear (SOX10). This similarity may arise partly because the IHC-based training labels are coarse grained (e.g., one binary label per 64 × 64 micron tile), but these labels are not interchangeable, and the models trained on them do not make identical predictions or exhibit identical reasoning. Consequently, the extent to which the IHC-specific models agree on a particular piece of tissue in predictions or calculated reasoning may also reveal a rudimentary measure of certainty.

Using adjacent but inherently different IHC-stained tissue to derive training labels tackles a problem at the heart of supervised learning—that outputs are only as good as their inputs. In pathology and computational models alike, the phrase "garbage in, garbage out" emphasizes poor-quality data, such as from non-representative biopsy sampling, incomplete tissue preservation, or inconsistent labeling,[36,37] hamper diagnostic accuracy. However, the gold standard of expert human annotation to identify melanocytes remains unsatisfactory for precise computational labeling because humans can be inconsistent and lack perfect interobserver consensus even at the expert level.[38–41] These inconsistencies can arise not only from error but also from differing areas of emphasis, expertise,[42] and positive-criteria definitions.[43] Consequently, we attempted to exploit biological multimodality as a surrogate for ground truth, accepting only the slide-wide binary human input that melanocytic atypia existed within a whole slide image. We then used IHC staining to label adjacent H&E sections to train a CNN of pathologist-diagnosed MIS images.

The resulting models learned features indicative of melanocyte presence (Figures 5 and 6) within H&E images and performed well on validation patient samples (Figures 2A and 3A). Models are robust to similar-looking tissue areas that do not contain melanocytic cells (Figure 4) and can use information from different stains in alternative but complementary ways (Figure 6A). We can map our predictions back onto tissue samples to provide an interpretable view for pathologists to consider, and we observe what morphological features trained networks are activated by (Figures 5, 6A, and 6B). Despite the overall small training dataset sizes and the biological differences between the different IHC targets, MelanA (cytoplasmic) and SOX10 (nuclear), the independent CNN models learned remarkably similar pixel-wise rationales for their predictions (Figures 5 and 6C).

While one would hope that two CNN models that are right for the right reasons ought to independently arrive at a convergent underlying visual logic despite learning from semantically different training labels, this has not, to our knowledge, been shown in dermatopathology, or for independent models trained on separate IHC stains. Exacerbating the challenge, relying on real-world "noisy" archival datasets at this scale would seem only to further militate against the models, nonetheless, having arrived at convergent rationales (Figures 6 and S6). Questions like this motivate the explainable AI and interpretability subfields of deep learning, from which the saliency mapping techniques we leverage emerged. Indeed, opening the "black box" of machine and deep learning models using computational interpretability techniques can reveal when models are relying on unconvincing rationales or have fallen prey to shortcut learning.[44] These remain otherwise silent pitfalls in drug discovery and biomedicine.[45–47]

Saliency maps visually illustrate "why" a model predicts there are (or are not) increased numbers of melanocytes in

an H&E input tile. For the clinically minded, these ''salient features'' can be thought of as both pertinent positives *and* pertinent negatives, with some of the features highlighted by the saliency maps representing specific features in the image that explain why an object *is not* a melanocyte. Similarly, pathologists develop a gestalt of when increased melanocytes are likely to be present in the epidermis based on the surrounding architecture, degree of basilar keratinocytic hyperpigmentation or pigmented parakeratosis, and presence or absence of a clinically targeted keratinocytic neoplasm or inflammatory condition. The saliency maps are consistent with the computational models leveraging similar field-of-view-level data when identifying features of the image that appear unrelated to a melanocyte, but this is only a hypothesis without further testing, such as synthetic counterfactual experiments.

The most striking findings of the study were the consistent and convergent nature of the two CNNs' predictions and saliency maps despite the observation that deep learning models can fail to generalize in scientific and medical settings.[45,48] Some areas of diagnostic pathology, such as levels of melanocytic atypia, are not black and white, requiring technical skill and subjective expert opinion. Just as pathologists must make diagnostic decisions in imperfect real-world conditions, these computational models ''learned'' to grapple with imperfect inputs to provide reproducible tile-level melanocyte ''labels'' in various histopathologic digital images over time and across two different institutions. Future studies would ideally expand the models' training with additional levels of atypia, akin to pathology residents learning more subtle disease variations after tackling overtly benign versus malignant examples.

Limitations of the study

Several caveats merit mention. Biologically, eccrine glands challenged both models. In future studies, additional training with a special stain, such as a periodic acid-Schiff stain[49] highlighting secretory granules, may help the models differentiate between eccrine glands and melanocytes. Computationally, compared to some large-scale studies on tens to hundreds of thousands of private multi-tissue slides,[15,50] this melanocytic tissue dataset is small, varied, and visually noisy. It spans two disparate melanocytic IHC stains and medical institutions using different slide scanners and sensors. Indeed, the archival and real-world nature of the slide pairs meant that these biopsy cases were among the most pathologically complex, requiring the additional step of IHC slides for diagnosis. As we drew them from physical archives, the IHC slides were not always cut ''back to back'' and demonstrated a varying degree of fading due to time and storage conditions. We ultimately had to discard half of the digitized slides from model training due to image-quality concerns or the physical tissue sections being too different across the slides for meaningful comparison. Consequently, employing fine-grained deep learning models and strategies such as semantic segmentation (e.g., U-Net[51,52]) or object detection[53,54] was impossible, including intriguing but data-hungry new architectures such as vision transformers.[55]

RESOURCE AVAILABILITY

Lead contact

Requests for further information and resources should be directed to and will be fulfilled by the lead contact, Michael J. Keiser (keiser@keiserlab.org).

Materials availability

This study did not generate new unique reagents.

Data and code availability

- Comprehensive trained neural network model weight data are freely available in Zenodo (https://doi.org/10.5281/zenodo.16938226) and GitHub (http://github.com/keiserlab/dermato-paper). H&E and IHC WSIs were collected from confidential patient samples under IRB approvals that did not grant distribution of clinical-archive medical data.
- The source code for the WSI data preparation and convolutional neural networks, including trained model weights, is freely available under the open-source MIT license at Zenodo (doi: https://doi.org/10.5281/zenodo.16938226) and GitHub (http://github.com/keiserlab/dermato-paper).
- Any additional information required to reanalyze the data reported in this work paper is available from the lead contact upon request.

ACKNOWLEDGMENTS

This work was supported by CZI grant DAF2018-191905 (https://doi.org/10.37921/550142lkcjzw) from the Chan Zuckerberg Initiative DAF, an advised fund of Silicon Valley Community Foundation (funder https://doi.org/10.13039/100014989) (M.J.K.). We thank Dr. Kerri Rieger at Stanford University for her mentorship.

AUTHOR CONTRIBUTIONS

Conceptualization, E.S.K. and M.J.K; methodology, M.T., G.G., S.G., E.S.K., and M.J.K.; software, M.T., G.G., S.G., and N.M.; validation, M.T., S.G., and E.S.K.; formal analysis, M.T., G.G., S.G., and E.S.K.; investigation, M.T., G.G., S.G., and N.M.; resources, E.S.K. and M.J.K.; data curation, M.T., G.G., S.G., N.M., and E.S.K.; writing - original draft: M.T., G.G., S.G., E.S.K., and M.J.K.; writing - review and editing, all; visualization, M.T., G.G., and S.G.; supervision, E.S.K. and M.J.K.; project administration, M.J.K; funding acquisition, M.J.K.

DECLARATION OF INTERESTS

G.G. is now an employee of Genentech. M.T. is now a student at Mount Sinai.

DECLARATION OF GENERATIVE AI AND AI-ASSISTED TECHNOLOGIES IN THE WRITING PROCESS

During the preparation of this work the authors used ChatGPT-4o (https://chat.openai.com, August 2024) to suggest edits for readability, clarity, and conciseness after we had written the manuscript draft. After using this tool/service, the authors reviewed and edited the content as needed and take full responsibility for the content of the publication.

STAR★METHODS

Detailed methods are provided in the online version of this paper and include the following:

- KEY RESOURCES TABLE
- EXPERIMENTAL MODEL AND STUDY PARTICIPANTS DETAILS
  - Ethics approval and consent to participate
  - Sample cohort
- METHOD DETAILS
  - Sample preparation
  - Dataset curation
  - Tissue extraction and filtering

CellPress
OPEN ACCESS

- Image alignment
- Stain-specific labeling
- Convolutional neural network model training and assessment
- Prediction heatmaps and IHC label maps
- Generating saliency and agreement maps

- QUANTIFICATION AND STATISTICAL ANALYSIS

SUPPLEMENTAL INFORMATION

Supplemental information can be found online at https://doi.org/10.1016/j.crmeth.2025.101201.

Received: October 10, 2024
Revised: April 21, 2025
Accepted: September 17, 2025
Published: October 20, 2025

REFERENCES

1. Shen, D., Wu, G., and Suk, H.-I. (2017). Deep Learning in Medical Image Analysis. Annu. Rev. Biomed. Eng. *19*, 221–248. https://doi.org/10.1146/annurev-bioeng-071516-044442.

2. Brody, H. (2013). Medical imaging. Nature *502*, S81. https://doi.org/10.1038/502s81a.

3. Naylor, C.D. (2018). On the Prospects for a (Deep) Learning Health Care System. JAMA *320*, 1099–1100. https://doi.org/10.1001/jama.2018.11103.

4. Cireşan, D.C., Giusti, A., Gambardella, L.M., and Schmidhuber, J. (2013). Mitosis detection in breast cancer histology images with deep neural networks. Med. Image Comput. Comput. Assist. Interv. *16*, 411–418. https://doi.org/10.1007/978-3-642-40763-5_51.

5. Cruz-Roa, A., Basavanhally, A., González, F., Gilmore, H., Feldman, M., Ganesan, S., Shih, N., Tomaszewski, J., and Madabhushi, A. (2014). Automatic detection of invasive ductal carcinoma in whole slide images with convolutional neural networks. In Medical Imaging 2014: Digital Pathology (SPIE), pp. 904103. https://doi.org/10.1117/12.2043872.

6. Ertosun, M.G., and Rubin, D.L. (2015). Automated Grading of Gliomas using Deep Learning in Digital Pathology Images: A modular approach with ensemble of convolutional neural networks. AMIA Annu. Symp. Proc. *2015*, 1899–1908.

7. Litjens, G., Sánchez, C.I., Timofeeva, N., Hermsen, M., Nagtegaal, I., Kovacs, I., Hulsbergen-van de Kaa, C., Bult, P., van Ginneken, B., and van der Laak, J. (2016). Deep learning as a tool for increased accuracy and efficiency of histopathological diagnosis. Sci. Rep. *6*, 26286. https://doi.org/10.1038/srep26286.

8. Wang, H., Cruz-Roa, A., Basavanhally, A., Gilmore, H., Shih, N., Feldman, M., Tomaszewski, J., Gonzalez, F., and Madabhushi, A. (2014). Mitosis detection in breast cancer pathology images by combining handcrafted and convolutional neural network features. J. Med. Imaging *1*, 034003. https://doi.org/10.1117/1.JMI.1.3.034003.

9. Office of the Commissioner (2024). FDA Allows Marketing of First Whole Slide Imaging System for Digital Pathology (U.S. Food and Drug Administration). https://www.fda.gov/news-events/press-announcements/fda-allows-marketing-first-whole-slide-imaging-system-digital-pathology.

10. Salman, N.N. (1990). FDA—Medical device news corner: Outline for the premarket approval (PMA) application. J. Appl. Biomater. *1*, 259–261. https://doi.org/10.1002/jab.770010312.

11. Center for Devices, and Radiological Health (2024). Artificial Intelligence and Machine Learning (AI/ML)-Enabled Medical Devices (U.S. Food and Drug Administration). https://www.fda.gov/medical-devices/software-medical-device-samd/artificial-intelligence-and-machine-learning-aiml-enabled-medical-devices.

12. U.S. Food and Drug Administration (2021). Evaluation of Automatic Class III Designation for Paige Prostate Decision Summary (DEN200080) (Center for Devices and Radiological Health).

13. Krizhevsky, A., Sutskever, I., and Hinton, G.E. (2017). ImageNet classification with deep convolutional neural networks. Commun. ACM *60*, 84–90. https://doi.org/10.1145/3065386.

14. Esteva, A., Robicquet, A., Ramsundar, B., Kuleshov, V., DePristo, M., Chou, K., Cui, C., Corrado, G., Thrun, S., and Dean, J. (2019). A guide to deep learning in healthcare. Nat. Med. *25*, 24–29. https://doi.org/10.1038/s41591-018-0316-z.

15. Campanella, G., Hanna, M.G., Geneslaw, L., Miraflor, A., Werneck Krauss Silva, V., Busam, K.J., Brogi, E., Reuter, V.E., Klimstra, D.S., and Fuchs, T.J. (2019). Clinical-grade computational pathology using weakly supervised deep learning on whole slide images. Nat. Med. *25*, 1301–1309. https://doi.org/10.1038/s41591-019-0508-1.

16. Esteva, A., Kuprel, B., Novoa, R.A., Ko, J., Swetter, S.M., Blau, H.M., and Thrun, S. (2017). Dermatologist-level classification of skin cancer with deep neural networks. Nature *542*, 115–118. https://doi.org/10.1038/nature21056.

17. Liu, Y., Jain, A., Eng, C., Way, D.H., Lee, K., Bui, P., Kanada, K., de Oliveira Marinho, G., Gallegos, J., Gabriele, S., et al. (2020). A deep learning system for differential diagnosis of skin diseases. Nat. Med. *26*, 900–908. https://doi.org/10.1038/s41591-020-0842-3.

18. Wies, C., Schneider, L., Haggenmüller, S., Bucher, T.-C., Hobelsberger, S., Heppt, M.V., Ferrara, G., Krieghoff-Henning, E.I., and Brinker, T.J. (2024). Evaluating deep learning-based melanoma classification using immunohistochemistry and routine histology: A three center study. PLoS One *19*, e0297146. https://doi.org/10.1371/journal.pone.0297146.

19. Han, S.S., Park, G.H., Lim, W., Kim, M.S., Na, J.I., Park, I., and Chang, S.E. (2018). Deep neural networks show an equivalent and often superior performance to dermatologists in onychomycosis diagnosis: Automatic construction of onychomycosis datasets by region-based convolutional deep neural network. PLoS One *13*, e0191493. https://doi.org/10.1371/journal.pone.0191493.

20. Hekler, A., Utikal, J.S., Enk, A.H., Solass, W., Schmitt, M., Klode, J., Schadendorf, D., Sondermann, W., Franklin, C., Bestvater, F., et al. (2019). Deep learning outperformed 11 pathologists in the classification of histopathological melanoma images. Eur. J. Cancer *118*, 91–96. https://doi.org/10.1016/j.ejca.2019.06.012.

21. He, B., Bukhari, S., Fox, E., Abid, A., Shen, J., Kawas, C., Corrada, M., Montine, T., and Zou, J. (2022). AI-enabled in silico immunohistochemical characterization for Alzheimer's disease. Cell Rep. Methods *2*, 100191. https://doi.org/10.1016/j.crmeth.2022.100191.

22. Latonen, L., Koivukoski, S., Khan, U., and Ruusuvuori, P. (2024). Virtual staining for histology by deep learning. Trends Biotechnol. *42*, 1177–1191. https://doi.org/10.1016/j.tibtech.2024.02.009.

23. Jackson, C.R., Sriharan, A., and Vaickus, L.J. (2020). A machine learning algorithm for simulating immunohistochemistry: development of SOX10 virtual IHC and evaluation on primarily melanocytic neoplasms. Mod. Pathol. *33*, 1638–1648. https://doi.org/10.1038/s41379-020-0526-z.

24. Kucharski, D., Kleczek, P., Jaworek-Korjakowska, J., Dyduch, G., and Gorgon, M. (2020). Semi-Supervised Nests of Melanocytes Segmentation Method Using Convolutional Autoencoders. Sensors *20*, 1546. https://doi.org/10.3390/s20061546.

25. Liu, K., Mokhtari, M., Li, B., Nofallah, S., May, C., Chang, O., Knezevich, S., Elmore, J., and Shapiro, L. (2021). Learning Melanocytic Proliferation Segmentation in Histopathology Images from Imperfect Annotations. In 2021 IEEE/CVF Conference on Computer Vision and Pattern Recognition Workshops (CVPRW) (IEEE), pp. 3761–3770. https://doi.org/10.1109/CVPRW53098.2021.00417.

26. Elmore, J.G., Barnhill, R.L., Elder, D.E., Longton, G.M., Pepe, M.S., Reisch, L.M., Carney, P.A., Titus, L.J., Nelson, H.D., Onega, T., et al.

(2017). Pathologists' diagnosis of invasive melanoma and melanocytic proliferations: observer accuracy and reproducibility study. BMJ *357*, j2813. https://doi.org/10.1136/bmj.j2813.

27. Piepkorn, M.W., Longton, G.M., Reisch, L.M., Elder, D.E., Pepe, M.S., Kerr, K.F., Tosteson, A.N.A., Nelson, H.D., Knezevich, S., Radick, A., et al. (2019). Assessment of Second-Opinion Strategies for Diagnoses of Cutaneous Melanocytic Lesions. JAMA Netw. Open *2*, e1912597. https://doi.org/10.1001/jamanetworkopen.2019.12597.
28. Arumi-Uria, M., McNutt, N.S., and Finnerty, B. (2003). Grading of atypia in nevi: correlation with melanoma risk. Mod. Pathol. *16*, 764–771. https://doi.org/10.1097/01.MP.0000082394.91761.E5.
29. Shea, C.R., Vollmer, R.T., and Prieto, V.G. (1999). Correlating architectural disorder and cytologic atypia in Clark (dysplastic) melanocytic nevi. Hum. Pathol. *30*, 500–505. https://doi.org/10.1016/s0046-8177(99)90191-0.
30. Khoury, J.D., Solary, E., Abla, O., Akkari, Y., Alaggio, R., Apperley, J.F., Bejar, R., Berti, E., Busque, L., Chan, J.K.C., et al. (2022). The 5th edition of the World Health Organization Classification of Haematolymphoid Tumours: Myeloid and Histiocytic/Dendritic Neoplasms. Leukemia *36*, 1703–1719. https://doi.org/10.1038/s41375-022-01613-1.
31. Tellez, D., Balkenhol, M., Otte-Holler, I., van de Loo, R., Vogels, R., Bult, P., Wauters, C., Vreuls, W., Mol, S., Karssemeijer, N., et al. (2018). Whole-Slide Mitosis Detection in H&E Breast Histology Using PHH3 as a Reference to Train Distilled Stain-Invariant Convolutional Networks. IEEE Trans. Med. Imaging *37*, 2126–2136. https://doi.org/10.1109/TMI.2018.2820199.
32. Huang, G., Liu, Z., Van Der Maaten, L., and Weinberger, K.Q. (2017). Densely Connected Convolutional Networks. In 2017 IEEE Conference on Computer Vision and Pattern Recognition (CVPR) (IEEE), pp. 2261–2269. https://doi.org/10.1109/CVPR.2017.243.
33. North, J.P., Kageshita, T., Pinkel, D., LeBoit, P.E., and Bastian, B.C. (2008). Distribution and significance of occult intraepidermal tumor cells surrounding primary melanoma. J. Invest. Dermatol. *128*, 2024–2030. https://doi.org/10.1038/jid.2008.41.
34. Arganda-Carreras, I., Sorzano, C.O.S., Marabini, R., Carazo, J.M., Ortiz-de-Solorzano, C., and Kybic, J. (2006). Consistent and elastic registration of histological sections using vector-spline regularization. In Computer Vision Approaches to Medical Image Analysis Lecture notes in computer science (Springer Berlin Heidelberg), pp. 85–95. https://doi.org/10.1007/11889762_8.
35. Elston, D.M., Ferringer, T., Ko, C., High, W.A., and DiCaudo, D.J. (2024). Dermatopathology (Elsevier Health Sciences).
36. Raykar, V.C., Yu, S., Zhao, L.H., Jerebko, A., Florin, C., Valadez, G.H., Bogoni, L., and Moy, L. (2009). Supervised learning from multiple experts: whom to trust when everyone lies a bit. In Proceedings of the 26th Annual International Conference on Machine Learning (ACM), pp. 889–896. https://doi.org/10.1145/1553374.1553488.
37. Smyth, P., Fayyad, U., Burl, M., Perona, P., and Baldi, P. (1994). Inferring ground truth from subjective labelling of Venus images. Neural Inf Process Syst, 1085–1092.
38. Murphy, M., Loosemore, A., Ferrer, I., Wesseling, P., Wilkins, P.R., and Bell, B.A. (2002). Neuropathological diagnostic accuracy. Br. J. Neurosurg. *16*, 461–464. https://doi.org/10.1080/0268869021000030267.
39. Sarwar, S., Dent, A., Faust, K., Richer, M., Djuric, U., Van Ommeren, R., and Diamandis, P. (2019). Physician perspectives on integration of artificial intelligence into diagnostic pathology. npj Digit. Med. *2*, 28. https://doi.org/10.1038/s41746-019-0106-0.
40. Khullar, D., Jha, A.K., and Jena, A.B. (2015). Reducing Diagnostic Errors–Why Now? N. Engl. J. Med. *373*, 2491–2493. https://doi.org/10.1056/NEJMp1508044.
41. Graber, M.L. (2013). The incidence of diagnostic error in medicine. BMJ Qual. Saf. *22*, ii21–ii27. https://doi.org/10.1136/bmjqs-2012-001615.
42. Yan, Y., Rosales, R., Fung, G., Subramanian, R., and Dy, J. (2014). Learning from multiple annotators with varying expertise. Mach. Learn. *95*, 291–327. https://doi.org/10.1007/s10994-013-5412-1.
43. Wong, D.R., Tang, Z., Mew, N.C., Das, S., Athey, J., McAleese, K.E., Kofler, J.K., Flanagan, M.E., Borys, E., White, C.L., 3rd., et al. (2022). Deep learning from multiple experts improves identification of amyloid neuropathologies. Acta Neuropathol. Commun. *10*, 66. https://doi.org/10.1186/s40478-022-01365-0.
44. Geirhos, R., Jacobsen, J.-H., Michaelis, C., Zemel, R., Brendel, W., Bethge, M., and Wichmann, F.A. (2020). Shortcut learning in deep neural networks. Nat. Mach. Intell. *2*, 665–673. https://doi.org/10.1038/s42256-020-00257-z.
45. Chuang, K.V., and Keiser, M.J. (2018). Adversarial Controls for Scientific Machine Learning. ACS Chem. Biol. *13*, 2819–2821. https://doi.org/10.1021/acschembio.8b00881.
46. Young, A.T., Fernandez, K., Pfau, J., Reddy, R., Cao, N.A., von Franque, M.Y., Johal, A., Wu, B.V., Wu, R.R., Chen, J.Y., et al. (2021). Stress testing reveals gaps in clinic readiness of image-based diagnostic artificial intelligence models. npj Digit. Med. *4*, 10. https://doi.org/10.1038/s41746-020-00380-6.
47. Chuang, K.V., and Keiser, M.J. (2018). Comment on "Predicting reaction performance in C–N cross-coupling using machine learning.". Science *362*, eaat8603. https://doi.org/10.1126/science.aat8603.
48. Futoma, J., Simons, M., Panch, T., Doshi-Velez, F., and Celi, L.A. (2020). The myth of generalisability in clinical research and machine learning in health care. Lancet Digit. Health *2*, e489–e492. https://doi.org/10.1016/S2589-7500(20)30186-2.
49. PAS (Periodic acid-Schiff) https://www.pathologyoutlines.com/topic/stainspas.html
50. Chen, R.J., Ding, T., Lu, M.Y., Williamson, D.F.K., Jaume, G., Song, A.H., Chen, B., Zhang, A., Shao, D., Shaban, M., et al. (2024). Towards a general-purpose foundation model for computational pathology. Nat. Med. *30*, 850–862. https://doi.org/10.1038/s41591-024-02857-3.
51. Ronneberger, O., Fischer, P., and Brox, T. (2015). U-Net: Convolutional Networks for Biomedical Image Segmentation. Preprint at arXiv. https://doi.org/10.48550/ARXIV.1505.04597.
52. Minaee, S., Boykov, Y., Porikli, F., Plaza, A., Kehtarnavaz, N., and Terzopoulos, D. (2022). Image Segmentation Using Deep Learning: A Survey. IEEE Trans. Pattern Anal. Mach. Intell. *44*, 3523–3542. https://doi.org/10.1109/TPAMI.2021.3059968.
53. Redmon, J., Divvala, S., Girshick, R., and Farhadi, A. (2015). You Only Look Once: Unified, Real-Time Object Detection. Preprint at arXiv. https://doi.org/10.48550/ARXIV.1506.02640.
54. Zhao, Z.-Q., Zheng, P., Xu, S.-T., and Wu, X. (2019). Object Detection With Deep Learning: A Review. IEEE Trans. Neural Netw. Learn. Syst. *30*, 3212–3232. https://doi.org/10.1109/TNNLS.2018.2876865.
55. Dosovitskiy, A., Beyer, L., Kolesnikov, A., Weissenborn, D., Zhai, X., Unterthiner, T., Dehghani, M., Minderer, M., Heigold, G., Gelly, S., et al. (2020). An Image is Worth 16x16 Words: Transformers for Image Recognition at Scale. Preprint at arXiv. https://doi.org/10.48550/ARXIV.2010.11929.
56. pyvips: python binding for libvips using cffi (Github). https://pypi.org/project/pyvips/.
57. OpenCV - open Computer Vision library (2021). OpenCV. https://opencv.org/.
58. ipywidgets: Interactive Widgets for the Jupyter Notebook (Github). https://ipywidgets.readthedocs.io
59. Paszke, A., Gross, S., Massa, F., Lerer, A., Bradbury, J., Chanan, G., Killeen, T., Lin, Z., Gimelshein, N., Antiga, L., et al. (2019). PyTorch: An imperative style, high-performance deep learning library. Preprint at arXiv. https://doi.org/10.48550/arXiv.1912.01703.
60. Captum · Model Interpretability for PyTorch https://captum.ai.

CellPress
OPEN ACCESS

61. Selvaraju, R.R., Cogswell, M., Das, A., Vedantam, R., Parikh, D., and Batra, D. (2016). Grad-CAM: Visual Explanations from Deep Networks via Gradient-based Localization. Preprint at arXiv. https://doi.org/10.48550/ARXIV.1610.02391.

62. Sundararajan, M., Taly, A., and Yan, Q. (2017). Axiomatic Attribution for Deep Networks. Preprint at arXiv. https://doi.org/10.48550/ARXIV.1703.01365.

63. Gupta, A., and Arora, S. (2019). A Simple Saliency Method That Passes the Sanity Checks. Preprint at arXiv. https://doi.org/10.48550/ARXIV.1905.12152.

64. The Cancer Genome Atlas program (TCGA) (2022). https://www.cancer.gov/ccg/research/genome-sequencing/tcga

65. HistomicsTK. A Python toolkit for pathology image analysis algorithms (Github). https://digitalslidearchive.github.io/HistomicsTK/.

STAR★METHODS

KEY RESOURCES TABLE

REAGENT or RESOURCE	SOURCE	IDENTIFIER
**Antibodies**		
SOX10 (UCSF)	Biocare Medical	Sox10 (M), Predilute, 25 mL; API3099H; RRID: AB_2861289
SOX10 (Stanford)	Ventana predilute	clone SP267, cat# 760-4968, RRID: AB_2894718
MelanA/MART1 (UCSF)	Agilent Technologies	FLEX Mono Mo a Hu Melan-A, cl A103, RTU; IR63361-2
MelanA/MART1 (Stanford)	Ventana predilute	CONFIRM MART-1/melan A (A103) Primary An; clone A103, cat# 790-2990; Roche cat# 05278350001
MelPro (UCSF)	Biocare Medical	Pan Melanoma + Ki67, Predilute 25ML; PM362DSH; RRID: AB_10581494
MelPro (Stanford)	Ventana predilute<br>Dako/Agilent concentrate	Melanoma Triple Cocktail; clone HMB45 + A103 + T311, cat# 790-4677<br>clone MIB-1, cat# M724001-2; Roche cat# 06527787001
**Deposited data**		
PyTorch, Trained convolutional neural network model weight data	This paper	https://doi.org/10.5281/zenodo.16938226 (http://github.com/keiserlab/dermato-paper)
**Software and algorithms**		
DenseNet121 convolutional neural network (CNN) model	Huang et al.[32]	https://doi.org/10.1109/CVOR.2017.243
Python, custom package (Convolutional neural network code, scripts, and example outputs for melanocytic cell prediction)	This paper	https://doi.org/10.5281/zenodo.16938226 (http://github.com/keiserlab/dermato-paper)
Pyvips, open source library	Python binding for libvips using cffi[56]	https://github.com/libvips/pyvips
OpenCV, open source library	Open Computer Vision library (2021)[57]	https://opencv.org/
ipywidgets, open source library	Interactive Widgets for the Jupyter Notebook[58]	https://github.com/jupyter-widgets/ipywidgets
bUnwarpJ, open source alignment algorithm	Arganda-Carreras et al.[34]	https://imagej.net/plugins/bunwarpj/#references-citation
PyTorch library	Paszke et al.[59]	https://pytorch.org/
Captum library	Model Interpretability for PyTorch[60]	https://captum.ai
Guided Grad-CAM algorithm	Selvaraju et al.[61]	https://doi.org/10.48550/ARXIV.1610.02391
Integrated gradients algorithm	Sundararajan et al.[62]<br>Gupta and Arora[63]	https://doi.org/10.48550/ARXIV.1703.01365<br>https://doi.org/10.48550/arXiv.1905.12152
**Other**		
Clinical Melanoma WSIs	UCSF clinical archives, 2011-2015	N/A
Clinical Melanoma WSIs	Stanford clinical archives, 2011-2015	N/A
TCGA WSIs, non-cutaneous	The Cancer Genome Atlas program (2022)[64]	https://www.cancer.gov/ccg/research/genome-sequencing/tcga

EXPERIMENTAL MODEL AND STUDY PARTICIPANTS DETAILS

Ethics approval and consent to participate

We obtained all materials through pre-approved teaching slides from UCSF's dermatopathology program or from patients who gave informed consent to distribute and use their samples at UCSF or Stanford. The Institutional Review Board (IRB) at the University of

CellPress
OPEN ACCESS

California, San Francisco, and Stanford University oversaw approval. Access to data followed current laws, regulations, and IRB guidelines. WSI patient samples were de-identified and did not contain personal health information.

Sample cohort

We generated digital pathology images from archival patient H&E and IHC stained slides of diagnosed melanoma *in situ* from either UCSF's Dermatopathology Service hospital cases or Stanford's Dermatopathology Service hospital cases. All samples were gathered between 2011 and 2015, with patients aged between 43 and 73. Each patient sample comprised a pair of WSIs: a WSI of H&E-stained tissue sections and a WSI of corresponding IHC-stained sections. At the original diagnosis, pathologists extracted and digitally scanned all available slides from various biopsies, including/incisional, shave, and punch biopsies. However, only H&E and IHC WSI biopsy pairs optimized for nearness based on alignment were included in the study.

METHOD DETAILS

Sample preparation

Each cutaneous biopsy or excisional tissue underwent standard processing for H&E and IHC stained sections at time of original histopathologic diagnosis. For H&E staining, patient samples were formalin-fixed, sectioned, paraffin-embedded, cut and placed on a glass slide, and stained with hematoxylin and eosin (H&E). For IHC staining, tissue samples from the same paraffin blocks were similarly prepared but treated with one of three antibodies to highlight melanocytes: MelanA, SOX10, and MelPro (dual MelanA and Ki-67 stain). Some pairs were cut in exact serial tissue sections, and others were cut on a separate day after the tissue block was refaced at the discretion of the original diagnostic pathologist. We scanned each clinical case's original diagnostic H&E and IHC glass slides to generate the WSI pairs. We digitized whole slide images at 40× magnification, corresponding to a resolution of 0.25 microns per pixel (MPP), using a Philips slide scanner (Philips Ultra Fast Scanner Research Use Only/UFS1.6 RUO) or an Aperio scanscope (AxioVision; Leica Biosystems) and stored them as compressed pyramidal image files (BigTIFF and SVS images).

Dataset curation

Of an original dataset of 242 newly scanned cases of melanoma, we selected 61 WSI pairs (122 WSIs) from the UCSF and Stanford clinical archives because these 61 cases had an accompanying IHC stain. Each pair comprises a WSI containing an H&E-stained slide and an independent but complementary WSI IHC-stained slide. Of 61 candidate pairs, we removed 38 pairs by manual inspection due to poor data quality, such as folded, damaged, or blurry tissue or irreconcilable size differences between H&E and IHC stained tissue sections. The final dataset consisted of 23 patient WSI pairs (46 WSIs), including 10 SOX10 stains, 10 MelanA stains (also known as MART1), and 3 MelPro stains (Data S1; Tables S1 and S2 detail dataset composition). We created two datasets by grouping patient pairs across institutions (UCSF: 14/23 samples; Stanford: 9/23 samples) by their IHC stains: one comprising WSIs with either MelanA or MelPro IHCs (combined: 13/23 WSIs), named the "MelanA" dataset, and a "SOX10" dataset (10/23 WSIs). SOX10, or SRY related HMG box 10 protein, is a nuclear immunohistochemical (IHC) stain, MelanA, also known as MART1 (Melanoma Antigen Recognized by T cells 1), is a cytoplasmic immunohistochemical stain, and MelPro is a double-stain that highlights mitotically active nuclei (Ki67 stain) within the cytoplasmic-stained (MelanA stain) melanocyte population, with brown chromogen (nuclear) and red chromogen (cytoplasmic), respectively.

For morphologically diverse negative training examples, we randomly collected 45 non-cutaneous H&E tissue samples from a subset of TCGA to create 135,000 negative labeled tiles[64] (Table S3). These negative controls focused on pancreatic, breast, and lung carcinomas (Table S3). The rationale for adding diverse negative H&E examples was to improve model generalization and ensure it does not simply learn to over-interpret hematoxylin as a surrogate for melanocytes. We assessed this procedure against models entirely lacking TCGA training examples and those with half as many TCGA-derived negative tiles, to verify that the non-cutaneous H&E examples did not reduce the model's performance (Figures S1A and S1B).

Tissue extraction and filtering

To automate tissue extraction, we developed a custom toolkit in Python (Figure S1C). The toolkit first identified the HSV color space of a WSI's background. Using this information, it selected candidate tissue foreground regions that met a size requirement. Thresholds for chroma, size, and degree of region-enclosing were manually adjustable, allowing for optimal tissue extraction. After the user refined the H&E and IHC WSI tissue regions, the toolkit matched tissue section pairs. Matching occurred automatically based on tissue locations in the WSI and could be manually adjusted by the user if necessary. Cases where the matched H&E or IHC section had digital artifacts, excessive tissue damage, was missing, was too small, or was indistinguishable from the background were filtered out. The toolkit then individually cropped and masked the background of successfully extracted tissue sections. The toolkit employed the open-source libraries pyvips,[56] OpenCV,[57] and IpyWidgets.[58]

Image alignment

We used archival IHC-stained tissue sections as proxy melanocyte labels for matched H&E sections. This method required that adjacent sections were morphologically similar and that a proxy label from one section relayed information about the other. Further, at the local level, the density of IHC staining for melanocytes was correlated with the probability of melanocytes being present within the

same region of the adjacent H&E slice. As models operated on high-magnification H&E inputs at the 256 × 256 pixels level, alignment between H&E and IHC tissue sections was crucial.

We align matched H&E and IHC tissue sections at the section-pair level, at native resolution (40× magnification; 0.25 MPP), using the open-source alignment algorithm bUnwarpJ[34] (Figure 1A). In brief, as calculated by an energy function, the algorithm registered image pairs using 2D elastic deformations (B-splines) to minimize differences. Matching tissue sections often varied in shape and size after extraction from WSIs. The algorithm zero-padded (i.e., fills in gaps with black pixels) the smallest section from each matched pair to the size of the larger section before alignment. Additionally, we cropped sections otherwise too large to align (>5GB) to create two smaller pairs instead of one large pair. We manually inspected alignments, scored them, and considered coefficients returned from the alignment algorithm. Alignment coefficients and manual inspection scores were similar (Table S2). We discarded candidate pairs that were unalignable or received a low alignment score.

Stain-specific labeling

We used multiple IHC stains that operated as different melanocytic biomarkers: nuclear and cytoplasmic. We used red and brown chromogen SOX10 and red chromogen MelanA and MelPro stains. Using computational color deconvolution, we converted red-green-blue (RGB) IHC-stained images into an intermediary hematoxylin-eosin-diaminobenzidine (HED) color space. The brown color of 3,3′-diaminobenzidine (DAB) denoted antibody expression for MelanA, SOX10, or MelPro.

Before isolating the DAB, we removed major artifacts, such as blue or green ink and tissue areas containing hemorrhage (Figure S2, ink example). Otherwise, artifacts exhibited strong "false positive" DAB signal indistinguishable from true melanocyte expression. We applied a sequence of Otsu thresholding steps to segment candidate artifact regions[65] and removed ink features using predefined hue-saturation-value (HSV) color exclusion ranges. Furthermore, we manually corrected mislabeled non-melanocyte regions identified as melanocytes (Figure S2).

Following artifact removal, we separated DAB signal from the IHC stain images to extract nuclei for SOX10 and cytoplasmic pixels for MelanA stains. We kept the resulting images at 40× magnification. We generated a training dataset for the models by partitioning the images into 256 × 256 pixels tiles, with a stride (overlap) of 128 pixels for training and 256 pixels for validation and test data.

We used positive-signal threshold calculations specific to the melanocytic biomarker type (Figure 1B; see GitHub open code repository). For SOX10, we labeled H&E tiles positive if their matched IHC tile contained more than two discrete DAB-positive nuclei. For MelanA, we required that DAB-positive pixels comprised more than 3% of the tile area. All H&E tiles failing these criteria received a melanocyte-negative training label. We established thresholds by a board-certified dermatopathologist (ESK) manually inspecting the tiles and their labels across a range of potential values (Figure S3). We assessed the effect of a range of training-label threshold choices on model performance (Figure S3). In an inherent biological limitation, despite their widespread diagnostic use, none of the IHC stains are exclusive to melanocytic cells. For instance, SOX10 also stains dermal nerve bundles and eccrine glands, but it is considered specific for melanocytes within the epidermis.

Convolutional neural network model training and assessment

As the archival slides originally served clinical diagnostic needs, generating high-magnification and relevant paired WSI training data post hoc often raised challenges. We consequently had to discard a substantial portion of the dataset during preprocessing, necessitating operating in a low-data regime for deep learning. Consequently, we adopted a training and evaluation strategy motivated by 5-fold cross-validation, with splits into 60% training, 20% validation, and 20% testing data (tiles, stratified by patient case) for each fold.

We injected TCGA negative tiles into training (70%) and validation (30%) batches, using a batch size of 240 tiles (Table S4). We normalized H&E image pixel input ranges by subtracting their mean value and dividing by the standard deviation of the training set. We trained independent stain-specific CNNs using a DenseNet121 architecture with early stopping using a patience of 8 epochs. We used a stochastic gradient descent (SGD) optimizer with a learning rate of 1e-4 after a grid search hyperparameter optimization that explored combinations of optimizer = {SGD, Adam} and learning rate = {1e-5, 3e-5, 1e-4, 3e-4, 1e-3, 3e-3}. We assessed performance by the CNNs' ability to predict H&E tile labels derived from their unseen IHC counterparts (see "stain-specific labeling" above). We measured the AUROC and the AURPC for each model on its representative test hold-out set per fold and reported the all-fold averages as SOX10 and MelanA CNN performance.

Prediction heatmaps and IHC label maps

We generated prediction heatmaps to visually summarize the CNN's confidence of melanocyte presence across entire H&E tissue sections using a custom multiprocessing script (see GitHub repository) with PyTorch.[59] We generated 256 × 256 tiles centered on every 6th pixel (i.e., stride = 6). We zero-padded tiles to reach the appropriate size, when needed, at the edges of the image. The CNN calculates a prediction score on each tile independently. We converted and normalized prediction scores to RGB values in the matplotlib "viridis" color space and visualized them over the original H&E image using Python.

By contrast, IHC label maps illustrate the "best case" visualization of melanocytic-positive labels on tissue sections, accounting for the visual smoothing that inherently arises from 256 × 256 pixels (0.25 MPP) tiles when displayed at the same 6-pixel stride. These maps derive directly from the hidden DAB-based labels (see "stain-specific labeling" above) and represent what a "perfect" prediction heatmap could achieve. Generating label maps follows a similar process to prediction heatmaps, except that instead of using the

trained CNN to get a prediction based on the H&E, the labeling method highlights the tile-smoothed actual DAB-positive melanocyte area (yellow overlay) on the IHC images.

Generating saliency and agreement maps

Saliency mapping methods highlight the cytologic and architectural pixel features a CNN finds important within an H&E input image. To examine which pixels in an H&E tissue section achieve the highest attribution values numerically, we used the Captum library[60] to apply guided gradient-weighted class activation mapping (Guided Grad-CAM[61]) and integrated gradients[62,63] on all convolutional layers of DensetNet121.[32] We also used the Captum library's "NoiseTunnel" to generate five samples of the input tile with Gaussian noise to allow for more stable attributions. We used Guided Grad-CAM's attributions in Figures 5 and 6 because they were more visually distinct than those from integrated gradients (Figure S5).

After generating attributions for each convolutional layer of the model, we averaged these and overlaid them on the H&E images using the "viridis" colormap (Figure 6A) and custom versions of the reversed "plasma" colormap for the "explained-heatmap" overlays (Figures 6B and 6C, right). The "agreement map" (Figure 6C, left) used a custom colormap with solid colors assigned to the Agreement, MelanA, and SOX10 categories. Figure 6B and 6c′s H&E color signal is also conditioned on the models' positive predictive signal (model confidence threshold = 0.9). In other words, grayed areas are not predicted to have MelanA or Sox10 signal, per their original matched stain. The "explained-heatmap" and "agreement maps" are stitched views 1024 $\times$ 1024 pixels or 4 $\times$ 4 tiles in size. Because attribution values can vary widely between tiles for differing models, we employed Captum's attribute normalization feature for consistent comparison. We also applied Gaussian smoothing to remove edge effects between tiles. We used Python matplotlib, numpy, and OpenCV libraries to generate the images.

We quantified the "agreement maps" and overall test set cross-stain "blob" (object) saliency statistics by computing pixel-wise similarity metrics per shared blob using the Intersection of Union (IoU) and the Dice coefficient scores between the two saliency maps on the region of interest (Figure S6A) and across the test set tiles (Figure S6B). We quantified saliency map overlap at the blob level across the test dataset by: (1) Generating GradCAM attributions of the MelanA/MelPro and SOX10 models averaged across each layer of the model for each qualifying tile in the dataset, (2) performing blob detection (requiring $\geq$100 pixels area) on each attribution map using morphological opening and closing operations, (3) identifying cross-stain blobs, and (4) calculating Intersection over Union (IoU) and Dice scores on them. Qualifying tiles were those with predicted confidence >0.9 by either the MelanA/MelPro or SOX10 model. 41 tiles met these conditions in the test set (Figure 6). The test-set calculation differed from a similar calculation on Figure 6C's 4 $\times$ 4-tile stitched map visualization (Figure 6), which correspondingly stitched together neighboring tiles' attributions before checking for the cross-stain blobs.

QUANTIFICATION AND STATISTICAL ANALYSIS

We trained and evaluated the models using 5-fold cross-validation. For each fold, we used 20% of the data as the test set with the splits stratified by patient. We optimized model parameters via hyperparameter grid search. To evaluate performance, we calculated the average AUROC and the average AUPRC across the 5 test sets. We conducted all analyses in Python. See the method details section for additional analysis details.

Cell Reports Methods, Volume *5*

Supplemental information

Deep learning finds convergent melanocytic morphology despite noisy archival slides

Mikio Tada, Garrett Gaskins, Sina Ghandian, Nicholas Mew, Michael James Keiser, and Elizabeth Sarah Keiser

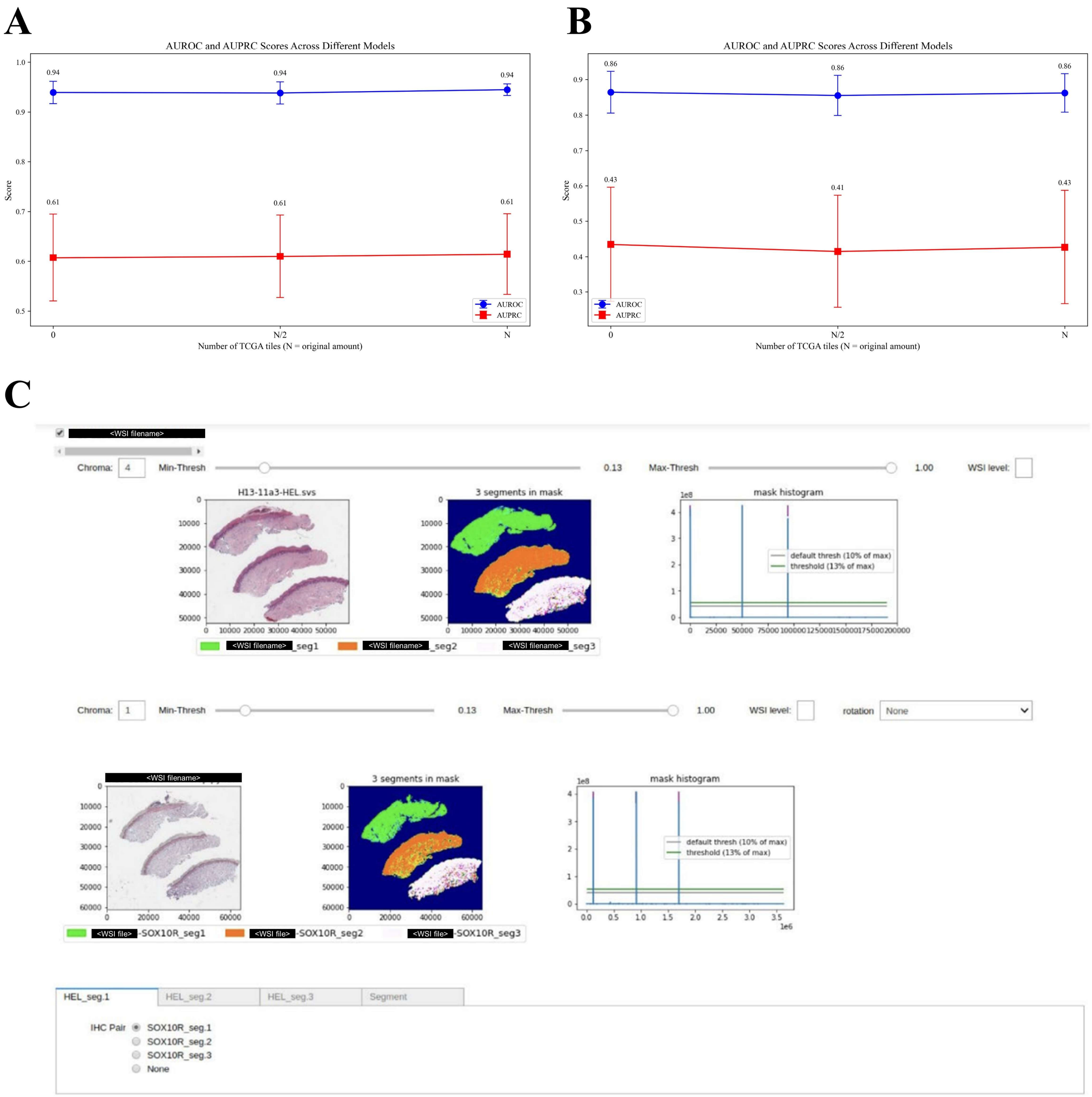

Figure S1. Impact of non-cutaneous TCGA tiles on model performance and automated tissue extraction toolkit, related to Figure 1 and STAR Methods. A) AUROC and AUPRC scores for MelanA models trained on varying amounts of TCGA tiles (0, N/2, N). In all cases, n = 5 folds. B) AUROC and AUPRC scores for SOX10 models trained on varying amounts of TCGA tiles (0, N/2, N). n = 5 folds. C) An example of a sample pair extracted using the automated toolkit. The toolkit visualizes both the H&E (top, left) and IHC (middle, left) whole slide images (WSIs) and extracts tissue regions according to threshold values set for each of the ipywidgets provided. Matching occurs automatically based on tissue locations in the WSI; the user can manually adjust it if necessary (bottom).

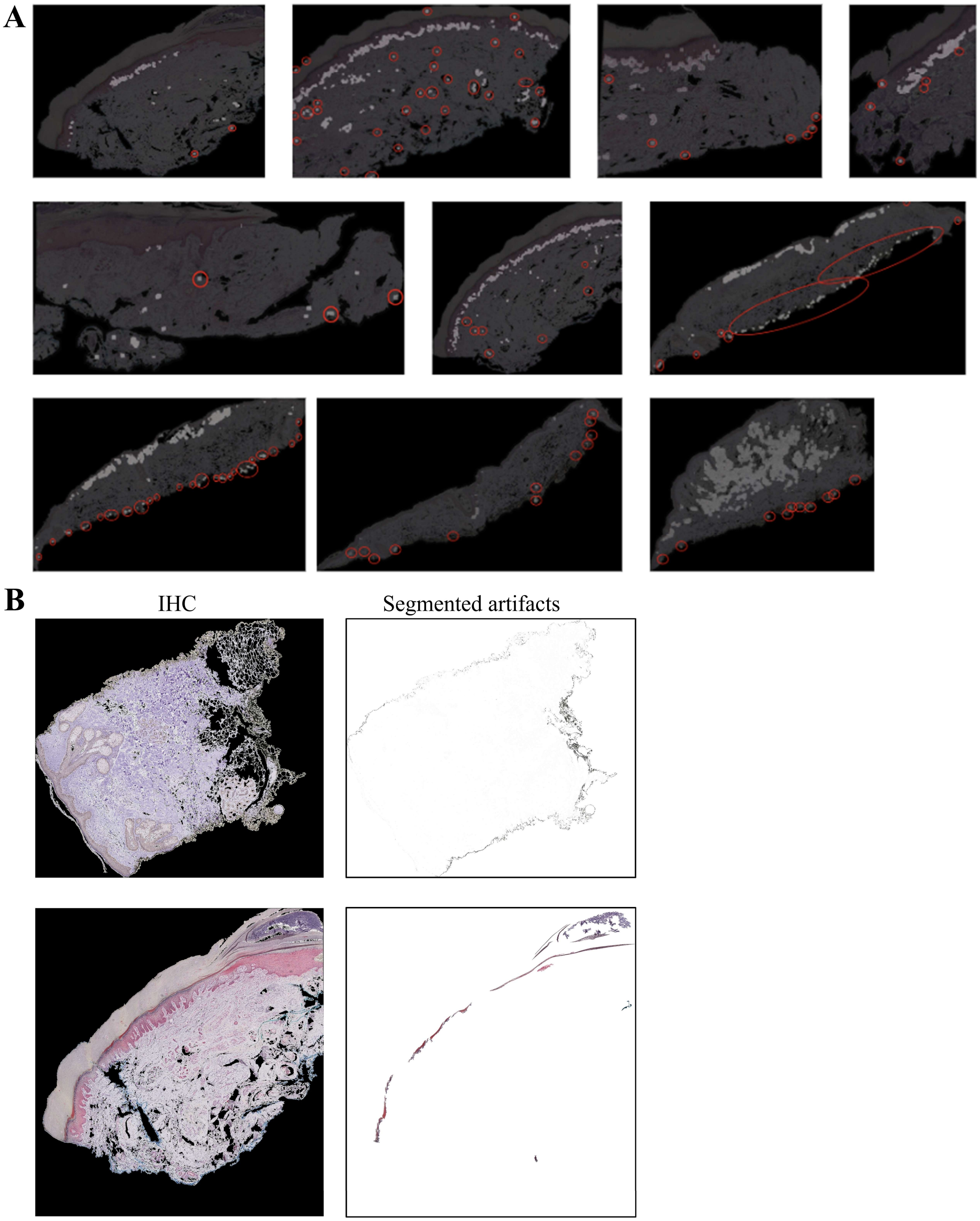

Figure S2. Examples of tissue artifacts computationally and manually removed from IHC WSIs, related to STAR Methods. A) Tissue artifacts that were computationally removed. The top row shows ink, and the bottom shows blood. B) Manual label correction by removal of false-positive areas. The labeling pipeline sometimes misidentified non-melanocyte regions as melanocytes due to tissue artifacts. To ensure label accuracy, we manually corrected these errors (red circles).

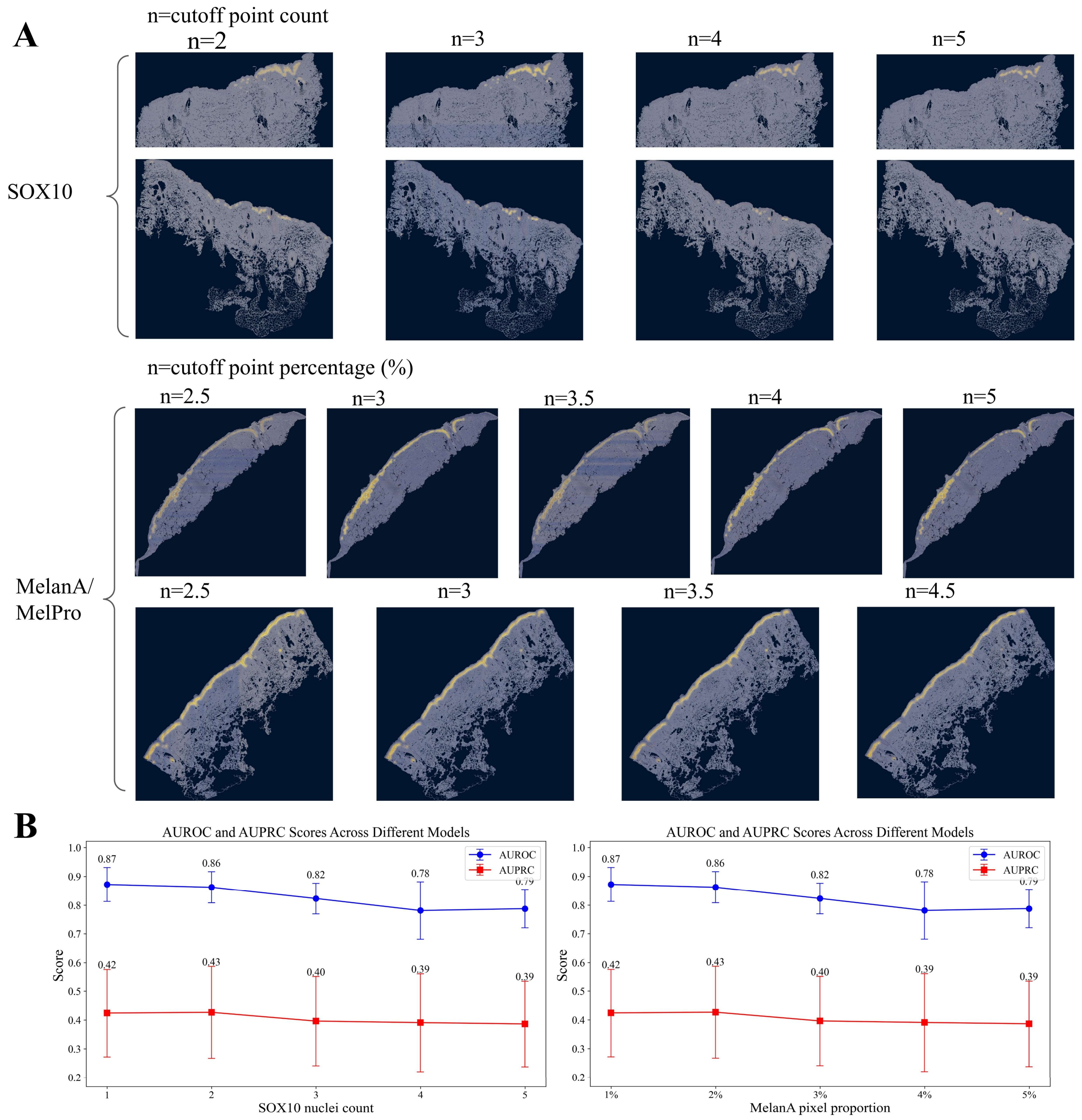

Figure S3. The effect of varying minimum signal thresholds on IHC "label maps" and model performance, related to Figures 2 and 3 and STAR Methods. A) "Label maps" using progressively increasing (more stringent) minimum-signal thresholds, *n*, for two SOX10 (top row) and two MelanA/MelPro (bottom row) WSIs. Label maps derive directly from positive IHC stain but are coarser-grained than the stain itself due to the 256x256 pixel tiles. B) Model performance evaluation across different training labels. AUROC and AUPRC scores for models trained with different SOX10 nuclei count thresholds (1-5) evaluated on the standard Test dataset (threshold=2 nuclei). *n*= 5 folds (left). AUROC and AUPRC scores for models trained on varying MelanA pixel proportion thresholds (1-5%) evaluated in all cases on the standard Test dataset (threshold=3%). *n* = 5 folds (right).

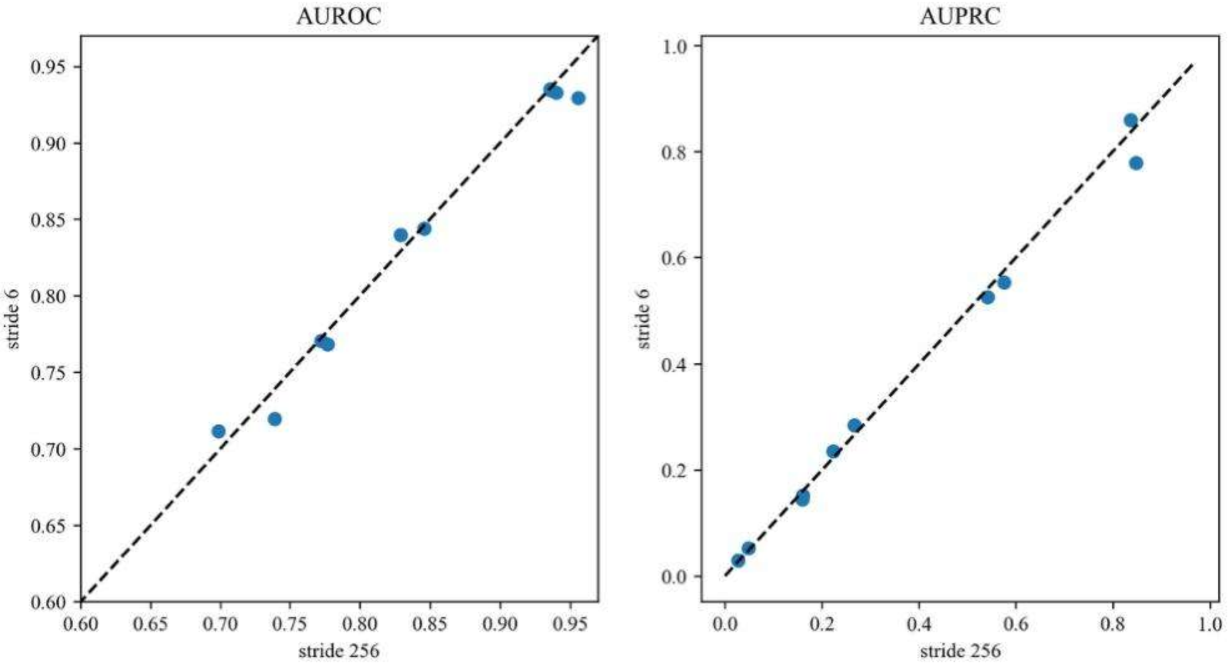

Figure S4. Comparison of performance calculated by AUROC score using stride 6-pixel (y-axis) versus 256-pixel (x-axis) strides on two test sets, related to STAR Methods. Each blue dot represents a tissue section. Stride 256-pixel calculations are much faster to compute due to the n^2 scaling of the heatmap calculation (where n is proportional to 1/stride).

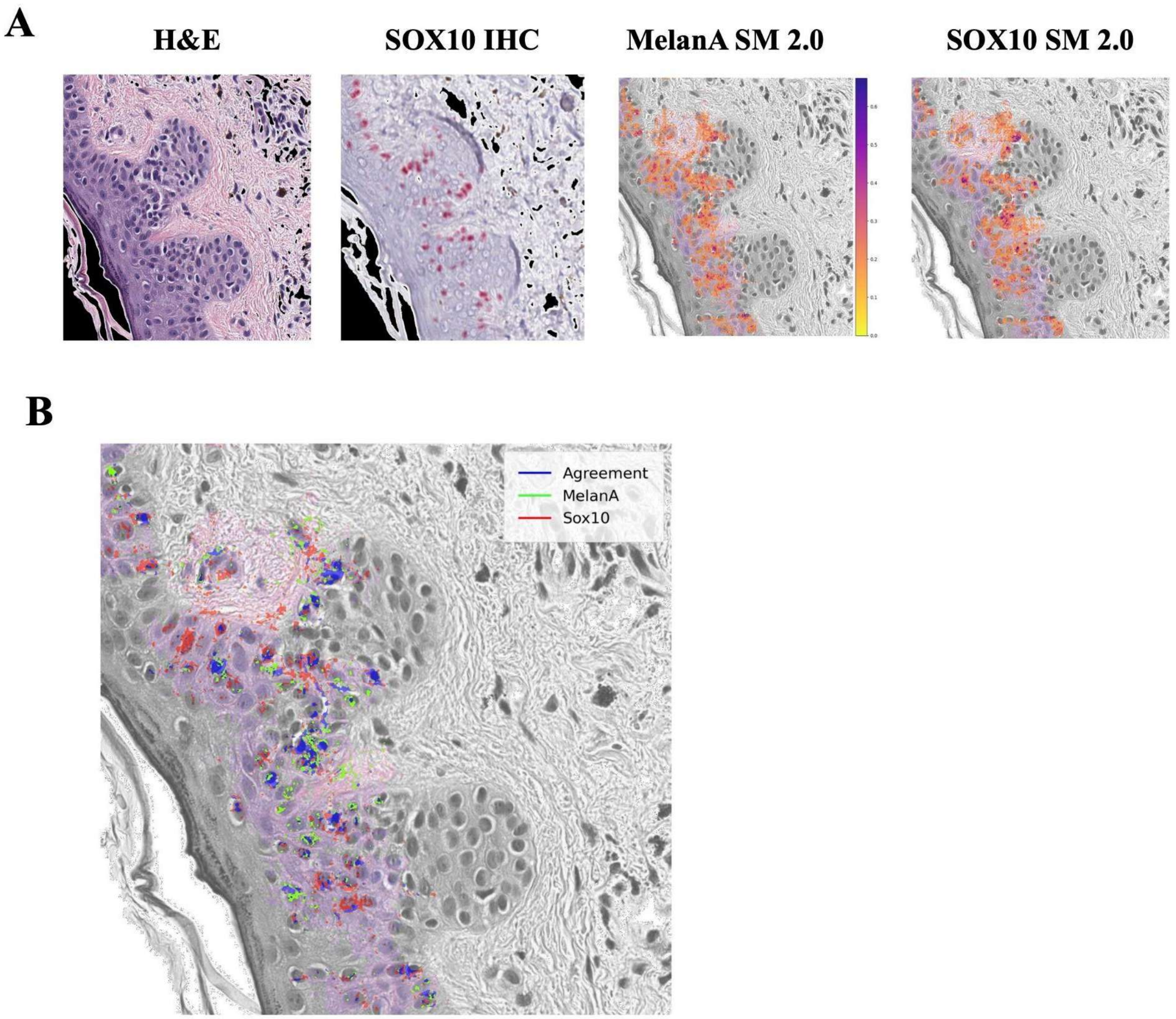

Figure S5. Recalculation of saliency maps for Figure 6b-c using a different attribution method, Integrated Gradients (IG), related to Figure 6 and STAR Methods. A) Saliency map comparison of MelanA and SOX10 models using IG. The IG method uses a different reasoning than Guided Grad-CAM to attribute salience to individual pixels, although it can be less intuitive when applied to images. Interestingly, the regions where independent IG calculations on each model converge on the same pixels (blue) qualitatively appear more coherent than those specific to either model alone (red, green). B) Agreement map comparing MelanA and SOX10 attributions on the same tissue region. Grayscale tissue denotes regions where the MelanA model confidence threshold is < 0.9. We only calculate attributions within the colored (high-confidence) region.

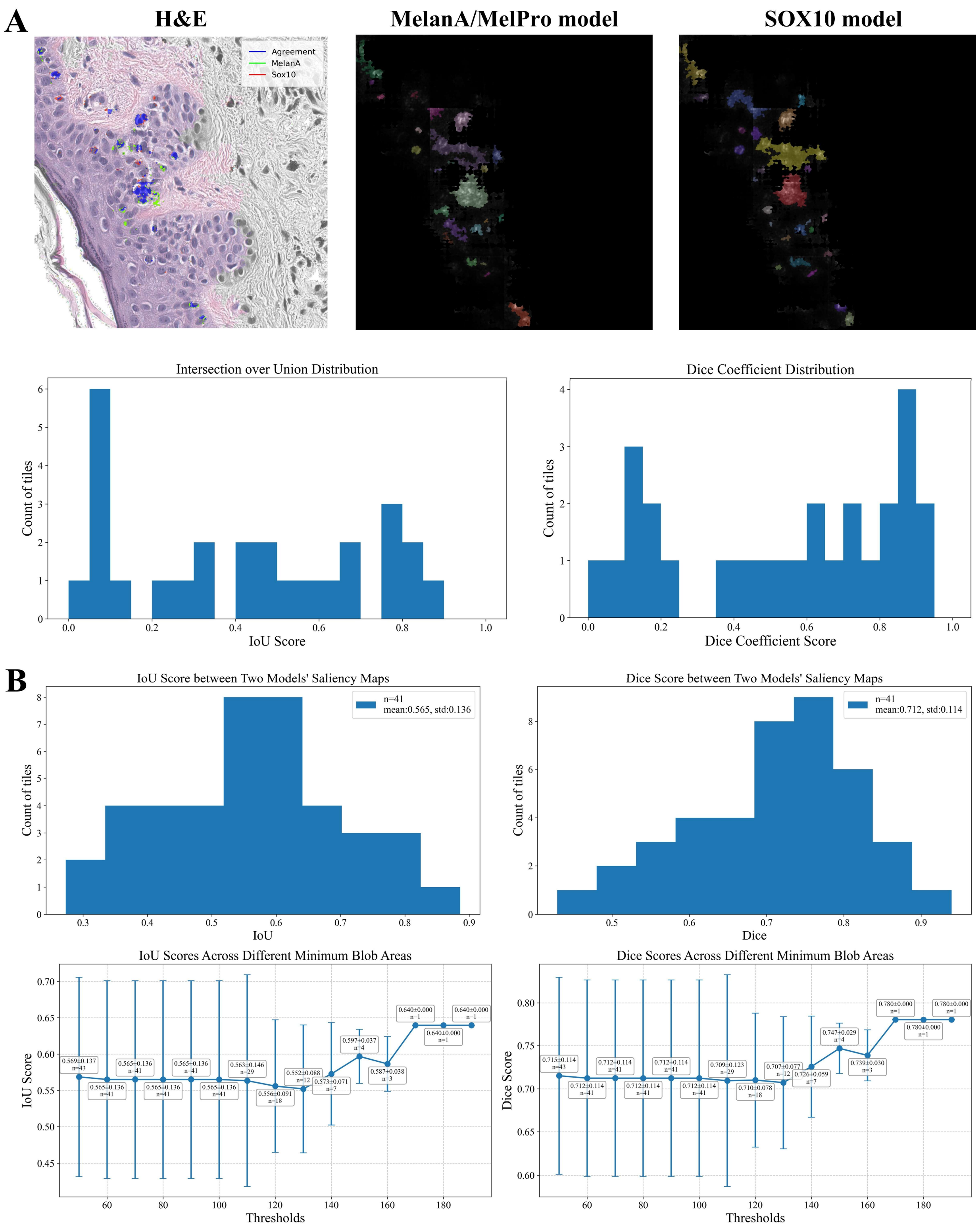
A
H&E
Agreement
MelanA
Sox10
MelanA/MelPro model
SOX10 model
Intersection over Union Distribution
Count of tiles
IoU Score
Dice Coefficient Distribution
Count of tiles
Dice Coefficient Score
B
IoU Score between Two Models' Saliency Maps
n=41
mean:0.565, std:0.136
Count of tiles
IoU
Dice Score between Two Models' Saliency Maps
n=41
mean:0.712, std:0.114
Count of tiles
Dice
IoU Scores Across Different Minimum Blob Areas
IoU Score
Thresholds
Dice Scores Across Different Minimum Blob Areas
Dice Score
Thresholds

Figure S6. Quantification of saliency maps similarity between MelanA/MelPro and SOX10 models at the region-of-interest, with an evaluation of different blob-size thresholds at the tile levels, related to Figure 6 and STAR Methods. A) Saliency map similarity at the region-of-interest. The region of H&E image with overlaid saliency maps (top left) from Figure 6c and visualization of individual saliency regions from MelanA/MelPro model (top middle) and SOX10 model (top right), with distinct colors representing distinct detected salient blobs. Histogram quantifying the similarity metrics between the two models' saliency maps. The bottom left panel shows the IoU distribution (0.428±0.286); the bottom right panel shows the Dice coefficient distribution (0.540±0.301). n = 27 blobs identified at ≥100 pixel area each. B) Histogram of IoU scores (top left, 0.565±0.136) and Dice scores (top right, 0.712±0.114) at tile-level between two models' saliency maps. IoU (bottom left) and Dice (bottom right) scores across different choices of minimum pixel-area threshold (50-190px) used to determine candidate cross-stain blobs.

SUPPLEMENTAL TABLES

WSI ID	Stain Type	Institution	# of Sections	Stain Color	Color Extracted
WSI-42	SOX10	UCSF	2	Red	Red
WSI-44	SOX10	UCSF	2	Red	Red
WSI-47	SOX10	UCSF	3	Red	Red
WSI-52	SOX10	Stanford	6	Red	Red
WSI-55	SOX10	Stanford	2	Red	Red
WSI-57	SOX10	Stanford	1	Brown	Brown
WSI-58	SOX10	Stanford	1	Brown	Brown
WSI-59	SOX10	Stanford	4	Brown	Brown
WSI-60	SOX10	Stanford	2	Red	Red
WSI-61	SOX10	Stanford	3	Red	Red
WSI-02	MELA	UCSF	4	Red	Red
WSI-03	MELA	UCSF	2	Red	Red
WSI-06	MELA	UCSF	7	Red	Red
WSI-07	MELA	UCSF	4	Red	Red
WSI-09	MELA	UCSF	1	Red	Red
WSI-12	MELA	UCSF	2	Red	Red
WSI-13	MELA	UCSF	3	Red	Red
WSI-14	MELA	UCSF	5	Red	Red
WSI-15	MELA	UCSF	1	Red	Red
WSI-16	MELA	UCSF	3	Red	Red
WSI-28	MelPro	UCSF	2	Red (cytoplasmic) + Brown (nuclear)	Red
WSI-30	MelPro	Stanford	1	Red (cytoplasmic) + Brown (nuclear)	Red
WSI-32	MelPro	Stanford	2	Red (cytoplasmic) + Brown (nuclear)	Red

Table S1. Overview of whole slide images used to train and evaluate the models, related to STAR Methods.

WSI ID	Stain Type	Institution	Sections (HE)	Sections (IHC)	Stain Color	Preview Notes	Action	Alignment Status
WSI-01	MELA	UCSF	6	6	Red	Tissue itself is strange. Previously removed	Not Used	Not Aligned
WSI-02	MELA	UCSF	8	8	Red	3rd level image damaged -- damage may impact alignment preview	Used	Aligned
WSI-03	MELA	UCSF	6	2	Red	6 slices compared to 2 rotated slices -- 3rd slice on top removed bc of bad alignment	Used	Aligned
WSI-04	MELA	UCSF	2	1	Red	Good -- can't preview due to huge size disparity -- going to assume at highest res it will work out -- **Have to manually add params	Attempted	Aligned Poorly
WSI-05	MELA	UCSF	3	3	Red	Iffy -- 3rd pairing is mismatch at equal sizing so removed -- other two may align, but not likely to very well	Not Used	Not Aligned
WSI-06	MELA	UCSF	8	8	Red	Good	Used	Aligned
WSI-07	MELA	UCSF	5	5	Red	Good -- keeping segment 1	Used	Aligned
WSI-08	MELA	UCSF	8	4	Red	IHC images on wildly larger scale than HE -- can't preview -- can't ECC align	Not Used	Not Aligned
WSI-09	MELA	UCSF	8	8	Red	Good -- skip first 2 smallest segments as they are difficult to align	Used	Aligned
WSI-10	MELA	UCSF	8	8	Red	Good -- remove bottom 2 segments as cannot ECC align between HE and IHC	Attempted	Aligned Poorly
WSI-11	MELA	UCSF	4	4	Red	Good -- likely too big for opencv -- skip for now and use previous alignments	Attempted	Aligned Poorly
WSI-12	MELA	UCSF	4	2	Red	Good	Used	Aligned
WSI-13	MELA	UCSF	12	12	Red	Good -- exclude bottom 2 segments by size -- removed section 10-9 HE-IHC for bad alignment	Used	Aligned
WSI-14	MELA	UCSF	8	8	Red	Good	Used	Aligned
WSI-15	MELA	UCSF	18	6	Red	Good -- removed bottom slice from each trio (total of 6 slices) due to bad segmentation	Used	Aligned
WSI-16	MELA	UCSF	6	6	Red	May not be able to align due to size disparity	Used	Aligned
WSI-17	MELA	UCSF	10	10	Red	Good -- ignore middle 2 slices for all pairings	Attempted	Aligned Poorly
WSI-18	MELA	UCSF	6	2	Red	Poor pairing -- can't preview -- likely can't align	Not Used	Not Aligned
WSI-19	MELA	UCSF	5	5	Red	Good -- remove 5<->5	Attempted	Aligned Poorly
WSI-20	MELA	Stanford	3	3	Brown	Good	Not Used	Aligned
WSI-21	MELA	Stanford	3	3	Brown	IHC sections are missing bottom half + angle disparity	Not Used	Not Aligned
WSI-22	MELA	Stanford	4	4	Brown	IHC sections are missing bottom half + angle disparity	Not Used	Not Aligned
WSI-23	MELA	Stanford	2	2	Brown	HE seg1 contains schlieren lines on right side -- Seg2 shapes different	Attempted	Aligned Poorly
WSI-24	MELA	Stanford	4	2	Brown	IHC seg2 needs to be resampled	Not Used	Not Aligned

WSI-25	MELA	Stanford	1	1	Brown	Bad IHC stain	Not Used	Not Aligned
WSI-26	MELA	Stanford	1	1	Brown	Good	Attem pted	Not Aligned
WSI-27	MelPro	UCSF	12	4	Red	Good	Not Used	Not Aligned
WSI-28	MelPro	UCSF	18	6	Red	Good -- bottom 3 IHCs are not attached to slide correctly -- applying only to top 3	Used	Aligned
WSI-29	MelPro	Stanford	6	2	Red	Good --alignments for pairs 1-3 may be difficult -- Copy in p16	Attem pted	Not Aligned
WSI-30	MelPro	Stanford	3	3	Red	Good -- Slight amount of IHC bottom missing in comparison to H&E	Used	Aligned
WSI-31	MelPro	Stanford	3	3	Red	Good -- IHC 2 may be treated as 2 segments instead of 1 (examine whether chroma 0. vs 1. is necessary)	Not Used	Aligned
WSI-32	MelPro	Stanford	3	3	Red	Good -- NA	Used	Aligned
WSI-33	SOX10	UCSF	3	2	Black	3 small H&E slices provided. 2 slightly larger IHC slices -- can't perform alignment preview	Not Used	Not Aligned
WSI-34	SOX10	UCSF	2	1	Black	2 small slices compared to 1 large rotated slice -- can't perform alignment preview	Not Used	Not Aligned
WSI-35	SOX10	UCSF	2	1	Black	2 small slices compared to 1 large rotated slice -- can't perform alignment preview	Not Used	Not Aligned
WSI-36	SOX10	UCSF	4	2	Black	4 slices compared to 2 larger rotated slices only -- can't perform alignment preview	Not Used	Not Aligned
WSI-37	SOX10	UCSF	2	2	Black	2 small slices compared to 1 large *broken* rotated slice -- can't perform alignment preview	Not Used	Not Aligned
WSI-38	SOX10	UCSF	2	1	Black	2 small slices compared to 1 large *broken* slice -- can't perform alignment preview	Not Used	Not Aligned
WSI-39	SOX10	UCSF	2	1	Black	2 small slices compared to 1 large rotated slice -- can't perform alignment preview	Not Used	Not Aligned
WSI-40	SOX10	UCSF	3	2	Black	Multiple slices compared to 2 separated portion of the same section --can't perform alignment preview	Not Used	Not Aligned
WSI-41	SOX10	UCSF	8	4	Black	Tissue is poor/choppy	Not Used	Not Aligned
WSI-42	SOX10	UCSF	3	3	Red	Good	Used	Aligned
WSI-43	SOX10	UCSF	8	4	Red	Good -- remove bottom slice tissue broken from IHC -- **WSI throws an error for align upon chroma change. Blurry IHC.	Not Used	Aligned
WSI-44	SOX10	UCSF	6	2	Red	Good -- All alignments are likely as good as they will get, but not optimal due to fundamental differences in shape between HE and IHC excisions	Used	Aligned
WSI-45	SOX10	UCSF	3	3	Red	Iffy -- most segments are too differently shaped / sized to incorporate -- May need to use original images rather than ECC	Not Used	Not Aligned
WSI-46	SOX10	UCSF	12	12	Brown	Good -- STAIN (GREY/BROWN) IS	Not	Aligned

						DIFFERENT THAN ORIGINAL LABEL. Negative control.	Used	
WSI-47	SOX10	UCSF	6	6	Red	Good -- does not perform well on ECC alignment	Used	Aligned
WSI-48	SOX10	UCSF	12	4	Red	Poor pairing and can't tell if slice is worthwhile	Not Used	Not Aligned
WSI-49	SOX10	UCSF	8	8	Red	Good	Attem pted	Aligned Poorly
WSI-50	SOX10	UCSF	6	6	Red	Good -- stain somewhat light	Attem pted	Aligned Poorly
WSI-51	SOX10	Stanford	3	3	Brown	Spurious -- Slices are huge and IHC stain is hard to separate from BG -- make sure masks are correctly matched	Attem pted	Aligned Poorly
WSI-52	SOX10	Stanford	3	3	Red	COPY of slices in MELA -- HE images seem like bigger area	Used	Aligned
WSI-53	SOX10	Stanford	4	3	Red	COPY of slices in MELA -- Could be hard to align if we don't fix angle disparity	Attem pted	Aligned Poorly
WSI-54	SOX10	Stanford	4	4	Brown	Good	Attem pted	Aligned Poorly
WSI-55	SOX10	Stanford	4	4	Red	Good	Used	Aligned
WSI-56	SOX10	Stanford	9	3	Red	Good	Attem pted	Aligned Poorly
WSI-57	SOX10	Stanford	1	1	Brown	Good -- See if difference in angle causes alignment to fail	Used	Aligned
WSI-58	SOX10	Stanford	1	1	Brown	Good	Used	Aligned
WSI-59	SOX10	Stanford	4	4	Brown	Good	Used	Aligned
WSI-60	SOX10	Stanford	3	3	Red	Good	Used	Aligned
WSI-61	SOX10	Stanford	3	3	Red	Good - Yellow smudge may cause problem with matches… make sure they are correct	Used	Aligned

Table S2. MelanA, MelPro, and SOX10 whole slide image dataset metadata and manual inspection notes, related to STAR Methods. Slides with action column “Used” were included in the calculations; “Not Used” and “Attempted” were discarded.

TCGA WSI SVS Filename	Tissue Source
TCGA-22-4594-01Z-00-DX1.3FCEBC89-8473-4841-87A2-F84AF58A7793.svs	Lung Squamous Cell Carcinoma
TCGA-2J-AABA-01Z-00-DX1.93B2B4EF-C302-4D00-ABE3-4862ACC81659.svs	Pancreatic Adenocarcinoma
TCGA-2J-AABK-01Z-00-DX1.AF5DE1FD-40EE-4149-8918-B53EC2DF727E.svs	Pancreatic Adenocarcinoma
TCGA-3A-A9I7-01Z-00-DX1.23EE4A93-A298-4522-837E-3EE10172D66C.svs	Pancreatic Adenocarcinoma
TCGA-3A-A9IB-01Z-00-DX1.77855A18-9E12-4F6A-8FBB-B5057656C493.svs	Pancreatic Adenocarcinoma
TCGA-3A-A9IH-01Z-00-DX1.578316D1-186E-4AE4-BD6A-DA426DE87829.svs	Pancreatic Adenocarcinoma
TCGA-3A-A9IL-01Z-00-DX1.BEB57CA5-223D-4330-BFFF-8202DCC857F3.svs	Pancreatic Adenocarcinoma
TCGA-3A-A9IN-01Z-00-DX1.A4FED037-D993-4F71-B422-14FC4E468B4C.svs	Pancreatic Adenocarcinoma
TCGA-3A-A9J0-01Z-00-DX1.322C8475-A1E3-4877-B3B5-921FDDB9698F.svs	Pancreatic Adenocarcinoma
TCGA-43-A56V-01Z-00-DX1.AA93FE03-FA7D-42C4-A118-B98C2400D9DA.svs	Lung Squamous Cell Carcinoma
TCGA-60-2722-01Z-00-DX1.f3781266-e8dc-4386-9702-5b29e6f2cfa3.svs	Lung Squamous Cell Carcinoma
TCGA-66-2742-01Z-00-DX1.8fdd6990-a08c-457b-80e4-586c619a784e.svs	Lung Squamous Cell Carcinoma
TCGA-A1-A0SE-01Z-00-DX1.04B09232-C6C4-46EF-AA2C-41D078D0A80A.svs	Breast Invasive Carcinoma
TCGA-A2-A04U-01Z-00-DX1.06D17357-46A8-4DC3-A22B-2F4EB6EE3F79.svs	Breast Invasive Carcinoma
TCGA-A2-A0CZ-01Z-00-DX1.A433A414-4F1B-4F99-8FD9-E64803F5E042.svs	Breast Invasive Carcinoma
TCGA-A8-A09R-01Z-00-DX1.392580F3-0CE5-4EDB-91CF-814AAD0DB649.svs	Breast Invasive Carcinoma
TCGA-AO-A0JC-01Z-00-DX1.C8DD421B-9799-4FE7-9224-5EAC6ED1028E.svs	Breast Invasive Carcinoma
TCGA-AO-A1KQ-01Z-00-DX1.CAB7D9A5-7030-4A33-BE51-9B04D67A7676.svs	Breast Invasive Carcinoma
TCGA-AR-A1AX-01Z-00-DX1.2389D54F-545E-499E-B392-DD731834460A.svs	Breast Invasive Carcinoma
TCGA-BH-A0DI-01Z-00-DX1.6A42D535-8842-4C36-8299-A40E9E56759D.svs	Breast Invasive Carcinoma
TCGA-D8-A1XS-01Z-00-DX2.ED8BBDB4-CEA6-4E47-8214-4666F3CC6E44.svs	Breast Invasive Carcinoma
TCGA-E9-A22D-01Z-00-DX1.b2867437-0add-4b7d-8002-fb09ed961942.svs	Breast Invasive Carcinoma
TCGA-F2-A44H-01Z-00-DX1.98C75E19-10DE-434A-AF1B-CDD182F6EDD5.svs	Pancreatic Adenocarcinoma
TCGA-F2-A7TX-01Z-00-DX1.2FB4B966-3F76-4BB7-B1E8-D6F651665479.svs	Pancreatic Adenocarcinoma
TCGA-FB-A4P5-01Z-00-DX1.D5440110-D217-4B4C-A8D2-7261B430F440.svs	Pancreatic Adenocarcinoma
TCGA-FB-A78T-01Z-00-DX1.1DC04A89-2428-489B-A70E-0D9C6D2A5E61.svs	Pancreatic Adenocarcinoma
TCGA-H6-A45N-01Z-00-DX1.80D3E1A9-02EB-4897-9632-F6FC00B3FA0F.svs	Pancreatic Adenocarcinoma
TCGA-HN-A2OB-01Z-00-DX1.14F1FBFB-4540-43CE-9D79-5BC628640424.svs	Breast Invasive Carcinoma
TCGA-HV-A5A4-01Z-00-DX1.00C72860-A4C4-41FB-87BA-7C4381FAF2BD.svs	Pancreatic Adenocarcinoma

TCGA-HZ-8005-01Z-00-DX1.e49bbccf-eab2-4f2f-b882-406b90fb2020.svs	Pancreatic Adenocarcinoma
TCGA-HZ-8315-01Z-00-DX1.F6B3F80E-3630-426E-AB2C-7F2EC5B63BFC.svs	Pancreatic Adenocarcinoma
TCGA-HZ-8317-01Z-00-DX1.BD28612C-D35D-4664-8B88-A85EF99013AB.svs	Pancreatic Adenocarcinoma
TCGA-HZ-8317-01Z-00-DX2.FDB366FF-AAA9-4FDC-A1F4-BA021904ED94.svs	Pancreatic Adenocarcinoma
TCGA-HZ-8637-01Z-00-DX1.5943021F-C94B-4CED-B45F-7A288F7188E0.svs	Pancreatic Adenocarcinoma
TCGA-HZ-8638-01Z-00-DX1.AD9F30CA-8943-493E-8603-7D1CF41056E6.svs	Pancreatic Adenocarcinoma
TCGA-HZ-A77O-01Z-00-DX1.C0F88C8F-C68C-457B-A0FF-3B483FCE7385.svs	Pancreatic Adenocarcinoma
TCGA-IB-7644-01Z-00-DX1.A2E77093-90D2-4ED3-90EB-F14A03C3DA57.svs	Pancreatic Adenocarcinoma
TCGA-IB-8127-01Z-00-DX1.C7035E56-9D24-4EEA-A09E-8276382193CC.svs	Pancreatic Adenocarcinoma
TCGA-IB-A5SS-01Z-00-DX1.899575C7-D239-4A04-8827-044F0D8868C8.svs	Pancreatic Adenocarcinoma
TCGA-IB-AAUV-01Z-00-DX1.045691CD-E0F8-4992-BF49-43AF7F83C97A.svs	Pancreatic Adenocarcinoma
TCGA-NC-A5HT-01Z-00-DX1.9295B0E3-37FE-4914-AFB3-78B56C893B6D.svs	Lung Squamous Cell Carcinoma
TCGA-OL-A97C-01Z-00-DX1.BDEEDEE2-6D07-4046-A8A9-D6FF8F337393.svs	Breast Invasive Carcinoma
TCGA-US-A774-01Z-00-DX1.522FF138-153F-488A-BBBC-5EA68EFD80C7.svs	Pancreatic Adenocarcinoma
TCGA-UU-A93S-01Z-00-DX1.C4809779-DF5F-4F5D-A78C-B7F95F2D050F.svs	Breast Invasive Carcinoma
TCGA-Z5-AAPL-01Z-00-DX1.30371C08-9075-44A9-8ED7-560256D65A7C.svs	Pancreatic Adenocarcinoma
**Tissue Source**	**WSI Count**
Pancreatic Adenocarcinoma	27
Breast Invasive Carcinoma	13
Lung Squamous Cell Carcinoma	5

Table S3. TCGA identifiers for SVS whole slide images and summary of tissue sources for negative-control TCGA non-skin H&E tiles used in model training, related to STAR Methods.

Fold	Train and Validation		Test	
	Positive	Negative	Positive	Negative
MelanA 1	16,214	355,753	1,025	20,924
MelanA 2	15,034	354,610	800	9,422
MelanA 3	13,102	317,536	1,225	20,614
MelanA 4	15,281	383,511	855	12,648
MelanA 5	12,712	329,430	1,505	16,186
Sox10 1	8,788	282,041	1,099	20,507
Sox10 2	8,758	333,967	958	8,926
Sox10 3	8,141	283,539	489	14,778
Sox10 4	10,278	283,860	407	5,454
Sox10 5	8,432	269,898	389	8,517

Table S4. Breakdown of positive and negative tile counts for each fold, related to Figures 2 and 3 and STAR Methods.
